# GenRiskPro: A Comprehensive Whole-Genome Sequencing Analysis Platform for Clinical and Wellness Applications

**DOI:** 10.34133/csbj.0011

**Published:** 2026-03-06

**Authors:** Xiya Song, Xinmeng Liao, Emre Green, Ozlem Altay, Hasan Turkez, Jens Nielsen, Minho Shong, Gözde Yeşil, Bayram Yuksel, Mathias Uhlen, Cheng Zhang, Adil Mardinoglu

**Affiliations:** ^1^ Science for Life Laboratory, KTH – Royal Institute of Technology, Stockholm SE-17165, Sweden.; ^2^ Department of Medical Biology, Faculty of Medicine, Atatürk University, Erzurum 25240, Turkiye.; ^3^ BioInnovation Institute, DK2200 Copenhagen, Denmark.; ^4^ Graduate School of Medical Science and Engineering, Korea Advanced Institute of Science and Technology, Daejeon, Republic of Korea.; ^5^ Phenome Omics R&D, Mehmet Ali Aydinlar Acibadem University, Istanbul, Turkiye.; ^6^ The Roger Williams Institute of Liver Studies, Faculty of Life Sciences & Medicine, King’s College London, London SE5 9NU, UK.; ^7^ Centre for Host-Microbiome Interactions, Faculty of Dentistry, Oral & Craniofacial Sciences, King’s College London, London SE1 9RT, UK.

## Abstract

Despite rapid advances in whole-genome sequencing (WGS), translating genomic findings into individualized insights remains challenging. We present GenRiskPro, a clinical decision-support and research platform, which automates WGS variant calling, annotation, prioritization, and reporting to deliver actionable findings and facilitate precision wellness. (To test the GenRiskPro platform, log on to https://www.phenomeportal.org/dashboard using the following credentials: Username: user@test.com; Password: test.) GenRiskPro integrates rare and common variant prioritization in a unified pipeline and in-house database, enabling both rare and complex disease and trait association analyses. Variant reporting is supported via LongevityCloud, which features a web portal for clinicians to review, adjust, and authorize the return of results in tabular and PDF formats, alongside a mobile app with artificial intelligence (AI) integration for sequenced individuals. Case studies using Turkish (TR, *n* = 275) and Swedish (SW, *n* = 101) WGS data assessed platform performance and variant prioritization: (a) predefined gene panels yielded a 1.82% positive rate for actionable findings per American College of Medical Genetics and Genomics (ACMG) secondary findings guidelines; (b) phenotype-driven support diagnosed cases including muscular dystrophy and microcephaly; (c) cohort-level ClinVar reassessment identified potentially misclassified pathogenic variants; (d) rare variant burden analysis revealed enrichment in ABCA4 for TR and SMPD1 in SW; and (e) population analysis highlighted carrier differences in trait-associated SNPs (rs12913832 and rs4988235) and PGx variants (CYP2B64 and CYP2B66). GenRiskPro unifies databases, literature, web development, and AI for rapid, user-friendly genomic analysis and reporting, which fosters collaboration among hospitals, researchers, clinicians, and patients.

## Graphical Abstract

**Figure:**
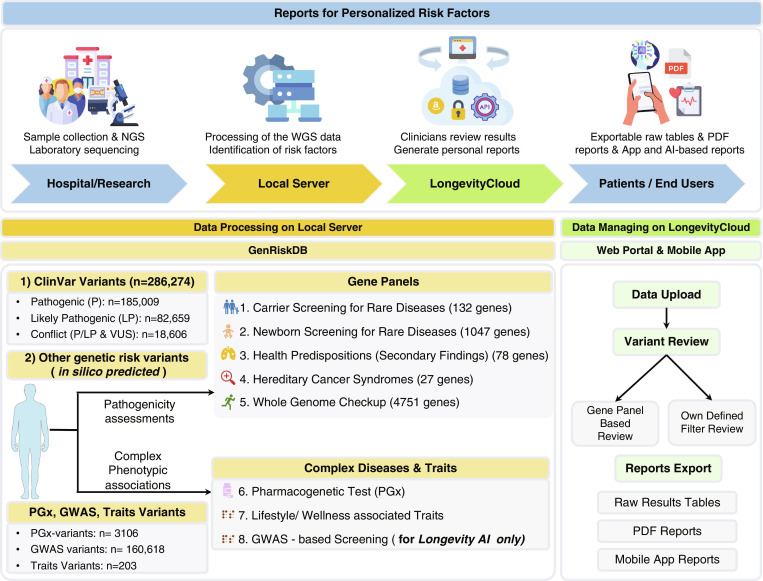

## Introduction

Whole-genome sequencing (WGS) identifies genetic variants across coding and noncoding regions [1] at an affordable cost, leading to diverse applications, such as population genomic studies [2–4], risk screening in healthy individuals [5], stratification of patients [6–8], clinical diagnosis in rare diseases [9,10], and precision medicine applications [11]. WGS has also been proposed as an alternative to traditional newborn screening methods such as tandem mass spectrometry [12]. Despite its growing accessibility, WGS adoption in clinical settings and wellness requires standardized automated pipelines for data analysis, interpretation, and reporting to deliver actionable insights and tangible individual health benefits [13].WGS studies in underrepresented populations, such as the Turkish (TR) population, are particularly valuable for addressing data imbalances in global genomic research [14–17]. Developing a comprehensive, end-to-end genomic sequencing pipeline represents a promising approach to addressing this data imbalance.

WGS scenarios for screening and diagnosis can differ substantially depending on the criteria for gene and variant inclusion. In this case, applying different curated gene panels following reporting guidelines are efficient tools for prioritizing WGS results based on the indication of the testing, ensuring that only selected promising results are reported back to individuals. Notable examples include the American College of Medical Genetics and Genomics’ (ACMG’s) secondary (incidental) findings (ACMG SF v.3.1) list [18] and prenatal carrier screening list of autosomal recessive (AR) patterns that consider the high carrier frequency among the population [19]. ACMG encourages the continuous reporting and sharing of variants among these lists with the community [20]. The BabySeq [21] project in the USA and hereditary cancer-focused studies, such as Tsaousis et al. [22], further demonstrated the utility of gene panels in various contexts.

Several computational platforms have been developed to report WGS data for different purposes. For instance, the Genome-to-Treatment, USA (GTRx) [23] and Karolinska-Rare Diseases (GMCK-RD, Sweden) [10] efforts present pipelines for accelerating the diagnosis of rare monogenic diseases. Clinical platforms, such as Varsome and Franklin, are designed for interpreting single variants or single cases, which limits their utility in large-scale preventive screening, wellness guidance, and complex disease risk profiling for healthy cohorts. Commercial genetic sequencing platforms, such as 23andMe, often focus on specific screening types or direct-to-consumer (DTC) wellness reports. Still, these platforms are typically not open-source, have limited and fixed variant targets, and lack access to their internal databases [24–26]. The GENCOV study [27] was a recent attempt to address this issue, but it lacked a digital platform for efficient visualization and communication with clinicians and patients. Some variant prioritization tools were developed, but they also only focused on clinical pathogenic variants, such as MutationDistiller [28] and UniVar [29], which contribute fewer insights toward healthy individuals.

Digital health is a prospering concept that emphasizes medical information exchange through electronic communications, with genomics being a growing pioneer in this field [30]. However, genomic reports integrated into interactive mobile applications remain scarce, despite their potential to enhance transparency and communication between clinicians and patients.

**Fig. 1.**
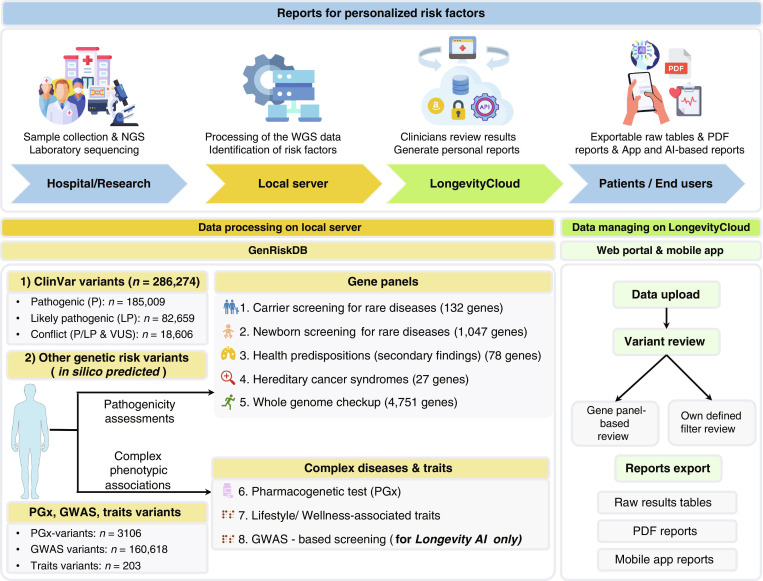
Overview of the design and content of the GenRiskPro platform. A WGS data processing platform for end-to-end WGS data analysis and personalized report generation for WGS-based risks was used. A curated database, GenRiskDB, was used to match the established genotype–phenotype database to reveal the genetic risk of rare diseases and complex diseases/traits. The prioritized outputs are accessible via a web portal for clinicians for manual curation before final delivery. The outputs include downloadable tabular files and adjustable PDF reports for all information for phenotypic prioritizations based on rare and common variants. These outputs assist in future clinical decisions, personal wellness improvements, and precision medicine applications.

In this study, we developed an end-to-end platform called GenRiskPro, a comprehensive WGS data-processing platform that integrates guidelines-based gene panel screening, rare disease variant interpretation, and complex trait-associated variant analysis. GenRiskPro enables comprehensive reporting for both research and return-to-individual contexts. Key features include (a) GenRiskDB, a comprehensive database used for variant annotations, interpretations, and prioritizations on the local server; (b) LongevityCloud, a cloud-based module that enables data uploading, analyzing and report generation, with functionality for manual variant review and refinement via a web portal, and delivery of genetic testing results to the patient via a mobile phone app (Fig. 1). A fine-tuned large language model (LLM), LongevityAI, is developed in the App for patients to interactively communicate their WGS-based reports.

To demonstrate its utility, GenRiskPro processed WGS data of a TR cohort (*n* = 275) enrolled in the first phase of the Anatolian Precision Medicine Initiative (APMI), and a Swedish (SW) cohort (*n* = 101) previously enrolled in the SW SciLifeLab SCAPIS Wellness Profiling (S3WP) study [31] was applied as another cohort, which, together with TR, served as performance tests and case studies for GenRiskPro applications.

## Results

### GenRiskPro: A comprehensive end-to-end WGS data-processing platform

We developed a WGS Risk Profiling tool (GenRiskPro), a comprehensive platform that enables efficient variant prioritization and reporting for large-scale cohorts, thereby supporting both clinical decision-making and multifaceted research initiatives. GenRiskPro generates 4 types of information: (a) clinical risk factors separated by gene panels; (b) pharmacogenomics (PGx) sections; (c) a wellness and lifestyle-associated traits section; and (d) GWAS-based complex trait profiles (Fig. 1). Each of these is provided as an independent, downloadable tabular file for easy access. With these results profiled by the GenRiskPro local server and GenRiskDB, we integrated analysis of phenotype-associated rare and common variants within the same pipeline, a departure from previous studies that focused exclusively on either rare or common variants [10,23,32]. Clinically associated variants and wellness-related indicators are partitioned into distinct sections within the GenRiskPro portal. This separation, combined with the platform’s clinician-gated access, ensures that nuanced counseling remains central to the diagnostic process and mitigates the risk of participant overinterpretation. For annotation databases that are regularly updated, the GenRiskPro platform incorporates an annual update to ensure that variant reports reflect the most current knowledge while maintaining version control for reproducibility.

A test user account with full authority over the entire system is provided in the main text and in Supplementary Material 1 for easy access to the functions in GenRiskPro. The user manual is available on the GenRiskPro web portal’s main page.

### Standardized workflow and databases on the local server for variant annotation and prioritization

**Fig. 2.**
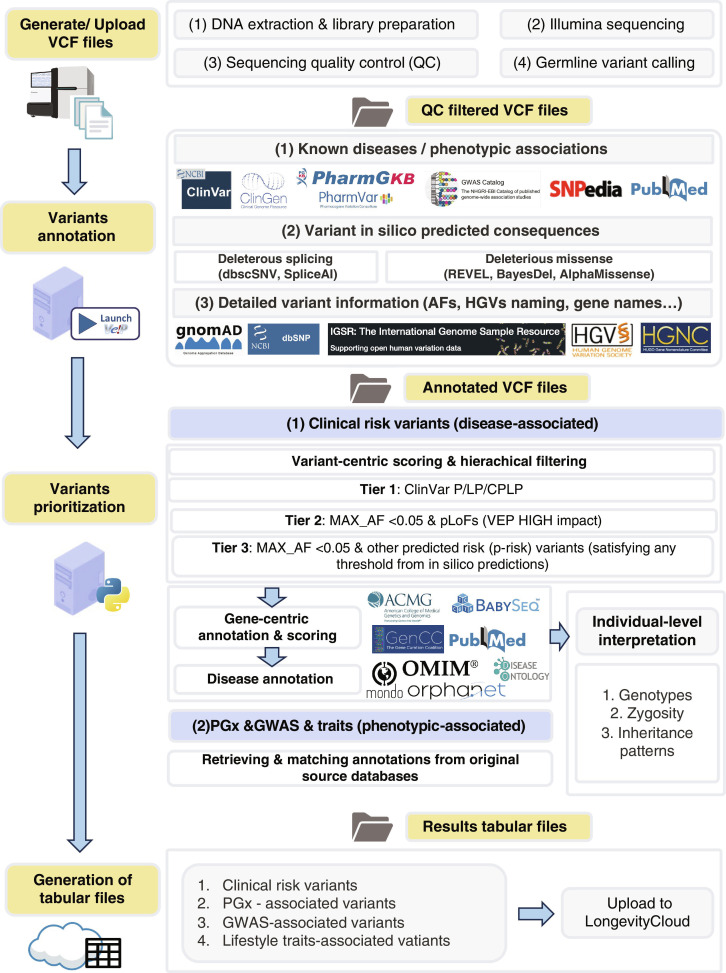
Schematic workflow of the GenRiskPro variant prioritization and annotation pipeline. The pipeline transforms raw genomic data into clinically actionable insights through 4 primary stages. (Top) Genomic data, originating from DRAGEN-called or user-uploaded QC-filtered VCF files, undergo systematic Variant Annotation against the integrated GenRiskDB. This process incorporates variant-centric scoring, population allele frequencies, and multialgorithmic in silico functional predictions (e.g., REVEL, SpliceAI, and AlphaMissense). (Center) During variant prioritization, a hierarchical classification logic is applied. Variants are triaged into clinical risk categories—comprising ClinVar-validated (P/LP/CPLP), predicted loss-of-function (pLoFs), and predicted risk (p-risk) variants, as well as PGx, GWAS, and lifestyle-associated traits. This stage integrates evidence from expert-curated databases (ClinGen, OMIM, and PharmGKB) and automated ACMG classifications. (Bottom) Prioritized variants are processed into structured Results Tabular Files, facilitating individual-level interpretation of genotypes, zygosity, and inheritance patterns. Final outputs are synchronized to the LongevityCloud, enabling secure retrieval and visualization via web and mobile interfaces.

GenRiskPro annotates and prioritizes variants on a local server with a customized workflow (Fig. 2). Variant calling is performed by the Dynamic Read Analysis for GENomics (DRAGEN) V3 Germline pipeline [32]. After generating or directly uploading quality control (QC)-filtered variants on Variant-Calling Format (VCF), variants are annotated against a comprehensive database, GenRiskDB, for variant-centric scoring and filtering. Beyond known phenotypic- or disease-associated variants (from ClinVar [33]), GenRiskPro annotates variants based on predicted molecular consequences, allele frequencies (AFs) from public databases, and in silico algorithm scores, including REVEL [34], dbscSNV [35], spliceAI [36], BayesDel [37], and AlphaMissense [38]. The pipeline also integrates established medical genetics resources, including expert-curated variants (ClinGen [39]), ACMG automatic classification tools (GeneBe [40]), and gene–disease-specific inheritance patterns (OMIM [41]). For clinical risk variants, after filtering for known benign variants, we categorized them into 3 hierarchical groups. First, variants with existing ClinVar records with pathogenic (P), likely pathogenic (LP), or conflicting (CPLP, defined as P/LP vs. VUS) were defined as ClinVar variants. Among the variants not presenting these records, those with “HIGH” molecular consequences by Ensembl Variant Predictor (VEP [42]) are classified as predicted loss-of-function variants (pLoFs). Finally, the remaining variants that were neither ClinVar variants nor pLoFs, but met thresholds across any in silico algorithms, are categorized as predicted risk variants (p-risk variants).

GenRiskDB is highly integrated, built on 14 established databases and the published literature, covering variant-centric, gene-centric, and disease/phenotype-centric information. A major effort is also being made to provide patient-friendly descriptions of all diseases and phenotypes, as well as identified variants.

On the variant-centric level, it included 286,274 clinically associated variant annotations from ClinVar [33] (Fig. S1), 3,106 pharmacogenetics (PGx)-associated variant annotations and star allele annotations from PharmGKB [43], 202 curated wellness/lifestyle-associated variant annotations from SNPedia and published literature curation (for 13 subcategories, including athleticism-associated [44] and personality/psychopathology-associated [45] SNPs), and 160,618 genome-wide association study (GWAS)-associated variant annotations from the GWAS Catalog [46] (Fig. 1). For PGx and trait analysis, the focus is on preserving all genotype-specific information pertinent to individual variations and their implications. Specific haplotype-based annotations were handled separately, including star allele assignments in the PGx section and APOE haplotype classification (ε2, ε3, and ε4 alleles) in the trait section, which are relevant to Alzheimer’s disease risk assessment. The corresponding genotype-based annotations are retrieved from GenRiskDB based on the sample’s specific genotype.

On the gene-centric level, GenRiskDB incorporates 5 predefined gene panels by collecting and aligning multiple gene panels defined by well-known literature resources [18,19,22,23,47–49] (Fig. 1 and Table 1). This includes (a) Health Predispositions, (b) Newborn Screening, (c) Carrier Screening, (d) Hereditary Cancer Syndromes, and (e) Whole Genome Checkup, including all the above genes along with expanded disease-associated or relevant genes from literature curation (Expanded Literature Diseases) and from the ClinVar disease database (Expanded ClinVar_ Diseases). In summary, the database comprises 4,751 genes associated with 6,836 diseases, resulting in 10,168 gene–disease pairs with disease ontologies categorized all diseases into 15 subtypes (Data S1), such as cardiovascular diseases, hematologic disorders, and immune disorders, among others. These pairs are classified by confidence level and intended application: high-confidence pairs (*n* = 1,589, with 1,082 unique genes) used in screening of asymptomatic individuals and broader moderate (*n* = 6,020, with 4,122 unique genes) or low-confidence (*n* = 2,567, with 1,551 unique genes) panels for phenotype-driven diagnostics. This confidence assessment specifically evaluated the appropriateness of reporting gene–disease associations within the designated intention of genetic testing. Gene Curation Coalition (GenCC) database classifications were used to compare against literature-derived confidence levels in GenRiskDB (Table S1) and were incorporated as additional annotation for each gene in GenRiskDB. The distribution of gene–disease relationships, inheritance patterns, and overlaps across different panels is presented in Fig. S2, with 52.7% of genes linking to single diseases, while others correlated with multiple diseases.

**Table 1.** Summary of literature-based gene panels integration for the GenRiskDB

Panel name	Data resources	Gene no.	Disease no.	Intention of testing	Gene–disease report confidence level
Health predispositions	ACMG secondary findings list (v3.1) [18]	78	84	Health screening for incidental findings during sequencing Identification of actionable adult-onset disease predispositions in asymptomatic individuals	High confidence
Newborn screening	1. Genes used by Babyseq projects (Categories A and B) [48] 2. Genes met the criteria from the GTRx platform (358 genes met GTRx criteria) [23] 3. Genes mentioned by Recommended Uniform Screening Panel (RUSP) [49] and list of neonatal screening in Europe [48]	1,047	1,231	Newborn infants’ health check, for childhood/early-onset diseases	High confidence (1 and 2) Moderate confidence (3)
Carrier screening	1. Carrier screening genes from “High-Carrier frequency” diseases provided by the ACMG list [19] 2. Carrier status reporting genes from the 23andMe list	1,063	1,287	Expanded carrier screening: heritable findings relevant to reproductive planning (carrier status) for diseases with recessive inheritance pattern	High confidence (2 and 3) Moderate confidence (several pairs from 1)
Heriditary cancer syndromes	27 genes associated with cancer syndrome by previous literature [22]	27	20	Screening for cancer syndromes that concern a higher possibility of developing cancers for specific individuals	High confidence
Expanded literature diseases	Causative genes resources for rare monogenic diseases [47]	2,471	3,073	Usually used for clinical diagnostics and research	Moderate confidence
Expanded ClinVar diseases	All genes related to any diseases from ClinVar database	3,194	2,625	Usually used for clinical diagnostics and research	Moderate confidence (casual) Low confidence (associations)
Whole-genome checkup	A combination panel integrated all genes from the above panels	4,751	6,836	A summary check for all the well-known disease-related genes, research for WGS full analysis	Mixed

On the disease-centric level, except for curated disease ontologies, patient-friendly descriptions for each disease were also obtained from Mondo, OMIM, and Orphanet databases.

GenRiskPro local server generates 4 tabular files per sample that are uploaded to LongevityCloud and serve as the foundation for PDF and mobile report generation. They can be exported in a standardized format (CSV) within a zip folder using the web portal. The clinical risk file contains variants categorized into different risk scores based on their potential pathogenicity by variant-centric and gene-centric assessments and flexible rules of disease name matching between ClinVar submissions and GenRiskDB (Table S2). We developed this categorization that aids in filtering variants in different scenarios, such as screening and diagnosis. Each variant entry includes comprehensive annotation comprising over 160 data contents, including risk scores, ClinVar review star ratings, submitted disease associations, ClinGen curation data when available, in silico prediction scores, and public AFs. The output also incorporates genotype information and gene–disease inheritance patterns, which are essential elements for consideration during the individualized reporting phase. The PGx file contains SNPs and haplotypes identified, along with associated drugs, annotations, evidence levels, and URLs. This mainly integrated the PharmGKB and PharmVar. The GWAS file includes significant variants (*P* < 5 × 10^−8^) with risk alleles, associated traits, and study accession numbers using the GWAS Catalog. The traits file contains in-house curated data on major lifestyle-affecting traits that could be beneficial for tested individuals, which was gathered from literature and SNPedia. All 3 files feature precise genotype handling with functional descriptions based on specific genotypes stored as a part of GenRiskDB.

### LongevityCloud: A reporting module for personalized genetic risk factors

**Fig. 3.**
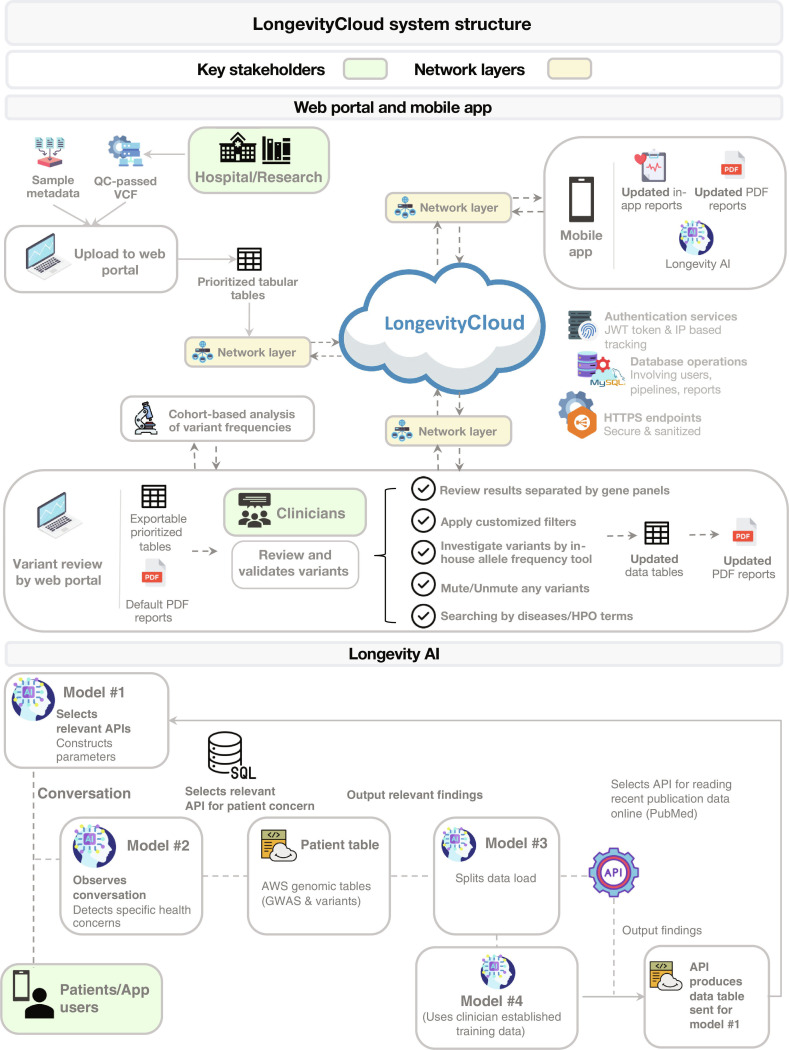
Overview of the LongevityCloud, including a web portal and a mobile app. LongevityCloud integrates 3 key stakeholders—hospitals/research facilities, clinicians, and patients—through a comprehensive module comprising a web portal and mobile application. The web portal serves as a variant review and reporting tool for clinicians and researchers, enabling bulk upload of VCF files, customizable variant filtering, in-house frequency investigation, disease/phenotype searches, and generation of rich-content PDF reports. A 2-tiered reporting system allows automated prefiltered reports and clinician-refined reports linked to both PDF and mobile outputs. The mobile application delivers patient-friendly reports, enhancing communication and supporting intervention planning. LongevityCloud also incorporates Longevity AI, a fine-tuned language model trained on genomic and clinical data, to help patients understand the relevant genetic findings and GWAS profiles.

We present LongevityCloud, a module that contains a web portal and a mobile application that facilitates collaborative engagement among 3 key stakeholders, including hospitals/research facilities, clinicians, and patients undergoing sequencing to advance precision medicine and digital health on a joint effort (Fig. 3).

The web portal serves as a variant review and curation tool for clinicians to generate an approved final PDF, also updating content from the mobile App for returning to patients. It is also applicable for researchers to generate tabular files and reports based on their sequencing files through file upload capabilities. The web portal features include the following: (a) an easily accessible page for bulk-uploading VCF files up to 5 samples a batch; (b) efficient VCF-to-tabular data processing; (c) a set of variant review tools, including filtering by custom gene/variant parameters, investigation by in-house AFs (with flexible calculation possibilities by selecting the projects involved, such as only TR, or only SW, or multiple-cohort together), enable/disable variants, and disease/phenotypic searching functions; and (d) rich-content PDF report generation. PDF report generation for sequenced individuals involves a 2-tiered processing approach. Firstly, LongevityCloud by default initiates an automatically generated PDF, which focuses on positive findings among adult-onset actionable diseases from the ACMG SF v3.1 list and carrier screening findings (Table S3). Secondly, the authorized clinicians can manually adjust filtering thresholds via the web portal, apply predefined gene panels or selected gene filters, enable/disable any variants, and regenerate refined reports (PDF and mobile app) that are linked with the web portal. The new enabled variants manually selected by clinicians will be added to the PDF under a new panel, “clinical selection”, or to the clinician-selected panels, such as carrier status. The initial PDF report has deactivated gene–disease pairs from newborn screening panels, as those are considered childhood-onset diseases.

GenRiskPro specializes in providing patient-friendly delivery of the genetic testing results. While the platform efficiently identifies and categorizes potential actionable variants, no findings are returned directly to the patient without clinician or genetic counselor review and confirmation. Using the web portal’s PDF generation system, all PDF and mobile reports for each individual can be generated in high throughput. These reports included explanatory figures, clear labels, and text descriptions to support translational genetics. A sample PDF report is attached to Supplementary Material 2**.** The mobile app retrieved information from the web portal and generated tailored reports, which were achieved by assigning sequencing results to the corresponding mobile app account. This further enhanced consultation efficiency and planning of potential intervention strategies. We also proposed Longevity AI, an LLM specifically trained to interpret and clarify complex genomic data. It operates within the data flow, accessing clinician-filtered patient data and GWAS profiles and supporting interactive conversations for promoting patient engagement and understanding of the testing results. The model is fine-tuned on a curated dataset that includes medical records, academic research, and specialized risk reports. This extensive training enables Longevity AI to answer questions posted based on an individual’s genetic profile from the secure database.

Due to the low health relevance and the minimal effect of single variant alleles, GWAS variants were omitted from the generation of direct reports of diseases and were rather considered a potential retrievable resource for each individual for facilitating precision medicine usage. Longevity AI reads the significant GWAS variants (*P* < 5 × 10^−8^ from the GWAS Catalog) based on the chosen phenotype/diseases as context to give answers and explanations of significant trait-associated SNPs. GWAS variants might be relevant to some rare diseases and also give more insights into complex traits and diseases, which suggests potential improvements in lifestyles. Some example questions and answers are shown in Fig. S3.

### Performance benchmark and variant prioritization outcomes

**Fig. 4.**
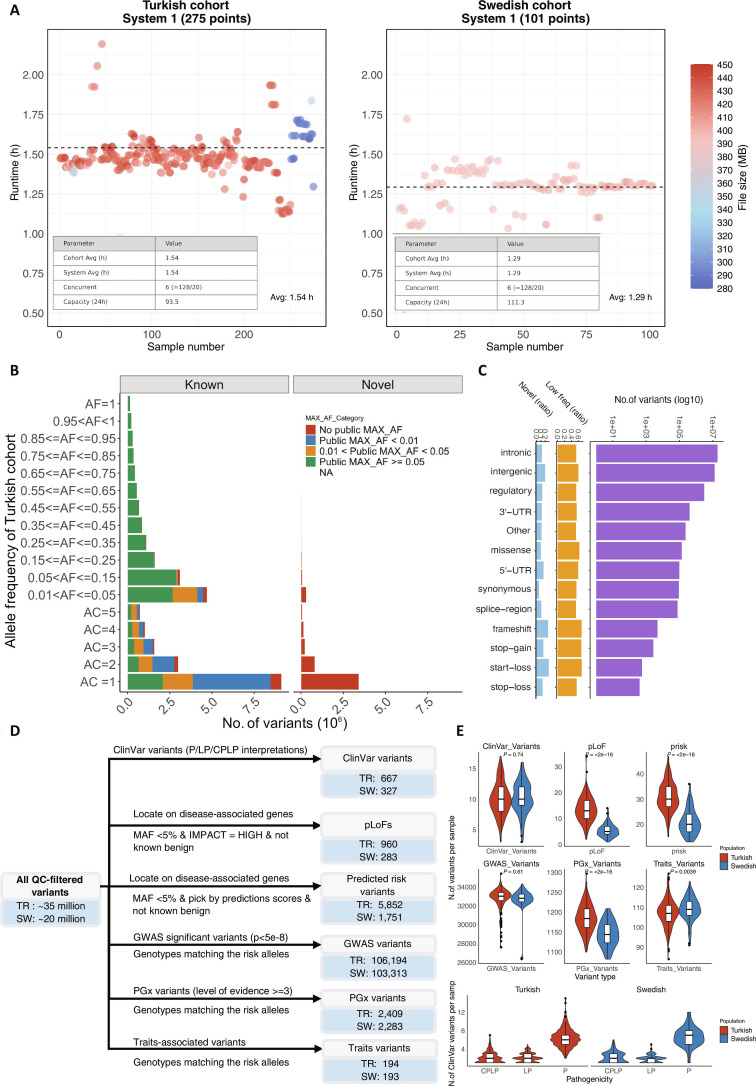
Running performance of the GenRiskPro local server and summary of variant prioritization results. (A) Sample processing times for 275 Turkish (TR) and 101 Swedish (SW) genomes on System 1. Average runtimes were 1.54 h (TR) and 1.29 h (SW), with file size indicated by color. (B) Count distribution of known and novel variants in the TR cohort, colored by public maximum allele frequencies. (C) The predicted consequences of all variants and the ratio of low-frequency and novel variants in each category of consequences. (D) Hierarchical prioritization of genetic risk indicators and summary counts for TR and SW cohorts across initial risk categories, including ClinVar P/LP/CPLP, pLoFs, and p-risk variants, as well as phenotypically associated variants (GWAS, PGx, and trait-associated variants). These counts represent initial genetic risk indicators before clinical interpretation. (E) Per-sample variant counts after GenRiskPro prioritization. The results demonstrate that phenotypic-associated genetic risk indicators are reduced to manageable counts at the individual level, facilitating downstream clinician review to identify clinically actionable findings. pLoFs and p-risk variants were more frequent in TR sequencing results than SW.

To demonstrate the ability, GenRiskPro was used to analyze the WGS data of an in-house TR cohort (*n* = 275) with an average sequencing depth of 36.79× (Fig. S4) in the first phase of the APMI and an SW cohort (*n* = 101) from the S3WP study. The performance of the GenRiskPro local server was evaluated on 2 local systems processing TR and SW cohort data, measuring end-to-end processing times from VCF input to structured tabular outputs. System 1 with HDD storage (Fig. 4A) is capable of processing per sample at an average of 1.54 h (92.4 min), using 128 total threads with 6 concurrent samples and a daily capacity of 93.5 samples. Notably, TR samples 251 to 275 were tested starting from uploading QC-filtered VCF files. System 2, under development as a beta version (Fig. S5), incorporates SSD storage and partial VEP memory loading. This demonstrated improvement of processing TR samples in 1.06 h (63.6 min), which is 30.9% faster, and SW samples in 0.89 h (53.4 min), supporting 19 concurrent samples (384 total threads) with a daily capacity of 435.9 samples.

From the merged multisample VCF sequencing outputs, we discovered approximately 35.8 million (35,725,453) distinct alternate alleles from the in-house TR cohort. The TR WGS data included 5.3 million (5,296,948) (14.8%) novel alternative alleles, most of which (65.6%) are singletons as expected. The AF of variants located on autosomes (34,510,548) in the TR cohort was calculated and is shown in Fig. 4B. We compared low-frequency (public MAX_AF <0.05) ratios and novel alternative alleles across 13 consequence categories (Fig. 4C). Notably, frameshift, start-loss, and stop-gain variants were more common among the low-frequency variants, with frameshift and start-loss variants also exhibiting higher proportions of novel variants. In parallel, we processed the WGS data for the SW cohort [31] (*n* = 101) using GenRiskPro’s bulk upload function and identified the potential genetic risk variants, as in the TR cohort. We found 10,653,536 (53.0%) singletons, rare, and low-frequency variants in the SW cohort, as shown in Fig. S6A and B. We detail the number of total detected variants grouped by AFs in both TR and SW cohorts (Table S4).

Figure 4D summarizes cohort-level variant counts from GenRiskPro’s all tabular outputs, which are categorized as ClinVar variants, pLoFs, p-risk variants, PGx variants, GWAS variants, and traits-associated variants as initial prioritization results presented for various applications. ClinVar variants matching GenRiskDB genes received higher scores, while nonmatching variants received lower scores. This filtering process retained 667 variants for TR and 327 for SW among all processed samples. Regarding pLoFs, 75.4% (TR) and 85.5% (SW) of them identified within genes in GenRiskDB were singletons. GWAS and trait variants mainly include common variants. Thus, most of them were identified in both TR and SW. Figure 4E demonstrates that GenriskPro’s filtering and prioritization pipeline effectively reduced clinical condition-associated variants to actionable quantities: ClinVar P/LP/CPLP variants (median *n* = 10 for both cohorts, with *n* = 6 in TR and *n* = 7 in SW for pathogenic variants specifically), pLoF variants (*n* = 13 in TR, *n* = 5 in SW), and predicted risk variants for further investigation (*n* = 30 in TR, *n* = 20 in SW). Beyond clinical conditions, we generated comprehensive individual genetic profiles including GWAS associations, PGx markers, and lifestyle-related traits. These profiles create a full view of genetic profiles for each sample, which will be used as input for Longevity AI. Comparing sequencing cohorts, the quantity of ClinVar, GWAS, and trait variants identified did not markedly differ between the TR and SW cohorts. TR samples exhibited higher counts of pLoFs, p-risk variants, and PGx variants than SW individuals.

### Case study 1: Standardized panel-based screening and analysis for clinical and research applications

**Fig. 5.**
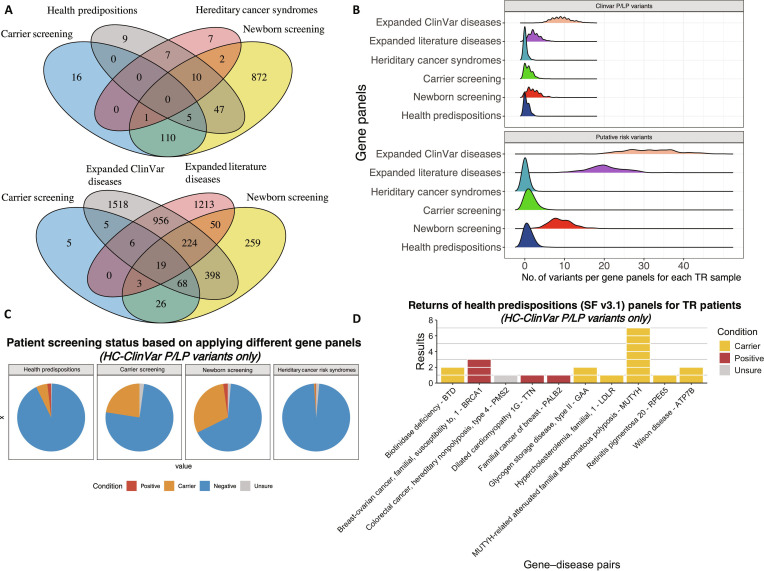
Conventional reporting strategies by applying predefined gene panels and example cases of secondary findings in the TR cohort. (A) Overlap of genes among carrier screening, newborn screening, and expanded ClinVar and literature disease panels. (B) Counts of prioritized variants per sample across gene panels. ClinVar P/LP variants are limited (0 to 5/sample) in screening panels, while putative risk variants, including pLoF and predicted risk (p-risk) variants, are more frequent, especially in expanded panels. (C) Classification of individual results (positive, carrier, negative, and unsure) based on high-confidence ClinVar P/LP variants and gene-specific inheritance patterns. (D) Classification of individual results of secondary findings in 78 genes recommended by the Health Predispositions (ACMG SF v3.1) panel identified in the TR cohort.

Standard gene panel screening represents a foundational genomic analysis approach used by both clinicians for patient screening and researchers for population studies. We implemented 5 predefined gene panels into the system based on literature reviews and several considerations. ACMG SF v.3.1 is used for the inclusion of incidental findings for actionable diseases and applied as basic health predisposition checks for phenotype-free individuals of any age, which includes the gene panel “Health Predispositions”. ACMG recommends reviewing and reporting potential pathogenic variants among these genes. The Newborn Screening panels primarily utilize genes proposed from various newborn sequencing projects, addressing a major concern regarding the early onset and curability of the associated diseases. Carrier Screening was designed as a list combining conventional AR genes with high carrier frequencies among populations, as proposed by the ACMG. The gene list of Hereditary Cancer Syndromes can be used for identifying an increased risk of hereditary cancers. Figure 5A illustrates the overlaps among the integrated gene panels. The upper Venn diagram shows the intersection of genes in 4 screening panels, with 110 genes shared between the carrier and newborn screening panels. The lower diagram demonstrates the intersection of 2 expanded panels (causative genes derived from literature [47] and disease-relevant genes from ClinVar) with carrier and newborn screening panels, revealing 3,687 known disease-associated or related genes outside these screening panels.

**Fig. 6.**
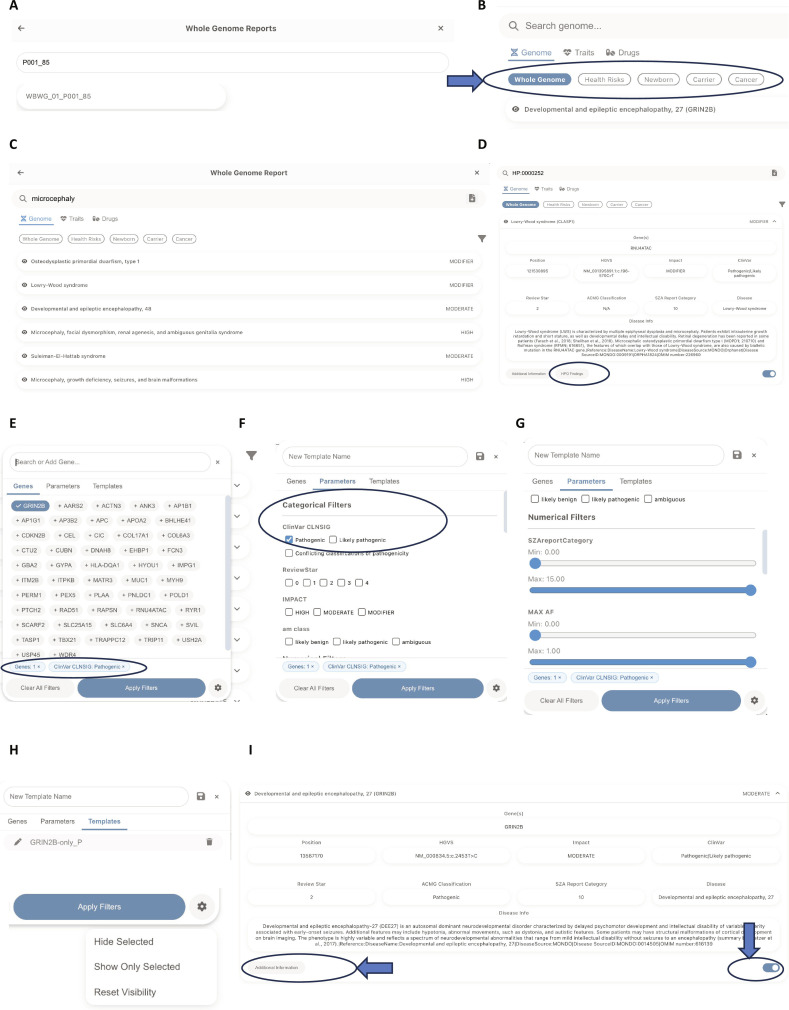
Web portal functions for facilitating variant review and customized diagnostic reporting for clinicians. (A) Sample search by name. (B) Variant filtering by predefined gene panels. (C) Disease-based search. (D) Filtering using observed HPO terms with expandable “HPO findings” details. (E) Gene name filter. (F) Categorical parameter filters. (G) Numerical parameter filters. (H) Saving filters as templates for batch actions. (I) Detailed variant review with enable/disable options. Clinicians use these tools to refine findings and generate PDF/mobile reports for patient return.

First, the panels are useful for easier filtering of variants on the web portal for various indication-based testing (Fig. 6B), organizing PDF reports (Table S3), and visualizing risk variants by subgroups on the mobile app (Fig. S7).

Second, GenRiskPro’s tabular outputs facilitate cohort-level identification of risk variants, particularly within predefined gene panels of interest to various studies. Figure 5B summarizes the amount of prioritized clinical variants across different gene panels, showing manageable numbers for focused review in each panel after processing. The upper panel shows ClinVar pathogenic/likely pathogenic (P/LP) variants, typically ranging from 0 to 5 per sample across all screening panels. The lower panel displays the amounts of pLoFs and p-risk variants, with higher counts per sample, particularly in the expanded panels.

The clinical tabular files enable flexible user-defined filtering based on column information to report high-confidence variants within specific gene panels, such as the ACMG SF list and the Newborn Screening list. At the individual level for variant conditions, we focused on high-confidence ClinVar P/LP (HC-ClinVar P/LP) variants that met the following criteria: public Max_MAF < 0.05; at least 2 review stars; not classified as benign/likely_benign by AlphaMissense and classified as pathogenic or likely pathogenic by the GeneBe automatic ACMG classifier. Based on this filter, we categorized individual results as positive, carrier, negative, or unsure according to gene–disease-specific inheritance patterns in screening panels (Fig. 5C). Regarding the TR cohort, we evaluated 78 genes using the gene panel “Health Predispositions”, which follows ACMG SF 3.1 guidelines (Fig. 5D). Applying this stringent filtering revealed 13 unique P/LP ClinVar variants in 20 of the 275 TR individuals from 18 distinct families (Data S2). According to gene–disease-specific inheritance patterns, 3 variants in 5 individuals (1.82%) were classified as having positive conditions (Fig. 5D). Additionally, 9 variants from 15 individuals return a carrier condition, and 1 variant for an individual returns an uncertain disease condition due to an unknown inheritance pattern in OMIM (PMS2-lynch syndrome 4, OMIM No. 614337) (Fig. 5D).

Using the same criteria, we detailed the strictly filtered P/LP variants detected within 1,082 high-confidence disease-associated genes from screening panels in the TR cohort (Data S3) and the SW (Data S4). Of these genes, 96% (1,043/1,082) are included in the Newborn Screening panel. In total, 150 unique P/LP variants were identified within 117 genes in 144 individuals, with each individual having been identified with mostly (88.2%) fewer than or equal to 2 P/LP variants. Similarly, a total of 80 unique variants within 71 genes were identified in the SW cohort. Although P/LP variants were detected, GenRiskPro classified most conditions as “Carrier” status, with only 13 positive out of 219 conditions in the TR cohort and 7 positive out of 107 in the SW cohort. These results offer valuable insights for subsequent research about population-specific genome screening projects.

### Case study 2: Personalized diagnostic workflows for supporting clinical decision-making

While standardized panels provide baseline screening, the diagnostic workflow requires deeper customization based on patient phenotypes. The following demonstrates how GenRiskPro facilitates this process for clinicians.

The web portal of LongevityCloud facilitates diagnostic reporting for clinicians through variant exploration and review functions (Fig. 6). We provided detailed guidance in GenRiskPro’s tutorial document accessible on the web portal. From original clinical risk variant files, a lenient filtering criterion is applied to preserve more potentially disease-relevant variants associated with 6,836 diseases from all panels. Firstly, the sample data can be found by searching for the sample name in the portal (Fig. 6A). Then, the web portal allows for an interactive review of variants using a set of tools: a predefined gene panel (Fig. 6B), disease names (Fig. 6C), or observed Human Phenotype Ontology (HPO) terms (Fig. 6D). Diseases matched to HPO terms are marked with an “HPO findings” tag, which users can expand to access detailed descriptions. Additional filtering options enable users to refine variant selection based on multiple criteria. These include gene name filtering (Fig. 6E), categorical parameters such as ClinVar review status (Fig. 6F), and numerical parameters such as in silico prediction scores, including SpliceAI (Fig. 6G). All user-defined filters can be saved as “Templates” for batch hiding or reversing selection based on the saved filter (Fig. 6H). To give full access to all annotations, the “Additional Information” is an expanded information page that allows viewing all 166 contents in detail, with a search box for quickly locating the column of interest. Lastly, the buttons on the right side allow clinicians to turn any risk variants on or off for the final report generation (Fig. 6I). Ultimately, clinicians can generate PDF or mobile reports of the disease-associated variants and return them to the patients. This approach is prevalently used in clinical settings, particularly for assisting clinicians in diagnosing genetically related diseases after observing symptomatic evidence.

We illustrated the potential value of investigating risk variants through a whole-gene disease database driven by a phenotype-based search approach, using 3 cases: 2 family-related females (P001_106 and P001_96) with muscular dystrophy and a 3-year-old boy with microcephaly (P001_85). Examples of phenotype-associated variants prioritized through disease name and description matching for these 3 patients are listed in Data S5.

Both females shared 2 known pathogenic CAPN3 variants that are phenotypically relevant; all with heterozygous status: a missense mutation, c.2338G>C (rs778768583, ClinVar ID 195641), and a stop-gain mutation, c.962G>A (rs1595828703, ClinVar ID 813987). Standard screening panels linked CAPN3 to AR limb-girdle muscular dystrophy type 2A (AR-LGMD2A), while expanded diagnostic panels also identified an association with autosomal dominant limb-girdle muscular dystrophy type 4 (AD-LGMDD4). This enables clinicians to reduce the broad investigation area into 2 scenarios: whether patients have AR-LGMD2A through 2 variants’ compound heterozygous mechanisms (high confidence) or AD-LGMDD4 from single variant effects (low confidence). For the 3-year-old boy with microcephaly, GenRiskPro identified a heterozygous pathogenic variant in a noncoding region, c. 196-570C>T (rs750325275, ClinVar ID 218082), for an RNA gene, RNU4ATAC, associated with microcephalic osteodysplastic primordial dwarfism, type I (MOPD I), which is an AR disease. This directly suggests the need for further evaluation of copy-number variation identification for RNU4ATAC. Further phenotype-driven screening was used to expand the potential candidate variants. Searching for “microcephaly” revealed that the patient is a carrier of 6 additional predicted risk variants. With no definitive positive findings, we then searched using another phenotype term, “intellectual disability”, and results returned a ClinVar pathogenic variant in expanded panels with GRIN2B c.2453T>C (rs879254016, ClinVar ID 245960), which leads to microcephaly in GRIN2B-related syndrome through autosomal dominant inheritance [50].

### Case study 3: Cohort-based research applications using downloadable prioritized variant lists

#### Re-evaluating ClinVar variants with cohort-based AFs

**Fig. 7.**
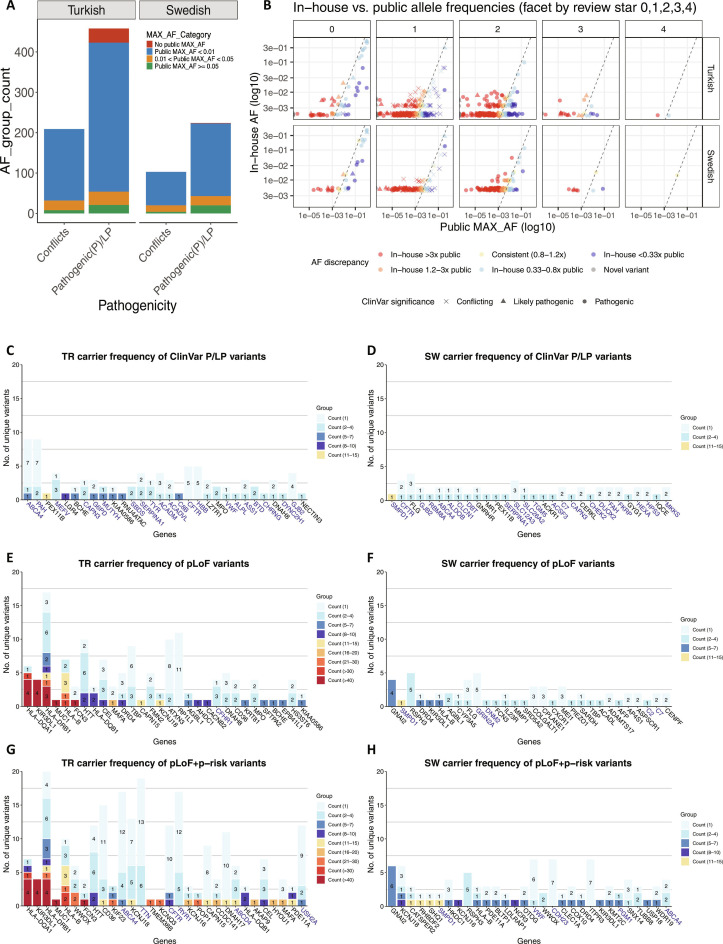
Downloadable tables of prioritized variants, enabling further review of clinically actionable findings and assessment of gene-level heterogeneity for genetic risk indicators from 2 cohorts. (A) Distribution of ClinVar variants identified in TR and SW cohorts, grouped by significance categories, with public Max_AF indicated. (B) Comparison of in-house allele frequencies and public Max_AF for ClinVar variants in TR and SW cohorts, shown by different ClinVar review star categories (0 to 4). (C and D) Top 30 genes with the highest carrier frequencies of ClinVar P/LP variants (public MAX_AF <0.05) in TR (C) and SW (D) cohorts; genes included in the high-confidence screening are shown in blue on the *x*-axis, and all other genes are shown in black. (E and F) Top 30 genes with the highest carrier frequencies of pLoF variants in TR (E) and SW (F) cohorts. (G and H) Top 30 genes with the highest carrier frequencies when combining pLoFs and predicted risk variants in TR (G) and SW (H) cohorts.

While pathogenic variants with high AF exist, their numbers are relatively limited. The variants submitted to ClinVar often include such cases, posing challenges in interpretation and validation. Cohort-based analysis could be a good resource to re-evaluate the ClinVar variants, especially in resolving variants with conflicting interpretations, and evaluating variants from the perspectives of public-database-underrepresented populations. According to our analysis of identified ClinVar variants (TR = 667, SW =327), we observed that both conflicting variants and P/LP variants exhibit a proportion of variants with public maximum AFs (public MAX_AF) over 0.05 (Fig. 7A). We then calculated in-house AFs for both the TR and SW cohorts and compared them with the maximum allele frequencies (MAX_AF) among public databases (Fig. 7B). ClinVar Variants with consistently high AF are suggested for re-evaluating the pathogenicity, as represented in the upper right side of each subplot. These variants were mostly with a low review star value of zero, with expectations. An example is rs7417106 (ClinVar ID 1320032) showing high AF in both the cohort and public databases. High discrepancy between the in-house cohort and public Max_AF may suggest cohort-specific findings, such as a CPLP variant rs751608665 (ClinVar ID 638508) that appeared at more than 3-fold higher than public Max_AF.

#### Rare variants’ gene-based burden characteristics across cohorts

GenRiskPro-generated risk variant profiles can be used to explore the enrichments of disease-associated genes by configurable selections of rare variants. This could serve as the first step of rare-variant selection for gene burden testing, an advanced methodology that examines associations between gene-based rare variant enrichment and complex diseases. Here, we showcased 3 selection strategies for rare/low-frequency (public MAX_AF <0.05) variants across the top 30 most prevalent genes identified in each approach: (a) ClinVar P/LP variants, (b) pLoF variants, and (c) pLoFs and all other predicted risk variants. We presented these results for the TR and SW cohorts, which included 1,082 high-confidence genes from 4 gene panels, highlighted in blue (Fig. 7C to H).

For ClinVar P/LP variants, ABCA4 was identified as the top prevalent gene with the highest carrier frequencies (16 individuals) (Fig. 7C). In the SW cohort, we identified SMPD1 with the highest P/LP carrier frequencies (11 individuals) (Fig. 7D). The frameshift variant of SMPD1 (rs756366019) was notably prevalent. The TR cohort exhibited greater diversity in pathogenic variants, with ABCA4 and PAH showing multiple P/LP variants (*n* = 9) across different frequency groups. In the SW cohort, FLG had the most unique P/LP variants (*n* = 4), followed by SLC12A3 and CFTR (*n* = 3). Next, we analyzed the 30 genes with the highest carrier frequencies of pLoFs (Fig. 7E and F) and combined pLoFs and predicted risk variants (Fig. 7G and H) across both cohorts. Despite the similar filtering and scoring, the composition of these pLoFs/predicted risk variants differed markedly between the TR and SW cohorts. For pLoF variants, HLA-DQA1 shows the highest carrier frequency in TR. In the SW cohort, GNAI2 demonstrated the highest carrier frequency. When combining pLoFs with predicted risk variants, HLA-DQA1 remained the most frequent gene in the TR cohort. Notably, WWOX emerged with increased prominence compared to the pLoF-only analysis, appearing among the top 6 genes, ranking higher than FCN3. In the SW cohort, while GNAI2 maintained its dominant position, KCNJ18 showed a substantial increase in carrier frequency as the second most frequent gene. In summary, we demonstrated the substantial gene-based heterogeneity between the 2 populations. We illustrated how the inclusion of low-frequency variants substantiallyaltered the comparative gene burden profiles across cohorts.

#### Exploring the population specificity of complex traits and pharmacogenomic predispositions

**Fig. 8.**
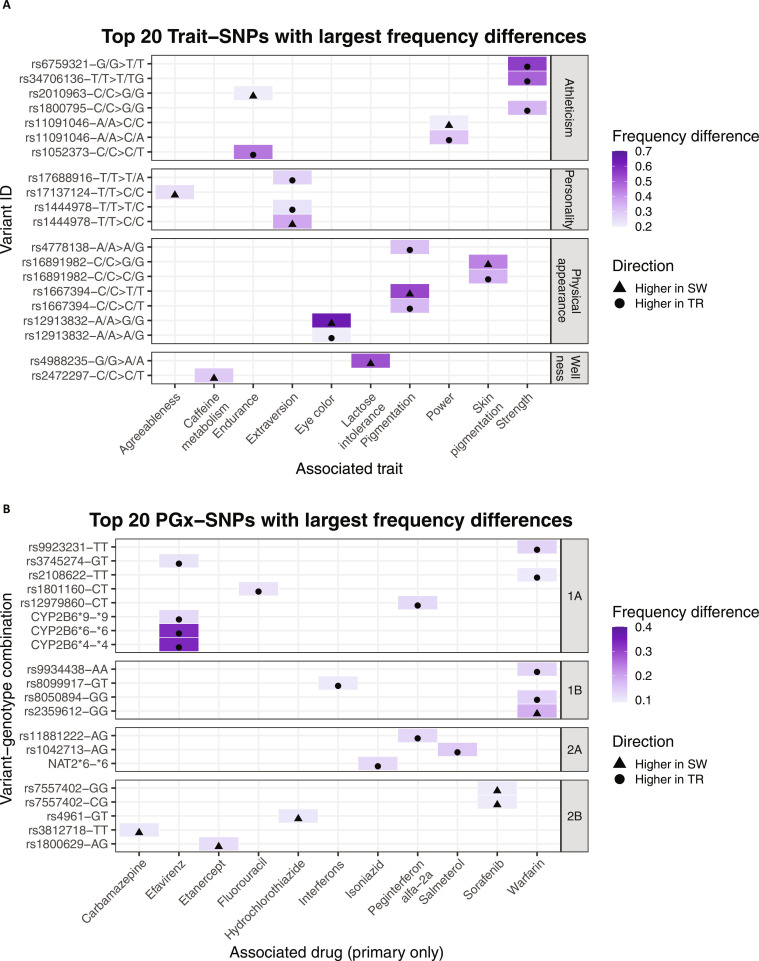
GenRiskPro for exploring genetic predispositions of complex lifestyle-associated traits and pharmacogenetic features. (A) Genotype-specific frequency differences in trait-associated SNPs between TR and SW cohorts with the most pronounced frequency difference. (B) Distribution of pharmacogenetic variants across 4 evidence levels (1A, 1B, 2A, and 2B) in TR and SW cohorts with the most pronounced frequency difference.

GenRiskPro integrates a comprehensive database matching to retrieve extensive, complex traits’ genetic predispositions. This could benefit the individualized lifestyle adjustments, guidance for medication usage, and potential public health decisions. In our analysis of lifestyle-associated traits across two cohorts, we observed expected findings. For examples, rs662799(G>A) were frequent in both TR and SW, which A allele (public MAX_AF>0.9) is associated with normal triglyceride levels and weight gain; the SW cohort exhibited high frequencies of rs16891982 (C>G) associated with skin pigmentation. Both cohorts primarily harbor the APOE ε3/ε3 haplotype, which is associated with a neutral Alzheimer’s risk. For comparison, Fig. 8A illustrates substantial genotype-specific differences in carrier frequency for trait-associated SNPs. The most pronounced difference was observed in rs12913832-A/A>G/G (eye color, 0.64 higher in SW), followed by rs6759321-G/G>T/T (strength, 0.55 higher in TR) and rs1667394-C/C>T/T (pigmentation, 0.53 higher in SW). Under the wellness category, the SW population exhibited higher frequencies in rs4988235-G/G->A/A for lactose tolerance (0.52 vs. 0.01 in TR), indicating greater ability to digest milk. This finding aligns with established research demonstrating high lactose tolerance in Northern/Nordic European populations from previous studies [51]. The top prevalent findings and general carrier frequency distributions of 13 different subcategories of traits for the TR and SW are presented in Fig. S8.

Regarding PGx findings, each individual was identified with a median number of 22 and 18 PGx variants (level of evidence 1A, 1B, 2A, and 2B) for TR and SW, respectively. NAT2 *4, SLCO1B1 *37, and NAT2 *5 were most frequent for both cohorts. Some PGx variants showed cohort-specific presence; for example, CYP2D6 *2 and CYP2D6 *4 were identified in the TR cohort but absent in the SW cohort. Figure 8B demonstrates PGx variant distribution across 4 levels of evidence (1A, 1B, 2A, and 2B) with the top 20 highest population carrier frequency differences, showing that TR exhibited more prevalence of CYP2B6 variants affecting efavirenz metabolism (CYP2B6*4 and CYP2B6*6, both approximately 0.33 higher in TR). Specifically, we evaluated several key PGx variants previously published in studies. The rs12979860 (CT/TT) within *IFNL3*, which impacts hepatitis C virus infection [52] and liver fibrosis [53], showed higher prevalence in TR (TR = 46.2%, SW = 33.7%). The rs9923231(CT/TT) within *VKORC1*, which is associated with warfarin dosage [54], showed a slightly higher prevalence in TR (TR = 48.7%, SW = 44.6%). The top prevalent findings and general carrier frequency distributions of PGx findings for the TR and SW are presented in Fig. S9. A full list of comparisons of trait and PGx (level of evidence 1A, 1B, 2A, and 2B) carrier frequencies is available in Data S6.

In summary, we showcased GenRiskPro’s usability for clinicians, patients, and researchers. It allows clinicians to apply different strategies to report the variants and balance the benefits of retaining all potential risk variants against the risk of overreporting. GenRiskPro could also be applied in research to revise variants’ pathogenicity based on cohort-level AFs and identify ethnic-specific genetic variant burdens by population-based comparison.

## Discussion

Genomic medicine, with its advances in precise genotyping and genome-based risk reporting, had the greatest impact on personalized medicine to date [55]. Our GenRiskPro platform represents a significant step toward this direction, offering an end-to-end solution for WGS data processing, prioritizing, and reporting. To suit the diverse needs of clinicians, patients, and researchers, the establishment of such a platform requires a concerted effort across different domains, including genomics, bioinformatics, software development, data science, and regulatory compliance. This progress necessitates streamlined data processing, management, and effective communication with stakeholders. GenRiskPro enables systematic generation, management, and analysis of genomic data, ultimately translating scientific insights into tangible individual health benefits. This iterative process involves feedback from end-users to ensure that the GenRiskPro platform meets the needs of genomic researchers and addresses the challenges of large-scale genomic data.

As shown in our case studies, GenRiskPro uses LongevityCloud to integrate specialized databases and dynamic filtering for analyzing genetic risk factors, balancing the benefits and risks of genome testing and reporting with categorized panels, variant risk scores, and detailed content information. In this way, it offers an approach for flexible and customizable report generation, focusing on high-confidence or phenotypically relevant variants, while maintaining low-confidence or nonphenotypic risk variants as a record for data retrieval in case any abnormal health condition is present in a later stage. These records, including a full profile of GWAS-based risk variants, may be used to illuminate the interfamilial variability and genetic heterogeneity of the diseases, which is currently not clearly known.

Since the high turnover speed of sequencing results is particularly crucial for diagnosing acute genetic diseases [23], especially when it applies to newborn screening projects, LongevityCloud module significantly expedites report generation and accessibility of first-hand data for clinicians and patients. This report system suggests a combination of automated generation and clinical adjustments to satisfy the need for timely delivery of the results. A mobile app with artificial intelligence-enhanced interactive communication further enhanced the speed and the efficiency, which is also invaluable for translating medical findings back to patients. These reporting methods collectively underscore the potential benefits of early sequencing in rare disease diagnosis and proactive health management.

We also emphasized the necessity and benefits of running and managing genetic sequencing projects on a unified platform. By summarizing cohort-level findings and cohort-specific AF through a standardized pipeline, GenRiskPro enables systematic assessment of variant pathogenicity and reveals genetic features specific to the corresponding cohort. For example, ClinVar, as a major submission-based variant–disease association database, leads to different interpretations of the same variants with unclear details, which poses the need for variant curation. As of 2024, Clinical Genome Resource (ClinGen) [39] has created a total of 5,722 expert-curated variants across 92 genes, representing just 0.2% of ClinVar’s total records. In our cohort, we investigated the AF of ClinVar pathogenic (P), likely pathogenic (LP), and conflicting P/LP variants. Some of these variants showed high AF (>0.05) in both public and our cohorts, suggesting further review of their pathogenicity. We also found cohort-specific variants with much higher AF than public data, which is notable when considering reporting pathogenic findings for the specific sequencing cohort. Overall, our data showed that a TR individual or an SW individual carries a similar median number of ClinVar variants (10 per person). This result parallels the reported number from another TR genomics study [14]. We revealed that most ClinVar pathogenic variants or pLoF variants show low penetrance in our cohorts, which is in line with large biobank studies (UKBB and BioMe) that discovered less than or equal to 5% risk difference for individuals with the variant allele [56]. This perspective is further supported by recent large-scale biobank research [57], which demonstrates that the population-level penetrance of certain pathogenic variants may be considerably lower than traditionally assumed, ranging from 9.4% to 28.1% for specific inherited conditions. Identification of these variants is rather useful when the phenotypes are present, for example, in our case studies of diagnostic reports. Pathogenic variant identification via WGS is specifically useful for rare diseases and contributes to the successful diagnosis of approximately 30% to 50% of suspected rare disease patients [58]. In the current version of GenRiskPro, we have not been able to directly provide the penetrance, expressivity, and age-related risk of variants. However, clinicians may use the hyperlinks from ClinVar and OMIM for further investigation and apply the in-house reference panel to investigate the in-house AFs of certain variants among all analyzed samples. In summary, our study proved the low penetrance of most of the ClinVar pathogenic and pLoF variants, providing a reference for curating the pathogenicity of ClinVar and pLoF variants, especially for the underrepresented populations.

A common consensus is that genomic studies should increase the diversity of underrepresented populations outside European descent, which is the major ethnic group of genomic studies [59]. The WGS data generated from ATPM projects will accelerate the understanding of underlying evidence when interpreting specific variants from a population-specific view, especially for ACMG-relevant secondary findings. We illustrated this by exploring the genetic heterogeneity between TR and SW, especially for their rare variant burdens and PGx variant prevalences.

As a specific feature compared to other platforms, GenRiskPro simplifies the generation of genetic profiles that affect personal medicine intake and lifestyle improvement, as a new step toward precision medicine and digital health. These common variants have been well-studied and proven to benefit personal wellness. Regarding PGx variants, GenRiskPro focused on the effort of including as many known PGx targets as possible, which could serve as a good complement for previous PGx callers that were limited to several genes, such as PharmCAT [60] and Stargazer [61]. All the individuals carried approximately 21 actionable (1A/B, 2A, and 2B) PGx variants. Similarly, a previous evaluation of ~ 43,000 individuals was carried out, who all carried at least one variant within 16 actionable PGx-associated loci [62]. Well-known PGx SNPs that impact health decisions (such as rs12979860 and rs9923231) are identified and described by GenRiskPro outputs. These findings provide additional evidence of the possible benefits of performing a PGx evaluation together with WGS to implement the pharmacogenetic guidelines into practice [63]. In addition, some of the lifestyle-associated SNPs showed substantially differences, such as metabolic specificity of lactose among different cohorts. Altogether, GenRiskPro’s wellness reporting provides new insights into the use of predisposition sequencing in healthy individuals, which might accelerate individuals’ positive reactions to possible medical or lifestyle changes, as suggested by the PeopleSeq project [64].

This study is not free of limitations. De novo variant identification by trio-data or compound heterozygote analysis is still under development because of a lack of phased data. While GenRiskPro serves as a clinical decision-support tool and facilitates research in clinical genomics, the clinical risk variant module is not intended to function as a validated diagnostic system. Diplotype-predicted phenotypes of PGx variants were not solved in GenRiskPro’s PGx section, while GenRiskPro only generates clinical annotations based on SNPs and matched PGx haplotypes. The limited sample sizes (*n* = 275 and *n* = 100) of our 2 cohorts, especially the TR cohort with family relationships, might not unbiasedly reveal the carrier frequencies and in-house AFs of variants. A larger sample size is being accumulated by the GenRiskPro platform during this project, which will provide additional resources for further biological insights.

## Methods

### Recruitment of study participants in the TR cohort

A total of 275 TR individuals (154 males, 121 females) from 180 distinct families were recruited between January 2022 and June 2023 in the first phase of the APMI. At APMI, a total of 10,000 healthy individuals and patients diagnosed with >150 different conditions (25 cancers, 100 rare, and 25 complex diseases) will be recruited. Biological specimens, including blood, urine, saliva, and fecal samples, and tissue (where clinically feasible and consented), will be collected between 2022 and 2025. While the broader APMI initiative aims to generate multiomics data (e.g., transcriptomics and metabolomics), the current study focuses exclusively on WGS data derived from blood-based germline DNA.

The first phase of the TR cohort consisted of 272 individuals without known genetically heritable diseases. Additionally, 2 individuals from the same family self-reported muscular dystrophy, and one individual was diagnosed with microcephaly.

All study participants were informed that the purpose of the study was to evaluate personal health risks associated with genomic variants. The participation in this study was voluntary and without exclusion criteria. All the participants signed the written informed consent in this study. The personal identifiers of each participant were recorded, including family relationships, gender, self-reported chronic disease, drug consumption, smoking, alcohol consumption, height, and weight.

### Acquisition of WGS data for the TR cohort and SW cohort

Blood samples were collected from all 275 individuals to perform WGS analysis. Venous EDTA blood samples (5 ml) were obtained from each participant and transferred to a sequencing laboratory using a transport box chilled (4 to 10 °C) with ice packs. To purify total genomic DNA from human whole blood samples, an automated extractor, QIASYMPHONY (Qiagen, Germany), was used. The manual extraction of nucleic acids was conducted for the samples for which the isolation kits were not compatible with QIASYMPHONY. The quality and quantity of DNA samples were initially assessed spectrophotometrically with NanoDrop (Thermo Fisher, USA). Before the library prep, gDNA samples were quantified more precisely fluorometrically with the Qubit Broad Range dsDNA quantitation kit (Waltham, MA, USA). From the gDNA samples (*n* = 275), PCR-free NGS libraries were constructed with the Illumina DNA PCR-free Prep kit with the input of 300 to 400 ng. The sequencing was performed on the Illumina NovaSeq 6000 platform. Raw sequence data in Binary Base Call (BCL) format were demultiplexed and converted to FASTQ with DRAGEN Software v3.9.5. Paired-end reads were then aligned to the NCBI reference sequence (GRCh38) by using the Burrows–Wheeler Aligner (BWA). The variant calling was performed by the Germline Small Variant Caller from the Dragen server V3 (Illumina) with the default hard filter applied. The MultiQC reports were generated to evaluate the sequencing quality.

WGS data of the SW cohort were generated as part of the S3WP study [31]. The methods of sample collection and sequencing were described in the original paper. The SW cohort included 101 individuals without known genetically heritable diseases.

### WGS outputs of the TR cohort

WGS of the 275 TR individuals revealed an average sequencing depth of 36.79× (Fig. S4), with an average coverage of 0.96. Following standard quality filtering by DRAGEN, a typical TR WGS sample contained a median of 4.98 million variant sites, with 4.79 million located on autosomes. This variant count is higher than the median numbers observed in the East Asian and European (EAS and EUR, approximately 4.7 million) populations, comparable to the American population (AMR, around 4.8 million) and lower than the African (AFR, around 5.8 million) population, as presented in the high-coverage 1KGP3 project [2]. The transition/transversion (Ts/Tv) ratios calculated for each WGS sample ranged from 1.99 to 2.00, aligning with the typical range for the human genome.

### The integrated components and technologies for creating GenRiskPro

The specification of technical details and components for GenRiskPro was outlined in Supplementary Methods and Fig. S10, including the architectural design, integration strategies, technology stack, testing procedures, and user support measures employed in the process of platform development. These components collectively enable efficient data collection, storage, analysis, interpretation, visualization, and automated reporting of GenRiskPro with its web portal and mobile app.

### Construction of GenRiskDB with customized variant annotation and prioritization

To analyze the effects of the variants identified in individuals, we first constructed a gene panel-based database by collecting gene–disease relationships from established literature, clinical guidelines, and resources listed in Table 1. A full list of genes was provided in Data S1. Genes were organized into panels according to the intended clinical application and source of collected gene–disease pairs. Specifically, high-confidence pairs were derived from authoritative and guideline-backed panels, such as the ACMG secondary findings list, BabySeq recommended genes, and established hereditary cancer gene lists, which are considered appropriate for screening in asymptomatic individuals. Moderate-confidence pairs were collected from broader clinical databases and literature-based panels, including expanded rare disease genes and comprehensive ClinVar gene–disease mappings, suitable primarily for phenotype-driven diagnostics and research. Low-confidence pairs included provisional or association-level gene–disease relationships, such as those flagged as “association only” in ClinVar and broader panels without consensus clinical endorsement. Lastly, GenCC classifications were added to GenRiskDB as additional information for each pair. All diseases were categorized into 15 subtypes, such as cardiovascular diseases and immune disorders, using records from disease ontology databases, including MONDO and disease ontology (DO), complemented by manual curation for diseases without sufficient information. Lastly, the inheritance patterns of all gene–disease pairs were from the OMIM database.

Several variant-based resources were used in the compilation of a genotype-specified annotation consisting of the following:

1. Clinical reported variants from the ClinVar database (release 20240611). This included pathogenic, likely pathogenic, and conflicting pathogenic variants (CPLP), with submissions of pathogenic and uncertain showing up together.

2. Pharmacogenetic actionable variants with specific genotype annotations from the PharmaGKB (release 20240812) and PharmaVar (release 6.1.2). By integrating these 2 databases, 3 major sub-pgx-databases were created. A haplotype reference database was created by extracting entries containing star allele notations from the haplotype resource files. For each haplotype, associated rsIDs were compiled by parsing variant identifiers, establishing connections between star alleles and their component SNPs. Two complementary annotation dictionaries were developed: one for SNP–genotype associations indexed by rsID and genotype combinations, and another for gene-haplotype associations indexed by gene-star allele designations. This approach ensures thorough pharmacogenomic assessment, capturing both SNP-level and haplotype-level variants with clinical relevance to drug response. However, this SNVs/Indels-based assessment may have limited sensitivity in resolving complex PGx regions, such as CYP superfamily genes, which harbor copy number variants and gene rearrangements/deletions/duplications.

3. Genome-wide significant GWAS variants from the GWAS Catalog (release 20240727). We extracted disease/trait associations, genomic locations, genes, SNP identifiers, effect sizes, study accession numbers, and PMID. The dataset was refined by excluding multiloci associations, retaining only GWAS-SNPs with “rs” identifiers and defined risk alleles, applying a genome-wide significance threshold (*P* < 5 × 10^−8^), and requiring a nonempty value for quantitative effect estimates (OR/BETA).

4. Lifestyle–wellness-associated traits databases (own-curated). We collected common SNPs that are used in DTC counseling, literature, and SNPedia. The genotype-specific effects of these common SNPs were manually curated based on evidence. APOE ε2, ε3, and ε4 alleles were processed specifically by combining genotypes from 2 SNPs (rs429358 and rs7412) to report the risk of Alzheimer’s disease.

Additional resources were incorporated as further reference when considering the pathogenicity assessment of the prioritized list from GenRiskPro, including ClinGen curated variant information (release 20141209). In silico prediction tools, including dbscSNV (version 1.1), spliceAI, REVEL, BayesDel, GeneBe automatic ACMG classifier, and AlphaMissense, were downloaded and used as additional resources to assist in the pathogenicity assessment of variants.

### Processes of single-sample variant interpretation

The workflow of variant interpretation is described in Fig. 2. VCF files were generated from the WGS data, and hard filters were applied. The small variants that passed the hard filters were annotated by VEP (v113). During the annotation, the custom variant database was used as input to map and store all the variants specified in the variant database, and the variant filtration process was skipped. Variant public frequency data were retrieved from the GnomAD v2.0, v3.0,v4.0, and 1000G projects, and the maximum AF was used as the final public AF for the site. GenRiskPro retains all ClinVar variants (P, LP, and CPLP) during analysis. The code matches these variants to corresponding disease associations in GenRiskDB through direct disease identifier mapping and text-based disease name comparison. For variants not included in the variant database, the hard filter of the maximum global AF of 0.05 (MAX_AF <0.05) was applied. Several in silico prediction tools were subsequently applied to predict their pathogenicity. Firstly, pLoFs were defined by the “HIGH” impact classification from VEP annotation, which included frameshift variants, splicing site variants, and start/stop loss variants. Apart from those annotated by VEP, potential splice-altering variants were further assessed using dbscSNV for splicing consensus regions and SpliceAI for noncoding regions. Low-frequency variants with a dbscSNV ada_score (AdaBoost) or an rf_score (random forests) higher than the optimum cutoff of 0.6, or any of the 4 spliceAI scores higher than the recommended threshold of 0.5, were considered to affect downstream mRNA splicing potentially. Additionally, the deleterious effect of missense variants was evaluated based on REVEL, BayesDel, and AlphaMissense. Variants with a REVEL score greater than 0.75, BayesDel_addAF_score greater than 0.0692655, or a BayesDel-noAF_score higher than −0.0570105, or Alphamissense classified as “likely_pathogenic” were identified as potentially deleterious missense alterations. These thresholds were selected to maximize the sensitivity and specificity of predicting pathogenicity. The rationale and performance of the in silico thresholds (REVEL, BayesDel, AlphaMissense, dbscSNV, and SpliceAI) were evaluated through a benchmarking analysis against a curated ClinVar dataset (Supplementary Materials 3). A scoring table was used to define and sort the variants identified in individuals (Table S2). Known pathogenic variants associated with disease-causing genes were assigned a high genetic risk score, whereas the variants with conflicting pathogenicity associated with disease-associated genes were assigned a lower genetic risk score.

### Cohort-level all detected variant summary counts

The VCF outputs from each individual in the TR and SW cohorts were merged into a multisample VCF by BCFtools merge modules while setting the missing alleles to reference alleles. Multiple-allele sites were split into biallelic sites and left-normalized using the BCFtools norm module. Functional annotation of the cohort-level variant set was retrieved by VEP (v109). The AF of each biallelic variant was calculated by the VCFtools freq module. The novel variants are defined as the variants with empty coexisting known variants, that are compared with public variant repositories imported from the Ensembl Variant Effect Predictor (VEP, v113) including gnomAD [65] (Exome v2.1.1, Genome v3.1.2 and Genome v4), 1000 Genomes Project phase 3 [16], and dbSNP (v154) [66].

### Implementation and governance of LongevityAI

LongevityAI utilizes an open-weight 120B parameter LLM and is implemented as an agentic orchestration layer rather than a free-form generative system. To ensure data integrity and minimize hallucination risks, the model is restricted from direct access to raw genomic data or unstructured reports. Instead, the agent translates natural language user intent into specific parameters for predefined, deterministic analysis pipelines. LongevityAI then retrieves and summarizes the structured, static outputs generated by GenRiskPro pipelines, such as variant categories, inheritance patterns, and standardized annotations. The model, therefore, does not generate novel interpretations or infer results beyond the structured GenRiskPro outputs. Operationally, LongevityAI is strictly nondiagnostic and nonprescriptive, with logs maintained for systematic auditing and human expert review. It is explicitly prohibited from providing medical advice or treatment recommendations.

### Regulatory and ethical compliance

GenRiskPro is designed as a clinician-gated decision-support and research platform and does not function as a stand-alone diagnostic system. Its regulatory status under frameworks such as CE-IVDR or FDA Software as a Medical Device (SaMD) is subject to specific jurisdictional requirements and intended deployment contexts.

Data governance is enforced through UUID-based encryption and strict access controls, ensuring that datasets are accessible only to authenticated owners. Data sharing or linkage across accounts is disabled by default and requires explicit user action via cryptographic keys. The platform adheres to GDPR principles, including data minimization, purpose limitation, and user-controlled access. Ethical oversight, including informed consent and re-contact policies, is managed at the clinician and institutional levels, with GenRiskPro serving as the underlying secure infrastructure layer.

## Ethical Approval

Ethics approval of all human samples and data collection in the Turkish cohort was given by the Medical Faculty of Atatürk University, Ethical Committee for Clinical Research, Erzurum, Turkey, dated 2021 December 30/No:45. Ethics approval of all data included in this study and all analysis performed was given by the Regional Ethical Review Board in Stockholm, Sweden (ethics permit numbers 2024-00954-01).

## Data Availability

Source code for VCF processing is available at GitHub: https://github.com/xiyasong/Genomics_platform. A representative subset of 100 original VCF samples and the full raw tabular outputs from the pipeline, including genetic risk (for major rare and complex diseases, mapped using ClinVar and in silico predictions scores), PGx, GWAS, and traits, are deposited in Zenodo (DOI: 10.5281/zenodo.16409257) with open access. The complete WGS dataset in VCF and the raw FASTQ format are accessible upon request.
