# Supplementary material for: GenRiskPro: A Comprehensive Whole-Genome Sequencing Analysis Platform for Clinical and Wellness Applications
Source: Comput Struct Biotechnol J. 2026 Mar 6;35(2):0011. doi: 10.34133/csbj.0011 (PMC13394978; doi:10.34133/csbj.0011)
Supplement: Supplementary 1 — Figs. S1 to S10 Tables S1 to S4 Data S1 to S6 [file csbj.0011.f1.zip › Supplementary Figures.pdf]

Supplementary Figure 1

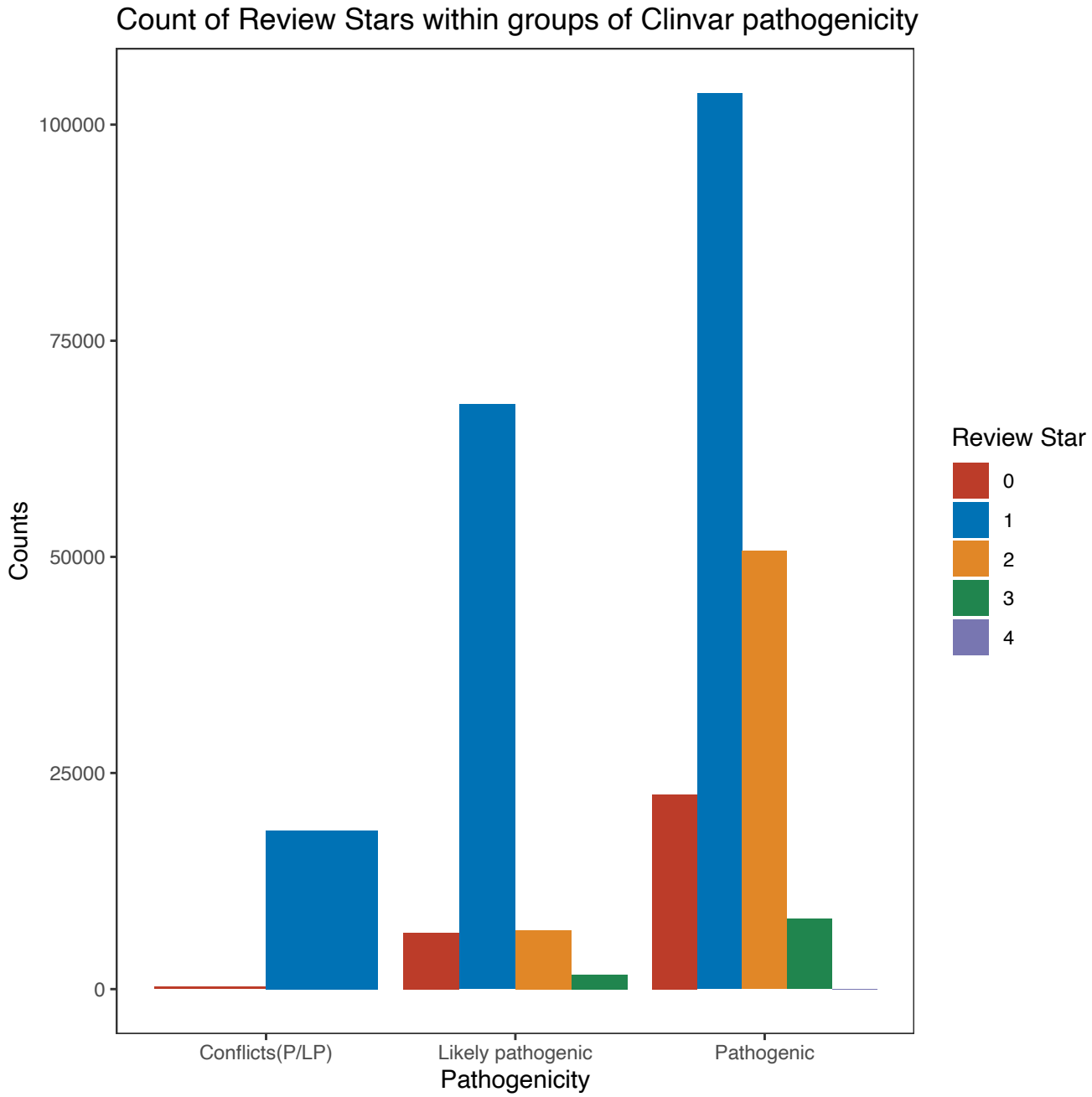

Supplementary Figure 2

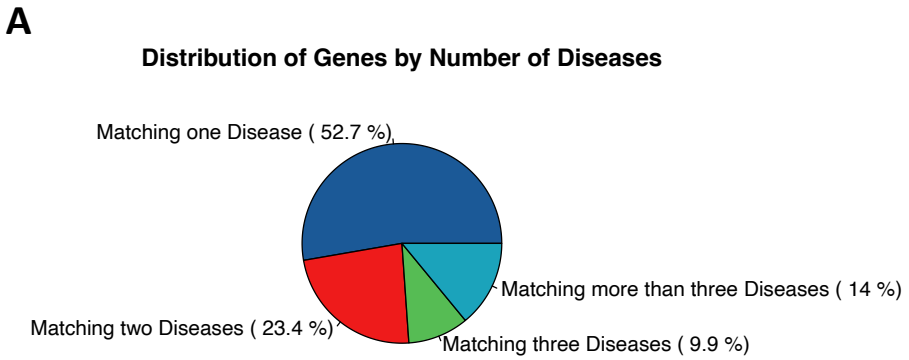

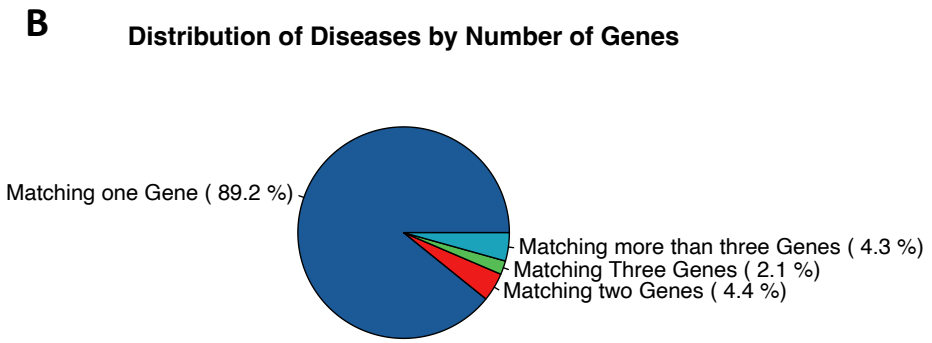

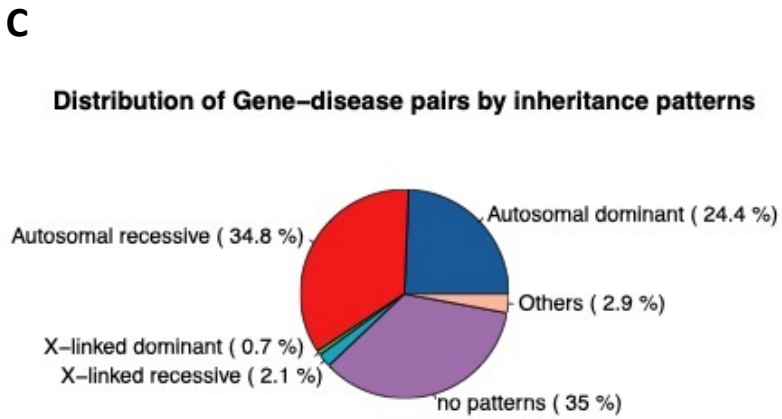

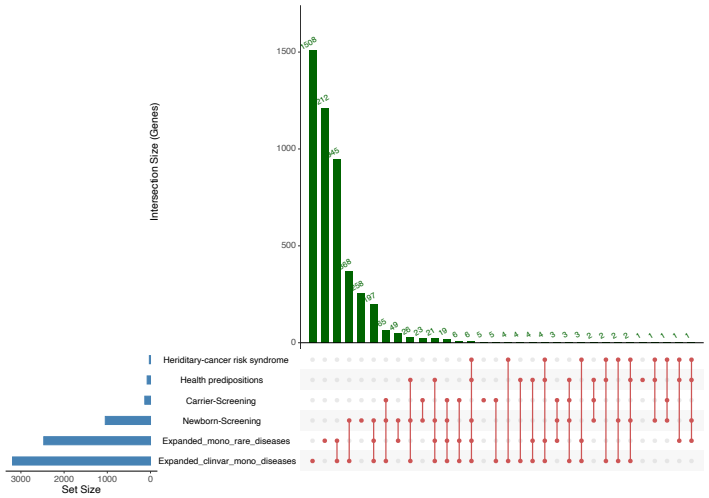

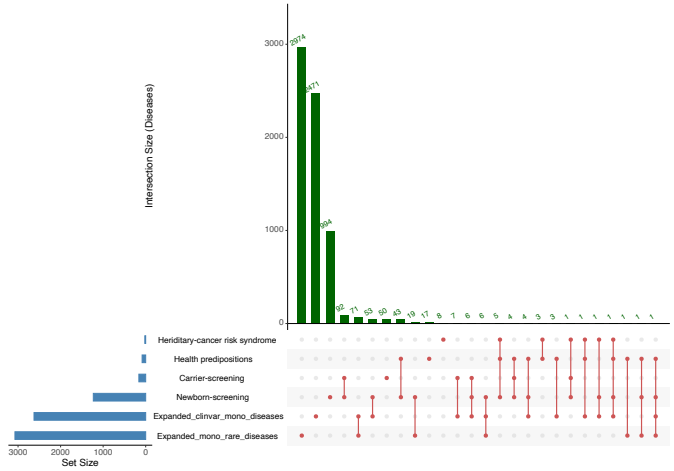

Supplementary Figure 3

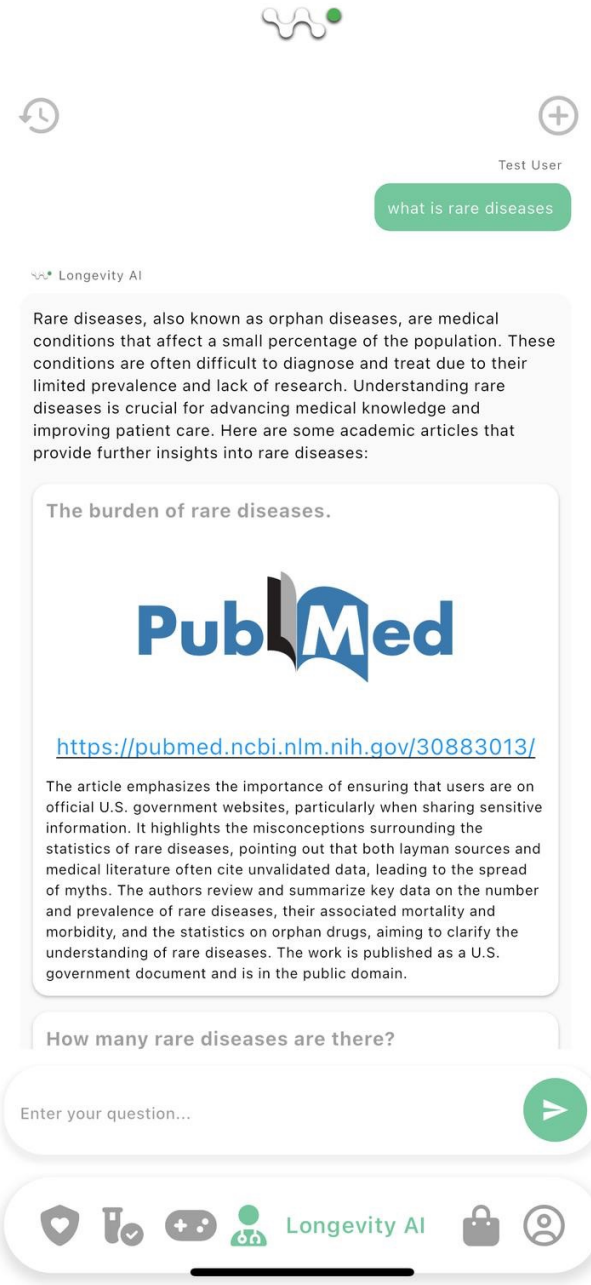

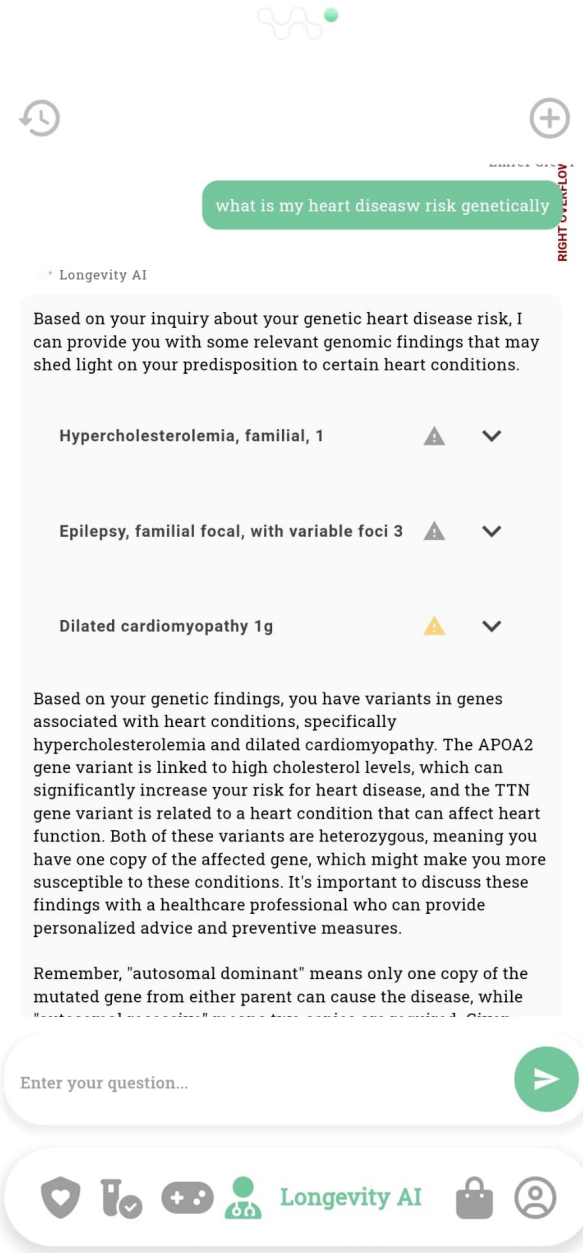

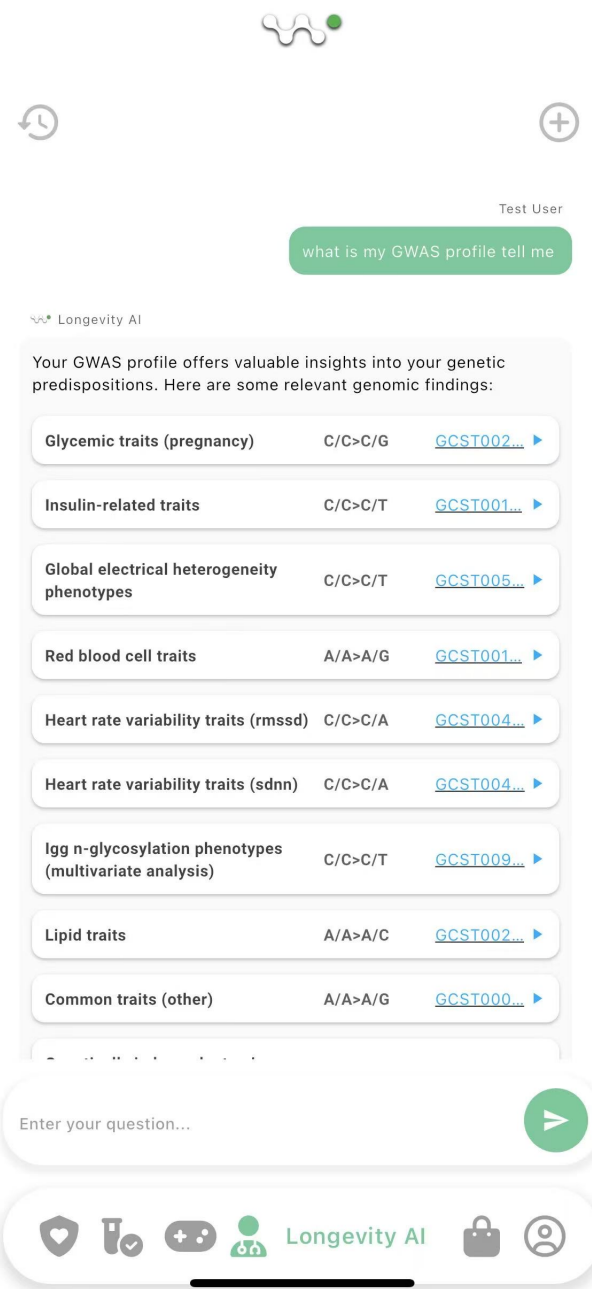

Supplementary Figure 4

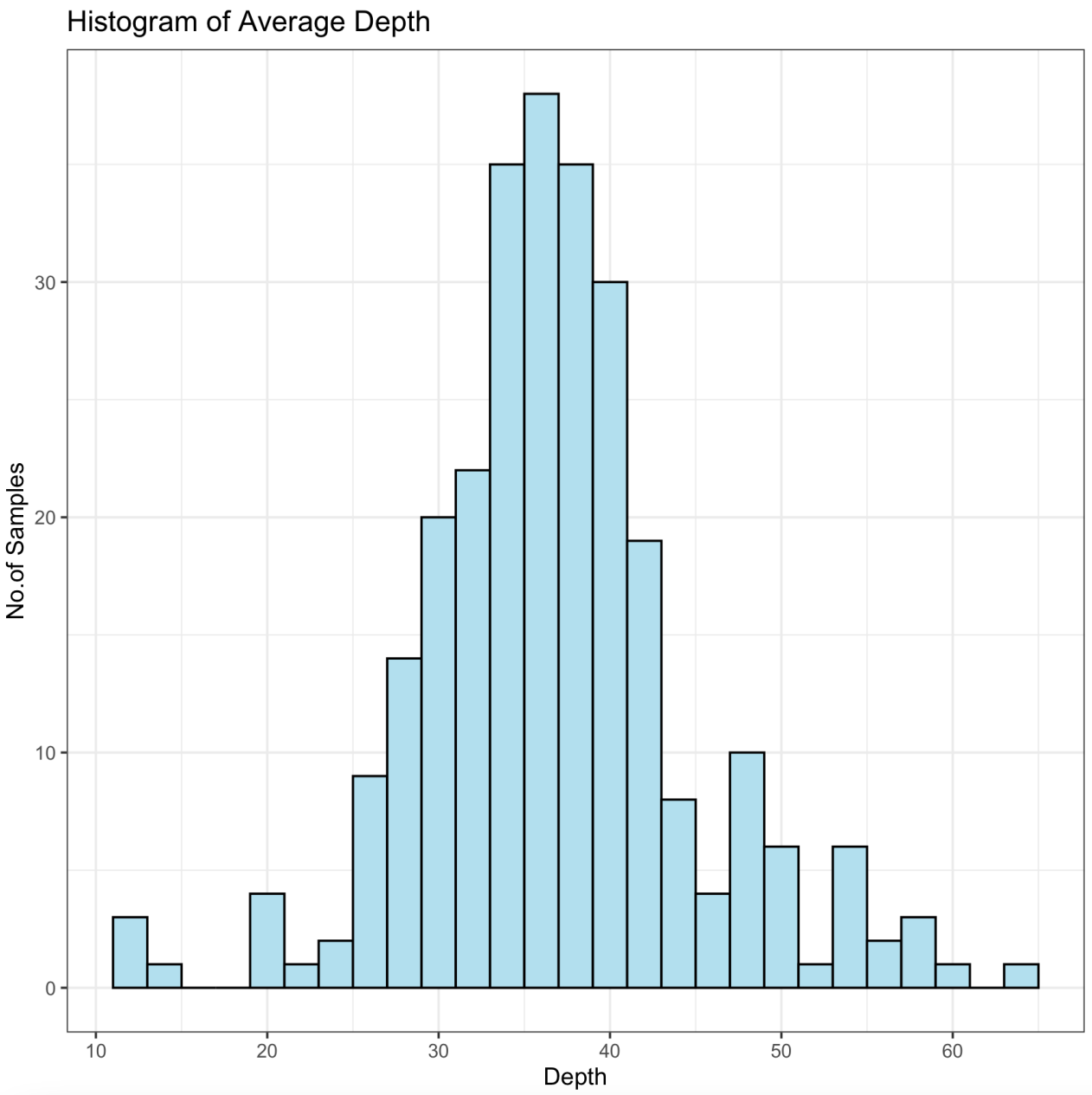

Supplementary Figure 5

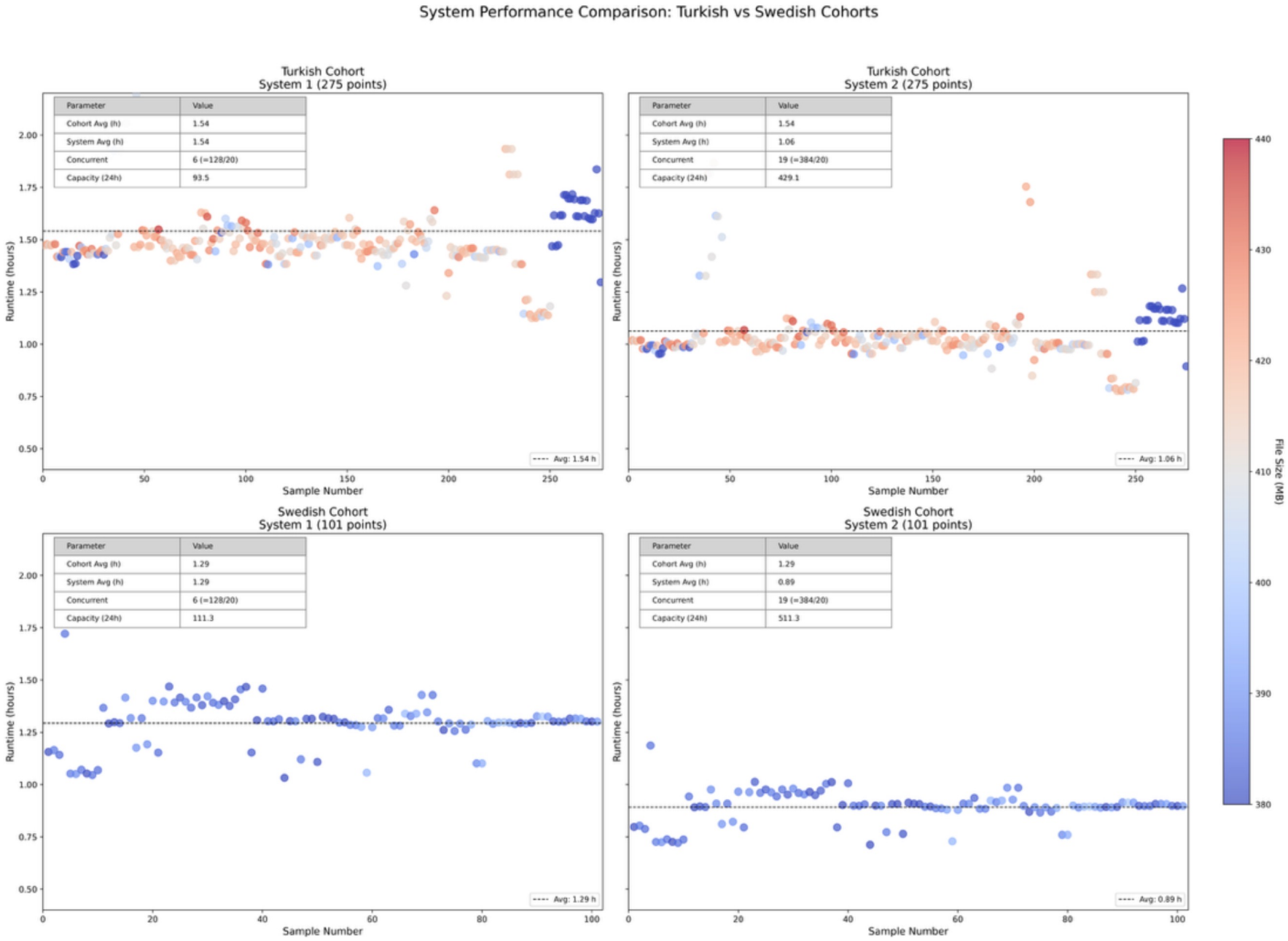

Supplementary Figure 6

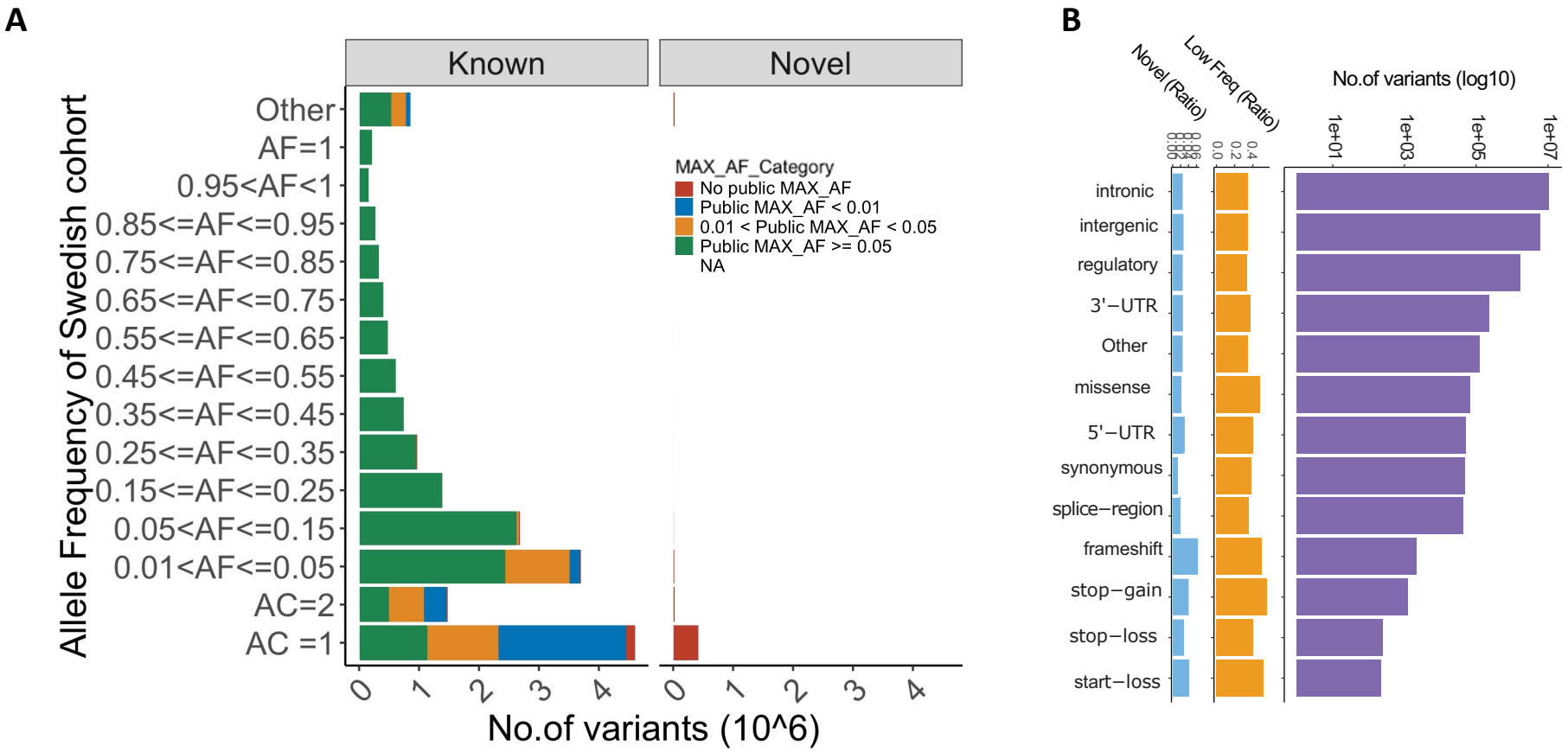

Supplementary Figure 7

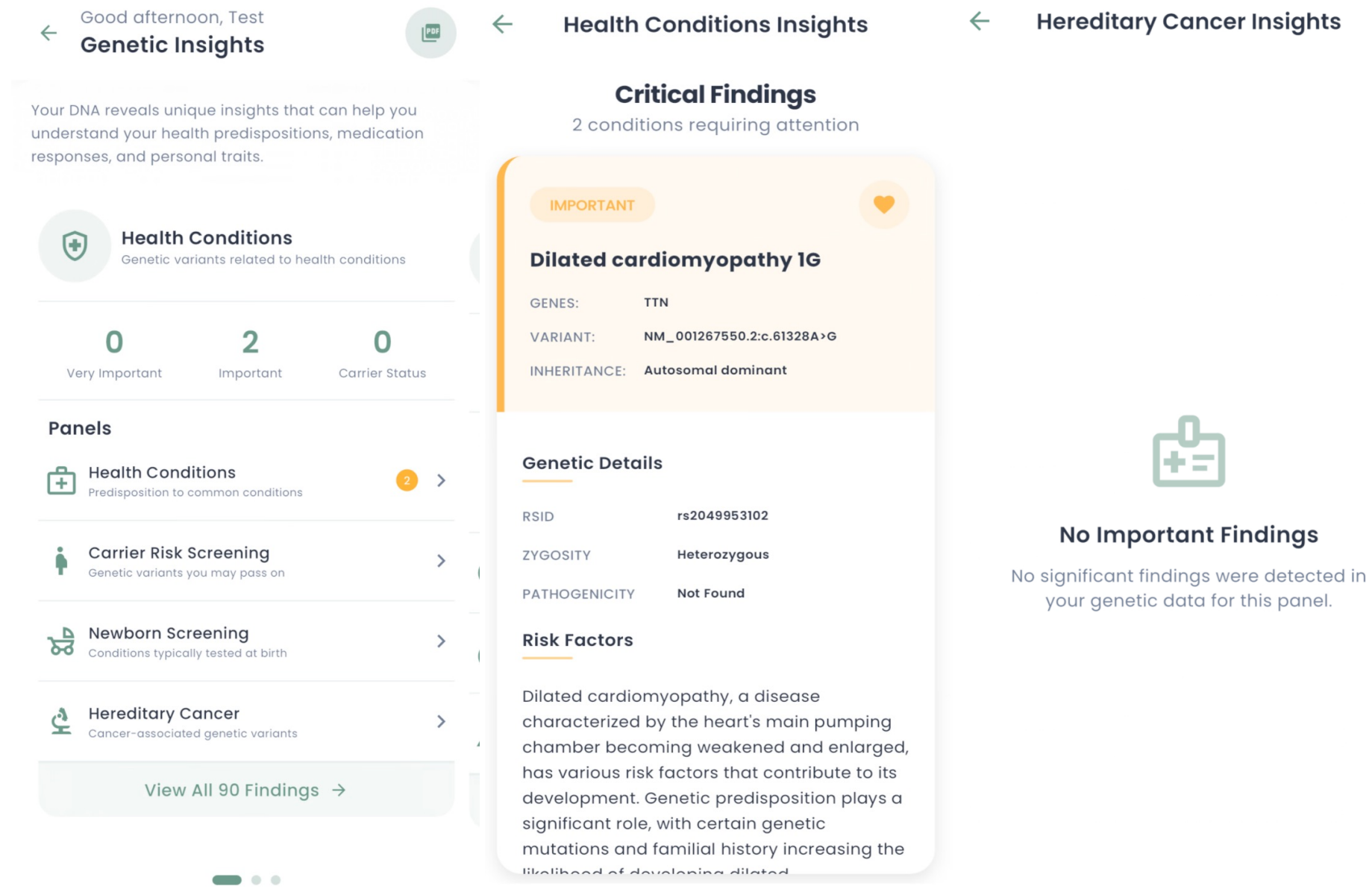

Supplementary Figure 8

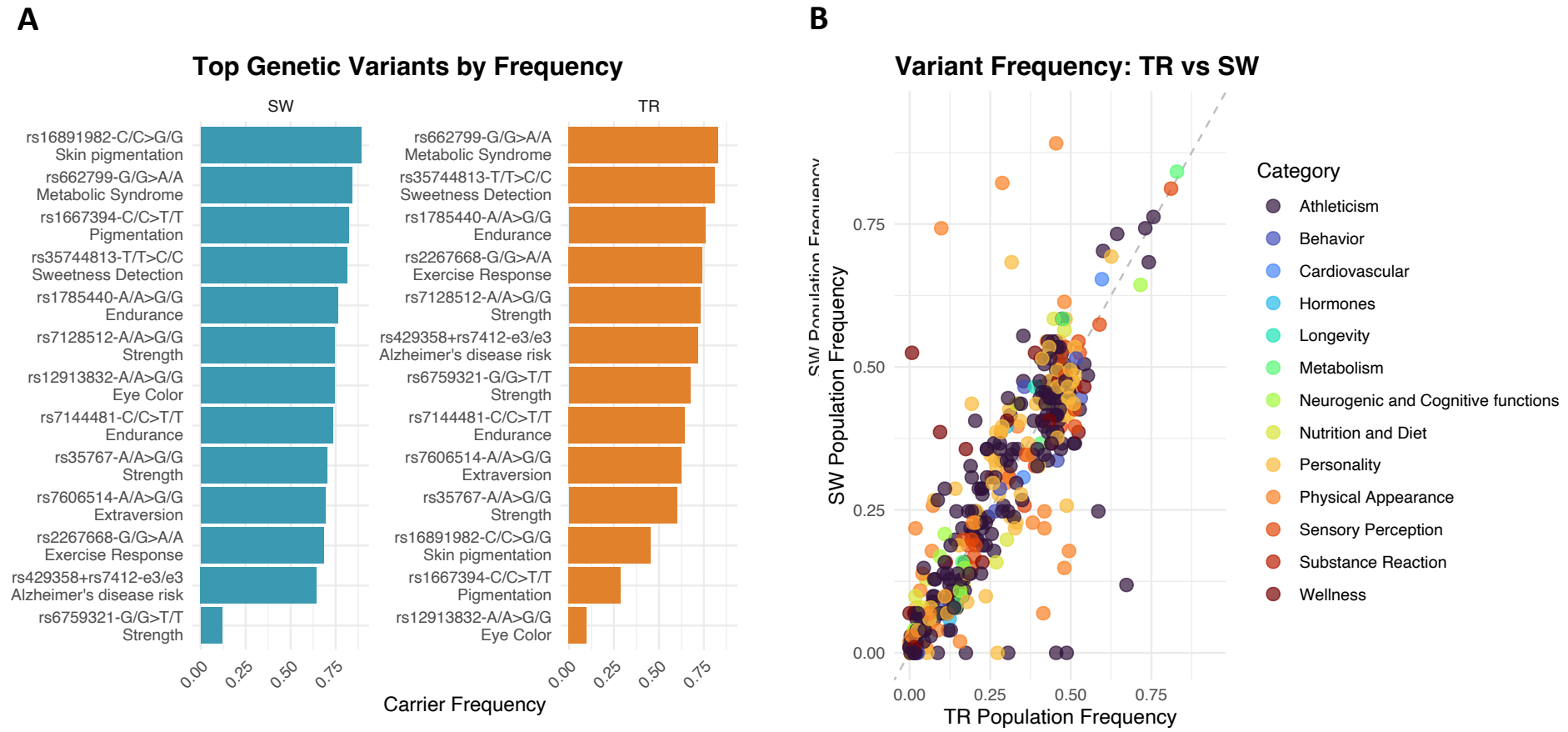

Supplementary Figure 9

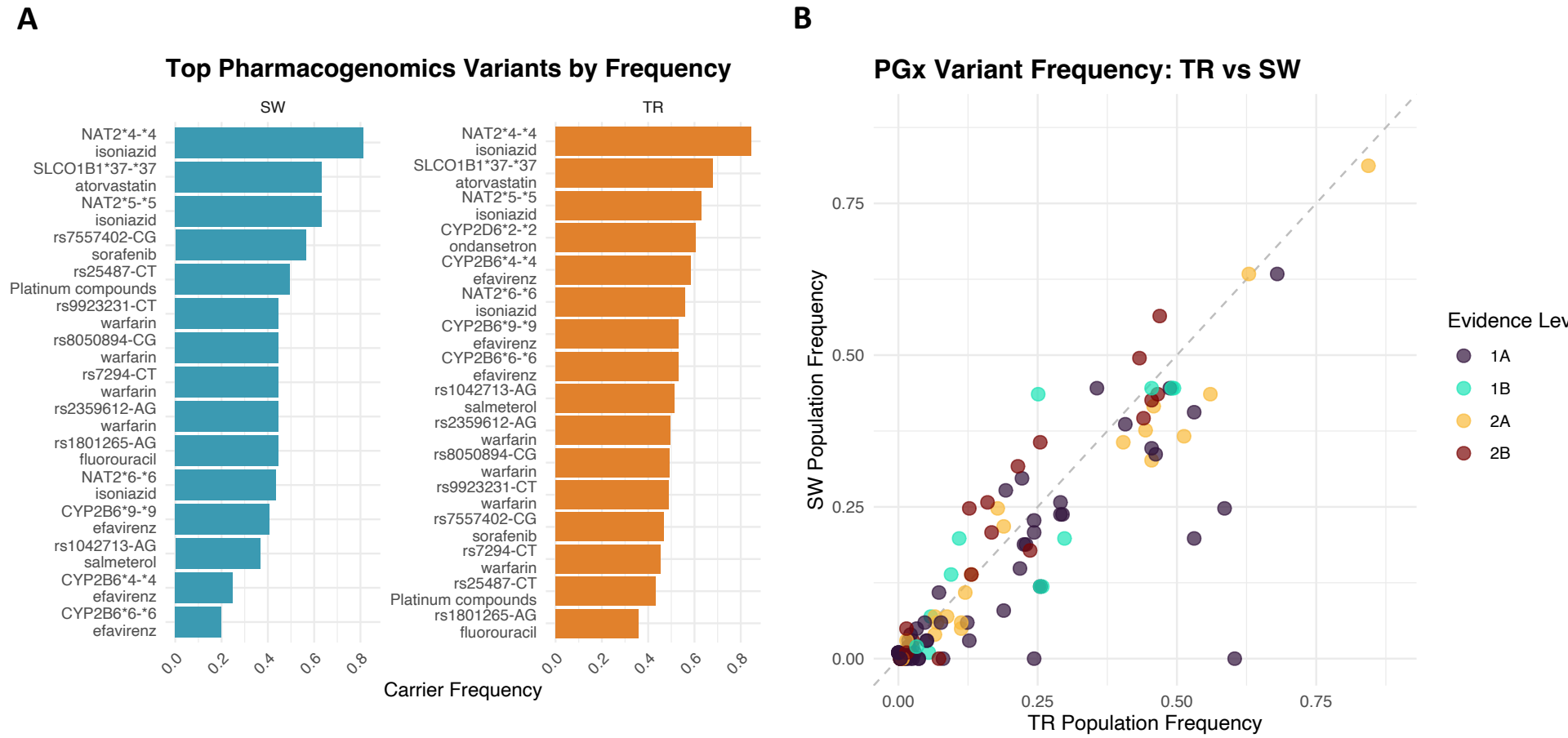

Supplementary Figure 10

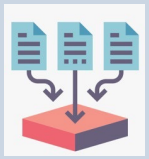

1. Data collection
-Web portal/App Interface (User-friendly)
-API Integration To Collect Data

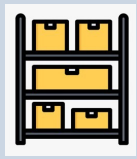

2. Data storage
-Database Management System (MySQL)
-Cloud Storage (AWS)

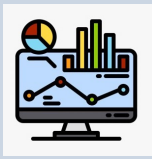

3. Data Analysis &Interpretation
-Analytics Pipeline and Local Server
-Expert Systems (Clinicians & Sequencers)

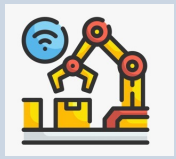

4. Data Visualization & Automated Reporting
- Visualization Tools & Custom Visual design
- Report generation
- Notification System

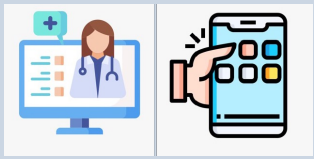

5. Web Portal/Mobile App
- Fronted Development
- User Authentication and Authorization
- Interactive Features (Query, Customize, Export Reports)

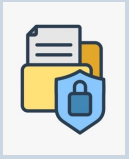

6. Security & Compliance
- Data Encryption (At rest and in transit)
- Compliance Standards

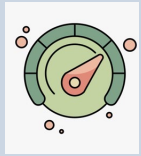

7. Scalability & Performance
- Load Balancing
- Scalable Architecture to accommodate increases in data volumn

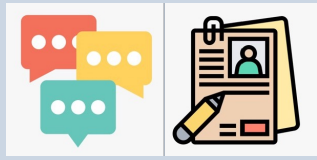

8. User Support & Documentation
- Communications with Expert Systems
- Documentation (User manual and PDF documents)
