# Supplementary material for: GenRiskPro: A Comprehensive Whole-Genome Sequencing Analysis Platform for Clinical and Wellness Applications
Source: Comput Struct Biotechnol J. 2026 Mar 6;35(2):0011. doi: 10.34133/csbj.0011 (PMC13394978; doi:10.34133/csbj.0011)
Supplement: Supplementary 1 — Figs. S1 to S10 Tables S1 to S4 Data S1 to S6 [file csbj.0011.f1.zip › Supplementary Material 1.pdf]

Supplementary Material 1

Test User account for accessing the GenRiskPro system

1. App Test User:

Email: *user@test.com*

Password: *test*

Link for Google: *https://play.google.com/store/apps/details?id=com.szalongevity.sza_mobile*

Link for Apple: *https://apps.apple.com/se/app/sza-longevity/id6468774325*

2. GenRiskPro platform Test User:

Email: *user@test.com*

Password: *test*

Link: *https://www.phenomeportal.org/dashboard*
