# Supplementary material for: GenRiskPro: A Comprehensive Whole-Genome Sequencing Analysis Platform for Clinical and Wellness Applications
Source: Comput Struct Biotechnol J. 2026 Mar 6;35(2):0011. doi: 10.34133/csbj.0011 (PMC13394978; doi:10.34133/csbj.0011)

Lifelong
Health Report

Name:	Report ID: WBWG_01_P001_262
DoB:	Patient ID: 01_P001_262
Gender:	Date: 6/7/2025

SZA Longevity
A healthy future awaits you

www.szalongevity.com
info@szalongevity.com
+90 216 570 20 00
İçerenköy Kerem Aydınlar Kampüsü, Kayışdağı Cd. No:32, 34752 Ataşehir/İstanbul

Dear patient,

Congratulations on reaching a pivotal moment in your personal health journey. The comprehensive insights in your unique report are designed to empower both you and your healthcare provider, paving the way for informed decisions about your well-being.

Your genome analysis is grounded in the latest advancements in medical genomics. It's an exciting field that continues to grow—expect ongoing enhancements to your insights as science advances. We're committed to updating you with new findings that can further illuminate your health picture.

For additional information and support resources, please visit our website. It's filled with useful content that can help you deepen your understanding of your health.

Should you have any inquiries or wish to discuss your results in detail, don't hesitate to reach out. Our team of expert genetic counselors is at your service. You can schedule an appointment via email at info@szalongevity.com or visit our contact page at www.szalongevity.com for local contact information.

We are honored to have played a part in unlocking the secrets of your genome. As your explorations into your genetics continue, remember that SZA Longevity is here to guide you every step of the way. Your feedback is invaluable to us—it helps enhance our services, ensuring that your journey with us is nothing short of exceptional.

Warm regards,

SZA Longevity Team

How Your Report is Organized

Understanding Your Report

- Information Overview
- How Your Results are Organized

Summary of Health Results

- Disease Risk Summary
- Carrier Status Summary
- Pharmacogenetics Summary

Detailed Health Results

- Disease Risk Details
- Carrier Status Details
- Pharmacogenetics Details

Genetic Susceptibilities that Influence Your Lifestyle

Glossary

Technical Note

Additional Resources

Information Overview

What is a Gene?

Genes are fundamental units of heredity, composed of DNA sequences that act as blueprints for building proteins—molecules that play critical roles in cellular function, structure, and regulation. Humans have approximately 20,000 genes, each responsible for guiding biological processes such as metabolism, immune response, and neurological development. Genes determine traits like eye color, hair texture, and even aspects of personality. Additionally, genes influence how we process nutrients, respond to environmental factors, and metabolize medications. Inheritance patterns shape genetic diversity, with each individual carrying two copies of most genes—one from each parent. Small changes in gene sequences, known as mutations or variants, can have significant effects, sometimes leading to inherited diseases, while other times contributing to beneficial adaptations such as resistance to certain infections. As scientific advancements continue, understanding our genetic blueprint helps us uncover the underlying mechanisms of health, disease, and evolution.

How Do Variants Occur?

Genetic variants are alterations in the DNA sequence that contribute to biological diversity and individuality. These variants can be inherited from parents or arise spontaneously due to environmental factors, cellular replication errors, or exposure to radiation and chemicals. While most genetic changes have no impact or even offer advantages—such as increased disease resistance—others may alter protein function, potentially leading to disorders or increased susceptibility to conditions like heart disease, cancer, or neurodegenerative disorders. There are several types of genetic variants, including single nucleotide polymorphisms (SNPs), insertions, deletions, and copy number variations (CNVs). Some variants influence physical characteristics, such as height and skin tone, while others affect internal processes, such as how the body metabolizes caffeine or responds to stress. Understanding genetic variations allows researchers and medical professionals to predict disease risk, develop targeted therapies, and personalize treatments based on an individual's unique genetic makeup.

Understanding Inheritance Patterns

Genetic inheritance follows distinct patterns that determine how traits and conditions are passed down through generations. Dominant inheritance occurs when a single altered copy of a gene is sufficient to express a trait or condition, as seen in disorders like Huntington's disease. In contrast, recessive inheritance requires two altered copies, one from each parent, for the trait to manifest, as is the case with cystic fibrosis or sickle cell anemia. Some traits follow sex-linked inheritance, where genes located on the X or Y chromosomes influence characteristics and disease susceptibility—such as hemophilia, which predominantly affects males due to the presence of only one X chromosome. Additionally, mitochondrial inheritance follows a maternal pattern, as mitochondria, the energy-producing structures in cells, contain their own DNA and are passed exclusively from mothers to offspring. Polygenic inheritance involves multiple genes working together to determine complex traits like intelligence, height, and disease susceptibility. Understanding inheritance patterns helps identify genetic risks, improve diagnostic accuracy, and guide reproductive decision-making.

How We Interpret Your Results

Genetic analysis involves sequencing DNA to identify variations and comparing findings with global scientific databases, clinical studies, and bioinformatics models. Using cutting-edge sequencing technologies like next-generation sequencing (NGS), we can detect genetic variants and assess their potential significance. Variants are classified into categories such as pathogenic (disease-causing), likely pathogenic, benign (harmless), and variants of uncertain significance (VUS). Our interpretation process considers multiple factors, including variant frequency in populations, inheritance patterns, and functional studies assessing gene expression and protein interactions. Additionally, pharmacogenetics—the study of how genes affect drug response—enables personalized treatment plans, ensuring that medications are prescribed based on an individual's genetic profile. This approach enhances precision medicine by tailoring healthcare strategies to prevent diseases, improve early diagnosis, and optimize therapeutic interventions. By continuously integrating the latest medical knowledge, we empower individuals to make informed health decisions based on their unique genetic landscape.

Understanding Your Report

How Your Data is Organized

There is a lot of information here. Take a look, and feel free to contact us at info@szalongevity.com if you have questions about your report. The first several summary pages of your report include all results that may have a medical impact on you and possibly other family members. Results are organized by their level of clinical relevance.

Immediate Attention Needed

These genetic findings require immediate medical attention. We have identified pathogenic or likely pathogenic variants in genes associated with serious health conditions that may significantly impact your well-being. For autosomal dominant conditions, having just one copy of these variants can be clinically significant, while for recessive conditions, the presence of two copies increases the likelihood of disease manifestation. These variants are well-documented in medical literature, including the American College of Medical Genetics and Genomics (ACMG) guidelines, which provide expert-reviewed classifications of genetic variants based on their clinical significance. The identified genetic changes may necessitate proactive medical management, further diagnostic testing, or consultations with healthcare professionals to assess potential implications and preventive measures.

What This Means For You:

- Consult with healthcare providers promptly
- May need specialist referrals
- Could require immediate preventive measures
- Important for family planning discussions
- May impact treatment decisions

Next Steps:

- Schedule an appointment with your healthcare provider
- Share this report with your medical team
- Consider genetic counseling for detailed guidance
- Discuss screening and prevention strategies
- Evaluate family testing options

Monitor Closely

These genetic findings, while important, may have a less immediate impact on your health. They include variants of uncertain significance in clinically important genes, or pathogenic variants with reduced penetrance. This means that while these genetic changes are significant, their exact impact on your health may vary.

What This Means For You:

- Regular medical monitoring recommended
- May influence preventive care decisions
- Could affect long-term health planning
- Relevant for family health history
- May need periodic reassessment

Next Steps:

- Discuss these findings during your next medical visit
- Develop a monitoring plan with your healthcare provider
- Stay informed about new research
- Consider lifestyle modifications if recommended
- Keep records for future medical reference

For Your Information

These genetic findings provide valuable information about your health but typically have lower immediate clinical impact. They may include variants associated with more common conditions or those with reduced penetrance. While not requiring urgent attention, this information can be valuable for your overall health management.

What This Means For You:

- Useful for general health awareness
- May influence lifestyle choices
- Could be relevant for future health decisions
- Part of your complete genetic profile
- May become more significant with new research

Next Steps:

- Keep this information for future reference
- Consider discussing during routine check-ups
- Be aware of related symptoms or conditions
- Update your family health history
- Stay informed about advances in genetic research

Summary

Name:
DoB:
Gender:
Report ID: WBWG_01_P001_262
Patient ID: 01_P001_262
Date: 6/7/2025

Summary

Health Results

This section summarizes genetic variants that may impact your health, categorized by importance. Very important variants require immediate medical attention, while important variants may need monitoring and potential lifestyle changes. Each finding includes recommendations for next steps and actions to manage health risks.

Immediate Attention Needed

Immediate medical attention is required. These findings indicate a high risk of developing severe or life-threatening conditions. The presence of pathogenic or likely pathogenic variants in crucial genes suggests a significantly increased predisposition to diseases such as hereditary cancers, cardiovascular disorders, or metabolic syndromes. According to medical literature, including guidelines from the American College of Medical Genetics (ACMG), individuals with these findings should consult a specialist, undergo confirmatory diagnostic testing if necessary, and discuss personalized risk mitigation strategies, including lifestyle modifications, preventive screenings, and potential medical interventions.

Neoplastic Syndrome and Cancer

Neoplastic syndromes and cancers have a notable genetic component, with mutations in specific genes increasing the risk of developing various types of cancer. Genetic risk assessments can identify individuals with hereditary cancer syndromes, enabling preventive measures such as enhanced surveillance, prophylactic surgeries, or chemoprevention. Understanding one's risk can guide better-informed lifestyle choices regarding their health, potentially leading to early detection and improved prognosis.

Breast-ovarian cancer, familial, susceptibility to, 1

Gene(s) & Variant(s)

- BRCA1
- NM_007294.4:c.2197_2201del
- Pathogenic
- Heterozygous
- Multi Factorial, Autosomal dominant

What It Means

You carry a variant increasing your risk for Breast-ovarian cancer, familial, susceptibility to, 1, a significant concern per ACMG. Proactive measures can reduce this risk. Further details are in the Detailed Disease Section.

Recommendations

- Discuss results with your physician(s). Medical intervention may be indicated.
- Genetic Counseling is strongly recommended.
- Share this information with at-risk relatives. They may benefit from genetic testing.
- Immediate Action Recommended.

Summary

Carrier Results

This section summarizes genetic variants that indicate carrier status for various conditions. Being a carrier typically does not affect your health but may be relevant for family planning decisions, understanding potential risks for future children, and informing relatives who may be planning families. Each finding includes recommendations for next steps and potential implications for family planning.

No Significant Carrier Status Variants Detected

Summary

Pharmacogenetics Analysis

Your genetic profile plays a crucial role in how your body processes and responds to medications. Pharmacogenetics is the study of how genetic differences influence individual responses to medications.

Immediate Attention Needed

(Major Gene-Drug Interaction)

These medications are classified under PharmGKB Level 1A, meaning they have the highest level of clinical evidence. Level 1A includes drug-gene interactions with specific dosing recommendations in FDA-approved labels or clinical guidelines. These interactions can significantly alter drug response, requiring immediate adjustments based on CPIC or FDA guidelines.

Fluorouracil

Gene	Genotype	Used for
DPYD	CT	Neoplasms

Both variants of rs1801159 are assigned normal function by CPIC. Patients with the CT genotype and cancer who are treated with fluorouracil, a fluoropyrimidine-based chemotherapy, may not have altered risk of drug toxicity as compared to patients with the CC or TT genotype. However, conflicting evidence has been reported. Other genetic and clinical factors may also influence risk of drug toxicity.

rs1801159 — **Toxicity**

Gene	Genotype	Used for
DPYD	CT	Neoplasms

Both variants of rs2297595 are assigned normal function by CPIC. Patients with the CT genotype and cancer who are treated with fluorouracil, a fluoropyrimidine-based chemotherapy, may not have altered risk of drug toxicity as compared to patients with the CC or TT genotype. However, conflicting evidence has been reported. Other genetic and clinical factors may also influence risk of drug toxicity.

rs2297595 — **Toxicity**

Gene	Genotype	Used for
DPYD	AG	Neoplasms

Both variants of rs1801265 are assigned normal function by CPIC. Patients with the AG genotype and cancer who are treated with fluorouracil, a fluoropyrimidine-based chemotherapy, may not have altered risk of drug toxicity as compared to patients with the AA or GG genotype. However, conflicting evidence has been reported. Other genetic and clinical factors may also influence risk of drug toxicity.

rs1801265 — **Toxicity**

Capecitabine

Gene	Genotype	Used for
DPYD	CT	Neoplasms

Both variants of rs1801159 are assigned normal function by CPIC. Patients with the CT genotype and cancer who are treated with capecitabine, a fluoropyrimidine-based chemotherapy, may not have altered risk of drug toxicity as compared to patients with the CC or TT genotype. However, conflicting evidence has been reported. Other genetic and clinical factors may also influence risk of drug toxicity.

rs1801159 — **Toxicity**

Gene	Genotype	Used for
DPYD	CT	Neoplasms

Both variants of rs2297595 are assigned normal function by CPIC. Patients with the CT genotype and cancer who are treated with capecitabine, a fluoropyrimidine-based chemotherapy, may not have altered risk of drug toxicity as compared to patients with the CC or TT genotype. However, conflicting evidence has been reported. Other genetic and clinical factors may also influence risk of drug toxicity.

rs2297595 — **Toxicity**

Gene	Genotype	Used for
DPYD	AG	Neoplasms

Both variants of rs1801265 are assigned normal function by CPIC. Patients with the AG genotype and cancer who are treated with capecitabine, a fluoropyrimidine-based chemotherapy, may not have altered risk of drug toxicity as compared to patients with the AA or GG genotype. However, conflicting evidence has been reported. Other genetic and clinical factors may also influence risk of drug toxicity.

rs1801265 — **Toxicity**

Warfarin

Gene	Genotype	Used for
VKORC1	CT	No info

Patients with the rs9923231 CT genotype may require a decreased dose of warfarin as compared to patients with the CC genotype or an increased dose as compared to patients with the TT genotype. Other genetic and clinical factors may also influence warfarin dose requirement.

rs9923231 — **Dosage**

Gene	Genotype	Used for
CYP4F2	TT	No info

Patients with the rs2108622 TT genotype may have increased warfarin dosage requirements as compared to patients with the CC or CT genotype. However, conflicting evidence has been reported. Other genetic and clinical factors may also affect warfarin dosage requirements.

rs2108622 — **Dosage**

Acenocoumarol

Gene	Genotype	Used for
VKORC1	CT	No info

Patients with the rs9923231 CT genotype may require a decreased dose of acenocoumarol as compared to patients with the CC genotype. However, conflicting evidence has been reported. Other genetic and clinical factors may also influence acenocoumarol dose requirements.

rs9923231 — **Dosage**

Phenprocoumon

Gene	Genotype	Used for
**VKORC1**	**CT**	**No info**

Patients with the rs9923231 CT genotype may require a lower dose when treated with phenprocoumon as compared to patients with the CC genotype. Other genetic and clinical factors may also influence phenprocoumon dose.

rs9923231 **Dosage**

Monitor Closely

(Significant Gene-Drug Interaction)

These interactions fall under PharmGKB Levels 1B, indicating limited or emerging evidence. Level 1B includes single-study reports or conflicting findings. While these findings are not currently actionable, they may become relevant as further research develops. Consider discussing with a healthcare provider for additional insights.

Warfarin

Gene	Genotype	Used for
**VKORC1**	**CT**	**over-anticoagulation**

Patients with the rs9923231 CT genotype may have increased risk of over-anticoagulation when treated with warfarin as compared with patients with genotype CC. However, conflicting evidence has been reported. Other genetic and clinical factors may also influence the toxicity to warfarin.

rs9923231 **Toxicity**

Gene	Genotype	Used for
**VKORC1**	**CT**	**No info**

Patients with the rs7294 CT genotype may require a higher dose of warfarin as compared to patients with the CC genotype. However, conflicting evidence has been reported. Other genetic and clinical factors may also affect warfarin dose requirements.

rs7294 **Dosage**

Gene	Genotype	Used for
**VKORC1**	**AG**	**No info**

Patients with the rs2359612 AG genotype may require a decreased dose of warfarin as compared to patients with the GG genotype. Other genetic and clinical factors may also influence dose of warfarin.

rs2359612 **Dosage**

Gene	Genotype	Used for
**VKORC1**	**CG**	**No info**

Patients with the rs8050894 CG genotype may require a lower dose of warfarin as compared to patients with the CC genotype. Other genetic and clinical factors may also influence warfarin dosage requirements.

rs8050894 **Dosage**

Gene	Genotype	Used for
**VKORC1**	**AG**	**No info**

Patients with the rs9934438 AG genotype may require a lower dose of warfarin as compared to patients with the GG genotype, and a higher dose as compared to patients with the AA genotype. However, conflicting evidence has been reported. Other clinical and genetic factors may also influence warfarin dose requirements.

rs9934438 **Dosage**

Disease Risk Detailed

Disease Risk Detailed

Health Results

This section summarizes genetic variants that may impact your health, categorized by importance. Very important variants require immediate medical attention, while important variants may need monitoring and potential lifestyle changes. Each finding includes recommendations for next steps and actions to manage health risks.

Immediate Attention Needed

Category	Disease	Variant
**Neoplastic Syndrome and Cancer**	Breast-ovarian cancer, familial, susceptibility to, 1	NM_007294.4:c.2197_2201del

Immediate Attention Needed

Neoplastic Syndrome and Cancer

Neoplastic syndromes and cancers have a notable genetic component, with mutations in specific genes increasing the risk of developing various types of cancer. Genetic risk assessments can identify individuals with hereditary cancer syndromes, enabling preventive measures such as enhanced surveillance, prophylactic surgeries, or chemoprevention. Understanding one's risk can guide better-informed lifestyle choices regarding their health, potentially leading to early detection and improved prognosis.

? Breast-ovarian cancer, familial, susceptibility to, 1

(Total reads: 32, Reference allele: 14, Alternative allele: 18)

Gene(s) & Variant(s)

Gene: BRCA1
Variant: NM_007294.4:c.2197_2201del
Zygosity: Heterozygous
Inheritance: Multi Factorial, Autosomal dominant
Pathogenicity: Pathogenic
rsID: rs80357507

What It Means

This is a very important genetic finding on your sequencing results, because this variant falls into one of the 78 most promising disease-causing genes suggested by American College of Medical Genetics and Genomics Secondary findings (SF), version3.1.(PMID: 35802134). This variant is presented in ClinVar, as identified as pathogenic/likely pathogenic; or that it could affect protein function. This disease is inherited in Your variant is a heterozygous variant, which means the variant is existed on only one copy of the paired chromosomes (you've inherited the different alleles of a gene from each of your parents).

Recommendations

- Discuss results with your physician(s). Medical intervention may be indicated.
- Genetic Counseling is strongly recommended.
- Share this information with at-risk relatives. They may benefit from genetic testing.
- Immediate Action Recommended.

Disease Epidemiology

Breast-ovarian cancer, when occurring in a familial context, represents a significant concern with regards to inherited cancer susceptibility. This cluster of cancer types, including both breast and ovarian malignancies, is often associated with specific genetic mutations, such as those in the BRCA1 and BRCA2 genes. The prevalence of this condition is estimated to be around 10-15% of all breast and ovarian cancer cases, with a higher incidence observed in certain populations. Familial breast-ovarian cancer exhibits variable expressivity and penetrance, contributing to its complexity. While it can manifest at any age, it often becomes apparent in middle-aged women. Both genders can be susceptible, although women are disproportionately affected, and its prevalence spans across diverse ethnic backgrounds.

Lifestyle and Prevention

Managing familial susceptibility to breast-ovarian cancer involves a proactive and holistic approach to prevention. Regular medical check-ups, including breast and ovarian screenings, are crucial for early detection and effective intervention. Adopting a healthy lifestyle, such as maintaining a balanced diet, engaging in regular physical activity, and avoiding tobacco and excessive alcohol consumption, can significantly reduce the risk of cancer development. Moreover, genetic counseling is essential for those with a family history of breast-ovarian cancer, as it can provide valuable insights into individual risk levels and guide personalized prevention strategies. This may include increased surveillance, chemoprevention, or risk-reducing surgeries. By staying informed and taking proactive steps, individuals can significantly enhance their well-being and effectively manage their risk of breast-ovarian cancer.

Risk Factors

Familial breast and ovarian cancer susceptibility is significantly influenced by several risk factors. Inherited genetic mutations, such as BRCA1 and BRCA2, play a crucial role in the predisposition to these cancers. Women carrying these genetic abnormalities are at a higher risk of developing breast and ovarian malignancies compared to the general population. Apart from genetics, reproductive factors also contribute to the risk. Early menarche, late menopause, and nulliparity have been associated with an increased likelihood of developing breast and ovarian cancers. Hormone replacement therapy (HRT) and oral contraceptives may also elevate the risk, particularly in long-term users. Lifestyle factors, such as alcohol consumption, obesity, and physical inactivity, have been linked to a higher incidence of breast and ovarian cancers. Furthermore, environmental factors, including exposure to certain chemicals and radiation, can increase susceptibility to these malignancies. Notably, a family history of breast or ovarian cancer can significantly influence an individual's risk, emphasizing the importance of cascade screening for at-risk relatives. These various risk factors highlight the complex interplay of genetic, reproductive, lifestyle, and environmental factors that contribute to the development of breast and ovarian cancers in families with a predisposition to these malignancies.

For Your Information

Neurological Disorders

Neurological disorders encompass a broad range of conditions affecting the brain, spinal cord, and nervous system. Genetic variations can influence susceptibility to various disorders, including Alzheimer's disease, epilepsy, and multiple sclerosis. Genetic testing provides valuable insights into one's risk, facilitating early intervention strategies, such as lifestyle modifications, medications, or therapies designed to slow disease progression, improve symptoms, and enhance quality of life.

? Neurodevelopmental disorder with coarse facies and mild distal skeletal abnormalities

(Total reads: 19, Reference allele: 14, Alternative allele: 5)

Gene(s) & Variant(s)

Gene: KDM6B
Variant:
NM_001348716.2:c.764_765insCCCC
Zygosity: Heterozygous
Inheritance: Autosomal dominant
Pathogenicity: Not Found in ClinVar

What It Means

This variant is presented in ClinVar, as identified as pathogenic/likely pathogenic; or that it could affect protein function. This disease is inherited in an autosomal dominant manner. Dominant means that a single copy of the mutated gene (from one parent) is enough to cause the disorder. Your variant is a heterozygous variant, which means the variant is existed on only one copy of the paired chromosomes (you've inherited the different alleles of a gene from each of your parents).

Recommendations

- Share this information with at-risk relatives. They may benefit from genetic testing.
- Consider genetic counseling.
- Discuss results with your physician at your next annual physical, as they may impact your health.
- Additional testing or confirmatory genetic testing may be recommended by your physician before taking any medical action.
- Lifestyle modifications may be helpful.

Disease Epidemiology

Neurodevelopmental disorder with coarse facies and mild distal skeletal abnormalities is a rare genetic condition, with its prevalence estimated to be around 1 in 100,000 individuals globally. This disorder exhibits significant variability in clinical presentation, even among family members, which adds to its complexity. The disorder typically becomes apparent in early childhood, and while both genders can be affected, it shows a slight male predominance. The condition affects individuals across various ethnic backgrounds, and its diagnosis is often based on a combination of clinical features, genetic testing, and imaging studies. The disorder is characterized by intellectual disability, coarse facial features, and mild distal skeletal abnormalities, and its management primarily involves a multidisciplinary approach, focusing on symptomatic treatment and supportive care.

Lifestyle and Prevention

Managing a neurodevelopmental disorder with coarse facies and mild distal skeletal abnormalities necessitates a multifaceted lifestyle approach for optimal prevention and symptom management. Crucial components include regular neurological and developmental assessments to track progress and address emerging concerns. Implementing targeted therapies, such as speech, occupational, and physical therapy, can significantly enhance functional abilities and quality of life. Adopting lifestyle modifications, like maintaining a regular sleep schedule, engaging in regular physical activity, and fostering a conducive learning environment, are essential for overall well-being. Genetic counseling remains indispensable in guiding personalized prevention strategies, family planning, and education for both the affected individual and their relatives. Early intervention and consistent adherence to the recommended management plan are vital for favorable long-term outcomes.

Risk Factors

Neurodevelopmental disorders with coarse facies and mild distal skeletal abnormalities encompass a range of conditions with varying risk factors. While the specific causes and contributors may differ, several factors have been identified as increasing the risk of developing these disorders. Genetic predisposition plays a significant role, with certain genetic mutations or variations contributing to the onset of these disorders. Environmental factors, such as maternal exposure to harmful substances during pregnancy, including alcohol, drugs, or toxic chemicals, can also increase the risk. Additionally, maternal health conditions, such as diabetes or infections, may contribute to the development of these neurodevelopmental disorders. Premature birth or low birth weight has also been linked to an increased risk. Furthermore, advanced parental age at conception may play a role in the development of these disorders. Lastly, nutritional deficiencies during critical periods of fetal development can also contribute to the onset of these conditions. These diverse risk factors highlight the complex interplay of genetic and environmental factors that can influence susceptibility to neurodevelopmental disorders with coarse facies and mild distal skeletal abnormalities.

Endocrine and Metabolic Disorders

Endocrine and metabolic disorders are characterized by disruptions in the body's hormonal systems and metabolic processes. These disorders can lead to serious health conditions such as diabetes, thyroid diseases, obesity, metabolic syndrome, and adrenal insufficiency. Hormonal imbalances can negatively affect energy production, growth and development, immune function, and overall body regulation. Genetic factors, environmental influences, dietary habits, and lifestyle choices play a crucial role in the development of these conditions. Early diagnosis and appropriate treatment can help manage symptoms and reduce the risk of complications.

Hypercholesterolemia, familial, 1

(Total reads: 31, Reference allele: 0, Alternative allele: 31)

Gene(s) & Variant(s)

Gene: APOA2
Variant: null
Zygosity: Homozygous
Inheritance: Autosomal recessive, Autosomal dominant
Pathogenicity: Pathogenic
rsID: rs5082

What It Means

This variant is presented in ClinVar, as identified as pathogenic/likely pathogenic; or that it could affect protein function. This disease is inherited in Your variant is a homozygous variant, which means the variant is existed on both copies of the paired chromosomes (you've inherited the same alleles of a gene from each of your parents).

Recommendations

- Share this information with at-risk relatives. They may benefit from genetic testing.
- Consider genetic counseling.
- Discuss results with your physician at your next annual physical, as they may impact your health.
- Additional testing or confirmatory genetic testing may be recommended by your physician before taking any medical action.
- Lifestyle modifications may be helpful.

Disease Epidemiology

Familial Hypercholesterolemia (FH) is a genetic disorder characterized by high levels of low-density lipoprotein (LDL), or "bad" cholesterol, in the blood. This condition is caused by mutations in genes responsible for processing and removing LDL from the bloodstream. The prevalence of FH is estimated to be around 1 in 200 to 500 individuals worldwide, although many cases go undiagnosed. FH exhibits an autosomal dominant pattern of inheritance, meaning that a child has a 50% chance of inheriting the mutated gene from an affected parent. However, variable expressivity and incomplete penetrance can also occur, contributing to the complexity of this disorder. FH can manifest at any age, but it often becomes apparent in early to mid-adulthood. Both genders are susceptible to FH, and it affects individuals across diverse ethnic backgrounds. If left untreated, FH can lead to premature cardiovascular disease, including coronary artery disease and stroke.

Lifestyle and Prevention

Managing familial hypercholesterolemia involves a proactive lifestyle approach for effective prevention. Regular cholesterol level monitoring, dietary modifications, and consistent medication adherence are crucial. A cholesterol-lowering diet, rich in fruits, vegetables, whole grains, and lean proteins, can significantly reduce LDL (bad) cholesterol levels. Regular exercise and maintaining a healthy weight are also essential lifestyle factors. Genetic counseling is vital for understanding the risk of transmission to offspring and developing personalized prevention strategies for individuals and their families. In addition, avoiding tobacco and limiting alcohol consumption can contribute to better long-term cardiovascular health.

Risk Factors

Familial Hypercholesterolemia (FH) is a genetic disorder characterized by significantly elevated cholesterol levels from birth, predisposing individuals to early onset of atherosclerosis and cardiovascular disease. The primary risk factor for FH is genetic inheritance, with mutations in the LDL receptor, APOB, or PCSK9 genes causing impaired clearance of low-density lipoprotein (LDL) from the bloodstream. This results in excessive LDL, or "bad cholesterol," accumulation in the body, initiating atherosclerosis from a young age. Compounding the risk, lifestyle factors such as unhealthy diet, physical inactivity, and obesity can exacerbate cholesterol levels and accelerate atherosclerosis progression. While FH is manageable through cholesterol-lowering medications, lifestyle modifications, and regular monitoring, the interplay of genetic predisposition and environmental factors necessitates vigilant care and early intervention to mitigate the risk of premature cardiovascular complications.

Syndromic Disease

This group encompasses disorders characterized by a collection of symptoms that occur together, indicating an underlying genetic syndrome.

DDX41-related hematologic malignancy predisposition syndrome

(Total reads: 24, Reference allele: 12, Alternative allele: 12)

Gene(s) & Variant(s)

Gene: DDX41
Variant: NM_016222.4:c.1266_1267del
Zygosity: Heterozygous
Inheritance: Autosomal dominant
Pathogenicity: Not Found in ClinVar

What It Means

This variant is presented in ClinVar, as identified as pathogenic/likely pathogenic; or that it could affect protein function. This disease is inherited in an autosomal dominant manner. Dominant means that a single copy of the mutated gene (from one parent) is enough to cause the disorder. Your variant is a heterozygous variant, which means the variant is existed on only one copy of the paired chromosomes (you've inherited the different alleles of a gene from each of your parents).

Recommendations

• Share this information with at-risk relatives. They may benefit from genetic testing.
• Consider genetic counseling.
• Discuss results with your physician at your next annual physical, as they may impact your health.
• Additional testing or confirmatory genetic testing may be recommended by your physician before taking any medical action.
• Lifestyle modifications may be helpful.

Disease Epidemiology

DDX41-related hematologic malignancy predisposition syndrome is a rare genetic disorder that predisposes affected individuals to an increased risk of developing various blood cancers, such as myelodysplastic syndromes (MDS), acute myeloid leukemia (AML), and other related hematologic malignancies. This condition is caused by mutations in the DDX41 gene, and its inheritance pattern is autosomal dominant, meaning that a person has a 50% chance of inheriting the mutated gene from an affected parent. However, de novo mutations can also occur. The prevalence of DDX41-related hematologic malignancy predisposition syndrome is estimated to be low, but its true prevalence is unknown due to underdiagnosis and underreporting. The syndrome can manifest at any age, but it typically becomes apparent in adulthood. Both genders can be affected, and the disorder appears to affect individuals of various ethnic backgrounds equally. Further studies are needed to better understand the epidemiology of this rare syndrome and to improve its diagnosis and management.

Lifestyle and Prevention

Effectively managing DDX41-related hematologic malignancy predisposition syndrome involves a proactive and holistic lifestyle approach for long-term prevention and well-being. Regular hematological assessments and adherence to prescribed medical surveillance are crucial for early detection and intervention. Implementing lifestyle modifications, such as maintaining a balanced diet, regular exercise, and avoiding harmful habits like smoking, can significantly contribute to overall health. Additionally, addressing psychological well-being through stress management techniques and support groups can help mitigate the impact of the syndrome. Genetic counseling plays a vital role in providing personalized risk assessment, prevention strategies, and family planning guidance for affected individuals and their families. Early intervention and consistent management can greatly improve prognosis and quality of life for those with DDX41-related hematologic malignancy predisposition syndrome.

Risk Factors

DDX41-related hematologic malignancy predisposition syndrome is a genetic condition that increases the risk of developing certain types of blood cancers. The syndrome is associated with mutations in the DDX41 gene, which plays a crucial role in DNA repair and maintenance. Several risk factors contribute to the development of DDX41-related hematologic malignancies. Inherited genetic predisposition is a significant factor, with family history of hematologic malignancies increasing the risk of developing the syndrome. Exposure to environmental toxins, such as benzene and other chemicals found in certain workplaces, may also contribute to the development of DDX41-related hematologic malignancies. Additionally, individuals with a history of autoimmune disorders, such as lupus or rheumatoid arthritis, may have an increased risk of developing DDX41-related hematologic malignancies due to chronic inflammation and immune system dysregulation. Advanced age is another risk factor, as the risk of hematologic malignancies generally increases with age. Finally, certain lifestyle factors, such as smoking and alcohol consumption, may also contribute to the development of DDX41-related hematologic malignancies. These diverse risk factors highlight the complex interplay influencing susceptibility to DDX41-related hematologic malignancies, and underscore the importance of early detection and intervention for individuals with a family history of hematologic malignancies or other risk factors.

Organ Health

Genetic factors significantly influence the health of various organs, including the liver, kidneys, and lungs. Genetic predispositions can affect the risk of organ-specific diseases such as polycystic kidney disease, liver cirrhosis, and cystic fibrosis. Genetic testing enables targeted actions to preserve organ health, including lifestyle adjustments, regular monitoring, and preventive treatments, aiming to prevent or delay the onset of disease and maintain organ function.

Occult macular dystrophy

(Total reads: 39, Reference allele: 23, Alternative allele: 16)

Gene(s) & Variant(s)

Gene: RP1L1
Variant: NM_178857.6:c.4026_4027insACAGAAGACGGGCTGCAAGAAGAGGGGGTGCAGTTAGAGGAAACTAAAACATAAGAAGGGCTGCAAGAAGAGGGGGTGCAGTTAGAGGGGACTAAA
Zygosity: Heterozygous
Inheritance: Autosomal dominant
Pathogenicity: Not Found in ClinVar

What It Means

This is a noteworthy genetic finding in your sequencing results, it is within one of the disease-causing genes in our database (except the ACMG SF v3.1 list). This variant is presented in ClinVar, as identified as pathogenic/likely pathogenic; or that it could affect protein function. This disease is inherited in an autosomal dominant manner. Dominant means that a single copy of the mutated gene (from one parent) is enough to cause the disorder. Your variant is a heterozygous variant, which means the variant is existed on only one copy of the paired chromosomes (you've inherited the different alleles of a gene from each of your parents).

Recommendations

- Share this information with at-risk relatives. They may benefit from genetic testing.
- Consider genetic counseling.
- Discuss results with your physician at your next annual physical, as they may impact your health.
- Additional testing or confirmatory genetic testing may be recommended by your physician before taking any medical action.
- Lifestyle modifications may be helpful.

Disease Epidemiology

Occult macular dystrophy (OMD) is a rare inherited retinal disorder, with a prevalence estimated to be around 1 in 50,000 individuals globally. This condition is characterized by its variable expressivity and incomplete penetrance, contributing to its complexity and challenges in diagnosis. OMD typically manifests in childhood or early adulthood, although some cases may remain undetected until later in life. Both genders are susceptible to OMD, and it affects individuals across diverse ethnic backgrounds. This condition primarily impacts the central vision, causing gradual and progressive vision loss, ultimately leading to legal blindness in some cases. Despite its challenges, ongoing research and advancements in diagnostic techniques continue to improve our understanding and management of this uncommon ophthalmological condition.

Lifestyle and Prevention

Managing occult macular dystrophy requires a proactive lifestyle approach for optimal prevention and symptom management. Regular ophthalmological examinations and monitoring of visual acuity are crucial for early detection and timely intervention. Adopting low-vision habits, such as optimizing lighting, using magnifying aids, and maintaining regular contrast, can significantly enhance visual function and quality of life. Additionally, nutritional adjustments, including a balanced diet rich in antioxidants and essential fatty acids, may contribute to slowing disease progression. Genetic counseling plays a vital role in providing personalized prevention strategies, addressing potential risks, and guiding family planning decisions for individuals and their relatives affected by occult macular dystrophy.

Risk Factors

Occult macular dystrophy is a group of inherited retinal disorders that can lead to progressive vision loss. While the exact causes of this condition are not yet fully understood, several risk factors have been identified. Genetic predisposition plays a significant role in the development of occult macular dystrophy, with certain genetic mutations being associated with an increased risk. Additionally, environmental factors, such as prolonged exposure to blue light from digital devices and spending long hours in sunlight, may contribute to the progression of the disease. Other risk factors include a family history of retinal diseases, age-related macular degeneration, and other genetic eye conditions. Furthermore, lifestyle factors such as smoking, poor nutrition, and a lack of antioxidants in the diet may also increase the risk of developing occult macular dystrophy. It is important to note that while some of these risk factors can be modified, others cannot, and further research is needed to fully understand the complex interplay of factors that contribute to the development and progression of this condition.

Organ Health

Tubulointerstitial kidney disease, autosomal dominant, 2

(Total reads: 47, Reference allele: 34, Alternative allele: 13)

Gene(s) & Variant(s)

Gene: MUC1
Variant: NM_001371720.2:c.1419-1C>G
Zygosity: Heterozygous
Inheritance: Autosomal dominant
Pathogenicity: Not Found in ClinVar
rsID: rs1553199150

What It Means

This is a noteworthy genetic finding in your sequencing results, it is within one of the disease-causing genes in our database (except the ACMG SF v3.1 list). This variant is presented in ClinVar, as identified as pathogenic/likely pathogenic; or that it could affect protein function. This disease is inherited in an autosomal dominant manner. Dominant means that a single copy of the mutated gene (from one parent) is enough to cause the disorder. Your variant is a heterozygous variant, which means the variant is existed on only one copy of the paired chromosomes (you've inherited the different alleles of a gene from each of your parents).

Recommendations

• Share this information with at-risk relatives. They may benefit from genetic testing.
• Consider genetic counseling.
• Discuss results with your physician at your next annual physical, as they may impact your health.
• Additional testing or confirmatory genetic testing may be recommended by your physician before taking any medical action.
• Lifestyle modifications may be helpful.

Disease Epidemiology

Tubulointerstitial kidney disease, autosomal dominant, type 2 (ADTKD-Type 2) is a rare inherited renal disorder with a prevalence estimated to be around 1-9 in 1,000,000 individuals worldwide. This condition is characterized by the progressive degeneration and fibrosis of the kidney's tubules and interstitium, leading to a decline in renal function over time. ADTKD-Type 2 exhibits autosomal dominant inheritance, meaning that affected individuals have a 50% chance of passing the disorder on to their offspring. The age of onset and severity of ADTKD-Type 2 can vary significantly, even among family members, contributing to its complexity. Both men and women are susceptible to this disorder, and it affects individuals across diverse ethnic backgrounds. The disease often manifests in adulthood, typically between the third and fifth decades of life, and is characterized by chronic kidney disease, proteinuria, and, in some cases, kidney failure.

Lifestyle and Prevention

Managing autosomal dominant tubulointerstitial kidney disease requires a proactive lifestyle approach for effective prevention and symptom management. Regular nephrology check-ups, monitoring of kidney function, and adherence to prescribed medications are crucial. Lifestyle modifications, such as maintaining a healthy fluid intake, following a balanced diet, and avoiding nephrotoxic substances, contribute significantly to long-term well-being. In addition, addressing and controlling any underlying conditions, like hypertension or diabetes, is essential for preventing disease progression. Genetic counseling plays a vital role in providing personalized prevention strategies for individuals and their families, helping them understand the genetic components and potential implications of the disease.

Risk Factors

Tubulointerstitial kidney disease, autosomal dominant, 2 is a genetic disorder that primarily affects the kidneys' functional units, the tubules and interstitium. Several risk factors contribute to the progression and severity of this condition. A key risk factor is a family history of the disease, as it is inherited in an autosomal dominant pattern. Additionally, exposure to certain medications, such as lithium, analgesics, and chemotherapy drugs, can increase the risk of developing tubulointerstitial kidney disease. Long-term dehydration and recurrent kidney infections may also contribute to the disease's onset and progression. Environmental factors, such as lead exposure, can exacerbate kidney damage and accelerate disease progression. Moreover, certain comorbidities, like hypertension and diabetes, can further strain the kidneys and increase the risk of tubulointerstitial kidney disease. Lastly, advanced age and male gender have been identified as risk factors, with the disease more prevalent in older males. Understanding these risk factors is crucial for early detection, intervention, and management of tubulointerstitial kidney disease, autosomal dominant, 2.

Organ Health

Corneal dystrophy, Fuchs endothelial, 8

(Total reads: 27, Reference allele: 14, Alternative allele: 13)

Gene(s) & Variant(s)

Gene: AGBL1
Variant: NM_001386094.1:c.3343C>T
Zygosity: Heterozygous
Inheritance: Autosomal dominant
Pathogenicity: Not Found in ClinVar
rsID: rs78205732

What It Means

This variant is presented in ClinVar, as identified as pathogenic/likely pathogenic; or that it could affect protein function. This disease is inherited in an autosomal dominant manner. Dominant means that a single copy of the mutated gene (from one parent) is enough to cause the disorder. Your variant is a heterozygous variant, which means the variant is existed on only one copy of the paired chromosomes (you've inherited the different alleles of a gene from each of your parents).

Recommendations

- Share this information with at-risk relatives. They may benefit from genetic testing.
- Consider genetic counseling.
- Discuss results with your physician at your next annual physical, as they may impact your health.
- Additional testing or confirmatory genetic testing may be recommended by your physician before taking any medical action.
- Lifestyle modifications may be helpful.

Disease Epidemiology

Corneal dystrophy, Fuchs endothelial type (FECD), is a genetic disorder affecting the cornea, the clear front part of the eye. With a prevalence estimated to be 3-4 per 10,000 individuals, FECD is characterized by the progressive loss of corneal endothelial cells, leading to decreased vision and eventual blindness if left untreated. This condition typically manifests in adulthood, with a higher incidence in women than men. FECD exhibits autosomal dominant inheritance, although sporadic cases also occur. The disorder affects individuals of all ethnic backgrounds, with certain populations, such as those of European descent, showing a higher prevalence. The expressivity and penetrance of FECD vary within families, contributing to its complexity and unpredictability.

Lifestyle and Prevention

Managing Corneal dystrophy, such as Fuchs endothelial dystrophy, requires a proactive lifestyle approach for effective prevention and symptom management. Regular ophthalmological assessments are crucial to monitor disease progression and inform appropriate interventions. A key aspect of prevention involves protecting the eyes from damage and strain, including measures such as wearing sunglasses, taking regular breaks during screen time, and staying hydrated. For Fuchs endothelial dystrophy, avoiding activities that exacerbate symptoms, such as swimming or using hot tubs, can help manage discomfort. In addition, maintaining a healthy lifestyle, including a balanced diet and regular exercise, can support overall eye health and slow the progression of the disease. Genetic counseling may also be beneficial in developing personalized prevention strategies for individuals and their families, given the hereditary nature of corneal dystrophies.

Risk Factors

Corneal dystrophy, Fuchs endothelial type, is a genetic eye condition characterized by the buildup of abnormal material and damage to the cornea's endothelium, leading to cloudy vision and other symptoms. Several risk factors contribute to the development and progression of this corneal disorder. Inherited genetics play a significant role, with the condition being passed down through families in an autosomal dominant pattern. Certain genetic mutations, particularly in the COL8A2 gene, have been associated with an increased risk of Fuchs endothelial corneal dystrophy. Environmental factors, such as exposure to UV radiation and certain chemicals, may exacerbate the condition and accelerate its progression. Additionally, age is a significant risk factor, with most cases diagnosed in middle-aged or older adults. Women are also at a higher risk, with the condition affecting them more frequently and severely than men. Other medical conditions, such as diabetes and other eye disorders, may also contribute to the development of Fuchs endothelial corneal dystrophy. These diverse risk factors highlight the complex interplay influencing susceptibility to this corneal disorder, and underscore the importance of early detection and intervention for effective management.

Organ Health

Congenital stationary night blindness autosomal dominant 1

(Total reads: 18, Reference allele: 11, Alternative allele: 7)

Gene(s) & Variant(s)

Gene: RHO
Variant: NM_000539.3:c.759G>T
Zygosity: Heterozygous
Inheritance: Autosomal dominant
Pathogenicity: Uncertain Significance
rsID: rs756658659

What It Means

This is a noteworthy genetic finding in your sequencing results, it is within one of the disease-causing genes in our database (except the ACMG SF v3.1 list). This variant is presented in ClinVar, as identified as pathogenic/likely pathogenic; or that it could affect protein function. This disease is inherited in an autosomal dominant manner. Dominant means that a single copy of the mutated gene (from one parent) is enough to cause the disorder. Your variant is a heterozygous variant, which means the variant is existed on only one copy of the paired chromosomes (you've inherited the different alleles of a gene from each of your parents).

Recommendations

• Share this information with at-risk relatives. They may benefit from genetic testing.
• Consider genetic counseling.
• Discuss results with your physician at your next annual physical, as they may impact your health.
• Additional testing or confirmatory genetic testing may be recommended by your physician before taking any medical action.
• Lifestyle modifications may be helpful.

Disease Epidemiology

Congenital stationary night blindness autosomal dominant 1 (CSNB1) is a genetic disorder that affects an individual's vision in low light conditions. With an estimated prevalence of 1 in 50,000 individuals globally, CSNB1 is a rare but significant condition that impacts visual perception. This disorder exhibits high penetrance but variable expressivity, contributing to its complexity and diversity in symptom presentation within families. Although CSNB1 can affect individuals of all ages, it is typically diagnosed in childhood or early adulthood. Both genders are equally susceptible, and the disorder spans across various ethnic backgrounds, with no known predilection for specific populations. CSNB1 is caused by mutations in the NYX gene, which plays a crucial role in the functioning of retinal cells responsible for vision in low light conditions.

Lifestyle and Prevention

Managing Congenital stationary night blindness autosomal dominant 1 requires a combination of medical and lifestyle approaches for optimal prevention and management. While the condition is primarily genetic and not directly influenced by lifestyle factors, implementing certain measures can enhance overall vision and quality of life. Regular ophthalmological examinations are crucial for monitoring the progression of the disease and adjusting management strategies as needed. While there is no cure for this condition, low vision aids and assistive devices can help maximize remaining vision. Adopting healthy habits, such as maintaining a balanced diet, staying physically active, and avoiding smoking, can contribute to overall well-being and may help reduce the risk of developing secondary complications. Genetic counseling is also essential for individuals and their families to understand the condition's impact and develop personalized prevention strategies. While lifestyle modifications may not directly impact the progression of Congenital stationary night blindness autosomal dominant 1, they can significantly improve the individual's quality of life and overall well-being.

Risk Factors

Congenital stationary night blindness, autosomal dominant 1, is a genetic disorder that affects an individual's ability to see in low light conditions. The disorder is characterized by the mutation of the NYX gene, which plays a crucial role in the functioning of rod cells in the eye. Several risk factors contribute to the manifestation and severity of this condition. The autosomal dominant inheritance pattern implies that a single copy of the mutated gene from either parent can lead to the disorder. A family history of the condition significantly increases the risk of an individual developing congenital stationary night blindness. Additionally, environmental factors, such as prolonged exposure to excessive light, can exacerbate the symptoms and contribute to the progression of the disorder. Certain medications, including specific antibiotics and diuretics, may also interact with the disorder and impact visual function. Furthermore, comorbidities such as cataracts, glaucoma, or other eye conditions can interfere with the overall visual performance of individuals with congenital stationary night blindness. These risk factors highlight the complex interplay of genetic and environmental factors that can influence the development and severity of congenital stationary night blindness, autosomal dominant 1.

Organ Health

Pigmentary retinal dystrophy

(Total reads: 18, Reference allele: 11, Alternative allele: 7)

Gene(s) & Variant(s)

Gene: RHO
Variant: NM_000539.3:c.759G>T
Zygosity: Heterozygous
Inheritance: Autosomal recessive, Autosomal dominant
Pathogenicity: Uncertain Significance
rsID: rs756658659

What It Means

This is a noteworthy genetic finding in your sequencing results, it is within one of the disease-causing genes in our database (except the ACMG SF v3.1 list). This variant is presented in ClinVar, as identified as pathogenic/likely pathogenic; or that it could affect protein function. This disease is inherited in Your variant is a heterozygous variant, which means the variant is existed on only one copy of the paired chromosomes (you've inherited the different alleles of a gene from each of your parents).

Recommendations

- Share this information with at-risk relatives. They may benefit from genetic testing.
- Consider genetic counseling.
- Discuss results with your physician at your next annual physical, as they may impact your health.
- Additional testing or confirmatory genetic testing may be recommended by your physician before taking any medical action.
- Lifestyle modifications may be helpful.

Disease Epidemiology

Pigmentary retinal dystrophy (PRD) is a group of inherited ocular disorders that affect the retina, a light-sensitive layer at the back of the eye. With a prevalence estimated to be around 1 in 10,000 to 1 in 25,000 individuals worldwide, PRD exhibits significant variability in its clinical presentation and progression. This condition can affect individuals of all ages, but it typically manifests in childhood or early adulthood. Both genders are susceptible, and its prevalence spans across diverse ethnic backgrounds. PRD is characterized by the progressive degeneration of photoreceptor cells and retinal pigment epithelium, leading to symptoms such as night blindness, decreased visual acuity, and progressive constriction of the visual field. The condition can range from mild to severe, and its progression can be highly variable even among family members, contributing to its complexity.

Lifestyle and Prevention

Managing pigmentary retinal dystrophy requires a proactive lifestyle approach for optimal prevention and symptom management. Regular ophthalmological examinations and consultations are crucial for monitoring disease progression and maintaining eye health. Adopting low vision aids and techniques can significantly enhance daily functioning and quality of life. Lifestyle adaptations, such as optimizing lighting conditions and incorporating assistive technology, play a significant role in long-term well-being. Genetic counseling and family screening are essential for early detection and personalized prevention strategies. Additionally, maintaining a balanced diet and engaging in regular physical activity can contribute to overall health and well-being for individuals with pigmentary retinal dystrophy.

Risk Factors

Pigmentary retinal dystrophy (PRD) is a group of genetic eye disorders characterized by progressive degeneration of the retina, leading to decreased vision and potential blindness. Several risk factors contribute to the development and progression of PRD. Inherited genetic mutations are the primary cause, with various genes associated with the condition. Specifically, mutations in the RDS, ROM1, PRPH2, and ABCA4 genes have been linked to PRD. While these genetic factors are unmodifiable, early detection and intervention can significantly improve the prognosis of PRD. Environmental factors, such as prolonged exposure to high-energy light, may exacerbate the condition. Additionally, comorbidities like diabetes and hypertension can increase the risk of PRD and worsen its progression. Age is another significant risk factor, as PRD typically manifests in early to mid-adulthood and progresses with age. Lastly, a family history of PRD or other retinal dystrophies increases the likelihood of developing the condition. Risk factors for PRD are complex and multifaceted, emphasizing the importance of early detection, genetic counseling, and appropriate management strategies.

Miscellaneous Diseases

The miscellaneous category encapsulates a diverse array of conditions with genetic underpinnings not easily classified under the conventional disease groups. This includes rare genetic disorders, malignancies, and conditions with complex genetic etiologies. Genetic testing in this area can uncover unique genetic profiles, guiding bespoke interventions, research opportunities, and support for affected individuals and their families. Tailored healthcare approaches can significantly improve outcomes for these often overlooked conditions.

Retinitis pigmentosa 4

(Total reads: 18, Reference allele: 11, Alternative allele: 7)

Gene(s) & Variant(s)

Gene: RHO
Variant: NM_000539.3:c.759G>T
Zygosity: Heterozygous
Inheritance: Autosomal recessive, Autosomal dominant
Pathogenicity: Uncertain Significance
rsID: rs756658659

What It Means

This is a noteworthy genetic finding in your sequencing results, it is within one of the disease-causing genes in our database (except the ACMG SF v3.1 list). This variant is presented in ClinVar, as identified as pathogenic/likely pathogenic; or that it could affect protein function. This disease is inherited in Your variant is a heterozygous variant, which means the variant is existed on only one copy of the paired chromosomes (you've inherited the different alleles of a gene from each of your parents).

Recommendations

• Share this information with at-risk relatives. They may benefit from genetic testing.
• Consider genetic counseling.
• Discuss results with your physician at your next annual physical, as they may impact your health.
• Additional testing or confirmatory genetic testing may be recommended by your physician before taking any medical action.
• Lifestyle modifications may be helpful.

Disease Epidemiology

Retinitis Pigmentosa 4 (RP4) is a form of inherited retinal degeneration, caused by mutations in the PRPH2 gene. Its epidemiology shares some similarities with hypertrophic cardiomyopathy, with variable expressivity and penetrance contributing to its complexity. The prevalence of RP4 is estimated to be around 1 in 3000 to 1 in 4000 individuals worldwide, affecting both genders equally and spanning across diverse ethnic backgrounds. The condition typically manifests in childhood or early adulthood, with symptoms progressing slowly over time. The symptoms include night blindness, decreased peripheral vision, and eventual loss of central vision, ultimately leading to blindness. The progression of RP4 can vary significantly among affected individuals, with some maintaining useful vision into late adulthood while others experience severe vision loss at a younger age.

Lifestyle and Prevention

Managing Retinitis Pigmentosa 4 requires a proactive lifestyle approach for optimal prevention and symptom management. Regular ophthalmological examinations and adherence to prescribed treatments are crucial for maintaining eye health. Implementing lifestyle modifications, such as maintaining a balanced diet rich in vitamin A and antioxidants, can contribute significantly to long-term well-being. Low vision rehabilitation and assistive devices can help maximize remaining vision. Genetic counseling plays a vital role in guiding individuals and their families in understanding the genetic implications and available prevention strategies. Emotional support and education about the condition are essential for promoting mental health and overall quality of life.

Risk Factors

Retinitis Pigmentosa 4 (RP4) is a genetic eye condition that leads to progressive vision loss. Several risk factors contribute to the development and progression of RP4. Inherited genetic mutations, particularly in the RPGR gene, are the primary contributors to RP4. These genetic variations can lead to the deterioration of photoreceptor cells in the retina, resulting in impaired vision. Environmental factors, such as exposure to UV radiation and smoking, may exacerbate the condition and accelerate vision loss. Additionally, certain medications, including some antibiotics and antimalarial drugs, have been associated with an increased risk of developing RP4. Comorbidities, such as diabetes and hypertension, can also contribute to the progression of RP4. Furthermore, individuals with a family history of RP4 are at a higher risk of developing the condition. Awareness of these risk factors and taking preventive measures, such as avoiding UV radiation, quitting smoking, and managing comorbidities, are crucial in mitigating the impact of RP4.

Hereditary Disease

This group includes diseases that are passed down through families due to genetic mutations, often following specific inheritance patterns.

Microcephaly, facial dysmorphism, renal agenesis, and ambiguous genitalia syndrome

(Total reads: 40, Reference allele: 0, Alternative allele: 40)

Gene(s) & Variant(s)

Gene: CTU2
Variant:
NM_001012759.3:c.1097+6_1097+11del
Zygosity: Homozygous
Inheritance: Autosomal recessive
Pathogenicity: Not Found in ClinVar

What It Means

This variant is presented in ClinVar, as identified as pathogenic/likely pathogenic; or that it could affect protein function. This disease is inherited in an autosomal recessive manner. An autosomal recessive disorder means two copies of an abnormal gene must be present in order for the disease or trait to develop. Your variant is a homozygous variant, which means the variant is existed on both copies of the paired chromosomes (you've inherited the same alleles of a gene from each of your parents).

Recommendations

• Share this information with at-risk relatives. They may benefit from genetic testing.
• Consider genetic counseling.
• Discuss results with your physician at your next annual physical, as they may impact your health.
• Additional testing or confirmatory genetic testing may be recommended by your physician before taking any medical action.
• Lifestyle modifications may be helpful.

Disease Epidemiology

Microcephaly, facial dysmorphism, renal agenesis, and ambiguous genitalia syndrome is a rare genetic disorder with a complex inheritance pattern, affecting multiple organ systems. The prevalence of this condition is estimated to be very low, but its true incidence is difficult to determine due to underdiagnosis and varying clinical presentations. This syndrome can affect individuals of any gender and ethnic background, with symptoms often present at birth or becoming apparent in early childhood. The severity of the disorder varies widely, even among family members, indicating variable expressivity and incomplete penetrance. Microcephaly, or abnormally small head size, is a hallmark feature, along with distinctive facial dysmorphisms. Renal agenesis, or the absence of one or both kidneys, and ambiguous genitalia are also common in this syndrome. The underlying genetic causes are heterogeneous, and the diagnosis is often made based on clinical examination, imaging studies, and genetic testing.

Lifestyle and Prevention

Managing a syndrome that includes microcephaly, facial dysmorphism, renal agenesis, and ambiguous genitalia requires a comprehensive and personalized approach to effectively prevent associated complications. Regular developmental assessments, monitoring of overall health, and adherence to prescribed medications are crucial. Lifestyle adjustments, such as implementing a healthy and balanced diet, regular exercise, and avoiding harmful habits, play a significant role in promoting long-term well-being for individuals with this syndrome. Genetic counseling is also essential for developing tailored prevention strategies and providing necessary support for the individual and their family. Early intervention therapies, such as physical, occupational, and speech therapy, can greatly improve outcomes and quality of life. By staying proactive and engaged in a comprehensive management plan, individuals with this syndrome can lead fulfilling and rewarding lives.

Risk Factors

Microcephaly, facial dysmorphism, renal agenesis, and ambiguous genitalia syndrome is a complex genetic condition with various contributing factors. Genetic mutations, often de novo, play a significant role in the expression of this syndrome. However, environmental factors and maternal health also contribute to the risk and severity of the syndrome. Maternal exposure to certain infectious agents, such as cytomegalovirus or rubella, during pregnancy can increase the risk of microcephaly and other anomalies. Poor maternal nutrition, particularly insufficient intake of essential nutrients like folic acid, may also play a role in the development of this syndrome. Advanced maternal age has been linked to a higher likelihood of chromosomal abnormalities, which can contribute to the occurrence of the syndrome. Furthermore, exposure to teratogens, including alcohol, drugs, and certain medications, can increase the risk of facial dysmorphism, renal agenesis, and ambiguous genitalia. Lastly, consanguinity between parents can also increase the risk of this syndrome due to the potential for recessive gene expression. Early identification and intervention can significantly improve outcomes for affected individuals, highlighting the importance of prenatal screening and monitoring for mothers and fetuses at risk.

Detailed Results

Pharmacogenetics Analysis

Your genetic profile plays a crucial role in how your body processes and responds to medications. Pharmacogenetics is the study of how genetic differences influence individual responses to medications.

Immediate Attention Needed

(Major Gene-Drug Interaction)

These medications are classified under PharmGKB Level 1A, meaning they have the highest level of clinical evidence. Level 1A includes drug-gene interactions with specific dosing recommendations in FDA-approved labels or clinical guidelines. These interactions can significantly alter drug response, requiring immediate adjustments based on CPIC or FDA guidelines.

Fluorouracil

Gene	Genotype	Used for
**DPYD**	**CT**	**Neoplasms**

Both variants of rs1801159 are assigned normal function by CPIC. Patients with the CT genotype and cancer who are treated with fluorouracil, a fluoropyrimidine-based chemotherapy, may not have altered risk of drug toxicity as compared to patients with the CC or TT genotype. However, conflicting evidence has been reported. Other genetic and clinical factors may also influence risk of drug toxicity.

rs1801159 **Toxicity**

Gene	Genotype	Used for
**DPYD**	**CT**	**Neoplasms**

Both variants of rs2297595 are assigned normal function by CPIC. Patients with the CT genotype and cancer who are treated with fluorouracil, a fluoropyrimidine-based chemotherapy, may not have altered risk of drug toxicity as compared to patients with the CC or TT genotype. However, conflicting evidence has been reported. Other genetic and clinical factors may also influence risk of drug toxicity.

rs2297595 **Toxicity**

Gene	Genotype	Used for
**DPYD**	**AG**	**Neoplasms**

Both variants of rs1801265 are assigned normal function by CPIC. Patients with the AG genotype and cancer who are treated with fluorouracil, a fluoropyrimidine-based chemotherapy, may not have altered risk of drug toxicity as compared to patients with the AA or GG genotype. However, conflicting evidence has been reported. Other genetic and clinical factors may also influence risk of drug toxicity.

rs1801265 **Toxicity**

Capecitabine

Gene	Genotype	Used for
**DPYD**	**CT**	**Neoplasms**

Both variants of rs1801159 are assigned normal function by CPIC. Patients with the CT genotype and cancer who are treated with capecitabine, a fluoropyrimidine-based chemotherapy, may not have altered risk of drug toxicity as compared to patients with the CC or TT genotype. However, conflicting evidence has been reported. Other genetic and clinical factors may also influence risk of drug toxicity.

rs1801159 **Toxicity**

Gene	Genotype	Used for
**DPYD**	**CT**	**Neoplasms**

Both variants of rs2297595 are assigned normal function by CPIC. Patients with the CT genotype and cancer who are treated with capecitabine, a fluoropyrimidine-based chemotherapy, may not have altered risk of drug toxicity as compared to patients with the CC or TT genotype. However, conflicting evidence has been reported. Other genetic and clinical factors may also influence risk of drug toxicity.

rs2297595 **Toxicity**

Gene	Genotype	Used for
**DPYD**	**AG**	**Neoplasms**

Both variants of rs1801265 are assigned normal function by CPIC. Patients with the AG genotype and cancer who are treated with capecitabine, a fluoropyrimidine-based chemotherapy, may not have altered risk of drug toxicity as compared to patients with the AA or GG genotype. However, conflicting evidence has been reported. Other genetic and clinical factors may also influence risk of drug toxicity.

rs1801265 **Toxicity**

Warfarin

Gene	Genotype	Used for
**VKORC1**	**CT**	**No info**

Patients with the rs9923231 CT genotype may require a decreased dose of warfarin as compared to patients with the CC genotype or an increased dose as compared to patients with the TT genotype. Other genetic and clinical factors may also influence warfarin dose requirement.

rs9923231 **Dosage**

Gene	Genotype	Used for
**CYP4F2**	**TT**	**No info**

Patients with the rs2108622 TT genotype may have increased warfarin dosage requirements as compared to patients with the CC or CT genotype. However, conflicting evidence has been reported. Other genetic and clinical factors may also affect warfarin dosage requirements.

rs2108622 **Dosage**

Acenocoumarol

Gene	Genotype	Used for
**VKORC1**	**CT**	**No info**

Patients with the rs9923231 CT genotype may require a decreased dose of acenocoumarol as compared to patients with the CC genotype. However, conflicting evidence has been reported. Other genetic and clinical factors may also influence acenocoumarol dose requirements.

rs9923231 **Dosage**

Phenprocoumon

Gene	Genotype	Used for
**VKORC1**	**CT**	**No info**

Patients with the rs9923231 CT genotype may require a lower dose when treated with phenprocoumon as compared to patients with the CC genotype. Other genetic and clinical factors may also influence phenprocoumon dose.

rs9923231 — **Dosage**

Monitor Closely (Significant Gene-Drug Interaction)

These interactions fall under PharmGKB Levels 1B, indicating limited or emerging evidence. Level 1B includes single-study reports or conflicting findings. While these findings are not currently actionable, they may become relevant as further research develops. Consider discussing with a healthcare provider for additional insights.

Warfarin

Gene	Genotype	Used for
**VKORC1**	**CT**	**over-anticoagulation**

Patients with the rs9923231 CT genotype may have increased risk of over-anticoagulation when treated with warfarin as compared with patients with genotype CC. However, conflicting evidence has been reported. Other genetic and clinical factors may also influence the toxicity to warfarin.

rs9923231 — **Toxicity**

Gene	Genotype	Used for
**VKORC1**	**CT**	**No info**

Patients with the rs7294 CT genotype may require a higher dose of warfarin as compared to patients with the CC genotype. However, conflicting evidence has been reported. Other genetic and clinical factors may also affect warfarin dose requirements.

rs7294 — **Dosage**

Gene	Genotype	Used for
**VKORC1**	**AG**	**No info**

Patients with the rs2359612 AG genotype may require a decreased dose of warfarin as compared to patients with the GG genotype. Other genetic and clinical factors may also influence dose of warfarin.

rs2359612 — **Dosage**

Gene	Genotype	Used for
**VKORC1**	**CG**	**No info**

Patients with the rs8050894 CG genotype may require a lower dose of warfarin as compared to patients with the CC genotype. Other genetic and clinical factors may also influence warfarin dosage requirements.

rs8050894 — **Dosage**

Gene	Genotype	Used for
**VKORC1**	**AG**	**No info**

Patients with the rs9934438 AG genotype may require a lower dose of warfarin as compared to patients with the GG genotype, and a higher dose as compared to patients with the AA genotype. However, conflicting evidence has been reported. Other clinical and genetic factors may also influence warfarin dose requirements.

rs9934438 — **Dosage**

For Your Information (Minor Gene-Drug Interaction)

These interactions fall under PharmGKB Levels 2A and 2B, indicating limited or emerging evidence. Level 2A includes single-study reports or conflicting findings, while Level 2B represents preliminary in vitro or computational data with uncertain clinical significance. While these findings are not currently actionable, they may become relevant as further research develops. Consider discussing with a healthcare provider for additional insights.

Warfarin

Gene	Genotype	Used for
**VKORC1**	**CT**	**time to therapeutic inr**

Patients with the rs9923231 CT genotype may require shorter time to therapeutic INR when treated with warfarin as compared with patients with genotype CC. However, conflicting evidence has been reported. Other genetic and clinical factors may also influence the response to warfarin.

rs9923231 — **Efficacy**

Gene	Genotype	Used for
**VKORC1**	**CT**	**time in therapeutic range**

Patients with the rs9923231 CT genotype may spent less time in INR therapeutic range (TTR) when treated with warfarin as compared with patients with genotype CC. However, conflicting evidence has been reported. Other genetic and clinical factors may also influence the response to warfarin.

rs9923231 — **Efficacy**

Gene	Genotype	Used for
**VKORC1**	**CT**	**Hemorrhage**

Patients with the rs9923231 CT genotype may have an increased risk of bleeding when treated with warfarin as compared to patients with the CC genotypes. However, conflicting evidence has been reported. Other clinical and genetic factors may also influence risk of warfarin-induced bleeding.

rs9923231 — **Toxicity**

Gene	Genotype	Used for
**VKORC1**	**AC**	**No info**

Patients with the rs2884737 AC genotype may require higher dose of warfarin as compared to patients with the CC genotype. Other clinical and genetic factors may also influence warfarin dosage requirements.

rs2884737 **Dosage**

Platinum compounds

Gene	Genotype	Used for
**XRCC1**	**CT**	**Neoplasms**

Patients with cancer and the rs25487 CT genotype may have decreased response when treated with platinum-based therapies as compared to patients with the CC genotype. However, conflicting evidence has been reported. Other genetic and clinical factors may also influence response to platinum-based regimens.

rs25487 **Efficacy**

Methotrexate

Gene	Genotype	Used for
**SLC19A1**	**CC**	**Arthritis, Rheumatoid**

Patients with the rs1051266 CC genotype and rheumatoid arthritis may have decreased response when treated with methotrexate as compared to patients with the TT genotype. However, conflicting evidence has been reported. Other genetic and clinical factors may also influence methotrexate response.

rs1051266 **Efficacy**

Gene	Genotype	Used for
**ATIC**	**CT**	**Arthritis, Rheumatoid**

Patients with the rs4673993 CT genotype and Rheumatoid Arthritis may have decreased response when treated with methotrexate as compared to patients with the CC genotype. Other genetic and clinical factors may also influence methotrexate response.

rs4673993 **Efficacy**

Gene	Genotype	Used for
**MTHFR**	**AG**	**Arthritis, Juvenile Rheumatoid;Arthritis, Psoriatic;Arthritis, Rheumatoid;Drug Toxicity**

Patients with the rs1801133 AG genotype and Arthritis who are treated with methotrexate may have an increased risk of toxicity as compared to patients with the GG genotype, or may have a decreased risk of adverse events as compared to patients with the AA genotype. However, conflicting evidence has been reported. Other genetic and clinical factors may also influence methotrexate toxicity. This drug-variant pair has been assigned a â no recommendationâ by DPWG, as it was determined to be not clinically actionable.

rs1801133 **Toxicity**

Gene	Genotype	Used for
**MTHFR**	**AG**	**Drug Toxicity;hematotoxicity;Leukopenia;Lymphoma;mucositis;Neoplasms;Neutropenia;Osteosarcoma;Precursor Cell Lymphoblastic Leukemia-Lymphoma;primary central nervous system lymphoma;Thrombocytopenia;Toxic liver disease**

Patients with the rs1801133 AG genotype and cancer who are treated with methotrexate may be at increased risk of toxicity as compared to patients with the GG genotype, and may be at decreased risk of toxicity compared to patients with the AA genotype. However, conflicting evidence has been reported. Other genetic and clinical factors may also influence risk of toxicity following methotrexate treatment. This drug-variant pair has been assigned a â no recommendationâ by DPWG, as it was determined to be not clinically actionable.

rs1801133 **Toxicity**

Salmeterol

Gene	Genotype	Used for
**ADRB2**	**AA**	**Asthma**

Patients with the rs1042713 AA genotype and asthma may have a decreased response to salmeterol as compared to patients with the GG genotype. However, conflicting evidence has been reported. Other genetic and clinical factors may also influence a response to salmeterol.

rs1042713 **Efficacy**

Nicotine

Gene	Genotype	Used for
**CHRNA5**	**AA**	**Tobacco Use Disorder**

Patients with rs16969968 AA genotype may have an increased risk for nicotine dependence when exposed to nicotine as compared to patients with the AG or GG genotypes. However, conflicting evidence has been reported. Some findings are based on haplotype studies with either rs680244 or rs680244, rs569207 rs578776, and rs1051730. Other genetic and clinical factors may influence risk of nicotine dependency.

rs16969968 **Toxicity**

Phenprocoumon

Gene	Genotype	Used for
**VKORC1**	**CT**	**Hemorrhage;over-anticoagulation;time above therapeutic range**

Patients with the CT genotype may have an increased risk of adverse events (bleeding, over-anticoagulation or increased time above therapeutic range) when treated with phenprocoumon as compared to patients with the CC genotype. Other clinical and genetic factors may also influence risk of adverse events to phenprocoumon.

rs9923231 — **Toxicity**

Gene	Genotype	Used for
**VKORC1**	**AG**	**No info**

Patients with the rs9934438 AG genotype may require a decreased dose of phenprocoumon as compared to patients with the GG genotype, but an increased dose as compared to patients with the AA genotype. Other genetic and clinical factors may also influence dose of phenprocoumon.

rs9934438 — **Dosage**

Sorafenib

Gene	Genotype	Used for
**EPAS1**	**CG**	**Drug Toxicity**

Patients with the rs7557402 CG genotype may have a decreased risk of dermatologic toxicity when treated with sorafenib as compared to patients with the GG genotype. Other genetic and clinical factors may also influence risk of dermatologic toxicity when treated with sorafenib.

rs7557402 — **Toxicity**

Acenocoumarol

Gene	Genotype	Used for
**VKORC1**	**AG**	**No info**

Patients with the rs9934438 AG genotype may require decreased dose of acenocoumarol as compared to patients with genotype GG. Other genetic and clinical factors may also influence the dose of acenocoumarol.

rs9934438 — **Dosage**

Gene	Genotype	Used for
**CYP4F2**	**TT**	**Atrial Fibrillation**

Patients with the rs2108622 TT genotype who are treated with acenocoumarol may require a higher dose as compared to patients with the CC genotype. However, conflicting evidence has been reported. Other genetic and clinical factors may also influence required acenocoumarol dose.

rs2108622 — **Dosage**

Carbamazepine

Gene	Genotype	Used for
**SCN1A**	**CT**	**Epilepsy**

Patients with the rs3812718 CT genotype who are treated with carbamazepine may require a higher dose as compared to patients with the CC genotype but a lower dose as compared to patients with the TT genotype. Other genetic and clinical factors may also influence dose of carbamazepine.

rs3812718 — **Dosage**

Genetic Susceptibilities that Influence Your Lifestyle

Your genetic profile contains valuable insights about how your genes influence various aspects of your life, from cognitive abilities to physical traits. Each category below represents a collection of genetic markers that may affect your predispositions and characteristics. Understanding these genetic influences can help you make more informed decisions about your lifestyle, diet, and wellness strategies.

- ## Neurogenic and Cognitive Functions

  Genetic factors play a crucial role in shaping brain development, synaptic plasticity, and neurotransmitter regulation, directly impacting cognitive functions like memory retention, learning abilities, and mental agility. Variants in genes related to neurogenesis can influence susceptibility to neurological disorders, cognitive resilience, and adaptability to learning environments, affecting both short-term memory and long-term cognitive health.

- ## Cardiovascular

  Cardiovascular health is significantly influenced by genetic variants that regulate blood pressure, cholesterol metabolism, and arterial integrity. These genetic factors can determine an individual's predisposition to conditions such as hypertension, atherosclerosis, and myocardial infarction. Moreover, genetic insights can guide personalized lifestyle changes to enhance heart health and reduce cardiovascular risks.

- ## Immune System

  The immune system's effectiveness is modulated by genetic variants that control cytokine production, pathogen recognition, and immune cell activation. These genetic differences can impact an individual's susceptibility to autoimmune diseases, infection resilience, and allergic reactions. Understanding these genetic factors can provide insights into personalized approaches to bolstering immune defense mechanisms.

- ## Athleticism

  Athletic performance is deeply intertwined with genetic factors that influence muscle fiber composition, aerobic capacity, and recovery rates. Genes affecting mitochondrial efficiency, oxygen utilization, and muscle hypertrophy can predict an individual's potential in endurance versus power-based sports. Genetic profiling can thus guide training regimens tailored to maximize athletic output.

- ## Metabolism

  Metabolic rate and nutrient processing are governed by genetic variants that influence insulin sensitivity, fat oxidation, and energy expenditure. These genetic factors can determine tendencies toward weight gain, energy levels, and responses to different dietary macronutrients. Personalized dietary strategies based on genetic information can optimize metabolic health and weight management.

- ## Behavior

  Behavioral tendencies, such as impulsivity, risk tolerance, and social interaction styles, are shaped by genetic factors affecting neurotransmitter pathways and receptor sensitivities. These genetic influences can affect decision-making patterns, stress responses, and even susceptibility to behavioral conditions. Understanding these factors allows for tailored behavioral interventions and stress management techniques.

- ## Nutrition and Diet

  Genetic variations can influence how nutrients are metabolized, impacting dietary preferences, nutrient deficiencies, and even food sensitivities. For instance, polymorphisms in genes related to lactose tolerance, gluten sensitivity, or omega-3 metabolism can determine the optimal diet for an individual. Nutrigenomics enables personalized nutrition plans for improved health outcomes.

- ## Sensory Perception

  The way we perceive sensory inputs such as taste, smell, and sound is influenced by genetic variants that affect receptor function and neural processing. These variations can alter taste preferences, odor sensitivities, and even auditory perception. Understanding these genetic traits can explain individual differences in sensory experiences and preferences.

- ## Substance Reaction

  Reactions to substances like caffeine, alcohol, and various medications are influenced by genetic variants affecting metabolic pathways. Enzyme activity levels, influenced by genetic polymorphisms, can determine the speed at which substances are metabolized, influencing tolerance levels and adverse reactions. Pharmacogenomics uses this knowledge to optimize drug efficacy and minimize side effects.

- ## Wellness

  Overall wellness is affected by genetic predispositions towards certain health conditions, stress resilience, and lifestyle adaptability. Genes influencing circadian rhythms, inflammation pathways, and detoxification processes can impact daily energy levels, mood stability, and overall health. Genetic insights provide a roadmap for lifestyle modifications that enhance well-being and longevity.

- ## Hormones

  Hormonal balance, crucial for physiological and psychological health, is regulated by genetic variants that influence hormone synthesis, receptor sensitivity, and feedback mechanisms. These genetic factors can determine susceptibility to conditions like thyroid disorders, metabolic syndrome, and reproductive health issues. Understanding these influences aids in personalized hormone management strategies.

Longevity

Longevity is shaped by genetic variants that regulate cellular repair mechanisms, oxidative stress response, and metabolic efficiency. Telomere length, mitochondrial function, and DNA repair capacity are key factors influenced by these genes, contributing to the aging process and lifespan. Genetic profiling for longevity markers provides insights into personalized anti-aging interventions.

Physical Appearance

Genetic factors significantly determine physical characteristics such as skin pigmentation, hair texture, and facial features. Variants in genes affecting melanin production, hair follicle shape, and facial bone structure contribute to the diversity in human appearance. Understanding these genetic influences can also provide insights into conditions like alopecia and skin disorders.

Personality

Personality traits, such as extroversion, conscientiousness, and emotional stability, have genetic underpinnings linked to neurotransmitter activity and brain structure. Genetic variations in serotonin and dopamine pathways can influence mood regulation, stress resilience, and social behavior. These insights help in understanding individual differences in personality and psychological well-being.

Neurogenic and Cognitive Functions

Genetic factors play a crucial role in shaping brain development, synaptic plasticity, and neurotransmitter regulation, directly impacting cognitive functions like memory retention, learning abilities, and mental agility. Variants in genes related to neurogenesis can influence susceptibility to neurological disorders, cognitive resilience, and adaptability to learning environments, affecting both short-term memory and long-term cognitive health.

Alzheimer's disease risk

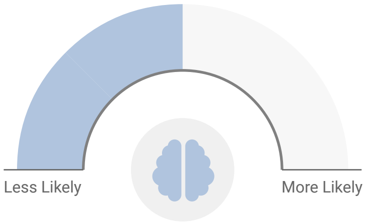

Having the C/C genotype in FN1 provides no conclusive evidence about your risk for Alzheimer's.

• **Gene:** FN1 • **rsID:** rs140926439 • **Your Genotype:** C/C • **Zygosity:** homo_ref

Description

In most cases, Alzheimer? does not have a single genetic cause. Instead, it can be influenced by multiple genes in combination with lifestyle and environmental factors. Consequently, a person may carry more than one genetic variant or group of variants that can either increase or reduce the risk of Alzheimer?.The apolipoprotein E (ApoE) gene makes a protein which, when combined with fat, becomes a lipoprotein. The lipoprotein ApoE is a very low-density lipoprotein, responsible in part for removing cholesterol from the bloodstream. Variations in ApoE affect cholesterol metabolism, which in turn alter your chances of having heart disease and in particular a heart attack or a stroke. Variations in ApoE are also associated with altered odds of having Alzheimer's disease and other diseases.There are three relatively common allelic variants of ApoE, as defined by two SNPs, rs429358 and rs7412 known as ApoE-_2, ApoE-_3, and ApoE-_4. The proteins produced by these genes are called ApoE2, ApoE3, and ApoE4. When rs429358 has the allele C and rs7412 has the allele T, the resulting isoform is _1.When rs429358 has the allele T and rs7412 has the allele T, the resulting isoform is _2.When rs429358 has the allele T and rs7412 has the allele C, the resulting isoform is _3.When rs429358 has the allele C and rs7412 has the allele C, the resulting isoform is _4.The most common variant overall is the "standard" ApoE-_3, and therefore more people inherited one ApoE-_3 from each parent than any other of the possible pairs of variants. Note that each of these types can actually have additional changes too, so there are different subtypes as well.APOE _2 may provide some protection against the disease. If Alzheimer? occurs in a person with this allele, it usually develops later in life than it would in someone with the APOE _4 gene. Roughly 5% to 10% of people have this allele.APOE _3, the most common allele, is believed to have a neutral effect on the disease ?neither decreasing nor increasing risk of Alzheimer?.APOE _4 increases risk for Alzheimer? and is associated with an earlier age of disease onset in certain populations. About 15% to 25% of people have this allele, and 2% to 5% carry two copies.

Extra Information

The variant rs140926439 located in FN1 was associated with strongly reduced risk of AD in APOE_4/4 carriers (OR_=_0.29; 95% CI [0.11, 0.78], P_=_0.014(PMID:38598053)An independent analysis in a large cohort of 7185 APOE_4 homozygous carriers found that rs140926439 variant in FN1 was protective of AD (OR_=_0.29; 95% CI [0.11, 0.78], P_=_0.014) and delayed age at onset of disease by 3.37 years (95% CI [0.42, 6.32], P_=_0.025). This is a relatively new finding in recent years.

Alzheimer's disease risk

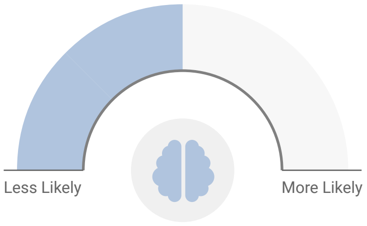

The ε3/ε3 genotype is the most common, found in about 60% of the population. This genotype is considered neutral, meaning it does not significantly increase or decrease the risk of developing Alzheimer's disease.

• **Gene:** APOE • **rsID:** rs429358+rs7412 • **Your Genotype:** e3/e3 • **Zygosity:**

Description

In most cases, Alzheimer? does not have a single genetic cause. Instead, it can be influenced by multiple genes in combination with lifestyle and environmental factors. Consequently, a person may carry more than one genetic variant or group of variants that can either increase or reduce the risk of Alzheimer?.The apolipoprotein E (ApoE) gene makes a protein which, when combined with fat, becomes a lipoprotein. The lipoprotein ApoE is a very low-density lipoprotein, responsible in part for removing cholesterol from the bloodstream. Variations in ApoE affect cholesterol metabolism, which in turn alter your chances of having heart disease and in particular a heart attack or a stroke. Variations in ApoE are also associated with altered odds of having Alzheimer's disease and other diseases.There are three relatively common allelic variants of ApoE, as defined by two SNPs, rs429358 and rs7412 known as ApoE-_2, ApoE-_3, and ApoE-_4. The proteins produced by these genes are called ApoE2, ApoE3, and ApoE4. When rs429358 has the allele C and rs7412 has the allele T, the resulting isoform is _1.When rs429358 has the allele T and rs7412 has the allele T, the resulting isoform is _2.When rs429358 has the allele T and rs7412 has the allele C, the resulting isoform is _3.When rs429358 has the allele C and rs7412 has the allele C, the resulting isoform is _4.The most common variant overall is the "standard" ApoE-_3, and therefore more people inherited one ApoE-_3 from each parent than any other of the possible pairs of variants. Note that each of these types can actually have additional changes too, so there are different subtypes as well.APOE _2 may provide some protection against the disease. If Alzheimer? occurs in a person with this allele, it usually develops later in life than it would in someone with the APOE _4 gene. Roughly 5% to 10% of people have this allele.APOE _3, the most common allele, is believed to have a neutral effect on the disease ?neither decreasing nor increasing risk of Alzheimer?.APOE _4 increases risk for Alzheimer? and is associated with an earlier age of disease onset in certain populations. About 15% to 25% of people have this allele, and 2% to 5% carry two copies.

Extra Information

This SNP, located in the fourth exon of the ApoE gene, affects the amino acid at position 130 of the resulting protein. The more common rs429358 allele is (T). If the allele is (C) and the same chromosome also harbors the rs7412(C) allele, the combination is known as an APOE-_4 allele. The APOE-_4 allele has a strong influence on the risk of Alzheimer's disease.CC genotype represents one of 2 snps relevant to classifying APOE genotype;CT genotype represents >3x increased risk for Alzheimer's; 1.4x increased risk for heart disease while TT represents common risk.

Parkinson's disease risk

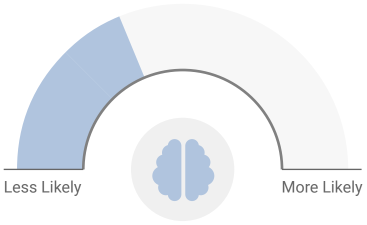

The G/G genotype suggests a more typical risk for Parkinson's compared to the other variants.

• **Gene:** LRRK2 • **rsID:** rs34637584 • **Your Genotype:** G/G • **Zygosity:** homo_ref

Description

Parkinson's disease is a neurodegenerative disorder that affects movement and can lead to tremors, stiffness, and difficulty with balance and coordination. While the exact cause of Parkinson's is not fully understood, research has identified several genetic variants that can increase the risk of developing the disease.

Extra Information

First discovered in 2004, rs34637584 is a SNP indicating a position within the LRRK2 that encodes a variant protein. This SNP is commonly referred to as the G2019S variant (or, mutation) based on the potential change from glycine (encoded by rs34637584(G) allele) to serine (encoded by the rs34637584(A) allele) at position 2019 of the LRRK2 protein.[PMID 15680456][PMID 15680455]One copy of a rs34637584(A) allele is sufficient to greatly increase one's risk for Parkinson's disease(refer to GA and AA genotype). It is considered a disease causing mutation because it is rarely found in healthy, elderly people without Parkinson's disease, and it has been found in both familial and sporadic types of the disease. While there are many different SNPs that can influence one's risk for Parkinson's disease, rs34637584 is an especially common cause of the disease in Berber Arabs and Ashkenazi Jews. Overall, the risk of Parkinson's disease for a person who inherits a rs34637584(A) allele is 28% at age 59, 51% at 69, and 74% at 79, according to the International LRRK2 Consortium.[PMID 18539534]The GG genotype represents a normal risk of developing Parkinson's diseases.

Parkinson's disease risk

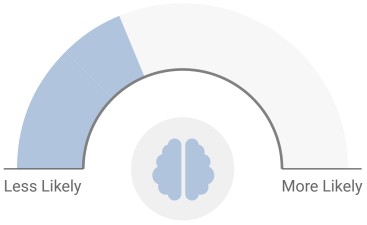

For Parkinson's disease risk, having the T/T genotype is generally seen as typical, with no increased risk.

• **Gene:** GBA • **rsID:** rs76763715 • **Your Genotype:** T/T • **Zygosity:** homo_ref

Description

Parkinson's disease is a neurodegenerative disorder that affects movement and can lead to tremors, stiffness, and difficulty with balance and coordination. While the exact cause of Parkinson's is not fully understood, research has identified several genetic variants that can increase the risk of developing the disease.

Extra Information

The N370S mutation in the GBA gene is linked to Gaucher disease Type 1, but it also increases the risk of developing Parkinson's disease. This connection highlights the broader impact of GBA gene mutations beyond Gaucher disease, particularly in relation to neurodegenerative diseases. The relationship is believed to be due to the role of glucocerebrosidase in cellular pathways that, when disrupted, contribute to the development of Parkinson's disease.C;C): High risk (6) for Gaucher's disease, type 1.(C;T): Carrier (3) of Gaucher disease allele; potential increased risk for Parkinson's.For the N370S variant (rs76763715) in the GBA gene: (C;C) High risk (6) for Gaucher's disease type 1;(C;T) Carrier (3) of Gaucher disease allele potential increased risk for Parkinson's (T;T) Common (0) variant with no associated risk.

Cardiovascular

Cardiovascular health is significantly influenced by genetic variants that regulate blood pressure, cholesterol metabolism, and arterial integrity. These genetic factors can determine an individual's predisposition to conditions such as hypertension, atherosclerosis, and myocardial infarction. Moreover, genetic insights can guide personalized lifestyle changes to enhance heart health and reduce cardiovascular risks.

Baseline Cholesterol

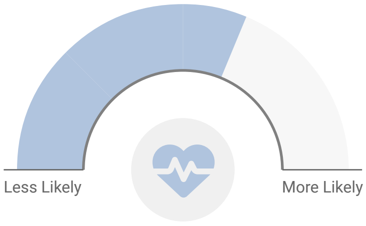

Your CT genotype in the PPARD gene suggests a possibility of having higher baseline cholesterol levels.

• **Gene:** PPARD • **rsID:** rs2016520 • **Your Genotype:** C/C>C/T • **Zygosity:** Heterozygous

Description

Cholesterol, a lipid (fat) molecule, is present in the cell membranes and plays a crucial role in synthesizing certain molecules like hormones and maintaining cell membrane structure. Adequate cholesterol levels are necessary for these physiological functions. Apart from obtaining cholesterol from dietary sources, our bodies can also synthesize it. Two main types of cholesterol, high-density lipoprotein (HDL) and low-density lipoprotein (LDL), exist. Both environmental factors and genetic influences contribute to our baseline cholesterol levels.

Extra Information

The rs2016520 CT/TT genotype has been linked to elevated cholesterol levels, as reported in a study (PMID: 18288282), compared to CC genotypes.The C or minor allele has been associated with higher production of PPAR delta gene products and lower risk of obesity(PMID: 22968028).

Blood Pressure

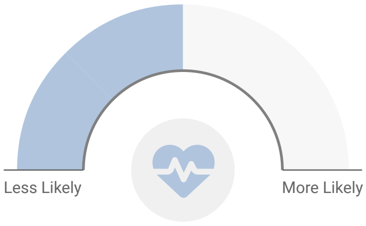

No significant effects on your blood pressure are noted with an A/A genotype.

• **Gene:** AGT • **rsID:** rs699 • **Your Genotype:** A/A • **Zygosity:** homo_ref

Description

Blood pressure is the force exerted by circulating blood on the walls of blood vessels. This pressure fluctuates based on factors such as one's situation, mental state, physical and mental activity, as well as health conditions. The nervous and endocrine systems play crucial roles in regulating blood pressure.

Extra Information

Research involving individuals of both European and Asian ancestry indicates that the rs699 CC genotype is correlated with an elevated likelihood of high blood pressure (hypertension) (PMID: 12805070)*.rs699 is a SNP in the angiotensin AGT gene that encodes a functional change. In most published literature, the name for this SNP is M235T, or perhaps Met235Thr, however its amino acid 268 (not 235) that varies based on the numbering in todays databases. rs699 is also occasionally known as C4072T.In any case, the rs699(C) allele encodes the threonine variant, which is associated with higher plasma angiotensin levels, and ultimately higher blood pressure leading to increased risk for hypertension associated disorders.This association was first reported in 1992 [pre-eclampsia (pregnancy-induced hypertension) [PMID 8513325, PMID 8348146] but did not present a risk in one African population [PMID 16059745].

Athleticism

Athletic performance is deeply intertwined with genetic factors that influence muscle fiber composition, aerobic capacity, and recovery rates. Genes affecting mitochondrial efficiency, oxygen utilization, and muscle hypertrophy can predict an individual's potential in endurance versus power-based sports. Genetic profiling can thus guide training regimens tailored to maximize athletic output.

Achilles Tendinopathy

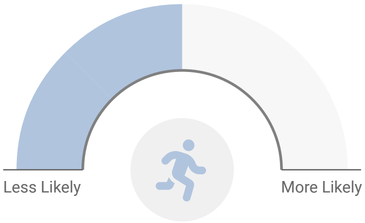

With the C/T genotype, your risk for Achilles Tendinopathy doesn’t stand out according to current data.

• **Gene:** MMP3 • **rsID:** rs679620 • **Your Genotype:** T/T>T/C • **Zygosity:** Heterozygous

Description

Achilles tendinopathy is characterized by the degeneration of collagen in the tendons connecting calf muscles to heel bones, leading to pain, swelling, and stiffness during and after activities like walking. More strenuous pursuits, such as running and jumping, can exacerbate these symptoms. The condition is often attributed to persistent overuse of the tendon without adequate rest, making it particularly prevalent among athletes, including runners.

Extra Information

The rs679620 AG genotype is associated with the typical likelihood of Achilles tendinopathy, as indicated by various studies (PMID(s): 19042922, 27222816, 28358823), while individuals with the GG genotype face a heightened risk for Achilles tendinopathy. In cases of Achilles tendon rupture (RUP), the MMP3 rs679620 GG genotype is notably more prevalent (44.0%) compared to controls (19.1%), where the AG genotype is more common (54.2%) (PMID: 19042922). Another study, albeit relatively small (~100 patients), found associations between Achilles tendinopathy risk and single nucleotide polymorphisms (SNPs) in the MMP3 gene. For rs679620, they found individuals with the (G;G) genotype exhibited an odds ratio of 2.5 (CI: 1.2 - 4.90, p = 0.010). Moreover, there's evidence of an interaction between rs679620(G) and COL5A1 rs12722(T) alleles, contributing to an increased risk for Achilles tendinopathy with a p-value of 0.006.

Endurance

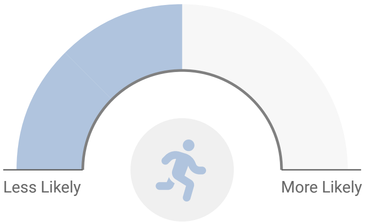

C/T genotype for MYBPC3 is not linked with significant changes in Endurance.

• **Gene:** MYBPC3 • **rsID:** rs1052373 • **Your Genotype:** C/C>C/T • **Zygosity:** Heterozygous

Description

An individuals endurance capacity is determined by many factors, including their muscle fibre typology, haemoglobin mass, mitochondrial biogenesis, maximal cardiac output, and maximal rate of oxygen consumption (VO2max), among others [118,119,120,121,122,123,124]. Indeed, there is evidence that these intermediate phenotypes have a substantial genetic influence, with literature indicating that genetic factors account for up to 70% of the variability in endurance-related traits [125]. Usually, genetic markers associated with endurance athlete status are determined by comparing allelic frequencies between endurance athletes (e.g., biathletes, road cyclists etc.) and controls.

Extra Information

The G-allele of rs1052373 in MYBPC3 (Myosin Binding Protein C, Cardiac) is linked to endurance. Al-Khelaifi, F.; et al. (2020) "Genome-Wide Association Study Reveals a Novel Association Between MYBPC3 Gene Polymorphism, Endurance Athlete Status, Aerobic Capacity and Steroid Metabolism" identified this association, suggesting a role in cardiovascular adaptation or muscle function beneficial for endurance. The reference allele is C (alternative T).

Endurance

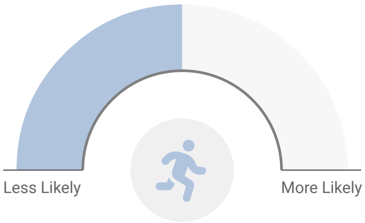

For your Endurance trait related to the UCP2 gene (rs660339), the A/A genotype doesn't show specific advantages.

• **Gene:** UCP2 • **rsID:** rs660339 • **Your Genotype:** G/G>A/A • **Zygosity:** Homozygous

Description

An individuals endurance capacity is determined by many factors, including their muscle fibre typology, haemoglobin mass, mitochondrial biogenesis, maximal cardiac output, and maximal rate of oxygen consumption (VO2max), among others [118,119,120,121,122,123,124]. Indeed, there is evidence that these intermediate phenotypes have a substantial genetic influence, with literature indicating that genetic factors account for up to 70% of the variability in endurance-related traits [125]. Usually, genetic markers associated with endurance athlete status are determined by comparing allelic frequencies between endurance athletes (e.g., biathletes, road cyclists etc.) and controls.

Extra Information

The T-allele (Val55) of rs660339 (Ala55Val) in UCP2 (Uncoupling Protein 2) is linked to endurance. Positive associations were reported by Ahmetov, I.I.; et al. (2008) "The use of molecular genetic methods for prognosis of aerobic and anaerobic performance in athletes," Ahmetov, I.I.; et al. (2009) "The combined impact of metabolic gene polymorphisms on elite endurance athlete status and related phenotypes," and Gronek, P.; et al. (2018) "Polygenic Study of Endurance-Associated Genetic Markers...in Polish Male Half Marathoners." These studies suggest the T-allele may enhance endurance, potentially by influencing energy efficiency or reducing oxidative stress. The reference allele is G (Ala55).

Endurance

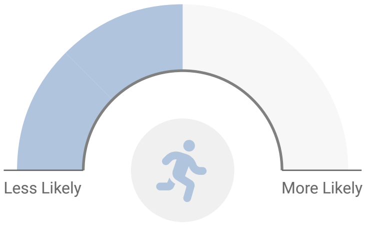

The C/G genotype for the NFATC4 gene doesn't strongly influence your endurance capacity.

• **Gene:** NFATC4 • **rsID:** rs2229309 • **Your Genotype:** G/G>G/C • **Zygosity:** Heterozygous

Description

An individuals endurance capacity is determined by many factors, including their muscle fibre typology, haemoglobin mass, mitochondrial biogenesis, maximal cardiac output, and maximal rate of oxygen consumption (VO2max), among others [118,119,120,121,122,123,124]. Indeed, there is evidence that these intermediate phenotypes have a substantial genetic influence, with literature indicating that genetic factors account for up to 70% of the variability in endurance-related traits [125]. Usually, genetic markers associated with endurance athlete status are determined by comparing allelic frequencies between endurance athletes (e.g., biathletes, road cyclists etc.) and controls.

Extra Information

The G-allele of rs2229309 in NFATC4 (Nuclear Factor of Activated T-cells Cytoplasmic 4) is linked to endurance. Ahmetov, I.I.; et al. (2009) "The combined impact of metabolic gene polymorphisms on elite endurance athlete status and related phenotypes" reported an association of the G-allele with endurance athlete status. NFATC4 is involved in muscle fiber type regulation. The reference allele is G (alternatives A,C).

Endurance

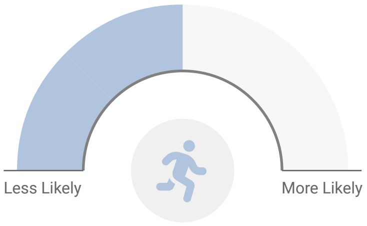

No notable effects are seen with the T/T genotype concerning your Endurance trait in relation to the TSHR gene.

• **Gene:** TSHR • **rsID:** rs7144481 • **Your Genotype:** C/C>T/T • **Zygosity:** Homozygous

Description

An individuals endurance capacity is determined by many factors, including their muscle fibre typology, haemoglobin mass, mitochondrial biogenesis, maximal cardiac output, and maximal rate of oxygen consumption (VO2max), among others [118,119,120,121,122,123,124]. Indeed, there is evidence that these intermediate phenotypes have a substantial genetic influence, with literature indicating that genetic factors account for up to 70% of the variability in endurance-related traits [125]. Usually, genetic markers associated with endurance athlete status are determined by comparing allelic frequencies between endurance athletes (e.g., biathletes, road cyclists etc.) and controls.

Extra Information

The C-allele of rs7144481 in TSHR (Thyroid Stimulating Hormone Receptor) is linked to endurance. A GWAS by Ahmetov, I.; et al. (2015) "Genome-wide association study identifies three novel genetic markers associated with elite endurance performance" identified this C-allele as associated with endurance status, suggesting a role in metabolic responses favorable for endurance. The reference allele is C (alternative T).

Endurance

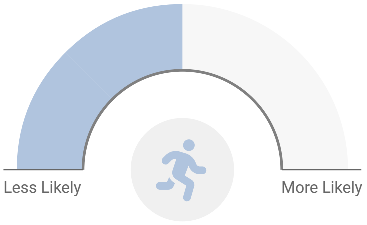

Your A/C genotype in the GABPB1 gene doesn't show significant effects on endurance.

• **Gene:** GABPB1 • **rsID:** rs12594956 • **Your Genotype:** C/C>C/A • **Zygosity:** Heterozygous

Description

An individuals endurance capacity is determined by many factors, including their muscle fibre typology, haemoglobin mass, mitochondrial biogenesis, maximal cardiac output, and maximal rate of oxygen consumption (VO2max), among others [118,119,120,121,122,123,124]. Indeed, there is evidence that these intermediate phenotypes have a substantial genetic influence, with literature indicating that genetic factors account for up to 70% of the variability in endurance-related traits [125]. Usually, genetic markers associated with endurance athlete status are determined by comparing allelic frequencies between endurance athletes (e.g., biathletes, road cyclists etc.) and controls.

Extra Information

The A-allele of rs12594956 in GABPB1 (GA-Binding Protein subunit Beta-1), also implicating the NRF-2 pathway, is linked to endurance. Positive associations with endurance capacity or athlete status were reported by He, Z.; et al. (2007) "NRF2 genotype improves endurance capacity in response to training" and Eynon, N.; et al. (2013) "The rs12594956 polymorphism in the NRF-2 gene is associated with top-level Spanish athlete's performance status." GABPB1 and NRF-2 are involved in regulating mitochondrial biogenesis, crucial for aerobic energy production. The reference allele is C (alternatives A,G).

Endurance

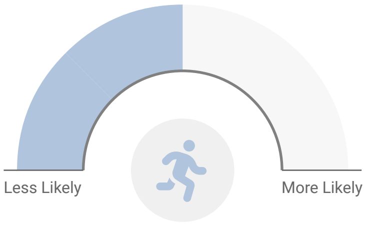

With A/G in RBFOX1, no clear evidence points to an effect on your endurance.

• **Gene:** RBFOX1 • **rsID:** rs7191721 • **Your Genotype:** A/A>A/G • **Zygosity:** Heterozygous

Description

An individuals endurance capacity is determined by many factors, including their muscle fibre typology, haemoglobin mass, mitochondrial biogenesis, maximal cardiac output, and maximal rate of oxygen consumption (VO2max), among others [118,119,120,121,122,123,124]. Indeed, there is evidence that these intermediate phenotypes have a substantial genetic influence, with literature indicating that genetic factors account for up to 70% of the variability in endurance-related traits [125]. Usually, genetic markers associated with endurance athlete status are determined by comparing allelic frequencies between endurance athletes (e.g., biathletes, road cyclists etc.) and controls.

Extra Information

The G-allele of rs7191721 in the RBFOX1 gene is linked to endurance. Ahmetov, I.; et al. (2015) "Genome-wide association study identifies three novel genetic markers associated with elite endurance performance" found this G-allele significantly more prevalent among elite endurance athletes. This suggests the G-allele (reference A, alternatives C,G,T) may contribute to favorable endurance performance, possibly through splicing regulation influencing muscle characteristics.

Endurance

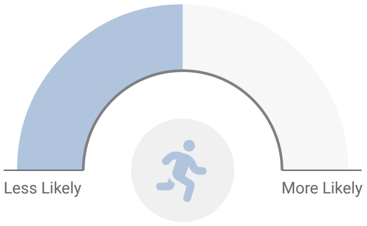

For G/T, there's no notable evidence for an effect on endurance in this genetic context.

• **Gene:** NFIA-AS2 • **rsID:** rs1572312 • **Your Genotype:** G/G>G/T • **Zygosity:** Heterozygous

Description

An individuals endurance capacity is determined by many factors, including their muscle fibre typology, haemoglobin mass, mitochondrial biogenesis, maximal cardiac output, and maximal rate of oxygen consumption (VO2max), among others [118,119,120,121,122,123,124]. Indeed, there is evidence that these intermediate phenotypes have a substantial genetic influence, with literature indicating that genetic factors account for up to 70% of the variability in endurance-related traits [125]. Usually, genetic markers associated with endurance athlete status are determined by comparing allelic frequencies between endurance athletes (e.g., biathletes, road cyclists etc.) and controls.

Extra Information

The C-allele of rs1572312 in NFIA-AS2 (NFIA Antisense RNA 2) is linked to endurance. Ahmetov, I.; et al. (2015) "Genome-wide association study identifies three novel genetic markers associated with elite endurance performance" and Malczewska-Lenczowska, J.; et al. (2022) "HIF-1α and NFIA-AS2 Polymorphisms as Potential Determinants of Total Hemoglobin Mass in Endurance Athletes" (linking it to hemoglobin mass) support this. The reference allele is G (alternative T).

Endurance

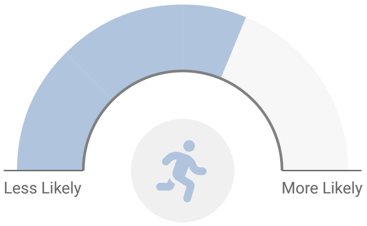

With the G/G genotype in the TRPM2 gene, you could have enhanced endurance performance based on certain studies.

• **Gene:** TRPM2 • **rsID:** rs1785440 • **Your Genotype:** A/A>G/G • **Zygosity:** Homozygous

Description

An individuals endurance capacity is determined by many factors, including their muscle fibre typology, haemoglobin mass, mitochondrial biogenesis, maximal cardiac output, and maximal rate of oxygen consumption (VO2max), among others [118,119,120,121,122,123,124]. Indeed, there is evidence that these intermediate phenotypes have a substantial genetic influence, with literature indicating that genetic factors account for up to 70% of the variability in endurance-related traits [125]. Usually, genetic markers associated with endurance athlete status are determined by comparing allelic frequencies between endurance athletes (e.g., biathletes, road cyclists etc.) and controls.

Extra Information

The G-allele of rs1785440 in the TRPM2 gene (oxidative stress response) is linked to endurance. Bulgay, C.; et al. (2023) "Exome-Wide Association Study of Competitive Performance in Elite Athletes" reported an association of this G-allele with competitive performance, potentially by aiding in managing exercise-induced oxidative stress or modulating calcium signaling. The reference allele is A (alternatives G,T).

Endurance

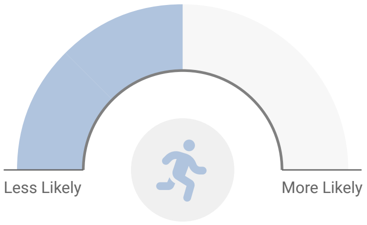

For your Endurance trait with the SPEG gene (rs7564856), the A/A genotype doesn't indicate strong effects on your endurance.

• **Gene:** SPEG • **rsID:** rs7564856 • **Your Genotype:** G/G>A/A • **Zygosity:** Homozygous

Description

An individuals endurance capacity is determined by many factors, including their muscle fibre typology, haemoglobin mass, mitochondrial biogenesis, maximal cardiac output, and maximal rate of oxygen consumption (VO2max), among others [118,119,120,121,122,123,124]. Indeed, there is evidence that these intermediate phenotypes have a substantial genetic influence, with literature indicating that genetic factors account for up to 70% of the variability in endurance-related traits [125]. Usually, genetic markers associated with endurance athlete status are determined by comparing allelic frequencies between endurance athletes (e.g., biathletes, road cyclists etc.) and controls.

Extra Information

The G-allele of rs7564856 in SPEG (Striated Muscle Enriched Protein Kinase) is linked to endurance. Kusić, D.; et al. (2020) "Striated muscle-specific serine/threonine-protein kinase β segregates with high versus low responsiveness to endurance exercise training" reported that this kinase (SPEG) segregates with endurance training responsiveness, suggesting a role for this gene in adaptation to endurance exercise. The reference allele is G (alternative A).

Endurance

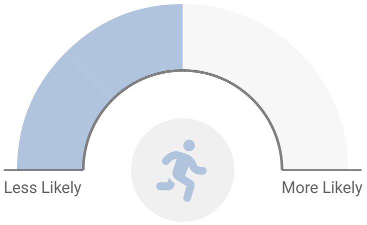

In the context of endurance, the A/T genotype for the VEGFR2 gene doesn't seem to affect your performance notably.

• **Gene:** VEGFR2 • **rsID:** rs1870377 • **Your Genotype:** T/T>T/A • **Zygosity:** Heterozygous

Description

An individuals endurance capacity is determined by many factors, including their muscle fibre typology, haemoglobin mass, mitochondrial biogenesis, maximal cardiac output, and maximal rate of oxygen consumption (VO2max), among others [118,119,120,121,122,123,124]. Indeed, there is evidence that these intermediate phenotypes have a substantial genetic influence, with literature indicating that genetic factors account for up to 70% of the variability in endurance-related traits [125]. Usually, genetic markers associated with endurance athlete status are determined by comparing allelic frequencies between endurance athletes (e.g., biathletes, road cyclists etc.) and controls.

Extra Information

The A-allele of rs1870377 in VEGFR2 (KDR gene, crucial for angiogenesis) is linked to endurance. Ahmetov, I.I.; et al. (2009) "Association of the VEGFR2 gene His472Gln polymorphism with endurance-related phenotypes" reported a positive association, suggesting it may improve endurance, possibly by enhancing muscle capillarization and oxygen delivery. The reference allele is T. (Note: JSON for ref 237 was empty).

Endurance

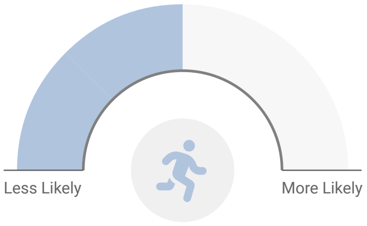

In your case, with the VEGFA gene and endurance, the C/G genotype doesn't indicate a strong effect.

• **Gene:** VEGFA • **rsID:** rs2010963 • **Your Genotype:** C/C>C/G • **Zygosity:** Heterozygous

Description

An individuals endurance capacity is determined by many factors, including their muscle fibre typology, haemoglobin mass, mitochondrial biogenesis, maximal cardiac output, and maximal rate of oxygen consumption (VO2max), among others [118,119,120,121,122,123,124]. Indeed, there is evidence that these intermediate phenotypes have a substantial genetic influence, with literature indicating that genetic factors account for up to 70% of the variability in endurance-related traits [125]. Usually, genetic markers associated with endurance athlete status are determined by comparing allelic frequencies between endurance athletes (e.g., biathletes, road cyclists etc.) and controls.

Extra Information

The C-allele of rs2010963 in VEGFA (Vascular Endothelial Growth Factor A) is linked to endurance. Ahmetov, I.I.; et al. (2009) "The combined impact of metabolic gene polymorphisms on elite endurance athlete status and related phenotypes," Prior, S.J.; et al. (2006) "DNA sequence variation in the promoter region of the VEGF gene impacts VEGF gene expression and maximal oxygen consumption," and Ahmetov, I.I.; et al. (2008) "Polymorphism of the vascular endothelial growth factor gene (VEGF) and aerobic performance in athletes" reported positive associations. These suggest the C-allele may enhance endurance, likely due to VEGFA's role in angiogenesis (new blood vessel formation), which improves oxygen delivery to muscles. The reference allele is C (alternatives G,T).

Endurance

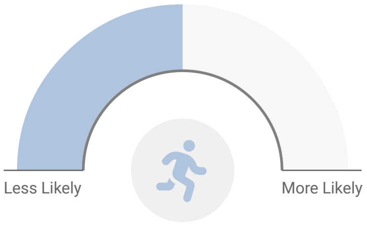

The C/G genotype hasn't been observed to substantially impact endurance in the AQP1 gene.

• **Gene:** AQP1 • **rsID:** rs1049305 • **Your Genotype:** G/G>G/C • **Zygosity:** Heterozygous

Description

An individuals endurance capacity is determined by many factors, including their muscle fibre typology, haemoglobin mass, mitochondrial biogenesis, maximal cardiac output, and maximal rate of oxygen consumption (VO2max), among others [118,119,120,121,122,123,124]. Indeed, there is evidence that these intermediate phenotypes have a substantial genetic influence, with literature indicating that genetic factors account for up to 70% of the variability in endurance-related traits [125]. Usually, genetic markers associated with endurance athlete status are determined by comparing allelic frequencies between endurance athletes (e.g., biathletes, road cyclists etc.) and controls.

Extra Information

The C-allele of rs1049305 in AQP1 (Aquaporin 1, water channel) is linked to endurance. Martínez, J.L.; et al. (2009) "Aquaporin-1 gene DNA variation predicts performance in Hispanic marathon runners," Rivera, M.A.; et al. (2011) "AQP-1 association with body fluid loss in 10-km runners," and Saunders, C.J.; et al. (2015) "A variant within the AQP1 30 -untranslated region is associated with running performance..." reported positive associations, potentially influencing hydration and thermoregulation. The reference allele is G (alternatives A,C).

Endurance

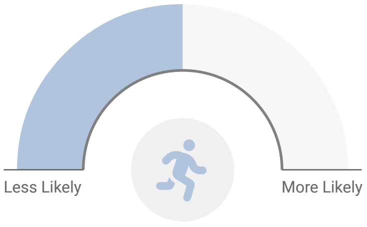

Holding the C/T genotype in your COL5A1 gene doesn't have a notable effect on endurance.

• **Gene:** COL5A1 • **rsID:** rs12722 • **Your Genotype:** C/C>C/T • **Zygosity:** Heterozygous

Description

An individuals endurance capacity is determined by many factors, including their muscle fibre typology, haemoglobin mass, mitochondrial biogenesis, maximal cardiac output, and maximal rate of oxygen consumption (VO2max), among others [118,119,120,121,122,123,124]. Indeed, there is evidence that these intermediate phenotypes have a substantial genetic influence, with literature indicating that genetic factors account for up to 70% of the variability in endurance-related traits [125]. Usually, genetic markers associated with endurance athlete status are determined by comparing allelic frequencies between endurance athletes (e.g., biathletes, road cyclists etc.) and controls.

Extra Information

The T-allele of rs12722 in COL5A1 (Collagen Type V Alpha 1 Chain) is linked to endurance. Posthumus, M.; et al. (2011) "The COL5A1 gene: A novel marker of endurance running performance" and Brown, J.C.; et al. (2011) "The COL5A1 gene, ultra-marathon running performance, and range of motion" reported positive associations, suggesting the T-allele might influence ligament/tendon properties favorably for endurance running. The reference allele is C.

Name:
DoB:
Gender:
Report ID: WBWG_01_P001_262
Patient ID: 01_P001_262
Date: 6/7/2025

Endurance

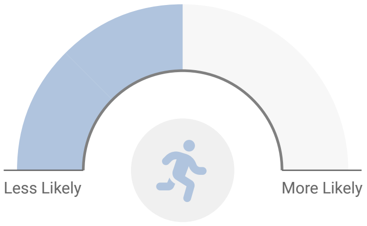

Having G/G in ACE hasn’t shown notable impact on endurance traits.

• **Gene:** ACE • **rsID:** rs4343 • **Your Genotype:** G/G • **Zygosity:** homo_ref

Description

An individuals endurance capacity is determined by many factors, including their muscle fibre typology, haemoglobin mass, mitochondrial biogenesis, maximal cardiac output, and maximal rate of oxygen consumption (VO2max), among others [118,119,120,121,122,123,124]. Indeed, there is evidence that these intermediate phenotypes have a substantial genetic influence, with literature indicating that genetic factors account for up to 70% of the variability in endurance-related traits [125]. Usually, genetic markers associated with endurance athlete status are determined by comparing allelic frequencies between endurance athletes (e.g., biathletes, road cyclists etc.) and controls.

Extra Information

The A-allele of rs4343 in ACE (often linked to I-allele of ACE I/D) is linked to endurance. A multitude of studies support this: Montgomery, H.E.; et al. (1998), Gayagay, G.; et al. (1998), Nazarov, I.B.; et al. (2001), Myerson, S.; et al. (1999), Jelakovic, B.; et al. (2000), Ahmetov, I.I.; et al. (2008), Alvarez, R.; et al. (2000), Collins, M.; et al. (2004), Lucía, A.; et al. (2005), Scanavini, D.; et al. (2002), Turgut, G.; et al. (2004), Tsianos, G.; et al. (2004), Cieszczyk, P.; et al. (2009), Min, S.K.; et al. (2009), Shenoy, S.; et al. (2010) (Refs 134 & 141 were empty). However, many studies including Lucía, A.; et al. (2005) (also listed as positive), Ash, G.I.; et al. (2011), Tobina, T.; et al. (2010), Ahmetov, I.I.; et al. (2009), Scott, R.A.; et al. (2005), Rankinen, T.; et al. (2000), Taylor, R.R.; et al. (1999), Orysiak, J.; et al. (2013), Ginevičienė, V.; et al. (2011), Muniesa, C.A.; et al. (2010), Papadimitriou, I.D.; et al. (2018), and Varillas-Delgado, D.; et al. (2022) report negative or controversial results. (Ref 145 empty). The reference allele is G.

Endurance

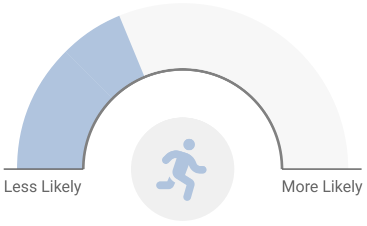

Possessing the G/G genotype in ACE could mean a reduced predisposition towards endurance traits.

• **Gene:** ACE • **rsID:** rs4341 • **Your Genotype:** G/G • **Zygosity:** homo_ref

Description

An individuals endurance capacity is determined by many factors, including their muscle fibre typology, haemoglobin mass, mitochondrial biogenesis, maximal cardiac output, and maximal rate of oxygen consumption (VO2max), among others [118,119,120,121,122,123,124]. Indeed, there is evidence that these intermediate phenotypes have a substantial genetic influence, with literature indicating that genetic factors account for up to 70% of the variability in endurance-related traits [125]. Usually, genetic markers associated with endurance athlete status are determined by comparing allelic frequencies between endurance athletes (e.g., biathletes, road cyclists etc.) and controls.

Extra Information

The C-allele of rs4341 in ACE (often linked to I-allele of ACE I/D) is linked to endurance. Many studies (as detailed for rs4343 above, refs [14,15,16,128-141]) support the I-allele (or linked SNPs like rs4341*C) for endurance. However, many others (refs [133,142-153]) provide negative or controversial evidence, highlighting the complexity of ACE genetics in performance. The reference allele is G.

Endurance

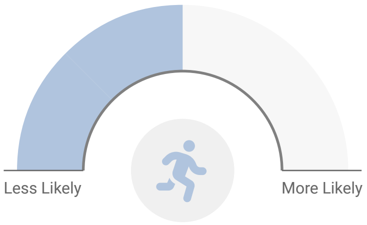

For your Endurance trait with the ADRB3 gene (rs4994), having the A/A genotype doesn't reveal any notable effects on endurance.

• **Gene:** ADRB3 • **rsID:** rs4994 • **Your Genotype:** A/A • **Zygosity:** homo_ref

Description

An individuals endurance capacity is determined by many factors, including their muscle fibre typology, haemoglobin mass, mitochondrial biogenesis, maximal cardiac output, and maximal rate of oxygen consumption (VO2max), among others [118,119,120,121,122,123,124]. Indeed, there is evidence that these intermediate phenotypes have a substantial genetic influence, with literature indicating that genetic factors account for up to 70% of the variability in endurance-related traits [125]. Usually, genetic markers associated with endurance athlete status are determined by comparing allelic frequencies between endurance athletes (e.g., biathletes, road cyclists etc.) and controls.

Extra Information

The G-allele (Arg64) of rs4994 (Trp64Arg) in ADRB3 (lipolysis) is studied for endurance. Santiago, C.; et al. (2011) "Trp64Arg polymorphism in ADRB3 gene is associated with elite endurance performance" reported a positive association. The role may relate to metabolic rate or fat utilization, though evidence can be population-specific. (Note: Ref 170 was empty). The reference allele is A (Trp64).

Name:
DoB:
Gender:

Report ID: WBWG_01_P001_262
Patient ID: 01_P001_262
Date: 6/7/2025

Endurance

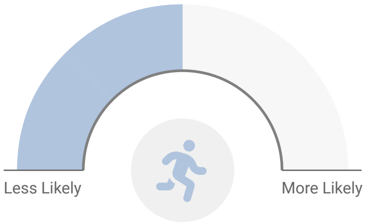

There's no strong indication for the A/A genotype in affecting your endurance traits based on the AGTR2 gene (rs11091046).

- **Gene:** AGTR2
- **rsID:** rs11091046
- **Your Genotype:** A/A
- **Zygosity:** homo_ref

Description

An individuals endurance capacity is determined by many factors, including their muscle fibre typology, haemoglobin mass, mitochondrial biogenesis, maximal cardiac output, and maximal rate of oxygen consumption (VO2max), among others [118,119,120,121,122,123,124]. Indeed, there is evidence that these intermediate phenotypes have a substantial genetic influence, with literature indicating that genetic factors account for up to 70% of the variability in endurance-related traits [125]. Usually, genetic markers associated with endurance athlete status are determined by comparing allelic frequencies between endurance athletes (e.g., biathletes, road cyclists etc.) and controls.

Extra Information

The C-allele of rs11091046 in AGTR2 (X chromosome, Angiotensin II Receptor Type 2) is linked to endurance. Mustafina, L.J.; et al. (2014) "AGTR2 gene polymorphism is associated with muscle fibre composition, athletic status and aerobic performance" reported an association with endurance-related phenotypes (the C-allele specifically with higher percentage of slow-twitch fibers). However, Guilherme, J.P.L.F.; et al. (2018) "The AGTR2 rs11091046 (A>C) polymorphism and power athletic status in top-level Brazilian athletes" found the A-allele associated with power, indicating conflicting or trait-specific effects for the alleles of this SNP. The reference allele is A (alternatives C,T).

Endurance

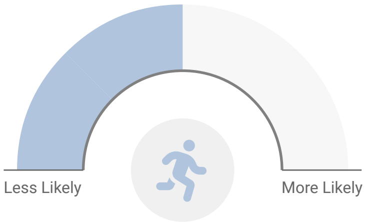

For your Endurance trait with the AMPD1 gene (rs17602729), G/G doesn't have a notable effect.

- **Gene:** AMPD1
- **rsID:** rs17602729
- **Your Genotype:** G/G
- **Zygosity:** homo_ref

Description

An individuals endurance capacity is determined by many factors, including their muscle fibre typology, haemoglobin mass, mitochondrial biogenesis, maximal cardiac output, and maximal rate of oxygen consumption (VO2max), among others [118,119,120,121,122,123,124]. Indeed, there is evidence that these intermediate phenotypes have a substantial genetic influence, with literature indicating that genetic factors account for up to 70% of the variability in endurance-related traits [125]. Usually, genetic markers associated with endurance athlete status are determined by comparing allelic frequencies between endurance athletes (e.g., biathletes, road cyclists etc.) and controls.

Extra Information

The C-allele of rs17602729 (C34T) in AMPD1 (adenosine monophosphate deaminase 1) is linked to endurance. Rubio, J.C.; et al. (2005) "Frequency of the C34T mutation of the AMPD1 gene in world-class endurance athletes...," Varillas-Delgado, D.; et al. (2022) "Genetic profiles to identify talents in elite endurance athletes...," Rico-Sanz, J.; et al. (2003) "Associations between cardiorespiratory responses to exercise and the C34T AMPD1 gene polymorphism...," Thomaes, T.; et al. (2011) "A genetic predisposition score for muscular endophenotypes predicts the increase in aerobic power after training...," and Ciȩszczyk, P.; et al. (2011) "Is the C34T polymorphism of the AMPD1 gene associated with athlete performance in rowing?" reported positive findings. Ginevičienė, V.; et al. (2014) "AMPD1 rs17602729 is associated with physical performance of sprint and power..." found an association with power, representing a conflicting outcome for endurance. The C-allele (normal enzyme) may prevent exercise intolerance. The reference allele is G.

Endurance

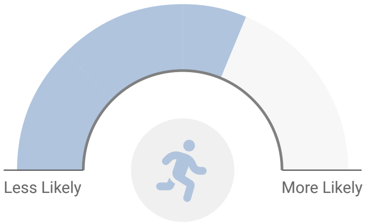

With an A/A genotype in the CDKN1A gene (rs236448), you might have a genetic edge in endurance tasks.

- **Gene:** CDKN1A
- **rsID:** rs236448
- **Your Genotype:** A/A
- **Zygosity:** homo_ref

Description

An individuals endurance capacity is determined by many factors, including their muscle fibre typology, haemoglobin mass, mitochondrial biogenesis, maximal cardiac output, and maximal rate of oxygen consumption (VO2max), among others [118,119,120,121,122,123,124]. Indeed, there is evidence that these intermediate phenotypes have a substantial genetic influence, with literature indicating that genetic factors account for up to 70% of the variability in endurance-related traits [125]. Usually, genetic markers associated with endurance athlete status are determined by comparing allelic frequencies between endurance athletes (e.g., biathletes, road cyclists etc.) and controls.

Extra Information

The A-allele of rs236448 in CDKN1A (Cyclin Dependent Kinase Inhibitor 1A, also p21) is linked to endurance-related muscle fiber composition. Semenova, E.A.; et al. (2022) "Genome-Wide Association Study Identifies CDKN1A as a Novel Locus Associated with Muscle Fiber Composition" found the A-allele associated with a higher percentage of slow-twitch (Type I) muscle fibers, which are characteristic of endurance athletes due to their high oxidative capacity and fatigue resistance. The reference allele is A (alternative C).

Endurance

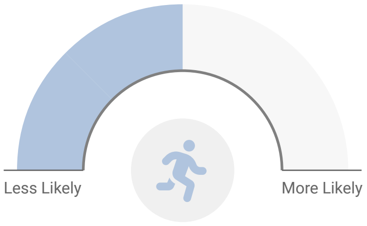

For T/T at CKM, we don't find any major impact on endurance traits.

• **Gene:** CKM • **rsID:** rs8111989 • **Your Genotype:** T/T • **Zygosity:** homo_ref

Description

An individuals endurance capacity is determined by many factors, including their muscle fibre typology, haemoglobin mass, mitochondrial biogenesis, maximal cardiac output, and maximal rate of oxygen consumption (VO2max), among others [118,119,120,121,122,123,124]. Indeed, there is evidence that these intermediate phenotypes have a substantial genetic influence, with literature indicating that genetic factors account for up to 70% of the variability in endurance-related traits [125]. Usually, genetic markers associated with endurance athlete status are determined by comparing allelic frequencies between endurance athletes (e.g., biathletes, road cyclists etc.) and controls.

Extra Information

The A-allele of rs8111989 in CKM (muscle creatine kinase) is linked to endurance. Rivera, M.A.; et al. (1997) "Muscle-specific creatine kinase gene polymorphism and VO2max in the HERITAGE Family Study," Rivera, M.A.; et al. (1999) "Linkage between a muscle-specific CK gene marker and VO2max in the HERITAGE Family Study," and Fedotovskaya, O.N.; et al. (2012) "Association of muscle-specific creatine kinase (CKMM) gene polymorphism with physical performance of athletes" reported positive associations. However, Lucía, A.; et al. (2005) "Is there an association between ACE and CKMM polymorphisms and cycling performance status during 3-week races?" and Martínez, J.L.; et al. (2009) "Lack of an Association Between CKMM Genotype and Endurance Performance Level in Hispanic Marathon Runners" found conflicting results. The reference allele is T.

Endurance

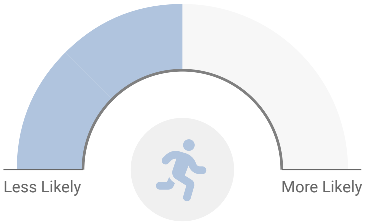

Having a T/T genotype does not appear to strongly influence endurance in any notable way.

• **Gene:** FTO • **rsID:** rs9939609 • **Your Genotype:** T/T • **Zygosity:** homo_ref

Description

An individuals endurance capacity is determined by many factors, including their muscle fibre typology, haemoglobin mass, mitochondrial biogenesis, maximal cardiac output, and maximal rate of oxygen consumption (VO2max), among others [118,119,120,121,122,123,124]. Indeed, there is evidence that these intermediate phenotypes have a substantial genetic influence, with literature indicating that genetic factors account for up to 70% of the variability in endurance-related traits [125]. Usually, genetic markers associated with endurance athlete status are determined by comparing allelic frequencies between endurance athletes (e.g., biathletes, road cyclists etc.) and controls.

Extra Information

The T-allele of rs9939609 in FTO (Fat Mass and Obesity-Associated Protein) is linked to endurance. Guilherme, J.; et al. (2019) "The A-allele of the FTO gene rs9939609 polymorphism is associated with decreased proportion of slow oxidative muscle fibers..." (paradoxically, as A is alt to T) and Zmijewski, P.; & Leońska-Duniec, A. (2021) "Association between the FTO A/T Polymorphism and Elite Athlete Status in Caucasian Swimmers" (focusing on A vs T). Eynon, N.; et al. (2013) "The FTO A/T polymorphism and elite athletic performance..." reported conflicting results. The T-allele is the reference; A is alternative. The literature is complex, often linking the A-allele (risk for obesity) to less favorable endurance profiles.

Endurance

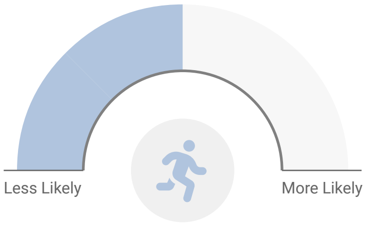

For your Endurance trait with the GABPB1 gene (rs7181866), having the A/A genotype doesn't show a strong impact, so your endurance might be determined more by other factors.

• **Gene:** GABPB1 • **rsID:** rs7181866 • **Your Genotype:** A/A • **Zygosity:** homo_ref

Description

An individuals endurance capacity is determined by many factors, including their muscle fibre typology, haemoglobin mass, mitochondrial biogenesis, maximal cardiac output, and maximal rate of oxygen consumption (VO2max), among others [118,119,120,121,122,123,124]. Indeed, there is evidence that these intermediate phenotypes have a substantial genetic influence, with literature indicating that genetic factors account for up to 70% of the variability in endurance-related traits [125]. Usually, genetic markers associated with endurance athlete status are determined by comparing allelic frequencies between endurance athletes (e.g., biathletes, road cyclists etc.) and controls.

Extra Information

The G-allele of rs7181866 near GABPB1 (mitochondrial biogenesis) is linked to endurance. He, Z.; et al. (2007) "NRF2 genotype improves endurance capacity in response to training" and Maciejewska-Karłowska, A.; et al. (2012) "The GABPB1 gene A/G polymorphism in Polish rowers" reported positive associations. However, Eynon, N.; et al. (2013) "The rs12594956 polymorphism in the NRF-2 gene is associated with top-level Spanish athlete's performance status" (focusing on NRF2) is noted as a controversial result in the broader pathway context. The reference allele is A.

Name: Report ID: WBWG_01_P001_262
DoB: Patient ID: 01_P001_262
Gender: Date: 6/7/2025

Endurance

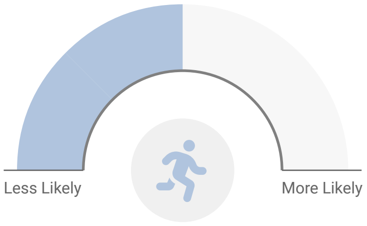

With the A/A genotype for the GSTP1 gene (rs1695), current data doesn't suggest notable differences in endurance.

• **Gene:** GSTP1 • **rsID:** rs1695 • **Your Genotype:** A/A • **Zygosity:** homo_ref

Description

An individuals endurance capacity is determined by many factors, including their muscle fibre typology, haemoglobin mass, mitochondrial biogenesis, maximal cardiac output, and maximal rate of oxygen consumption (VO2max), among others [118,119,120,121,122,123,124]. Indeed, there is evidence that these intermediate phenotypes have a substantial genetic influence, with literature indicating that genetic factors account for up to 70% of the variability in endurance-related traits [125]. Usually, genetic markers associated with endurance athlete status are determined by comparing allelic frequencies between endurance athletes (e.g., biathletes, road cyclists etc.) and controls.

Extra Information

The G-allele of rs1695 (Ile105Val) in GSTP1 (Glutathione S-Transferase Pi 1) is linked to endurance. Zarebska, A.; et al. (2014) "The GSTP1 c.313A>G polymorphism modulates the cardiorespiratory response to aerobic training" and Zarebska, A.; et al. (2017) "GSTP1 c.313A>G polymorphism in Russian and Polish athletes" reported positive associations, possibly via its role in antioxidant defense. The reference allele is A (Ile105).

Endurance

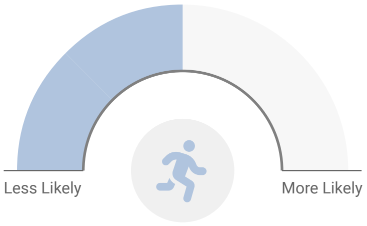

You have the C/C genotype in the HFE gene, which hasn't shown a strong link to Endurance.

• **Gene:** HFE • **rsID:** rs1799945 • **Your Genotype:** C/C • **Zygosity:** homo_ref

Description

An individuals endurance capacity is determined by many factors, including their muscle fibre typology, haemoglobin mass, mitochondrial biogenesis, maximal cardiac output, and maximal rate of oxygen consumption (VO2max), among others [118,119,120,121,122,123,124]. Indeed, there is evidence that these intermediate phenotypes have a substantial genetic influence, with literature indicating that genetic factors account for up to 70% of the variability in endurance-related traits [125]. Usually, genetic markers associated with endurance athlete status are determined by comparing allelic frequencies between endurance athletes (e.g., biathletes, road cyclists etc.) and controls.

Extra Information

The G-allele (H63) of rs1799945 (H63D) in HFE (Hemochromatosis gene) is linked to endurance. Varillas-Delgado, D.; et al. (2022) "Genetic profiles to identify talents in elite endurance athletes...," Deugnier, Y.; et al. (2002) "Increased body iron stores in elite road cyclists," Chicharro, J.L.; et al. (2004) "Mutations in the hereditary haemochromatosis gene HFE in professional endurance athletes," Hermine, O.; et al. (2015) "Eighty percent of French sport winners...have mutations in the hemochromatosis HFE gene," and Semenova, E.A.; et al. (2020) "The association of HFE gene H63D polymorphism with endurance athlete status..." reported positive associations, possibly via iron metabolism effects. The reference allele is C (D63).

Endurance

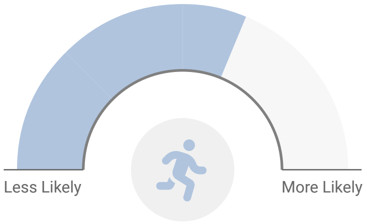

Having G/G in PPARA might really help you excel in endurance sports with an advantageous endurance capacity.

• **Gene:** PPARA • **rsID:** rs4253778 • **Your Genotype:** G/G • **Zygosity:** homo_ref

Description

An individuals endurance capacity is determined by many factors, including their muscle fibre typology, haemoglobin mass, mitochondrial biogenesis, maximal cardiac output, and maximal rate of oxygen consumption (VO2max), among others [118,119,120,121,122,123,124]. Indeed, there is evidence that these intermediate phenotypes have a substantial genetic influence, with literature indicating that genetic factors account for up to 70% of the variability in endurance-related traits [125]. Usually, genetic markers associated with endurance athlete status are determined by comparing allelic frequencies between endurance athletes (e.g., biathletes, road cyclists etc.) and controls.

Extra Information

The G-allele of rs4253778 in PPARA is linked to endurance. Ahmetov, I.I.; et al. (2006) "PPARalpha gene variation and physical performance in Russian athletes," Maciejewska, A.; et al. (2011) "Variation in the PPARα gene in Polish rowers," and Tural, E.; et al. (2014) "PPAR-α and PPARGC1A gene variants have strong effects on aerobic performance of Turkish elite endurance athletes" reported positive associations. (Ref 20 was empty). The G-allele may influence lipid metabolism and energy substrate utilization favorable for endurance. The reference allele is G (alternatives C,T).

Name:
DoB:
Gender:

Report ID: WBWG_01_P001_262
Patient ID: 01_P001_262
Date: 6/7/2025

Endurance

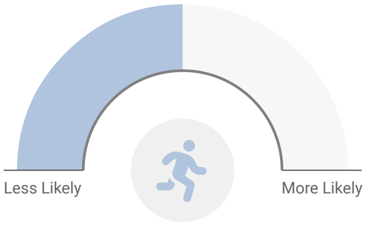

The G/G genotype in PPARGC1B lacks robust data linking it to endurance differences.

• **Gene:** PPARGC1B • **rsID:** rs7732671 • **Your Genotype:** G/G • **Zygosity:** homo_ref

Description

An individuals endurance capacity is determined by many factors, including their muscle fibre typology, haemoglobin mass, mitochondrial biogenesis, maximal cardiac output, and maximal rate of oxygen consumption (VO2max), among others [118,119,120,121,122,123,124]. Indeed, there is evidence that these intermediate phenotypes have a substantial genetic influence, with literature indicating that genetic factors account for up to 70% of the variability in endurance-related traits [125]. Usually, genetic markers associated with endurance athlete status are determined by comparing allelic frequencies between endurance athletes (e.g., biathletes, road cyclists etc.) and controls.

Extra Information

The C-allele of rs7732671 in PPARGC1B (energy metabolism, mitochondrial biogenesis) is linked to endurance. Ahmetov, I.I.; et al. (2009) "The combined impact of metabolic gene polymorphisms on elite endurance athlete status and related phenotypes" found the C-allele more prevalent among endurance athletes, suggesting enhanced mitochondrial function. The reference allele is G. (Note: JSON for ref 226 was empty).

Endurance

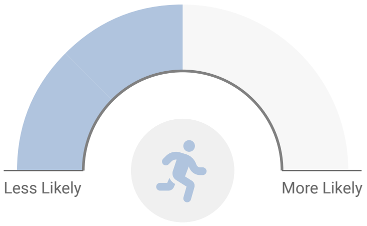

There aren't any known effects on endurance from the C/C genotype in PRDM1.

• **Gene:** PRDM1 • **rsID:** rs10499043 • **Your Genotype:** C/C • **Zygosity:** homo_ref

Description

An individuals endurance capacity is determined by many factors, including their muscle fibre typology, haemoglobin mass, mitochondrial biogenesis, maximal cardiac output, and maximal rate of oxygen consumption (VO2max), among others [118,119,120,121,122,123,124]. Indeed, there is evidence that these intermediate phenotypes have a substantial genetic influence, with literature indicating that genetic factors account for up to 70% of the variability in endurance-related traits [125]. Usually, genetic markers associated with endurance athlete status are determined by comparing allelic frequencies between endurance athletes (e.g., biathletes, road cyclists etc.) and controls.

Extra Information

The T-allele of rs10499043 in PRDM1 (PR Domain Containing 1) is linked to endurance, particularly the trainability of VO2max. Studies by Bouchard, C.; et al. (2011) "Genomic predictors of the maximal O2 uptake response to standardized exercise training programs" and Hall, E.C.R.; et al. (2021) "Genetic Polymorphisms Related to VO2max Adaptation Are Associated with Elite Rugby Union Status and Competitive Marathon Performance" identified this association. PRDM1 is a transcription factor involved in cellular differentiation and has been implicated in muscle adaptation to exercise. The reference allele is C (alternatives G,T).

Endurance

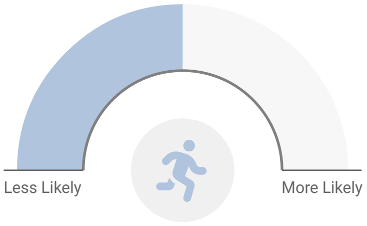

For the C/C combination in SIRT1, no clear endurance impact is observed.

• **Gene:** SIRT1 • **rsID:** rs41299232 • **Your Genotype:** C/C • **Zygosity:** homo_ref

Description

An individuals endurance capacity is determined by many factors, including their muscle fibre typology, haemoglobin mass, mitochondrial biogenesis, maximal cardiac output, and maximal rate of oxygen consumption (VO2max), among others [118,119,120,121,122,123,124]. Indeed, there is evidence that these intermediate phenotypes have a substantial genetic influence, with literature indicating that genetic factors account for up to 70% of the variability in endurance-related traits [125]. Usually, genetic markers associated with endurance athlete status are determined by comparing allelic frequencies between endurance athletes (e.g., biathletes, road cyclists etc.) and controls.

Extra Information

The G-allele of rs41299232 in SIRT1 (Sirtuin 1) is linked to endurance. Bulgay, C.; et al. (2023) "Exome-Wide Association Study of Competitive Performance in Elite Athletes" reported this association. SIRT1 is involved in metabolic regulation and cellular stress response. The reference allele is C (alternatives A,G).

Endurance

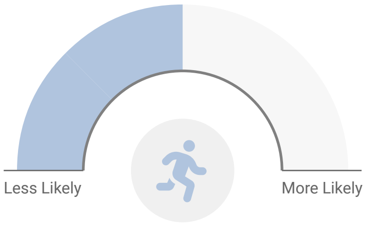

No strong relation found between the G/G genotype and endurance in the TFAM gene.

• **Gene:** TFAM • **rsID:** rs1937 • **Your Genotype:** G/G • **Zygosity:** homo_ref

Description

An individuals endurance capacity is determined by many factors, including their muscle fibre typology, haemoglobin mass, mitochondrial biogenesis, maximal cardiac output, and maximal rate of oxygen consumption (VO2max), among others [118,119,120,121,122,123,124]. Indeed, there is evidence that these intermediate phenotypes have a substantial genetic influence, with literature indicating that genetic factors account for up to 70% of the variability in endurance-related traits [125]. Usually, genetic markers associated with endurance athlete status are determined by comparing allelic frequencies between endurance athletes (e.g., biathletes, road cyclists etc.) and controls.

Extra Information

The C-allele of rs1937 in TFAM (Mitochondrial Transcription Factor A) is linked to endurance. Ahmetov, I.I.; et al. (2009) "The combined impact of metabolic gene polymorphisms on elite endurance athlete status..." and Ahmetov, I.I.; et al. (2010) "Association of mitochondrial transcription factor (TFAM) gene polymorphism with physical performance in athletes" reported positive associations, suggesting a role in mitochondrial biogenesis. Maruszak, A.; et al. (2014) "Mitochondrial DNA variation is associated with elite athletic status in the Polish population" found conflicting results. The reference allele is G.

Endurance

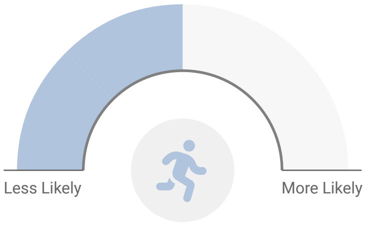

Your G/G genotype does not indicate distinct effects on endurance in context with the UCP3 gene.

• **Gene:** UCP3 • **rsID:** rs1800849 • **Your Genotype:** G/G • **Zygosity:** homo_ref

Description

An individuals endurance capacity is determined by many factors, including their muscle fibre typology, haemoglobin mass, mitochondrial biogenesis, maximal cardiac output, and maximal rate of oxygen consumption (VO2max), among others [118,119,120,121,122,123,124]. Indeed, there is evidence that these intermediate phenotypes have a substantial genetic influence, with literature indicating that genetic factors account for up to 70% of the variability in endurance-related traits [125]. Usually, genetic markers associated with endurance athlete status are determined by comparing allelic frequencies between endurance athletes (e.g., biathletes, road cyclists etc.) and controls.

Extra Information

The T-allele of rs1800849 in the UCP3 gene is linked to endurance. Ahmetov, I.I.; et al. (2008) "The use of molecular genetic methods for prognosis of aerobic and anaerobic performance in athletes" and Ahmetov, I.I.; et al. (2009) "The combined impact of metabolic gene polymorphisms on elite endurance athlete status and related phenotypes" reported positive associations. However, Hudson, D.E.; et al. (2004) "The -55 C/T polymorphism within the UCP3 gene and performance during the South African Ironman Triathlon" found no clear association, indicating controversy. The reference allele is G (alternatives A,C,T).

Exercise Response

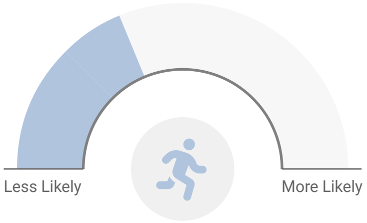

The A/G genotype suggests a possible less increase in individual anaerobic threshold and decreased aerobic fitness, but don't worry; exercise might still bring great benefits!

• **Gene:** PPARD • **rsID:** rs2267668 • **Your Genotype:** G/G>G/A • **Zygosity:** Heterozygous

Description

The body's response to exercise involves coordination among various systems, including musculoskeletal, cardiovascular, respiratory, endocrine, immune, and others. Genetic variants have the potential to influence this exercise response. For instance, certain variants may be associated with a lower VO2 max, a higher body fat percentage, or a more pronounced decrease in blood pressure following exercise.

Extra Information

The rs2267668 AA genotype has been associated with average exercise benefits in studies (PMID(s): 17327385, 18252792). The minor allele 'G' is independently linked to a less increase in individual anaerobic threshold (P = 0.002),and also decreased aerobic physical fitness. This allele (G) is also significantly associated with dynamic balance performance (P = 0.015, Pcorrected < 0.05). Moreover, young carriers of the 'G' allele exhibit low skeletal muscle mitochondrial function (P = 0.02). Furthermore, the GA genotype/polymorphism of rs2267668 is connected to polycystic ovary syndrome (PCOS) (OR = 1.24, P = 0.008).

Ligament Injury Susceptibility

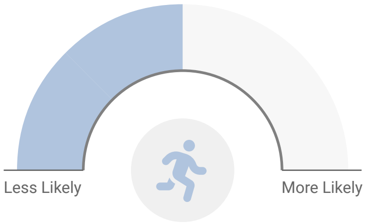

No notable effects of the C/C genotype on ligament injury risk have been observed.

• **Gene:** COL1A1 • **rsID:** rs1800012 • **Your Genotype:** C/C • **Zygosity:** homo_ref

Description

A ligament is a band of tissue that connects bone to bone. Ligament injuries, often involving the knee, are common. The knee has several ligaments, each serving a specific function. The Medial Collateral Ligament (MCL) runs along the inside of the knee, preventing it from bending inward, while the Lateral Collateral Ligament (LCL) runs along the outside, preventing excessive outward bending. The Anterior Cruciate Ligament (ACL), located in the middle, helps prevent the shin bone from sliding forward. The Posterior Cruciate Ligament (PCL) complements the ACL by preventing the shin bone from sliding backward under the thigh bone. Injuries to these ligaments can range from stretching to partial or complete tears, leading to symptoms such as pain, swelling, and a sensation of the knee ""giving out"" when pressure is applied.

Extra Information

Research involving individuals of European ancestry suggests that the rs1800012 GG genotype is associated with an average likelihood of tendon and/or ligament injuries, and rs1800012 GT/TT polymorphism may be associated with the reduced risk of sports-related tendon or ligament injuries(PMID: 28206959).rs1800012 represents a common (allele frequency ~20%) polymorphism consisting of a G-to-T substitution at the first base of a consensus site for the transcription factor Sp1 in the first intron of the COL1A1 gene. COL1A1 rs1800012 polymorphism (G-to-T) may be associated with the reduced risk of sports-related tendon or ligament injuries, especially in ACL injuries, and that rare TT may played as a protective role.The rs1800012(T) allele has been extensively studied, with most observations supporting at least some association with decreased bone mineral density, and increased risk for osteoporosis, fractures, and intervertebral disc disease.

Lumbar Disc Disease Susceptibility

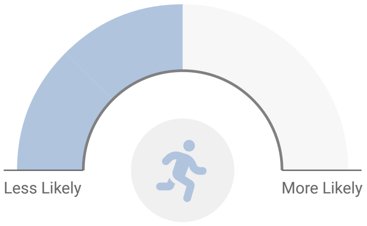

The G/G genotype doesn't seem to be linked with lumbar disc susceptibility changes.

• **Gene:** CILP • **rsID:** rs2073711 • **Your Genotype:** A/A>G/G • **Zygosity:** Homozygous

Description

Lumbar disc disease is a term commonly used to encompass various causes of back pain or sciatica. While muscle strain or ligament sprain can contribute to low back pain (LBP), the intervertebral disc is often implicated more frequently as a source of discomfort. However, it's essential to note that no single lumbar disc injury has been definitively identified as the sole cause of pain associated with lumbar disc disease.

Extra Information

In summary, the rs2073711 CC genotype has been associated with a typical risk of intervertebral disc degeneration (IVD), while CT and TT are representing lower risk of Lumbar Disk Disease.The rs2073711 SNP (1184T_C) is located in exon 8 of the cartilage intermediate layer protein gene (CILP). However, CILP gene is primarily known to be expressed abundantly in intervertebral discs, and was reported to have increased expression when degeneration occurs [PMID 15864306]. For the rs2073711 SNP, the ancestral allele is T and the disease associated risk allele is C. A study of Asian patients with lumbar disc disease (LDD) implicates each copy of a (C) allele of SNP rs2073711 (as oriented with respect to dbSNP) as increasing risk about 1.6 fold. [PMID 15864306].

Muscle Strength

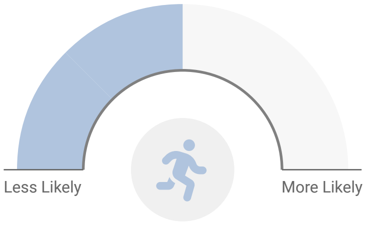

This genotype isn't well-studied in the context of Muscle Strength for this variant.

• **Gene:** CNTF • **rsID:** rs1800169 • **Your Genotype:** G/G>G/A • **Zygosity:** Heterozygous

Description

Muscle strength is defined as the maximum amount of force that a group of muscles (such as biceps, triceps, quadriceps, etc.) can apply to an object simultaneously. The growth and development of muscles are influenced by specific genetic variants, leading to variations in muscle strength among individuals. Additionally, environmental factors, including physical activity and the type of exercise training, play a significant role in determining muscle strength.

Extra Information

The rs1800169 GG genotype has been associated with typical muscle strength and grip strength in women, as reported in studies (PMID(s): 16696750, 17539378, 19628720).CNTF 1357 G _ A (rs1800169), which is GA or AA genotype is associated with muscle strength/power phenotypes in women. The associations in men still need furture investigation.

Muscle Volume

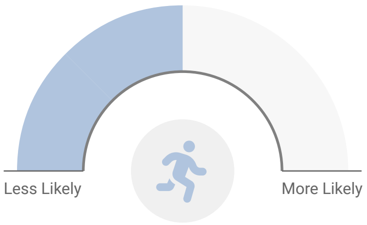

We don't have strong data linking the G/T genotype to changes in Muscle Volume here.

• **Gene:** IL15RA • **rsID:** rs2228059 • **Your Genotype:** T/T>T/G • **Zygosity:** Heterozygous

Description

Muscle volume refers to the overall size of one's muscles, particularly skeletal muscles responsible for movement. These muscles are composed of bundles of muscle fibers within individual muscle cells. The augmentation of muscle volume is typically attributed to the enlargement of muscle cell size rather than an increase in the number of cells. Environmental factors, such as exercise, can impact muscle volume, and genetic factors play a role in determining both one's inherent muscle volume and how effectively muscles respond to training.

Extra Information

The rs2228059 CC genotype has been associated with typical muscle volume and typical baseline cortical bone volume in males, according to a study (PMID: 18514540).The A-allele (CA,AA genotype) in rs2228059 was also reported associated with larger muscle volume but lower muscle quality in men (Pistilli et al., 2008).

Muscular Endurance

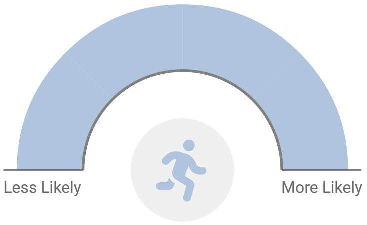

Your G/G genotype in the PPARA gene is an excellent match for endurance athletes, potentially enhancing your long-distance capabilities.

• **Gene:** PPARA • **rsID:** rs4253778 • **Your Genotype:** G/G • **Zygosity:** homo_ref

Description

Muscular endurance refers to the ability of muscles to exert strength over an extended period at a submaximal (less than the maximum) capacity. Sports that demand endurance often involve activities such as cycling, swimming, long-distance running, triathlons, and cross-country skiing.

Extra Information

The rs4253778 GG genotype and G allele has been associated with increased performance in endurance sports, as reported in a study (PMID: 26985127)*, compared to CC genotype.Note:Further studies recruiting athletes that represented different sports disciplines have revealed that it was more likely to find C allele carriers in a group of power-oriented athletes who were involved in short and very intense anaerobic effort (Ahmetov et al., 2006), while GG homozygotes were more prevalent among endurance-type athletes performing predominantly prolonged aerobic exertion (Eynon et al., 2010; Maciejewska et al., 2011). Gineviciene et al. (2010) confirmed the results of previous studies in Lithuanian male athletes showing that those with allele PPARA rs4253778 C had significantly higher muscle mass and better results in explosive strength of lower extremities than GG homozygotes (Ginevi_ien_ et al., 2010). Those results were in part explained by the analysis of muscle fiber composition of young men. It was shown that GG homozygotes had a higher percentage of slow-twitch fibers compared to CC homozygotes and the C allele was associated with the propensity to skeletal muscle hypertrophy (Ahmetov et al., 2006).

Power

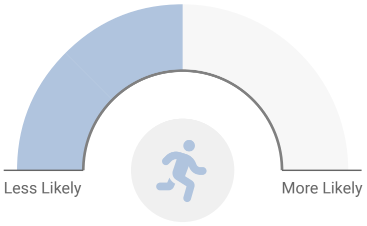

For your Power trait with the IGF2 gene (rs680), the C/C genotype doesn't seem to impact your power capabilities significantly.

• **Gene:** IGF2 • **rsID:** rs680 • **Your Genotype:** T/T>C/C • **Zygosity:** Homozygous

Description

Several characteristics are positively associated with power performance, including circulating levels of testosterone, percentage and cross-sectional area of fast-twitch muscle fibres, muscle mass and strength, body and calcaneus height, muscle fascicle length, and reaction time, among others [3,238,239,240,241,242,243,244]. The heritability of power-related phenotypes has been reported in the literature to range from approximately 49 to 86% in a range of phenotypes, including jumping ability [245,246]. Typically, genetic markers associated with power athlete status are determined by comparing allelic frequencies between power athletes (e.g., 100 m runners, shot putters, arm wrestlers, etc.) and untrained subjects.

Extra Information

The G-allele of rs680 in IGF2 (Insulin Like Growth Factor 2) is linked to power. Pickering, C.; et al. (2019) "A genome-wide association study of sprint performance in elite youth football players," Ben-Zaken, S.; et al. (2022) "The prevalence of IGF-I axis genetic polymorphisms among decathlon athletes," and Itaka, T.; et al. (2016) "G Allele of the IGF2 ApaI Polymorphism Is Associated with Judo Status" reported positive associations. IGF2 is crucial for growth and development. The reference allele is T (alternatives A,C,G).

Power

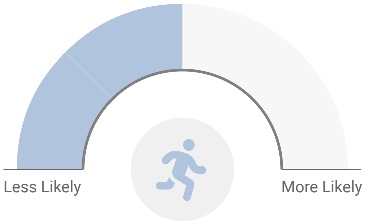

There's no strong evidence linking A/G in ACVR1B (rs2854464) to changes in power ability.

• **Gene:** ACVR1B • **rsID:** rs2854464 • **Your Genotype:** A/A>A/G • **Zygosity:** Heterozygous

Description

Several characteristics are positively associated with power performance, including circulating levels of testosterone, percentage and cross-sectional area of fast-twitch muscle fibres, muscle mass and strength, body and calcaneus height, muscle fascicle length, and reaction time, among others [3,238,239,240,241,242,243,244]. The heritability of power-related phenotypes has been reported in the literature to range from approximately 49 to 86% in a range of phenotypes, including jumping ability [245,246]. Typically, genetic markers associated with power athlete status are determined by comparing allelic frequencies between power athletes (e.g., 100 m runners, shot putters, arm wrestlers, etc.) and untrained subjects.

Extra Information

The A-allele of rs2854464 in ACVR1B (Activin A Receptor Type 1B, muscle growth regulator) is linked to power. Windelinckx, A.; et al. (2011) "...activin receptor 1B (ACVR1B) as a muscle strength gene" and Voisin, S.; et al. (2016) "ACVR1B rs2854464 Is Associated with Sprint/Power Athletic Status in a Large Cohort of Europeans but Not Brazilians" (positive in Europeans) support this. However, Voisin et al. (2016) also noted no association in Brazilians, and Venckunas, T.; Degens, H. (2022) "Genetic polymorphisms of muscular fitness in young healthy men" presented conflicting results, indicating population specificity or complex interactions. The reference allele is A (alternatives C,G).

Power

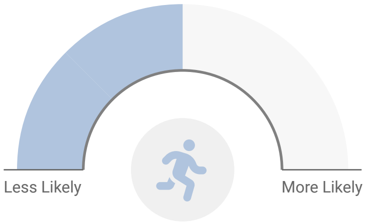

No significant influence on power is seen with an A/G genotype at this locus.

• **Gene:** NRXN3 • **rsID:** rs8011870 • **Your Genotype:** G/G>G/A • **Zygosity:** Heterozygous

Description

Several characteristics are positively associated with power performance, including circulating levels of testosterone, percentage and cross-sectional area of fast-twitch muscle fibres, muscle mass and strength, body and calcaneus height, muscle fascicle length, and reaction time, among others [3,238,239,240,241,242,243,244]. The heritability of power-related phenotypes has been reported in the literature to range from approximately 49 to 86% in a range of phenotypes, including jumping ability [245,246]. Typically, genetic markers associated with power athlete status are determined by comparing allelic frequencies between power athletes (e.g., 100 m runners, shot putters, arm wrestlers, etc.) and untrained subjects.

Extra Information

The G-allele of rs8011870 in NRXN3 (synaptic function) is linked to power. Guilherme, J.P.L.F.; et al. (2022) "Genomic Predictors of Brisk Walking Are Associated with Elite Sprinter Status" identified this association, suggesting a role for neuronal pathways in explosive performance. The reference allele is G (alternatives A,T).

Name:
DoB:
Gender:
Report ID: WBWG_01_P001_262
Patient ID: 01_P001_262
Date: 6/7/2025

Power

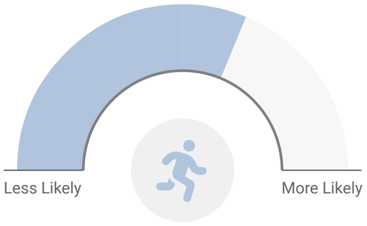

With the A/A genotype in your Power trait's CRTC1 gene, you might notice enhancements in explosive strength or speed.

• **Gene:** CRTC1 • **rsID:** rs11881338 • **Your Genotype:** T/T>A/A • **Zygosity:** Homozygous

Description

Several characteristics are positively associated with power performance, including circulating levels of testosterone, percentage and cross-sectional area of fast-twitch muscle fibres, muscle mass and strength, body and calcaneus height, muscle fascicle length, and reaction time, among others [3,238,239,240,241,242,243,244]. The heritability of power-related phenotypes has been reported in the literature to range from approximately 49 to 86% in a range of phenotypes, including jumping ability [245,246]. Typically, genetic markers associated with power athlete status are determined by comparing allelic frequencies between power athletes (e.g., 100 m runners, shot putters, arm wrestlers, etc.) and untrained subjects.

Extra Information

The A-allele of rs11881338 in CRTC1 (CREB Regulated Transcription Coactivator 1) is linked to power. Guilherme, J.P.L.F.; et al. (2022) "Genomic Predictors of Brisk Walking Are Associated with Elite Sprinter Status" identified this. CRTC1 is involved in regulating gene expression in response to cAMP signaling. The reference allele is T (alternatives A,C,G).

Power

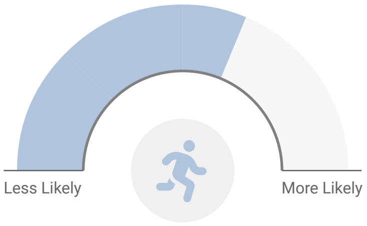

Possessing the A/G genotype in your ADAM15 gene might give you some advantages in power activities.

• **Gene:** ADAM15 • **rsID:** rs11264302 • **Your Genotype:** G/G>G/A • **Zygosity:** Heterozygous

Description

Several characteristics are positively associated with power performance, including circulating levels of testosterone, percentage and cross-sectional area of fast-twitch muscle fibres, muscle mass and strength, body and calcaneus height, muscle fascicle length, and reaction time, among others [3,238,239,240,241,242,243,244]. The heritability of power-related phenotypes has been reported in the literature to range from approximately 49 to 86% in a range of phenotypes, including jumping ability [245,246]. Typically, genetic markers associated with power athlete status are determined by comparing allelic frequencies between power athletes (e.g., 100 m runners, shot putters, arm wrestlers, etc.) and untrained subjects.

Extra Information

The G-allele of rs11264302 in ADAM15 (A Disintegrin And Metalloproteinase Domain 15) is linked to power. Guilherme, J.P.L.F.; et al. (2022) "Genomic Predictors of Brisk Walking Are Associated with Elite Sprinter Status" reported this association. ADAM15 is involved in cell adhesion and signaling, potentially impacting muscle cell interactions or repair. The reference allele is G (alternative A).

Power

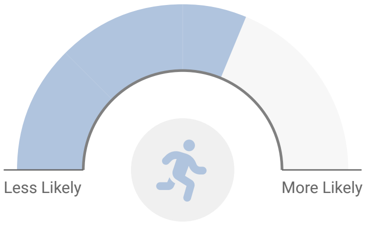

With your A/C genotype here, you might enjoy a moderate boost in power-related activities.

• **Gene:** NUP210 • **rsID:** rs2280084 • **Your Genotype:** C/C>C/A • **Zygosity:** Heterozygous

Description

Several characteristics are positively associated with power performance, including circulating levels of testosterone, percentage and cross-sectional area of fast-twitch muscle fibres, muscle mass and strength, body and calcaneus height, muscle fascicle length, and reaction time, among others [3,238,239,240,241,242,243,244]. The heritability of power-related phenotypes has been reported in the literature to range from approximately 49 to 86% in a range of phenotypes, including jumping ability [245,246]. Typically, genetic markers associated with power athlete status are determined by comparing allelic frequencies between power athletes (e.g., 100 m runners, shot putters, arm wrestlers, etc.) and untrained subjects.

Extra Information

The C-allele of rs2280084 in NUP210 (Nucleoporin 210) is linked to power. Bulgay, C.; et al. (2023) "Exome-Wide Association Study of Competitive Performance in Elite Athletes" reported this association, suggesting NUP210, a component of the nuclear pore complex, might influence cellular processes relevant to athletic performance. The reference allele is C (alternatives A,G,T).

Power

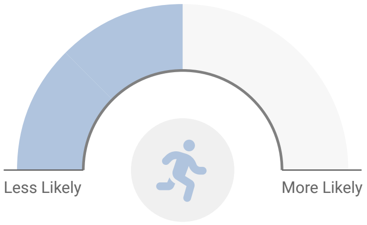

The A/A genotype in your ADRB2 gene doesn't show a strong correlation with power performance.

• **Gene:** ADRB2 • **rsID:** rs1042713 • **Your Genotype:** G/G>A/A • **Zygosity:** Homozygous

Description

Several characteristics are positively associated with power performance, including circulating levels of testosterone, percentage and cross-sectional area of fast-twitch muscle fibres, muscle mass and strength, body and calcaneus height, muscle fascicle length, and reaction time, among others [3,238,239,240,241,242,243,244]. The heritability of power-related phenotypes has been reported in the literature to range from approximately 49 to 86% in a range of phenotypes, including jumping ability [245,246]. Typically, genetic markers associated with power athlete status are determined by comparing allelic frequencies between power athletes (e.g., 100 m runners, shot putters, arm wrestlers, etc.) and untrained subjects.

Extra Information

The G-allele (Gly16) of rs1042713 (Arg16Gly) in ADRB2 (Beta-2 Adrenergic Receptor) is linked to power. Pickering, C.; et al. (2019) "A genome-wide association study of sprint performance in elite youth football players" and Sawczuk, M.; et al. (2013) "Association of the ADRB2 Gly16Arg and Glu27Gln polymorphisms with athlete status" reported associations, possibly via modulation of adrenergic signaling affecting muscle contractility or metabolism. The reference allele is G (alternatives A,C).

SZA Longevity
A healthy future awaits you

Name:
DoB:
Gender:
Report ID: WBWG_01_P001_262
Patient ID: 01_P001_262
Date: 6/7/2025

Power

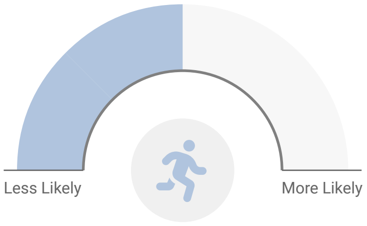

C/C doesn't seem to provide any clear advantage in power attributes based on current data.

• **Gene:** ADRB2 • **rsID:** rs1042714 • **Your Genotype:** G/G>C/C • **Zygosity:** Homozygous

Description
Several characteristics are positively associated with power performance, including circulating levels of testosterone, percentage and cross-sectional area of fast-twitch muscle fibres, muscle mass and strength, body and calcaneus height, muscle fascicle length, and reaction time, among others [3,238,239,240,241,242,243,244]. The heritability of power-related phenotypes has been reported in the literature to range from approximately 49 to 86% in a range of phenotypes, including jumping ability [245,246]. Typically, genetic markers associated with power athlete status are determined by comparing allelic frequencies between power athletes (e.g., 100 m runners, shot putters, arm wrestlers, etc.) and untrained subjects.

Extra Information
The G-allele (Gln27) of rs1042714 (Glu27Gln) in ADRB2 (Beta-2 Adrenergic Receptor) is linked to power. Pickering, C.; et al. (2019) "A genome-wide association study of sprint performance in elite youth football players" and Sawczuk, M.; et al. (2013) "Association of the ADRB2 Gly16Arg and Glu27Gln polymorphisms with athlete status" reported associations. This variant may influence adrenergic signaling affecting muscle function. The reference allele is G (alternatives A,C,T).

Power

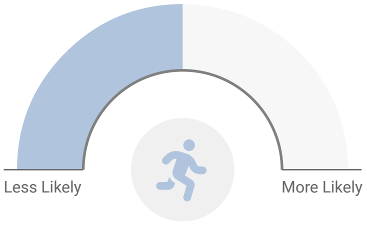

Your A/G genotype isn't linked to notable power performance variations.

• **Gene:** SOD2 • **rsID:** rs4880 • **Your Genotype:** A/A>A/G • **Zygosity:** Heterozygous

Description
Several characteristics are positively associated with power performance, including circulating levels of testosterone, percentage and cross-sectional area of fast-twitch muscle fibres, muscle mass and strength, body and calcaneus height, muscle fascicle length, and reaction time, among others [3,238,239,240,241,242,243,244]. The heritability of power-related phenotypes has been reported in the literature to range from approximately 49 to 86% in a range of phenotypes, including jumping ability [245,246]. Typically, genetic markers associated with power athlete status are determined by comparing allelic frequencies between power athletes (e.g., 100 m runners, shot putters, arm wrestlers, etc.) and untrained subjects.

Extra Information
The C-allele (Val) of rs4880 (Ala16Val) in SOD2 (Superoxide Dismutase 2, mitochondrial antioxidant) is linked to power. Ahmetov, I.I.; et al. (2014) "SOD2 gene polymorphism and muscle damage markers in elite athletes" reported that the CC (Val/Val) genotype was associated with lower levels of muscle damage markers in power athletes, suggesting better protection against oxidative stress. The reference allele is A (Ala).

Power

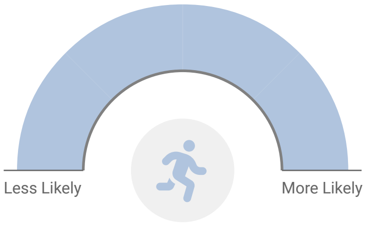

Excitingly, T/T in the NOS3 gene could enhance your performance in explosive power activities.

• **Gene:** NOS3 • **rsID:** rs2070744 • **Your Genotype:** C/C>T/T • **Zygosity:** Homozygous

Description
Several characteristics are positively associated with power performance, including circulating levels of testosterone, percentage and cross-sectional area of fast-twitch muscle fibres, muscle mass and strength, body and calcaneus height, muscle fascicle length, and reaction time, among others [3,238,239,240,241,242,243,244]. The heritability of power-related phenotypes has been reported in the literature to range from approximately 49 to 86% in a range of phenotypes, including jumping ability [245,246]. Typically, genetic markers associated with power athlete status are determined by comparing allelic frequencies between power athletes (e.g., 100 m runners, shot putters, arm wrestlers, etc.) and untrained subjects.

Extra Information
The T-allele of rs2070744 (T-786C) in the NOS3 promoter (nitric oxide production) is linked to power. Gómez-Gallego, F.; et al. (2009) "The -786 T/C polymorphism of the NOS3 gene is associated with elite performance in power sports," Drozdovska, S.B.; et al. (2013) "The association of gene polymorphisms with athlete status in ukrainians," and Murtagh, C.F.; et al. (2020) "The genetic profile of elite youth soccer players..." linked the T-allele to power athletes, possibly via NO's influence on blood flow or mitochondrial biogenesis. The reference allele is C (alternatives G,T).

Power

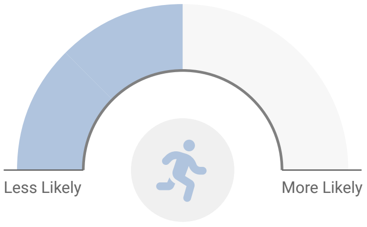

With the G/G genotype in your ACE gene (rs4343), you might have a slight edge in power-related activities.

• **Gene:** ACE • **rsID:** rs4343 • **Your Genotype:** G/G • **Zygosity:** homo_ref

Description
Several characteristics are positively associated with power performance, including circulating levels of testosterone, percentage and cross-sectional area of fast-twitch muscle fibres, muscle mass and strength, body and calcaneus height, muscle fascicle length, and reaction time, among others [3,238,239,240,241,242,243,244]. The heritability of power-related phenotypes has been reported in the literature to range from approximately 49 to 86% in a range of phenotypes, including jumping ability [245,246]. Typically, genetic markers associated with power athlete status are determined by comparing allelic frequencies between power athletes (e.g., 100 m runners, shot putters, arm wrestlers, etc.) and untrained subjects.

Extra Information
The G-allele of rs4343 in ACE is linked to power (often associated with D-allele of ACE I/D). Nazarov, I.B.; et al. (2001) "The angiotensin converting enzyme I/D polymorphism in Russian athletes," Myerson, S.; et al. (1999) "Human angiotensin I-converting enzyme gene and endurance performance," Wang, G.; et al. (2013) "Association analysis of ACE and ACTN3 in elite Caucasian and East Asian swimmers," Woods, D.; et al. (2001) "Elite swimmers and the D allele of the ACE I/D polymorphism," Costa, A.M.; et al. (2009) "Association between ACE D allele and elite short distance swimming," Boraita, A.; et al. (2010) "Cardiovascular adaptation...and Angiotensin-converting enzyme I/D polymorphism in elite athletes," and Papadimitriou, I.D.; et al. (2016) "ACTN3 R577X and ACE I/D gene variants influence performance in elite sprinters..." (Ref 145 was empty) support this. Conflicting results exist from studies like Ginevičienė, V.; et

al. (2011), Amir, O.; et al. (2007), Kim, C.H.; et al. (2010), Shahmoradi, S.; et al. (2014), and Scott, R.A.; et al.

(2010). The reference allele is G.

Name: Report ID: WBWG_01_P001_262
DoB: Patient ID: 01_P001_262
Gender: Date: 6/7/2025

Power

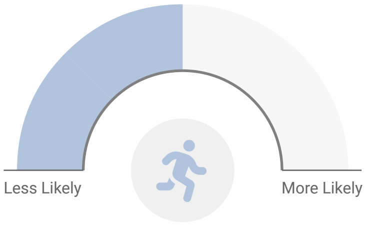

For your power trait with the ACE gene (rs4341), having the G/G genotype might give you a minor boost.

• **Gene:** ACE • **rsID:** rs4341 • **Your Genotype:** G/G • **Zygosity:** homo_ref

Description

Several characteristics are positively associated with power performance, including circulating levels of testosterone, percentage and cross-sectional area of fast-twitch muscle fibres, muscle mass and strength, body and calcaneus height, muscle fascicle length, and reaction time, among others [3,238,239,240,241,242,243,244]. The heritability of power-related phenotypes has been reported in the literature to range from approximately 49 to 86% in a range of phenotypes, including jumping ability [245,246]. Typically, genetic markers associated with power athlete status are determined by comparing allelic frequencies between power athletes (e.g., 100 m runners, shot putters, arm wrestlers, etc.) and untrained subjects.

Extra Information

The G-allele of rs4341 in ACE (often linked to D-allele of ACE I/D) is associated with power. Numerous studies including Nazarov, I.B.; et al. (2001) "The angiotensin converting enzyme I/D polymorphism in Russian athletes," Myerson, S.; et al. (1999) "Human angiotensin I-converting enzyme gene and endurance performance," Wang, G.; et al. (2013) "Association analysis of ACE and ACTN3 in elite Caucasian and East Asian swimmers," Woods, D.; et al. (2001) "Elite swimmers and the D allele of the ACE I/D polymorphism," Costa, A.M.; et al. (2009) "Association between ACE D allele and elite short distance swimming," Boraita, A.; et al. (2010) "Cardiovascular adaptation...and Angiotensin-converting enzyme I/D polymorphism in elite athletes," and Papadimitriou, I.D.; et al. (2016) "ACTN3 R577X and ACE I/D gene variants influence performance in elite sprinters..." support this. (Ref 145 was empty). However, Ginevičienė, V.; et al. (2011) "Genetic variation of the human ACE and ACTN3 genes...," Amir, O.; et al. (2007) "The ACE deletion allele is associated with Israeli elite endurance athletes," Kim, C.H.; et al. (2010) "ACE DD genotype is unfavorable to Korean short-term muscle power athletes," Shahmoradi, S.; et al. (2014) "Evaluation of ACE gene I/D polymorphism in Iranian elite athletes," and Scott, R.A.; et al. (2010) "ACTN3 and ACE genotypes in elite Jamaican and US sprinters" show conflicting results. The reference allele is G.

Power

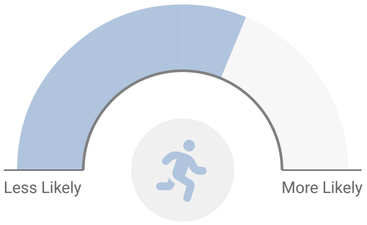

Your C/C genotype in AGRN may help enhance your power performance, as C is a favorable allele.

• **Gene:** AGRN • **rsID:** rs4074992 • **Your Genotype:** C/C • **Zygosity:** homo_ref

Description

Several characteristics are positively associated with power performance, including circulating levels of testosterone, percentage and cross-sectional area of fast-twitch muscle fibres, muscle mass and strength, body and calcaneus height, muscle fascicle length, and reaction time, among others [3,238,239,240,241,242,243,244]. The heritability of power-related phenotypes has been reported in the literature to range from approximately 49 to 86% in a range of phenotypes, including jumping ability [245,246]. Typically, genetic markers associated with power athlete status are determined by comparing allelic frequencies between power athletes (e.g., 100 m runners, shot putters, arm wrestlers, etc.) and untrained subjects.

Extra Information

The C-allele of rs4074992 in AGRN (neuromuscular junction maintenance) is linked to power. Bulgay, C.; et al. (2023) "Exome-Wide Association Study of Competitive Performance in Elite Athletes" identified this association, suggesting efficient NMJ function, critical for forceful muscle contractions, is influenced by this variant. The reference allele is C (alternatives A,T).

Power

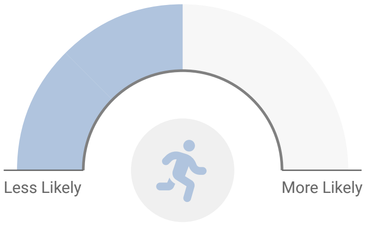

For your Power trait with the AGT gene (rs699), having A/A doesn't appear to provide notable effects.

• **Gene:** AGT • **rsID:** rs699 • **Your Genotype:** A/A • **Zygosity:** homo_ref

Description

Several characteristics are positively associated with power performance, including circulating levels of testosterone, percentage and cross-sectional area of fast-twitch muscle fibres, muscle mass and strength, body and calcaneus height, muscle fascicle length, and reaction time, among others [3,238,239,240,241,242,243,244]. The heritability of power-related phenotypes has been reported in the literature to range from approximately 49 to 86% in a range of phenotypes, including jumping ability [245,246]. Typically, genetic markers associated with power athlete status are determined by comparing allelic frequencies between power athletes (e.g., 100 m runners, shot putters, arm wrestlers, etc.) and untrained subjects.

Extra Information

The C-allele (T235) of rs699 (M235T) in AGT is linked to power. Pickering, C.; et al. (2019) "A genome-wide association study of sprint performance in elite youth football players," Gomez-Gallego, F.; et al. (2009) "The C allele of the AGT Met235Thr polymorphism is associated with power sports performance," and Zarębska, A.; et al. (2013) "Association of rs699 (M235T) polymorphism in the AGT gene with power but not endurance athlete status" suggest a link with power performance. The reference allele is A (M235).

Power

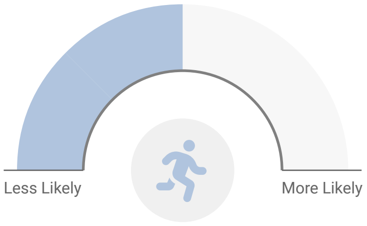

For your Power trait with the AGTR2 gene (rs11091046), having the A/A genotype might offer moderate benefits in power activities.

- **Gene:** AGTR2
- **rsID:** rs11091046
- **Your Genotype:** A/A
- **Zygosity:** homo_ref

Description

Several characteristics are positively associated with power performance, including circulating levels of testosterone, percentage and cross-sectional area of fast-twitch muscle fibres, muscle mass and strength, body and calcaneus height, muscle fascicle length, and reaction time, among others [3,238,239,240,241,242,243,244]. The heritability of power-related phenotypes has been reported in the literature to range from approximately 49 to 86% in a range of phenotypes, including jumping ability [245,246]. Typically, genetic markers associated with power athlete status are determined by comparing allelic frequencies between power athletes (e.g., 100 m runners, shot putters, arm wrestlers, etc.) and untrained subjects.

Extra Information

The A-allele of rs11091046 in AGTR2 (X chromosome) is linked to power. Mustafina, L.J.; et al. (2014) "AGTR2 gene polymorphism is associated with muscle fibre composition, athletic status and aerobic performance" and Guilherme, J.P.L.F.; et al. (2018) "The AGTR2 rs11091046 (A>C) polymorphism and power athletic status in top-level Brazilian athletes" reported associations, particularly in females. Yvert, T.P.; et al. (2018) "AGTR2 and sprint/power performance: A case-control replication study..." had controversial results. The reference allele is A (alternatives C,T).

Power

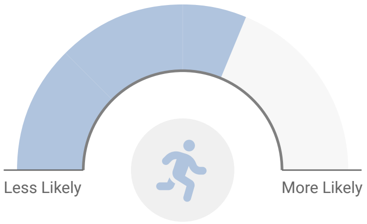

With the C/C genotype in AKAP6, you might see enhanced power-related performance.

- **Gene:** AKAP6
- **rsID:** rs12883788
- **Your Genotype:** C/C
- **Zygosity:** homo_ref

Description

Several characteristics are positively associated with power performance, including circulating levels of testosterone, percentage and cross-sectional area of fast-twitch muscle fibres, muscle mass and strength, body and calcaneus height, muscle fascicle length, and reaction time, among others [3,238,239,240,241,242,243,244]. The heritability of power-related phenotypes has been reported in the literature to range from approximately 49 to 86% in a range of phenotypes, including jumping ability [245,246]. Typically, genetic markers associated with power athlete status are determined by comparing allelic frequencies between power athletes (e.g., 100 m runners, shot putters, arm wrestlers, etc.) and untrained subjects.

Extra Information

The C-allele of rs12883788 in AKAP6 (A-Kinase Anchoring Protein 6) is linked to power. Guilherme, J.P.L.F.; et al. (2022) "Genomic Predictors of Brisk Walking Are Associated with Elite Sprinter Status" identified this association. AKAP6 is involved in organizing signaling complexes, which could influence muscle cell responses. The reference allele is C (alternatives A,T).

Power

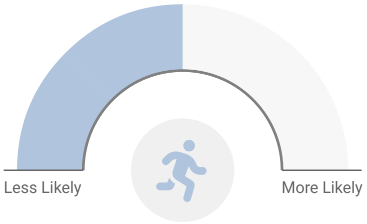

We see no strong evidence of G/G affecting your power traits.

- **Gene:** AMPD1
- **rsID:** rs17602729
- **Your Genotype:** G/G
- **Zygosity:** homo_ref

Description

Several characteristics are positively associated with power performance, including circulating levels of testosterone, percentage and cross-sectional area of fast-twitch muscle fibres, muscle mass and strength, body and calcaneus height, muscle fascicle length, and reaction time, among others [3,238,239,240,241,242,243,244]. The heritability of power-related phenotypes has been reported in the literature to range from approximately 49 to 86% in a range of phenotypes, including jumping ability [245,246]. Typically, genetic markers associated with power athlete status are determined by comparing allelic frequencies between power athletes (e.g., 100 m runners, shot putters, arm wrestlers, etc.) and untrained subjects.

Extra Information

The C-allele of rs17602729 (C34T) in AMPD1 (Adenosine Monophosphate Deaminase 1) is linked to power. Ginevičienė, V.; et al. (2014) "AMPD1 rs17602729 is associated with physical performance of sprint and power in elite Lithuanian athletes," Cies´zczyk, P.; et al. (2012) "Distribution of the AMPD1 C34T polymorphism in Polish power-oriented athletes," and Fedotovskaya, O.N.; et al. (2013) "Effect of AMPD1 gene polymorphism on muscle activity in humans" reported positive associations. The C-allele (normal enzyme activity) is thought to prevent exercise intolerance sometimes seen with the T-allele. The reference allele is G.

Power

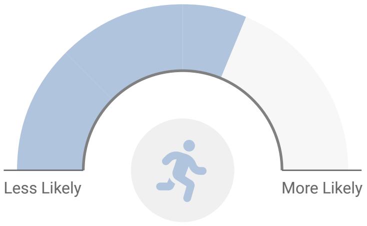

With the A/A genotype in the AUTS2 gene (rs10452738), you might exhibit traits that align with power athleticism, like increased fast-twitch muscle fibers.

• **Gene:** AUTS2 • **rsID:** rs10452738 • **Your Genotype:** A/A • **Zygosity:** homo_ref

Description

Several characteristics are positively associated with power performance, including circulating levels of testosterone, percentage and cross-sectional area of fast-twitch muscle fibres, muscle mass and strength, body and calcaneus height, muscle fascicle length, and reaction time, among others [3,238,239,240,241,242,243,244]. The heritability of power-related phenotypes has been reported in the literature to range from approximately 49 to 86% in a range of phenotypes, including jumping ability [245,246]. Typically, genetic markers associated with power athlete status are determined by comparing allelic frequencies between power athletes (e.g., 100 m runners, shot putters, arm wrestlers, etc.) and untrained subjects.

Extra Information

The A-allele of rs10452738 in AUTS2 (Autism Susceptibility Candidate 2) is linked to power. Guilherme, J.P.L.F.; et al. (2022) "Genomic Predictors of Brisk Walking Are Associated with Elite Sprinter Status" identified this association. AUTS2 has roles in neuronal development, which could indirectly influence motor control relevant to power. The reference allele is A (alternative G).

Power

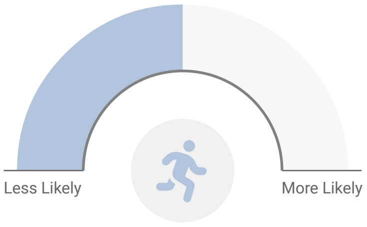

There's not enough evidence to suggest that the C/C genotype impacts power abilities.

• **Gene:** BDNF • **rsID:** rs10501089 • **Your Genotype:** C/C • **Zygosity:** homo_ref

Description

Several characteristics are positively associated with power performance, including circulating levels of testosterone, percentage and cross-sectional area of fast-twitch muscle fibres, muscle mass and strength, body and calcaneus height, muscle fascicle length, and reaction time, among others [3,238,239,240,241,242,243,244]. The heritability of power-related phenotypes has been reported in the literature to range from approximately 49 to 86% in a range of phenotypes, including jumping ability [245,246]. Typically, genetic markers associated with power athlete status are determined by comparing allelic frequencies between power athletes (e.g., 100 m runners, shot putters, arm wrestlers, etc.) and untrained subjects.

Extra Information

The A-allele of rs10501089 in BDNF (Brain-Derived Neurotrophic Factor) is linked to power. Guilherme, J.P.L.F.; et al. (2022) "The BDNF-Increasing Allele is Associated with Increased Proportion of Fast-Twitch Muscle Fibers, Handgrip Strength, and Power Athlete Status" reported associations with increased fast-twitch fibers, handgrip strength, and power athlete status. BDNF is crucial for neuronal health and synaptic plasticity. The reference allele is C (alternatives G,T).

Power

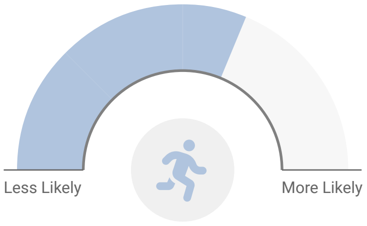

Having a T/T genotype might align you more with traits that favor power performance, such as muscle strength.

• **Gene:** CCT3 • **rsID:** rs11548200 • **Your Genotype:** T/T • **Zygosity:** homo_ref

Description

Several characteristics are positively associated with power performance, including circulating levels of testosterone, percentage and cross-sectional area of fast-twitch muscle fibres, muscle mass and strength, body and calcaneus height, muscle fascicle length, and reaction time, among others [3,238,239,240,241,242,243,244]. The heritability of power-related phenotypes has been reported in the literature to range from approximately 49 to 86% in a range of phenotypes, including jumping ability [245,246]. Typically, genetic markers associated with power athlete status are determined by comparing allelic frequencies between power athletes (e.g., 100 m runners, shot putters, arm wrestlers, etc.) and untrained subjects.

Extra Information

The T-allele of rs11548200 in CCT3 (Chaperonin Containing TCP1 Subunit 3) is linked to power. Guilherme, J.P.L.F.; et al. (2022) "Genomic Predictors of Brisk Walking Are Associated with Elite Sprinter Status" identified this association. CCT3 is part of a chaperonin complex involved in protein folding. The reference allele is T (alternative C).

Power

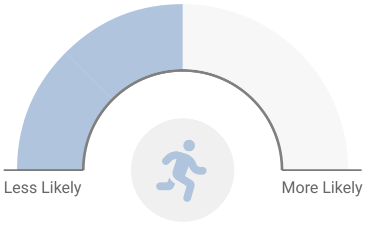

For your Power trait with the CDKN1A gene (rs236448), there's no strong evidence of notable effects with the A/A genotype.

• **Gene:** CDKN1A • **rsID:** rs236448 • **Your Genotype:** A/A • **Zygosity:** homo_ref

Description

Several characteristics are positively associated with power performance, including circulating levels of testosterone, percentage and cross-sectional area of fast-twitch muscle fibres, muscle mass and strength, body and calcaneus height, muscle fascicle length, and reaction time, among others [3,238,239,240,241,242,243,244]. The heritability of power-related phenotypes has been reported in the literature to range from approximately 49 to 86% in a range of phenotypes, including jumping ability [245,246]. Typically, genetic markers associated with power athlete status are determined by comparing allelic frequencies between power athletes (e.g., 100 m runners, shot putters, arm wrestlers, etc.) and untrained subjects.

Extra Information

The C-allele of rs236448 in CDKN1A (cell cycle inhibitor) is linked to power. Semenova, E.A.; et al. (2022) "Genome-Wide Association Study Identifies CDKN1A as a Novel Locus Associated with Muscle Fiber Composition" (which also discusses power implications) found an association, suggesting a role in muscle development or repair relevant to power. The reference allele is A (alternative C).

Name:
DoB:
Gender:
Report ID: WBWG_01_P001_262
Patient ID: 01_P001_262
Date: 6/7/2025

Power

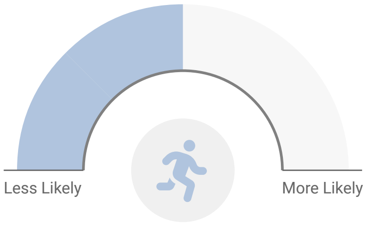

Having the T/T genotype doesn't show relevant effects on power performance.

• **Gene:** CKM • **rsID:** rs8111989 • **Your Genotype:** T/T • **Zygosity:** homo_ref

Description

Several characteristics are positively associated with power performance, including circulating levels of testosterone, percentage and cross-sectional area of fast-twitch muscle fibres, muscle mass and strength, body and calcaneus height, muscle fascicle length, and reaction time, among others [3,238,239,240,241,242,243,244]. The heritability of power-related phenotypes has been reported in the literature to range from approximately 49 to 86% in a range of phenotypes, including jumping ability [245,246]. Typically, genetic markers associated with power athlete status are determined by comparing allelic frequencies between power athletes (e.g., 100 m runners, shot putters, arm wrestlers, etc.) and untrained subjects.

Extra Information

The G-allele of rs8111989 in CKM (muscle creatine kinase) is linked to power. Saito, M.; et al. (2022) "Genetic profile of sports climbing athletes from three different ethnicities" reported a positive association. (Refs 272 and 273 were empty). However, Ginevičienė, V.; et al. (2021) "CKM Gene rs8111989 Polymorphism and Power Athlete Status" presented conflicting results. This suggests the G-allele (ref T, alt C, where C is the G on opposite strand) might optimize rapid ATP regeneration for power.

Power

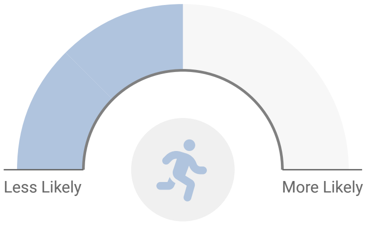

The G/G genotype doesn't exhibit a strong connection with power traits.

• **Gene:** CNTFR • **rsID:** rs41274853 • **Your Genotype:** G/G • **Zygosity:** homo_ref

Description

Several characteristics are positively associated with power performance, including circulating levels of testosterone, percentage and cross-sectional area of fast-twitch muscle fibres, muscle mass and strength, body and calcaneus height, muscle fascicle length, and reaction time, among others [3,238,239,240,241,242,243,244]. The heritability of power-related phenotypes has been reported in the literature to range from approximately 49 to 86% in a range of phenotypes, including jumping ability [245,246]. Typically, genetic markers associated with power athlete status are determined by comparing allelic frequencies between power athletes (e.g., 100 m runners, shot putters, arm wrestlers, etc.) and untrained subjects.

Extra Information

The T-allele of rs41274853 in CNTFR (Ciliary Neurotrophic Factor Receptor) is linked to power. Miyamoto-Mikami, E.; et al. (2016) "CNTFR Genotype and Sprint/power Performance: Case-control Association and Functional Studies" reported an association with sprint/power performance. CNTFR is involved in neuronal survival and muscle function. The reference allele is G (alternatives A,C).

Power

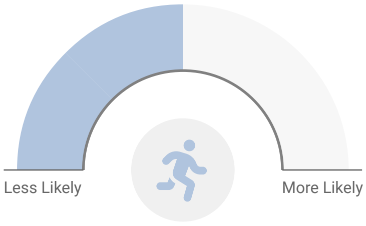

If you have the C/C genotype, we don't see significant effects on your Power trait related to CPNE5.

• **Gene:** CPNE5 • **rsID:** rs3213537 • **Your Genotype:** C/C • **Zygosity:** homo_ref

Description

Several characteristics are positively associated with power performance, including circulating levels of testosterone, percentage and cross-sectional area of fast-twitch muscle fibres, muscle mass and strength, body and calcaneus height, muscle fascicle length, and reaction time, among others [3,238,239,240,241,242,243,244]. The heritability of power-related phenotypes has been reported in the literature to range from approximately 49 to 86% in a range of phenotypes, including jumping ability [245,246]. Typically, genetic markers associated with power athlete status are determined by comparing allelic frequencies between power athletes (e.g., 100 m runners, shot putters, arm wrestlers, etc.) and untrained subjects.

Extra Information

The G-allele of rs3213537 in CPNE5 (Copine 5) is linked to power. Pickering, C.; et al. (2019) "A genome-wide association study of sprint performance in elite youth football players" and Guilherme, J.P.L.F.; et al. (2021) "Are Genome-Wide Association Study Identified Single-Nucleotide Polymorphisms Associated with Sprint Athletic Status? A Replication Study..." reported positive associations. CPNE5's function may relate to calcium-dependent signaling in muscle. The reference allele is C (alternative T).

Power

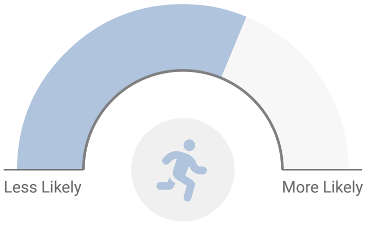

Having the A/A genotype in the CRTAC1 gene can positively be linked to enhanced power traits like muscle strength.

• **Gene:** CRTAC1 • **rsID:** rs2439823 • **Your Genotype:** A/A • **Zygosity:** homo_ref

Description

Several characteristics are positively associated with power performance, including circulating levels of testosterone, percentage and cross-sectional area of fast-twitch muscle fibres, muscle mass and strength, body and calcaneus height, muscle fascicle length, and reaction time, among others [3,238,239,240,241,242,243,244]. The heritability of power-related phenotypes has been reported in the literature to range from approximately 49 to 86% in a range of phenotypes, including jumping ability [245,246]. Typically, genetic markers associated with power athlete status are determined by comparing allelic frequencies between power athletes (e.g., 100 m runners, shot putters, arm wrestlers, etc.) and untrained subjects.

Extra Information

The A-allele of rs2439823 in CRTAC1 is linked to power. Guilherme, J.P.L.F.; et al. (2022) "Genomic Predictors of Brisk Walking Are Associated with Elite Sprinter Status" identified this association. CRTAC1 (Cartilage Acidic Protein 1) may have pleiotropic effects on musculoskeletal traits relevant to power. The reference allele is A (alternative G).

Name:
DoB:
Gender:
Report ID: WBWG_01_P001_262
Patient ID: 01_P001_262
Date: 6/7/2025

Power

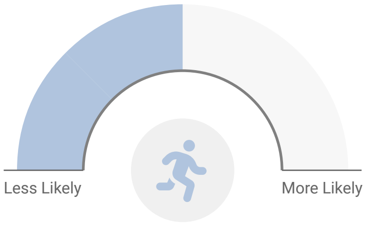

For C/C genotype in the E2F3 gene, there's currently no strong evidence for an effect on power.

• **Gene:** E2F3 • **rsID:** rs4134943 • **Your Genotype:** C/C • **Zygosity:** homo_ref

Description

Several characteristics are positively associated with power performance, including circulating levels of testosterone, percentage and cross-sectional area of fast-twitch muscle fibres, muscle mass and strength, body and calcaneus height, muscle fascicle length, and reaction time, among others [3,238,239,240,241,242,243,244]. The heritability of power-related phenotypes has been reported in the literature to range from approximately 49 to 86% in a range of phenotypes, including jumping ability [245,246]. Typically, genetic markers associated with power athlete status are determined by comparing allelic frequencies between power athletes (e.g., 100 m runners, shot putters, arm wrestlers, etc.) and untrained subjects.

Extra Information

The T-allele of rs4134943 in E2F3 (E2F Transcription Factor 3) is linked to power. Guilherme, J.P.L.F.; et al. (2022) "Genomic Predictors of Brisk Walking Are Associated with Elite Sprinter Status" reported this association. E2F3 is a cell cycle regulator, potentially influencing muscle cell proliferation or differentiation. The reference allele is C (alternatives A,T).

Power

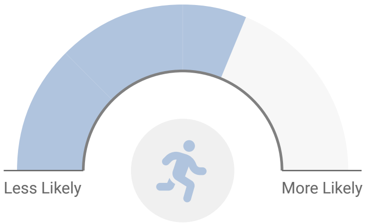

For those with the C/C genotype, you might have a better predisposition for power activities, perhaps benefiting from improved muscle strength.

• **Gene:** FHL2 • **rsID:** rs55680124 • **Your Genotype:** C/C • **Zygosity:** homo_ref

Description

Several characteristics are positively associated with power performance, including circulating levels of testosterone, percentage and cross-sectional area of fast-twitch muscle fibres, muscle mass and strength, body and calcaneus height, muscle fascicle length, and reaction time, among others [3,238,239,240,241,242,243,244]. The heritability of power-related phenotypes has been reported in the literature to range from approximately 49 to 86% in a range of phenotypes, including jumping ability [245,246]. Typically, genetic markers associated with power athlete status are determined by comparing allelic frequencies between power athletes (e.g., 100 m runners, shot putters, arm wrestlers, etc.) and untrained subjects.

Extra Information

The C-allele of rs55680124 in FHL2 (Four And A Half LIM Domains 2) is linked to power. Guilherme, J.P.L.F.; et al. (2022) "Genomic Predictors of Brisk Walking Are Associated with Elite Sprinter Status" reported this association. FHL2 is involved in muscle development and response to mechanical stress. The reference allele is C (alternative T).

Power

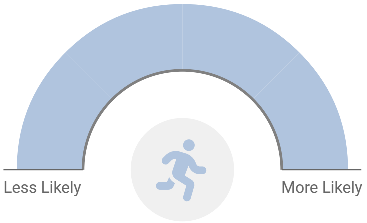

For power traits associated with GDF5, having the G/G genotype could boost your explosive performance!

• **Gene:** GDF5 • **rsID:** rs143384 • **Your Genotype:** G/G • **Zygosity:** homo_ref

Description

Several characteristics are positively associated with power performance, including circulating levels of testosterone, percentage and cross-sectional area of fast-twitch muscle fibres, muscle mass and strength, body and calcaneus height, muscle fascicle length, and reaction time, among others [3,238,239,240,241,242,243,244]. The heritability of power-related phenotypes has been reported in the literature to range from approximately 49 to 86% in a range of phenotypes, including jumping ability [245,246]. Typically, genetic markers associated with power athlete status are determined by comparing allelic frequencies between power athletes (e.g., 100 m runners, shot putters, arm wrestlers, etc.) and untrained subjects.

Extra Information

The G-allele of rs143384 in GDF5 (Growth Differentiation Factor 5) is linked to power. Guilherme, J.P.L.F.; et al. (2022) "Genomic Predictors of Brisk Walking Are Associated with Elite Sprinter Status" identified this association. GDF5 is involved in skeletal development and soft tissue repair, potentially influencing musculoskeletal characteristics favorable for power. The reference allele is G (alternative A).

Power

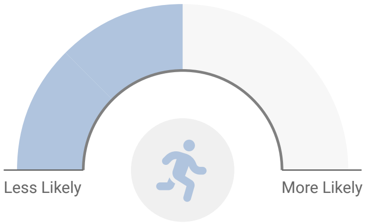

There's no strong evidence linking the A/A genotype with power traits in the HSD17B14 gene.

• **Gene:** HSD17B14 • **rsID:** rs7247312 • **Your Genotype:** A/A • **Zygosity:** homo_ref

Description

Several characteristics are positively associated with power performance, including circulating levels of testosterone, percentage and cross-sectional area of fast-twitch muscle fibres, muscle mass and strength, body and calcaneus height, muscle fascicle length, and reaction time, among others [3,238,239,240,241,242,243,244]. The heritability of power-related phenotypes has been reported in the literature to range from approximately 49 to 86% in a range of phenotypes, including jumping ability [245,246]. Typically, genetic markers associated with power athlete status are determined by comparing allelic frequencies between power athletes (e.g., 100 m runners, shot putters, arm wrestlers, etc.) and untrained subjects.

Extra Information

The G-allele of rs7247312 in HSD17B14 (Hydroxysteroid 17-Beta Dehydrogenase 14) is linked to power. Pickering, C.; et al. (2019) "A genome-wide association study of sprint performance in elite youth football players" reported this association in young football players, suggesting a role in metabolic pathways relevant to sprint performance. The reference allele is A (alternative G).

Name:
DoB:
Gender:
Report ID: WBWG_01_P001_262
Patient ID: 01_P001_262
Date: 6/7/2025

Power

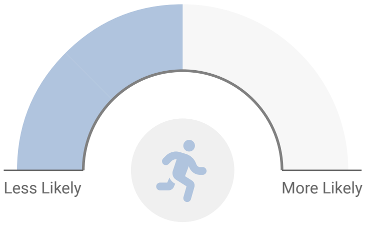

For your Power trait with the IGSF3 gene (rs699785), the G/G genotype isn't strongly associated with power performance changes.

• **Gene:** IGSF3 • **rsID:** rs699785 • **Your Genotype:** G/G • **Zygosity:** homo_ref

Description

Several characteristics are positively associated with power performance, including circulating levels of testosterone, percentage and cross-sectional area of fast-twitch muscle fibres, muscle mass and strength, body and calcaneus height, muscle fascicle length, and reaction time, among others [3,238,239,240,241,242,243,244]. The heritability of power-related phenotypes has been reported in the literature to range from approximately 49 to 86% in a range of phenotypes, including jumping ability [245,246]. Typically, genetic markers associated with power athlete status are determined by comparing allelic frequencies between power athletes (e.g., 100 m runners, shot putters, arm wrestlers, etc.) and untrained subjects.

Extra Information

The A-allele of rs699785 in IGSF3 (Immunoglobulin Superfamily Member 3) is linked to power. Guilherme, J.P.L.F.; et al. (2022) "Genomic Predictors of Brisk Walking Are Associated with Elite Sprinter Status" identified this association. IGSF3 is involved in cell adhesion and signaling. The reference allele is G (alternatives A,C,T).

Power

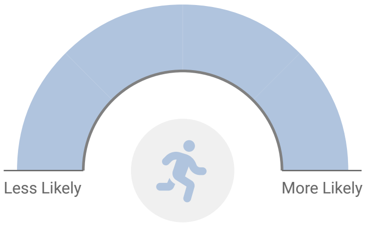

For your power trait with the ILRUN gene (rs205262), having the A/A genotype could boost your sprinting or weightlifting prowess.

• **Gene:** ILRUN • **rsID:** rs205262 • **Your Genotype:** A/A • **Zygosity:** homo_ref

Description

Several characteristics are positively associated with power performance, including circulating levels of testosterone, percentage and cross-sectional area of fast-twitch muscle fibres, muscle mass and strength, body and calcaneus height, muscle fascicle length, and reaction time, among others [3,238,239,240,241,242,243,244]. The heritability of power-related phenotypes has been reported in the literature to range from approximately 49 to 86% in a range of phenotypes, including jumping ability [245,246]. Typically, genetic markers associated with power athlete status are determined by comparing allelic frequencies between power athletes (e.g., 100 m runners, shot putters, arm wrestlers, etc.) and untrained subjects.

Extra Information

The A-allele of rs205262 near ILRUN is linked to power. Guilherme, J.P.L.F.; et al. (2022) "Genomic Predictors of Brisk Walking Are Associated with Elite Sprinter Status" reported this association, suggesting ILRUN might be involved in inflammatory or stress response pathways relevant to power. The reference allele is A (alternative G).

Power

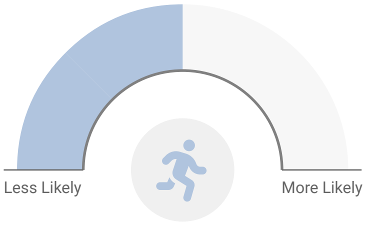

• **Gene:** PIEZO1 • **rsID:** rs572934641 • **Your Genotype:** TCCTCCTC[illegible]CCT/ TCCTCCTCCTCCTCCTCCTCCT • **Zygosity:** homo_ref

Description

Several characteristics are positively associated with power performance, including circulating levels of testosterone, percentage and cross-sectional area of fast-twitch muscle fibres, muscle mass and strength, body and calcaneus height, muscle fascicle length, and reaction time, among others [3,238,239,240,241,242,243,244]. The heritability of power-related phenotypes has been reported in the literature to range from approximately 49 to 86% in a range of phenotypes, including jumping ability [245,246]. Typically, genetic markers associated with power athlete status are determined by comparing allelic frequencies between power athletes (e.g., 100 m runners, shot putters, arm wrestlers, etc.) and untrained subjects.

Extra Information

The D-allele (deletion) of rs572934641 in PIEZO1 (mechanosensitive ion channel) is linked to power. Nakamichi, R.; et al. (2022) "The mechanosensitive ion channel PIEZO1 is expressed in tendons and regulates physical performance" found this deletion more prevalent in power athletes, possibly influencing muscle's response to mechanical stimuli or red blood cell function. The reference allele is a longer repeat sequence.

Power

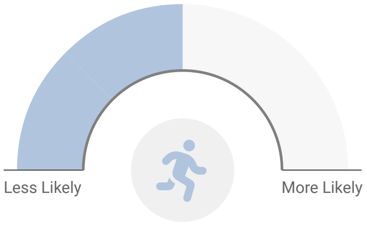

For G/G in PPARA, no meaningful impact on power is evident.

• **Gene:** PPARA • **rsID:** rs4253778 • **Your Genotype:** G/G • **Zygosity:** homo_ref

Description

Several characteristics are positively associated with power performance, including circulating levels of testosterone, percentage and cross-sectional area of fast-twitch muscle fibres, muscle mass and strength, body and calcaneus height, muscle fascicle length, and reaction time, among others [3,238,239,240,241,242,243,244]. The heritability of power-related phenotypes has been reported in the literature to range from approximately 49 to 86% in a range of phenotypes, including jumping ability [245,246]. Typically, genetic markers associated with power athlete status are determined by comparing allelic frequencies between power athletes (e.g., 100 m runners, shot putters, arm wrestlers, etc.) and untrained subjects.

Extra Information

The C-allele of rs4253778 in PPARA (lipid metabolism regulator) is linked to power. Ginevičienė, V.; et al. (2010) "Relating fitness phenotypes to genotypes in Lithuanian elite athletes," Ahmetov, I.I.; et al. (2006) "PPARalpha gene variation and physical performance in Russian athletes," and Maciejewska-Skrendo, A.; et al. (2021) "Does the PPARA Intron 7 Gene Variant (rs4253778) Influence Performance in Power/ Strength-Oriented Athletes?..." reported positive associations, possibly via effects on muscle fiber composition or metabolism. The reference allele is G (alternatives C,T).

Power

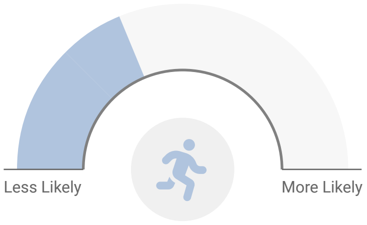

C/C might not greatly influence your power characteristics based on current data.

• **Gene:** PPARG • **rsID:** rs1801282 • **Your Genotype:** C/C • **Zygosity:** homo_ref

Description

Several characteristics are positively associated with power performance, including circulating levels of testosterone, percentage and cross-sectional area of fast-twitch muscle fibres, muscle mass and strength, body and calcaneus height, muscle fascicle length, and reaction time, among others [3,238,239,240,241,242,243,244]. The heritability of power-related phenotypes has been reported in the literature to range from approximately 49 to 86% in a range of phenotypes, including jumping ability [245,246]. Typically, genetic markers associated with power athlete status are determined by comparing allelic frequencies between power athletes (e.g., 100 m runners, shot putters, arm wrestlers, etc.) and untrained subjects.

Extra Information

The G-allele (Ala12) of rs1801282 (Pro12Ala) in PPARG is linked to power. Drozdovska, S.B.; et al. (2013) "The association of gene polymorphisms with athlete status in ukrainians," Ahmetov, I.I.; et al. (2008) "PPARG Gene polymorphism and locomotor activity in humans," and Maciejewska-Karlowska, A.; et al. (2013) "Association between the Pro12Ala polymorphism of the peroxisome proliferator-activated receptor γ gene and strength athlete status" reported positive associations. Guilherme, J.P.L.F.; et al. (2018) "Analysis of sports-relevant polymorphisms in a large Brazilian cohort of top-level athletes" found conflicting results. The reference allele is C (Pro12).

Power

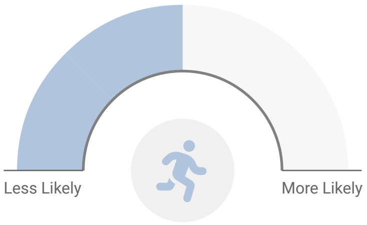

The C/C genotype doesn't show notable effects on your power performance.

• **Gene:** PPARG • **rsID:** rs2920503 • **Your Genotype:** C/C • **Zygosity:** homo_ref

Description

Several characteristics are positively associated with power performance, including circulating levels of testosterone, percentage and cross-sectional area of fast-twitch muscle fibres, muscle mass and strength, body and calcaneus height, muscle fascicle length, and reaction time, among others [3,238,239,240,241,242,243,244]. The heritability of power-related phenotypes has been reported in the literature to range from approximately 49 to 86% in a range of phenotypes, including jumping ability [245,246]. Typically, genetic markers associated with power athlete status are determined by comparing allelic frequencies between power athletes (e.g., 100 m runners, shot putters, arm wrestlers, etc.) and untrained subjects.

Extra Information

The T-allele of rs2920503 in PPARG is linked to power. Guilherme, J.P.L.F.; et al. (2022) "Genomic Predictors of Brisk Walking Are Associated with Elite Sprinter Status" reported an association, suggesting PPARG's role in metabolism and body composition could influence power phenotypes. The reference allele is C (alternative T).

Power

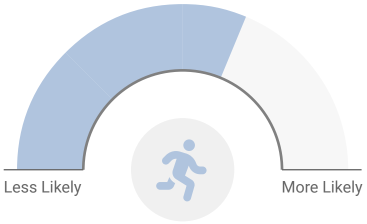

You could experience enhanced power performance with the C/C genotype in SLC39A8.

• **Gene:** SLC39A8 • **rsID:** rs13107325 • **Your Genotype:** C/C • **Zygosity:** homo_ref

Description

Several characteristics are positively associated with power performance, including circulating levels of testosterone, percentage and cross-sectional area of fast-twitch muscle fibres, muscle mass and strength, body and calcaneus height, muscle fascicle length, and reaction time, among others [3,238,239,240,241,242,243,244]. The heritability of power-related phenotypes has been reported in the literature to range from approximately 49 to 86% in a range of phenotypes, including jumping ability [245,246]. Typically, genetic markers associated with power athlete status are determined by comparing allelic frequencies between power athletes (e.g., 100 m runners, shot putters, arm wrestlers, etc.) and untrained subjects.

Extra Information

The C-allele of rs13107325 in SLC39A8 (metal ion transporter) is linked to power. Guilherme, J.P.L.F.; et al. (2022) "Genomic Predictors of Brisk Walking Are Associated with Elite Sprinter Status" identified this association. SLC39A8 is involved in zinc and manganese transport, crucial for enzyme function. The reference allele is C (alternatives A,T).

Power

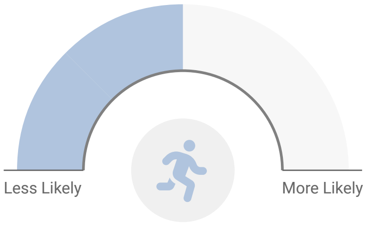

With the C/C genotype in the TRHR gene variant rs7832552, we don't see notable power performance effects.

• **Gene:** TRHR • **rsID:** rs7832552 • **Your Genotype:** C/C • **Zygosity:** homo_ref

Description

Several characteristics are positively associated with power performance, including circulating levels of testosterone, percentage and cross-sectional area of fast-twitch muscle fibres, muscle mass and strength, body and calcaneus height, muscle fascicle length, and reaction time, among others [3,238,239,240,241,242,243,244]. The heritability of power-related phenotypes has been reported in the literature to range from approximately 49 to 86% in a range of phenotypes, including jumping ability [245,246]. Typically, genetic markers associated with power athlete status are determined by comparing allelic frequencies between power athletes (e.g., 100 m runners, shot putters, arm wrestlers, etc.) and untrained subjects.

Extra Information

The T-allele of rs7832552 in TRHR (Thyrotropin Releasing Hormone Receptor) is linked to power. Saito, M.; et al. (2022) "Genetic profile of sports climbing athletes from three different ethnicities," Miyamoto-Mikami, E.; et al. (2017) "Lack of association between genotype score and sprint/power performance in the Japanese population" (paradoxically listed here but potentially refers to individual SNP vs score), and Khanal, P.; et al. (2020) "The Association of Multiple Gene Variants with Ageing Skeletal Muscle Phenotypes in Elderly Women" reported associations. TRHR influences thyroid hormone signaling. The reference allele is C.

Power

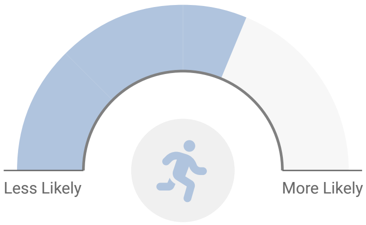

With the UBR5 gene rs10505025, having an A/A genotype may be associated with enhanced power performance, like being able to jump higher.

• **Gene:** UBR5 • **rsID:** rs10505025 • **Your Genotype:** A/A • **Zygosity:** homo_ref

Description

Several characteristics are positively associated with power performance, including circulating levels of testosterone, percentage and cross-sectional area of fast-twitch muscle fibres, muscle mass and strength, body and calcaneus height, muscle fascicle length, and reaction time, among others [3,238,239,240,241,242,243,244]. The heritability of power-related phenotypes has been reported in the literature to range from approximately 49 to 86% in a range of phenotypes, including jumping ability [245,246]. Typically, genetic markers associated with power athlete status are determined by comparing allelic frequencies between power athletes (e.g., 100 m runners, shot putters, arm wrestlers, etc.) and untrained subjects.

Extra Information

The A-allele of rs10505025 near UBR5 is linked to power. Seaborne, R.A.; et al. (2019) "UBR5 is a novel E3 ubiquitin ligase involved in skeletal muscle hypertrophy and recovery from atrophy" (functional study supporting UBR5's role) provides context for UBR5 variants influencing muscle phenotypes relevant to power. The reference allele is A (alternative G).

Power

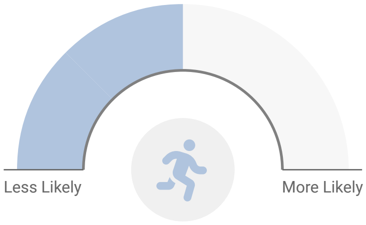

Your T/T genotype in UBR5 rs4734621 shows no clear evidence of influence on power performance.

• **Gene:** UBR5 • **rsID:** rs4734621 • **Your Genotype:** T/T • **Zygosity:** homo_ref

Description

Several characteristics are positively associated with power performance, including circulating levels of testosterone, percentage and cross-sectional area of fast-twitch muscle fibres, muscle mass and strength, body and calcaneus height, muscle fascicle length, and reaction time, among others [3,238,239,240,241,242,243,244]. The heritability of power-related phenotypes has been reported in the literature to range from approximately 49 to 86% in a range of phenotypes, including jumping ability [245,246]. Typically, genetic markers associated with power athlete status are determined by comparing allelic frequencies between power athletes (e.g., 100 m runners, shot putters, arm wrestlers, etc.) and untrained subjects.

Extra Information

The A-allele of rs4734621 near UBR5 (protein degradation, muscle remodeling) is linked to power. Seaborne, R.A.; et al. (2019) "UBR5 is a novel E3 ubiquitin ligase involved in skeletal muscle hypertrophy and recovery from atrophy" (while primarily functional, contextually supports UBR5's role) and studies on athlete cohorts suggest UBR5 variants may modulate muscle protein turnover beneficially for power. The reference allele is T (alternatives C,G).

Name:
DoB:
Gender:

Report ID: WBWG_01_P001_262
Patient ID: 01_P001_262
Date: 6/7/2025

Power

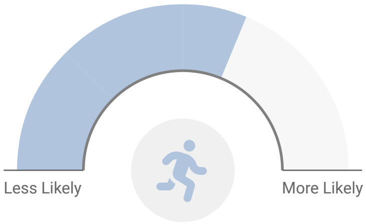

With the A/A genotype of your ZNF568 gene, you might have a natural edge in power activities like sprinting or shot putting.

• **Gene:** ZNF568 • **rsID:** rs1667369 • **Your Genotype:** A/A • **Zygosity:** homo_ref

Description

Several characteristics are positively associated with power performance, including circulating levels of testosterone, percentage and cross-sectional area of fast-twitch muscle fibres, muscle mass and strength, body and calcaneus height, muscle fascicle length, and reaction time, among others [3,238,239,240,241,242,243,244]. The heritability of power-related phenotypes has been reported in the literature to range from approximately 49 to 86% in a range of phenotypes, including jumping ability [245,246]. Typically, genetic markers associated with power athlete status are determined by comparing allelic frequencies between power athletes (e.g., 100 m runners, shot putters, arm wrestlers, etc.) and untrained subjects.

Extra Information

The A-allele of rs1667369 in ZNF568 (Zinc Finger Protein 568) is linked to power. Guilherme, J.P.L.F.; et al. (2022) "Genomic Predictors of Brisk Walking Are Associated with Elite Sprinter Status" reported this association. Zinc finger proteins are often transcription factors regulating gene expression. The reference allele is A (alternative C).

Strength

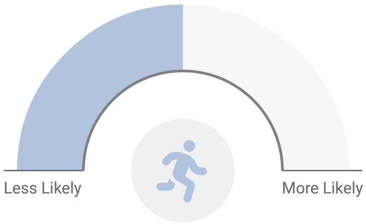

For your Strength trait with the GBF1 gene (rs2273555), carrying the A/A genotype might moderately enhance your strength performance.

• **Gene:** GBF1 • **rsID:** rs2273555 • **Your Genotype:** G/G>A/A • **Zygosity:** Homozygous

Description

Performance in strength-based sports is based on multiple factors. However, the factors considered to contribute substantially to strength phenotypes include skeletal muscle hypertrophy (muscle fibre size), hyperplasia, the predominance of fast-twitch muscle fibres, a greater muscle fascicle pennation angle, improved neurological adaptation, high glycolytic capacity, and increased circulatory testosterone [297]. Importantly, evidence exists that strength athletes exhibit vastly different transcriptomic, biochemical, anthropometric, physiological, and biomechanical characteristics compared to endurance athletes and/or controls

Extra Information

The A-allele of rs2273555 in GBF1 (Golgi Brefeldin A Resistant Guanine Nucleotide Exchange Factor 1) is linked to strength. A GWAS by Willems, S.M.; et al. (2017) "Large-scale GWAS identifies multiple loci for hand grip strength..." and athlete studies by Grishina, E.E.; et al. (2019) "Three DNA Polymorphisms Previously Identified as Markers for Handgrip Strength Are Associated with Strength in Weightlifters..." support this. GBF1 is involved in intracellular trafficking. The reference allele is G (alternatives A,T).

Strength

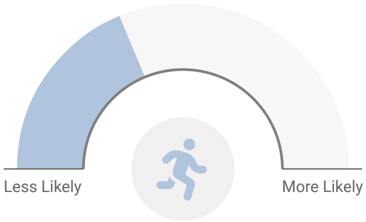

For your strength trait with ACTN3 (rs1815739), T/T could be less beneficial for maximum power output.

• **Gene:** ACTN3 • **rsID:** rs1815739 • **Your Genotype:** C/C>T/T • **Zygosity:** Homozygous

Description

Performance in strength-based sports is based on multiple factors. However, the factors considered to contribute substantially to strength phenotypes include skeletal muscle hypertrophy (muscle fibre size), hyperplasia, the predominance of fast-twitch muscle fibres, a greater muscle fascicle pennation angle, improved neurological adaptation, high glycolytic capacity, and increased circulatory testosterone [297]. Importantly, evidence exists that strength athletes exhibit vastly different transcriptomic, biochemical, anthropometric, physiological, and biomechanical characteristics compared to endurance athletes and/or controls

Extra Information

The C-allele (R-allele) of rs1815739 (R577X) in ACTN3, coding for α-actinin-3, is linked to strength/power. Studies like Pimjan, L.; et al. (2017) "A Study on ACE, ACTN3, and VDR Genes Polymorphism in Thai Weightlifters," Roth, S.M.; et al. (2007) "The ACTN3 R577X nonsense allele is under-represented in elite-level strength athletes," Erskine, R.M.; et al. (2014) "The individual and combined influence of ACE and ACTN3 genotypes on muscle phenotypes...," and Homma, H.; et al. (2022) "The Association between Total Genotype Score and Athletic Performance in Weightlifters" support this. However, Moreland, E.; et al. (2022) "Polygenic Profile of Elite Strength Athletes," Gineviciene, V.; et al. (2016) "Association analysis of ACE, ACTN3 and PPARGC1A...," Orysiak, J.; et al. (2018) "Individual and Combined Influence of ACE and ACTN3 Genes on Muscle Phenotypes in Polish Athletes," and Ben-Zaken, S.; et al. (2019) "Genetic Variability among Power Athletes..." report conflicting results. The reference allele is C.

Name: Report ID: WBWG_01_P001_262
DoB: Patient ID: 01_P001_262
Gender: Date: 6/7/2025

Strength

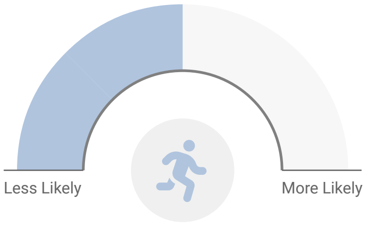

The A/G genotype under IGF1 for strength doesn't have a strong correlation with particular traits.

• **Gene:** IGF1 • **rsID:** rs35767 • **Your Genotype:** A/A>A/G • **Zygosity:** Heterozygous

Description

Performance in strength-based sports is based on multiple factors. However, the factors considered to contribute substantially to strength phenotypes include skeletal muscle hypertrophy (muscle fibre size), hyperplasia, the predominance of fast-twitch muscle fibres, a greater muscle fascicle pennation angle, improved neurological adaptation, high glycolytic capacity, and increased circulatory testosterone [297]. Importantly, evidence exists that strength athletes exhibit vastly different transcriptomic, biochemical, anthropometric, physiological, and biomechanical characteristics compared to endurance athletes and/or controls

Extra Information

The T-allele of rs35767 in the IGF1 promoter (muscle growth hormone) is linked to strength. Moreland, E.; et al. (2022) "Polygenic Profile of Elite Strength Athletes," Ben-Zaken, S.; et al. (2013) "Can IGF-I polymorphism affect power and endurance athletic performance?," and Kostek, M.C.; et al. (2010) "A polymorphism near IGF1 is associated with body composition and muscle function in women..." reported associations with strength or muscle mass, likely through IGF-1 modulation. The reference allele is A (alternatives C,G,T).

Strength

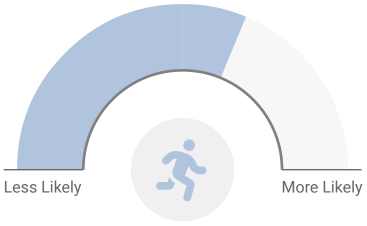

The T/T genotype in the HIF1A gene (rs11549465) could modestly boost your strength abilities.

• **Gene:** HIF1A • **rsID:** rs11549465 • **Your Genotype:** C/C>T/T • **Zygosity:** Homozygous

Description

Performance in strength-based sports is based on multiple factors. However, the factors considered to contribute substantially to strength phenotypes include skeletal muscle hypertrophy (muscle fibre size), hyperplasia, the predominance of fast-twitch muscle fibres, a greater muscle fascicle pennation angle, improved neurological adaptation, high glycolytic capacity, and increased circulatory testosterone [297]. Importantly, evidence exists that strength athletes exhibit vastly different transcriptomic, biochemical, anthropometric, physiological, and biomechanical characteristics compared to endurance athletes and/or controls

Extra Information

The T-allele (Ser582) of rs11549465 (Pro582Ser) in HIF1A (hypoxia response) is linked to strength. Gabbasov, R.T.; et al. (2013) "The HIF1A Gene Pro582Ser Polymorphism in Russian Strength Athletes," Khanal, P.; et al. (2020) "The Association of Multiple Gene Variants with Ageing Skeletal Muscle Phenotypes in Elderly Women," and Ahmetov, I.I.; et al. (2008) "Effect of HIF1A gene polymorphism on human muscle performance" suggest an advantage for strength athletes. Moreland, E.; et al. (2022) "Polygenic Profile of Elite Strength Athletes" had contrary findings. The reference allele is C.

Strength

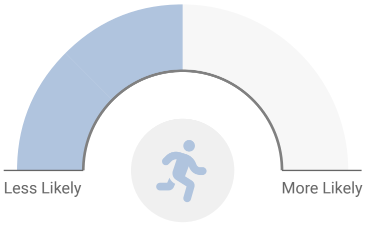

For your Strength trait with the ADPGK gene (rs4776614), the C/G genotype doesn't demonstrate a significant effect.

• **Gene:** ADPGK • **rsID:** rs4776614 • **Your Genotype:** C/C>C/G • **Zygosity:** Heterozygous

Description

Performance in strength-based sports is based on multiple factors. However, the factors considered to contribute substantially to strength phenotypes include skeletal muscle hypertrophy (muscle fibre size), hyperplasia, the predominance of fast-twitch muscle fibres, a greater muscle fascicle pennation angle, improved neurological adaptation, high glycolytic capacity, and increased circulatory testosterone [297]. Importantly, evidence exists that strength athletes exhibit vastly different transcriptomic, biochemical, anthropometric, physiological, and biomechanical characteristics compared to endurance athletes and/or controls

Extra Information

The C-allele of rs4776614 associated with ADPGK is linked to strength. Tikkanen, E.; et al. (2018) "Biological Insights into Muscular Strength: Genetic Findings in the UK Biobank" (GWAS) and Moreland, E.; et al. (2022) "Polygenic Profile of Elite Strength Athletes" (athlete studies) suggest the C-allele contributes to higher strength, possibly via ADPGK's role in glucose metabolism. The reference allele is C (alternatives A,G).

Strength

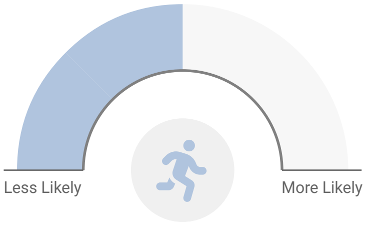

Possessing the A/G genotype in the DHODH gene (rs12599952) doesn't show any strong links to strength performance.

• **Gene:** DHODH • **rsID:** rs12599952 • **Your Genotype:** G/G>G/A • **Zygosity:** Heterozygous

Description

Performance in strength-based sports is based on multiple factors. However, the factors considered to contribute substantially to strength phenotypes include skeletal muscle hypertrophy (muscle fibre size), hyperplasia, the predominance of fast-twitch muscle fibres, a greater muscle fascicle pennation angle, improved neurological adaptation, high glycolytic capacity, and increased circulatory testosterone [297]. Importantly, evidence exists that strength athletes exhibit vastly different transcriptomic, biochemical, anthropometric, physiological, and biomechanical characteristics compared to endurance athletes and/or controls

Extra Information

The A-allele of rs12599952 related to DHODH (pyrimidine biosynthesis) is linked to strength. A GWAS by Tikkanen, E.; et al. (2018) "Biological Insights into Muscular Strength: Genetic Findings in the UK Biobank" and Moreland, E.; et al. (2022) "Polygenic Profile of Elite Strength Athletes" in athletes indicated the A-allele is linked to greater strength. The reference allele is G (alternatives A,C,T).

Strength

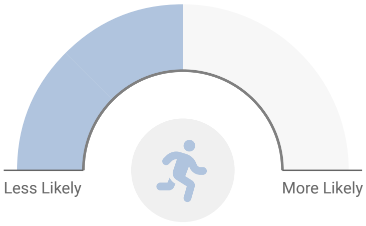

For your strength trait with the ACTG1 gene (rs6565586), having the A/A genotype might slightly enhance your muscle strength.

• **Gene:** ACTG1 • **rsID:** rs6565586 • **Your Genotype:** T/T>A/A • **Zygosity:** Homozygous

Description

Performance in strength-based sports is based on multiple factors. However, the factors considered to contribute substantially to strength phenotypes include skeletal muscle hypertrophy (muscle fibre size), hyperplasia, the predominance of fast-twitch muscle fibres, a greater muscle fascicle pennation angle, improved neurological adaptation, high glycolytic capacity, and increased circulatory testosterone [297]. Importantly, evidence exists that strength athletes exhibit vastly different transcriptomic, biochemical, anthropometric, physiological, and biomechanical characteristics compared to endurance athletes and/or controls

Extra Information

The A-allele of rs6565586 near ACTG1 (muscle actin) is linked to strength. A GWAS by Willems, S.M.; et al. (2017) "Large-scale GWAS identifies multiple loci for hand grip strength providing biological insights into muscular fitness" and athlete studies by Moreland, E.; et al. (2022) "Polygenic Profile of Elite Strength Athletes" support this, suggesting influence on muscle structure/contractile properties. The reference allele is T (alternatives A,C,G).

Strength

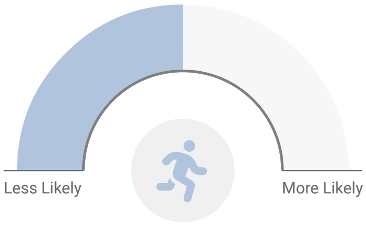

For your Strength trait with the KIF1B gene (rs11121542), having the A/G genotype doesn't show notable effects.

• **Gene:** KIF1B • **rsID:** rs11121542 • **Your Genotype:** G/G>G/A • **Zygosity:** Heterozygous

Description

Performance in strength-based sports is based on multiple factors. However, the factors considered to contribute substantially to strength phenotypes include skeletal muscle hypertrophy (muscle fibre size), hyperplasia, the predominance of fast-twitch muscle fibres, a greater muscle fascicle pennation angle, improved neurological adaptation, high glycolytic capacity, and increased circulatory testosterone [297]. Importantly, evidence exists that strength athletes exhibit vastly different transcriptomic, biochemical, anthropometric, physiological, and biomechanical characteristics compared to endurance athletes and/or controls

Extra Information

The G-allele of rs11121542 in KIF1B (kinesin family member) is linked to strength. A GWAS by Tikkanen, E.; et al. (2018) "Biological Insights into Muscular Strength: Genetic Findings in the UK Biobank" and athlete studies by Moreland, E.; et al. (2022) "Polygenic Profile of Elite Strength Athletes" support this. KIF1B is involved in intracellular transport. The reference allele is G (alternative A).

Strength

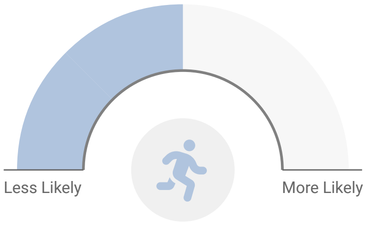

G/T in MTHFR generally doesn't correlate with significant strength changes.

• **Gene:** MTHFR • **rsID:** rs1801131 • **Your Genotype:** T/T>T/G • **Zygosity:** Heterozygous

Description

Performance in strength-based sports is based on multiple factors. However, the factors considered to contribute substantially to strength phenotypes include skeletal muscle hypertrophy (muscle fibre size), hyperplasia, the predominance of fast-twitch muscle fibres, a greater muscle fascicle pennation angle, improved neurological adaptation, high glycolytic capacity, and increased circulatory testosterone [297]. Importantly, evidence exists that strength athletes exhibit vastly different transcriptomic, biochemical, anthropometric, physiological, and biomechanical characteristics compared to endurance athletes and/or controls

Extra Information

The C-allele of rs1801131 (A1298C) in MTHFR (folate metabolism) is linked to strength. Moreland, E.; et al. (2022) "Polygenic Profile of Elite Strength Athletes," Zarebska, A.; et al. (2013) "Association of the MTHFR 1298A>C (rs1801131) polymorphism with speed and strength sports in Russian and Polish athletes," and Kikuchi, N.; et al. (2022) "Genes and Weightlifting Performance" linked the C-allele to strength athletes, possibly via methylation pathways. The reference allele is T (alternative G).

Strength

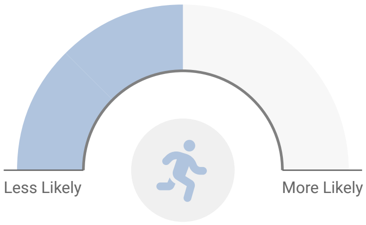

Having the A/T genotype in ADCY3 isn't linked to noticeable strength enhancement.

• **Gene:** ADCY3 • **rsID:** rs10203386 • **Your Genotype:** T/T>T/A • **Zygosity:** Heterozygous

Description

Performance in strength-based sports is based on multiple factors. However, the factors considered to contribute substantially to strength phenotypes include skeletal muscle hypertrophy (muscle fibre size), hyperplasia, the predominance of fast-twitch muscle fibres, a greater muscle fascicle pennation angle, improved neurological adaptation, high glycolytic capacity, and increased circulatory testosterone [297]. Importantly, evidence exists that strength athletes exhibit vastly different transcriptomic, biochemical, anthropometric, physiological, and biomechanical characteristics compared to endurance athletes and/or controls

Extra Information

The T-allele of rs10203386 near ADCY3 (cAMP signaling) is linked to strength. A GWAS by Tikkanen, E.; et al. (2018) "Biological Insights into Muscular Strength: Genetic Findings in the UK Biobank" and athlete studies by Moreland, E.; et al. (2022) "Polygenic Profile of Elite Strength Athletes" support this association, potentially through cAMP pathways influencing muscle. The reference allele is T (alternatives A,G).

Name: Report ID: WBWG_01_P001_262
DoB: Patient ID: 01_P001_262
Gender: Date: 6/7/2025

Strength

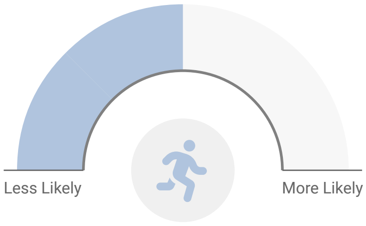

For your Strength trait with the LRPPRC gene (rs10186876), having the A/G genotype doesn't show notable effects.

• **Gene:** LRPPRC • **rsID:** rs10186876 • **Your Genotype:** A/A>A/G • **Zygosity:** Heterozygous

Description

Performance in strength-based sports is based on multiple factors. However, the factors considered to contribute substantially to strength phenotypes include skeletal muscle hypertrophy (muscle fibre size), hyperplasia, the predominance of fast-twitch muscle fibres, a greater muscle fascicle pennation angle, improved neurological adaptation, high glycolytic capacity, and increased circulatory testosterone [297]. Importantly, evidence exists that strength athletes exhibit vastly different transcriptomic, biochemical, anthropometric, physiological, and biomechanical characteristics compared to endurance athletes and/or controls

Extra Information

The A-allele of rs10186876 in LRPPRC (mitochondrial gene expression) is linked to strength. A GWAS by Willems, S.M.; et al. (2017) "Large-scale GWAS identifies multiple loci for hand grip strength..." and athlete studies by Moreland, E.; et al. (2022) "Polygenic Profile of Elite Strength Athletes" and Kikuchi, N.; et al. (2022) "Genes and Weightlifting Performance" support this, suggesting a role for mitochondrial function in strength. The reference allele is A (alternatives G,T).

Strength

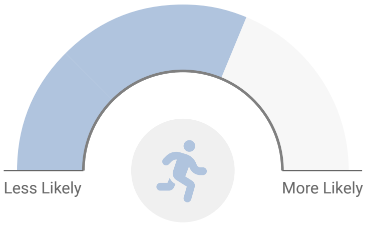

With T/T for R3HDM1, you might enjoy enhanced strength-related traits!

• **Gene:** R3HDM1 • **rsID:** rs6759321 • **Your Genotype:** G/G>T/T • **Zygosity:** Homozygous

Description

Performance in strength-based sports is based on multiple factors. However, the factors considered to contribute substantially to strength phenotypes include skeletal muscle hypertrophy (muscle fibre size), hyperplasia, the predominance of fast-twitch muscle fibres, a greater muscle fascicle pennation angle, improved neurological adaptation, high glycolytic capacity, and increased circulatory testosterone [297]. Importantly, evidence exists that strength athletes exhibit vastly different transcriptomic, biochemical, anthropometric, physiological, and biomechanical characteristics compared to endurance athletes and/or controls

Extra Information

The T-allele of rs6759321 associated with R3HDM1 is linked to strength. A GWAS by Tikkanen, E.; et al. (2018) "Biological Insights into Muscular Strength: Genetic Findings in the UK Biobank" and athlete studies by Moreland, E.; et al. (2022) "Polygenic Profile of Elite Strength Athletes" support this, though the gene's function in muscle is not well established. The reference allele is G (alternatives A,C,T).

Strength

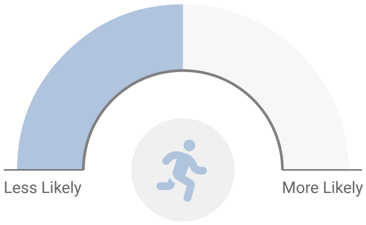

The C/T genotype doesn't show notable effects for strength with ARPP21 based on what we know.

• **Gene:** ARPP21 • **rsID:** rs1513475 • **Your Genotype:** T/T>T/C • **Zygosity:** Heterozygous

Description

Performance in strength-based sports is based on multiple factors. However, the factors considered to contribute substantially to strength phenotypes include skeletal muscle hypertrophy (muscle fibre size), hyperplasia, the predominance of fast-twitch muscle fibres, a greater muscle fascicle pennation angle, improved neurological adaptation, high glycolytic capacity, and increased circulatory testosterone [297]. Importantly, evidence exists that strength athletes exhibit vastly different transcriptomic, biochemical, anthropometric, physiological, and biomechanical characteristics compared to endurance athletes and/or controls

Extra Information

The C-allele of rs1513475 near ARPP21 (cAMP-Regulated Phosphoprotein 21) is linked to strength. A GWAS by Tikkanen, E.; et al. (2018) "Biological Insights into Muscular Strength: Genetic Findings in the UK Biobank" and athlete studies by Moreland, E.; et al. (2022) "Polygenic Profile of Elite Strength Athletes" support this. ARPP21 is involved in dopamine signaling. The reference allele is T (alternatives A,C,G).

Strength

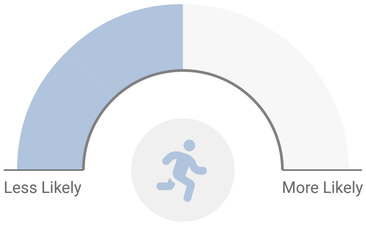

For C/T in PPARGC1A, we don't observe strong strength-related traits.

• **Gene:** PPARGC1A • **rsID:** rs8192678 • **Your Genotype:** C/C>C/T • **Zygosity:** Heterozygous

Description

Performance in strength-based sports is based on multiple factors. However, the factors considered to contribute substantially to strength phenotypes include skeletal muscle hypertrophy (muscle fibre size), hyperplasia, the predominance of fast-twitch muscle fibres, a greater muscle fascicle pennation angle, improved neurological adaptation, high glycolytic capacity, and increased circulatory testosterone [297]. Importantly, evidence exists that strength athletes exhibit vastly different transcriptomic, biochemical, anthropometric, physiological, and biomechanical characteristics compared to endurance athletes and/or controls

Extra Information

The A-allele (Ser482) of rs8192678 (Gly482Ser) in PPARGC1A (mitochondrial biogenesis, muscle fiber type) is linked to strength. Moreland, E.; et al. (2022) "Polygenic Profile of Elite Strength Athletes," Gineviciene, V.; et al. (2016) "Association analysis of ACE, ACTN3 and PPARGC1A gene polymorphisms in two cohorts of European strength and power athletes," and Homma, H.; et al. (2022) "The Association between Total Genotype Score and Athletic Performance in Weightlifters" reported positive associations, possibly by influencing muscle adaptation. The reference allele is C (Gly482).

Strength

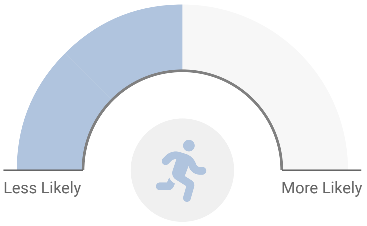

Having an A/G genotype in the GALNTL6 gene (rs558129) shows no marked impact on enhancing your strength.

• **Gene:** GALNTL6 • **rsID:** rs558129 • **Your Genotype:** G/G>G/A • **Zygosity:** Heterozygous

Description

Performance in strength-based sports is based on multiple factors. However, the factors considered to contribute substantially to strength phenotypes include skeletal muscle hypertrophy (muscle fibre size), hyperplasia, the predominance of fast-twitch muscle fibres, a greater muscle fascicle pennation angle, improved neurological adaptation, high glycolytic capacity, and increased circulatory testosterone [297]. Importantly, evidence exists that strength athletes exhibit vastly different transcriptomic, biochemical, anthropometric, physiological, and biomechanical characteristics compared to endurance athletes and/or controls

Extra Information

The T-allele of rs558129 in GALNTL6 is linked to strength/power. Díaz Ramírez, J.; et al. (2020) "The GALNTL6 Gene rs558129 Polymorphism is Associated with Power Performance" identified this association in athletes. GALNTL6's role may relate to protein glycosylation affecting muscle cells. The reference allele is G (alternative A).

Strength

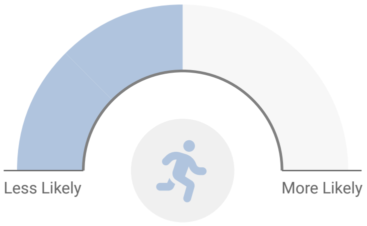

For your PHACTR1-related Strength trait, T/T doesn't appear to influence your abilities in strength sports.

• **Gene:** PHACTR1 • **rsID:** rs6905419 • **Your Genotype:** C/C>T/T • **Zygosity:** Homozygous

Description

Performance in strength-based sports is based on multiple factors. However, the factors considered to contribute substantially to strength phenotypes include skeletal muscle hypertrophy (muscle fibre size), hyperplasia, the predominance of fast-twitch muscle fibres, a greater muscle fascicle pennation angle, improved neurological adaptation, high glycolytic capacity, and increased circulatory testosterone [297]. Importantly, evidence exists that strength athletes exhibit vastly different transcriptomic, biochemical, anthropometric, physiological, and biomechanical characteristics compared to endurance athletes and/or controls

Extra Information

The C-allele of rs6905419 in PHACTR1 is linked to strength. A GWAS by Tikkanen, E.; et al. (2018) "Biological Insights into Muscular Strength: Genetic Findings in the UK Biobank" and athlete studies by Moreland, E.; et al. (2022) "Polygenic Profile of Elite Strength Athletes" and Kikuchi, N.; et al. (2022) "Genes and Weightlifting Performance" support this. PHACTR1 is involved in actin regulation and angiogenesis. The reference allele is C (alternatives A,T).

Strength

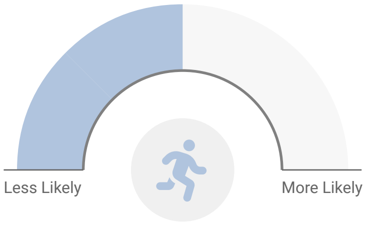

For your Strength trait with the MLN gene (rs12055409), having the A/G genotype doesn't show notable effects.

• **Gene:** MLN • **rsID:** rs12055409 • **Your Genotype:** A/A>A/G • **Zygosity:** Heterozygous

Description

Performance in strength-based sports is based on multiple factors. However, the factors considered to contribute substantially to strength phenotypes include skeletal muscle hypertrophy (muscle fibre size), hyperplasia, the predominance of fast-twitch muscle fibres, a greater muscle fascicle pennation angle, improved neurological adaptation, high glycolytic capacity, and increased circulatory testosterone [297]. Importantly, evidence exists that strength athletes exhibit vastly different transcriptomic, biochemical, anthropometric, physiological, and biomechanical characteristics compared to endurance athletes and/or controls

Extra Information

The G-allele of rs12055409 near MLN (motilin) is linked to strength. A GWAS by Tikkanen, E.; et al. (2018) "Biological Insights into Muscular Strength: Genetic Findings in the UK Biobank" and athlete studies by Grishina, E.E.; et al. (2019) "Three DNA Polymorphisms Previously Identified as Markers for Handgrip Strength Are Associated with Strength in Weightlifters..." support this. The mechanism by which a motilin-related variant influences strength is unclear. The reference allele is A (alternative G).

Strength

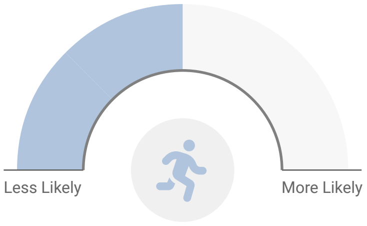

A C/C genotype for MMS22L doesn't appear to have notable effects on your strength.

• **Gene:** MMS22L • **rsID:** rs9320823 • **Your Genotype:** T/T>C/C • **Zygosity:** Homozygous

Description

Performance in strength-based sports is based on multiple factors. However, the factors considered to contribute substantially to strength phenotypes include skeletal muscle hypertrophy (muscle fibre size), hyperplasia, the predominance of fast-twitch muscle fibres, a greater muscle fascicle pennation angle, improved neurological adaptation, high glycolytic capacity, and increased circulatory testosterone [297]. Importantly, evidence exists that strength athletes exhibit vastly different transcriptomic, biochemical, anthropometric, physiological, and biomechanical characteristics compared to endurance athletes and/or controls

Extra Information

The T-allele of rs9320823, related to MMS22L (DNA repair), is linked to strength. A GWAS by Tikkanen, E.; et al. (2018) "Biological Insights into Muscular Strength: Genetic Findings in the UK Biobank" and athlete studies by Moreland, E.; et al. (2022) "Polygenic Profile of Elite Strength Athletes" and Kikuchi, N.; et al. (2022) "Genes and Weightlifting Performance" support this, possibly due to its role in muscle cell health. The reference allele is T (alternatives A,C).

Name:
DoB:
Gender:
Report ID: WBWG_01_P001_262
Patient ID: 01_P001_262
Date: 6/7/2025

Strength

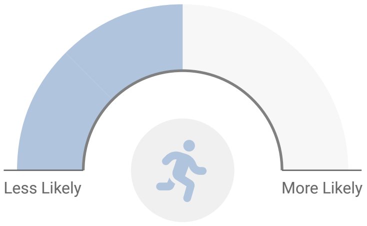

Having the C/G genotype might offer slight benefits for strength, given some connection to the G allele.

• **Gene:** IL6 • **rsID:** rs1800795 • **Your Genotype:** C/C>C/G • **Zygosity:** Heterozygous

Description

Performance in strength-based sports is based on multiple factors. However, the factors considered to contribute substantially to strength phenotypes include skeletal muscle hypertrophy (muscle fibre size), hyperplasia, the predominance of fast-twitch muscle fibres, a greater muscle fascicle pennation angle, improved neurological adaptation, high glycolytic capacity, and increased circulatory testosterone [297]. Importantly, evidence exists that strength athletes exhibit vastly different transcriptomic, biochemical, anthropometric, physiological, and biomechanical characteristics compared to endurance athletes and/or controls

Extra Information

The G-allele of rs1800795 in the IL6 promoter is linked to strength. Moreland, E.; et al. (2022) "Polygenic Profile of Elite Strength Athletes" and Ruiz, J.R.; et al. (2010) "The -174 G/C polymorphism of the IL6 gene is associated with elite power performance" linked the G-allele to strength athletes or beneficial training responses, possibly via IL-6's role in muscle hypertrophy/repair. The reference allele is C (alternatives G,T).

Strength

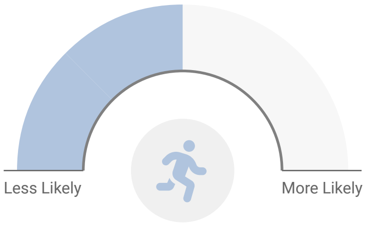

• **Gene:** GLIS3 • **rsID:** rs34706136 • **Your Genotype:** T/T>T/TG• **Zygosity:** Heterozygous

Description

Performance in strength-based sports is based on multiple factors. However, the factors considered to contribute substantially to strength phenotypes include skeletal muscle hypertrophy (muscle fibre size), hyperplasia, the predominance of fast-twitch muscle fibres, a greater muscle fascicle pennation angle, improved neurological adaptation, high glycolytic capacity, and increased circulatory testosterone [297]. Importantly, evidence exists that strength athletes exhibit vastly different transcriptomic, biochemical, anthropometric, physiological, and biomechanical characteristics compared to endurance athletes and/or controls

Extra Information

The TG allele (likely an indel/haplotype) of rs34706136 near GLIS3 is linked to strength. Guilherme, J.P.L.F.; et al. (2022) "Genomic predictors of testosterone levels are associated with muscle fiber size and strength" found this variant associated with muscle fiber size and strength, potentially via testosterone regulation. The reference allele is '-' (alternative G).

Strength

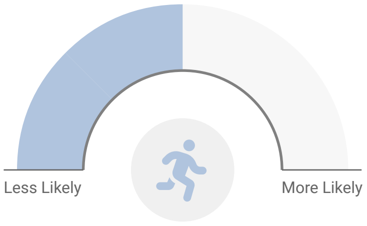

For your Strength trait, currently, we don't see a notable effect of the G/G genotype with your ABHD17C gene.

• **Gene:** ABHD17C • **rsID:** rs7165759 • **Your Genotype:** G/G • **Zygosity:** homo_ref

Description

Performance in strength-based sports is based on multiple factors. However, the factors considered to contribute substantially to strength phenotypes include skeletal muscle hypertrophy (muscle fibre size), hyperplasia, the predominance of fast-twitch muscle fibres, a greater muscle fascicle pennation angle, improved neurological adaptation, high glycolytic capacity, and increased circulatory testosterone [297]. Importantly, evidence exists that strength athletes exhibit vastly different transcriptomic, biochemical, anthropometric, physiological, and biomechanical characteristics compared to endurance athletes and/or controls

Extra Information

The A-allele of rs7165759 in the ABHD17C gene region is linked to strength. A GWAS by Tikkanen, E.; et al. (2018) "Biological Insights into Muscular Strength: Genetic Findings in the UK Biobank" and athlete analyses by Moreland, E.; et al. (2022) "Polygenic Profile of Elite Strength Athletes" support this association. The reference allele is G (alternative A).

Strength

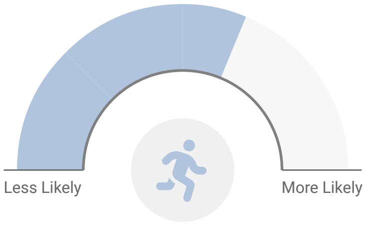

For your Strength trait with the ACE gene (rs4343), having the G/G genotype could enhance your performance in activities requiring strength.

• **Gene:** ACE • **rsID:** rs4343 • **Your Genotype:** G/G • **Zygosity:** homo_ref

Description

Performance in strength-based sports is based on multiple factors. However, the factors considered to contribute substantially to strength phenotypes include skeletal muscle hypertrophy (muscle fibre size), hyperplasia, the predominance of fast-twitch muscle fibres, a greater muscle fascicle pennation angle, improved neurological adaptation, high glycolytic capacity, and increased circulatory testosterone [297]. Importantly, evidence exists that strength athletes exhibit vastly different transcriptomic, biochemical, anthropometric, physiological, and biomechanical characteristics compared to endurance athletes and/or controls

Extra Information

The G-allele of rs4343 in ACE (often linked to D-allele of ACE I/D) is associated with strength/power. Colakoglu, M.; et al. (2005) "ACE genotype may have an effect on single versus multiple set preferences in strength training," Giaccaglia, V.; et al. (2008) "Interaction between angiotensin converting enzyme insertion/deletion genotype and exercise training on knee extensor strength in older individuals," Pimjan, L.; et al. (2017) "A Study on ACE, ACTN3, and VDR Genes Polymorphism in Thai Weightlifters," Melián Ortiz, A.; et al. (2021) "Muscle Work and Its Relationship with ACE and ACTN3 Polymorphisms Are Associated with the Improvement of Explosive Strength," and Khanal, P.; et al. (2022) "Polygenic Models Partially Predict Muscle Size and Strength..." reported positive links. Gineviciene, V.; et al. (2016) "Association analysis of ACE, ACTN3 and PPARGC1A gene polymorphisms in two cohorts of European strength and power athletes" presented conflicting evidence. (Note: Ref 301 was empty). The reference allele is G.

Strength

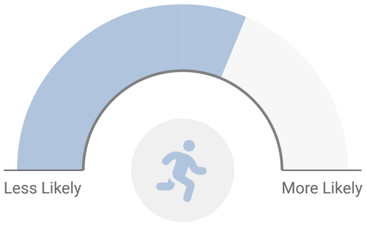

With the G/G genotype for the ACE gene (rs4341), you may gain a beneficial edge in strength-related activities.

• **Gene:** ACE • **rsID:** rs4341 • **Your Genotype:** G/G • **Zygosity:** homo_ref

Description

Performance in strength-based sports is based on multiple factors. However, the factors considered to contribute substantially to strength phenotypes include skeletal muscle hypertrophy (muscle fibre size), hyperplasia, the predominance of fast-twitch muscle fibres, a greater muscle fascicle pennation angle, improved neurological adaptation, high glycolytic capacity, and increased circulatory testosterone [297]. Importantly, evidence exists that strength athletes exhibit vastly different transcriptomic, biochemical, anthropometric, physiological, and biomechanical characteristics compared to endurance athletes and/or controls

Extra Information

The G-allele of rs4341 in ACE (often linked to D-allele of ACE I/D) is associated with strength. Colakoglu, M.; et al. (2005) "ACE genotype may have an effect on single versus multiple set preferences in strength training," Giaccaglia, V.; et al. (2008) "Interaction between angiotensin converting enzyme insertion/deletion genotype and exercise training on knee extensor strength in older individuals," Pimjan, L.; et al. (2017) "A Study on ACE, ACTN3, and VDR Genes Polymorphism in Thai Weightlifters," Melián Ortiz, A.; et al. (2021) "Muscle Work and Its Relationship with ACE and ACTN3 Polymorphisms...," and Khanal, P.; et al. (2022) "Polygenic Models Partially Predict Muscle Size and Strength..." showed positive links. Gineviciene, V.; et al. (2016) "Association analysis of ACE, ACTN3 and PPARGC1A..." presented conflicting results. (Note: Ref 301 was empty). The reference allele is G.

Strength

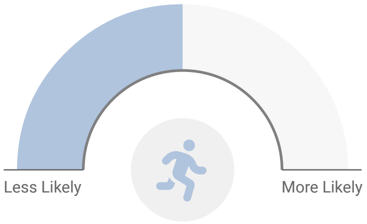

For your Strength trait with the AGT gene (rs699), having the A/A genotype doesn't show strong effects on your strength.

• **Gene:** AGT • **rsID:** rs699 • **Your Genotype:** A/A • **Zygosity:** homo_ref

Description

Performance in strength-based sports is based on multiple factors. However, the factors considered to contribute substantially to strength phenotypes include skeletal muscle hypertrophy (muscle fibre size), hyperplasia, the predominance of fast-twitch muscle fibres, a greater muscle fascicle pennation angle, improved neurological adaptation, high glycolytic capacity, and increased circulatory testosterone [297]. Importantly, evidence exists that strength athletes exhibit vastly different transcriptomic, biochemical, anthropometric, physiological, and biomechanical characteristics compared to endurance athletes and/or controls

Extra Information

The C-allele (T235) of rs699 (M235T) in AGT (angiotensinogen) is linked to strength. Ben-Zaken, S.; et al. (2019) "Genetic Variability among Power Athletes: The Stronger vs. Faster" and Aleksandra, Z.; et al. (2016) "The AGT Gene M235T Polymorphism and Response of Power-Related Variables to Aerobic Training" reported positive associations. However, Moreland, E.; et al. (2022) "Polygenic Profile of Elite Strength Athletes" presented conflicting results. The reference allele is A (M235).

Strength

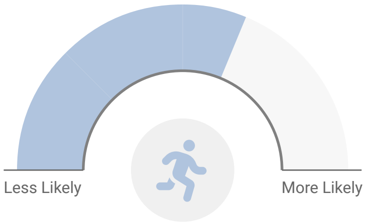

Possessing the G/G genotype in the ALDH2 gene may correlate with improved strength performance!

• **Gene:** ALDH2 • **rsID:** rs671 • **Your Genotype:** G/G • **Zygosity:** homo_ref

Description

Performance in strength-based sports is based on multiple factors. However, the factors considered to contribute substantially to strength phenotypes include skeletal muscle hypertrophy (muscle fibre size), hyperplasia, the predominance of fast-twitch muscle fibres, a greater muscle fascicle pennation angle, improved neurological adaptation, high glycolytic capacity, and increased circulatory testosterone [297]. Importantly, evidence exists that strength athletes exhibit vastly different transcriptomic, biochemical, anthropometric, physiological, and biomechanical characteristics compared to endurance athletes and/or controls

Extra Information

The G-allele (functional enzyme) of rs671 in ALDH2 (alcohol metabolism) is linked to strength in East Asians. Kikuchi, N.; et al. (2022) "The ALDH2 rs671 polymorphism is associated with athletic status and muscle strength in a Japanese population," De Almeida, K.Y.; et al. (2022) "ALDH2 gene polymorphism is associated with fitness in the elderly Japanese population," and Saito, A.; et al. (2022) "The Association between the ALDH2 rs671 Polymorphism and Athletic Performance in Japanese Power and Strength Athletes" suggest GG genotype might have advantages, possibly via efficient aldehyde clearance. The reference allele is G (alternative A).

Strength

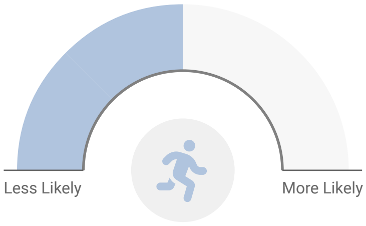

We haven't found any noteworthy effects related to strength for the G/G genotype here.

• **Gene:** ANGPT2 • **rsID:** rs890022 • **Your Genotype:** G/G • **Zygosity:** homo_ref

Description

Performance in strength-based sports is based on multiple factors. However, the factors considered to contribute substantially to strength phenotypes include skeletal muscle hypertrophy (muscle fibre size), hyperplasia, the predominance of fast-twitch muscle fibres, a greater muscle fascicle pennation angle, improved neurological adaptation, high glycolytic capacity, and increased circulatory testosterone [297]. Importantly, evidence exists that strength athletes exhibit vastly different transcriptomic, biochemical, anthropometric, physiological, and biomechanical characteristics compared to endurance athletes and/or controls

Extra Information

The A-allele of rs890022 in ANGPT2 (Angiopoietin 2) is linked to strength. Moreland, E.; et al. (2022) "Polygenic Profile of Elite Strength Athletes" and Matteini, A.M.; et al. (2016) "GWAS analysis of handgrip and lower body strength in older adults in the CHARGE consortium" (GWAS context) support this association. ANGPT2 is involved in angiogenesis and inflammation. The reference allele is G (alternatives A,C).

Strength

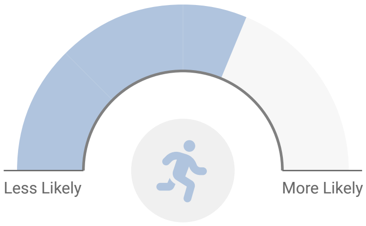

The C/C genotype in BCDIN3D might give you an advantage in strength activities.

• **Gene:** BCDIN3D • **rsID:** rs12367809 • **Your Genotype:** C/C • **Zygosity:** homo_ref

Description

Performance in strength-based sports is based on multiple factors. However, the factors considered to contribute substantially to strength phenotypes include skeletal muscle hypertrophy (muscle fibre size), hyperplasia, the predominance of fast-twitch muscle fibres, a greater muscle fascicle pennation angle, improved neurological adaptation, high glycolytic capacity, and increased circulatory testosterone [297]. Importantly, evidence exists that strength athletes exhibit vastly different transcriptomic, biochemical, anthropometric, physiological, and biomechanical characteristics compared to endurance athletes and/or controls

Extra Information

The C-allele of rs12367809 linked to BCDIN3D is associated with strength. A GWAS by Tikkanen, E.; et al. (2018) "Biological Insights into Muscular Strength: Genetic Findings in the UK Biobank" and athlete cohort studies by Moreland, E.; et al. (2022) "Polygenic Profile of Elite Strength Athletes" support this, suggesting it's a marker for higher strength. The reference allele is C (alternatives A,T).

Strength

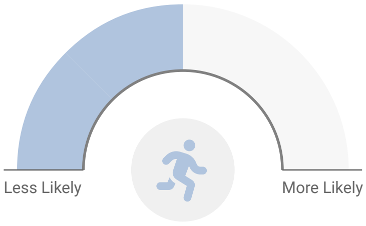

The T/T genotype in CKM does not reveal a significant connection with strength.

• **Gene:** CKM • **rsID:** rs8111989 • **Your Genotype:** T/T • **Zygosity:** homo_ref

Description

Performance in strength-based sports is based on multiple factors. However, the factors considered to contribute substantially to strength phenotypes include skeletal muscle hypertrophy (muscle fibre size), hyperplasia, the predominance of fast-twitch muscle fibres, a greater muscle fascicle pennation angle, improved neurological adaptation, high glycolytic capacity, and increased circulatory testosterone [297]. Importantly, evidence exists that strength athletes exhibit vastly different transcriptomic, biochemical, anthropometric, physiological, and biomechanical characteristics compared to endurance athletes and/or controls

Extra Information

The G-allele of rs8111989 in CKM (muscle creatine kinase) is linked to strength. A meta-analysis by Chen, C.; et al. (2017) "A meta-analysis of the association of CKM gene rs8111989 polymorphism with sport performance" and a study by Fedotovskaia, O.N.; et al. (2012) "Association of the muscle-specific creatine kinase (CKMM) gene polymorphism with physical performance of athletes" reported positive associations. However, Moreland, E.; et al. (2022) "Polygenic Profile of Elite Strength Athletes" and Ginevičienė, V.; et al. (2021) "CKM Gene rs8111989 Polymorphism and Power Athlete Status" presented conflicting results. The G-allele (ref T, alt C, where C is G on opposite strand) may relate to energy metabolism.

Name:
DoB:
Gender:

Report ID: WBWG_01_P001_262
Patient ID: 01_P001_262
Date: 6/7/2025

Strength

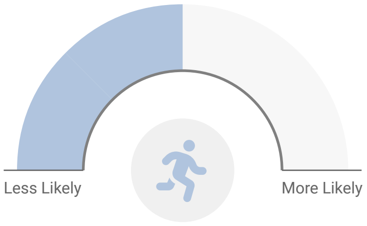

G/G in CNTFR doesn’t align with distinctive strength advantages.

• **Gene:** CNTFR • **rsID:** rs41274853 • **Your Genotype:** G/G • **Zygosity:** homo_ref

Description

Performance in strength-based sports is based on multiple factors. However, the factors considered to contribute substantially to strength phenotypes include skeletal muscle hypertrophy (muscle fibre size), hyperplasia, the predominance of fast-twitch muscle fibres, a greater muscle fascicle pennation angle, improved neurological adaptation, high glycolytic capacity, and increased circulatory testosterone [297]. Importantly, evidence exists that strength athletes exhibit vastly different transcriptomic, biochemical, anthropometric, physiological, and biomechanical characteristics compared to endurance athletes and/or controls

Extra Information

The T-allele of rs41274853 in CNTFR (Ciliary Neurotrophic Factor Receptor) is linked to strength. Miyamoto-Mikami, E.; et al. (2016) "CNTFR Genotype and Sprint/power Performance: Case-control Association and Functional Studies" (power context but relevant) and Homma, H.; et al. (2020) "Ciliary Neurotrophic Factor Receptor rs41274853 Polymorphism Is Associated with Weightlifting Performance in Japanese Weightlifters" reported positive associations. Moreland, E.; et al. (2022) "Polygenic Profile of Elite Strength Athletes" had contrary findings. The reference allele is G (alternatives A,C).

Strength

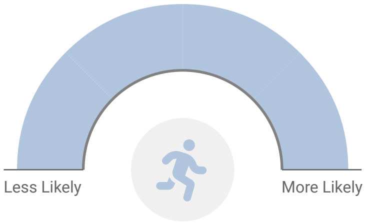

For CRTAC1 (rs563296), a G/G genotype may enhance your strength performance.

• **Gene:** CRTAC1 • **rsID:** rs563296 • **Your Genotype:** G/G • **Zygosity:** homo_ref

Description

Performance in strength-based sports is based on multiple factors. However, the factors considered to contribute substantially to strength phenotypes include skeletal muscle hypertrophy (muscle fibre size), hyperplasia, the predominance of fast-twitch muscle fibres, a greater muscle fascicle pennation angle, improved neurological adaptation, high glycolytic capacity, and increased circulatory testosterone [297]. Importantly, evidence exists that strength athletes exhibit vastly different transcriptomic, biochemical, anthropometric, physiological, and biomechanical characteristics compared to endurance athletes and/or controls

Extra Information

The G-allele of rs563296 in the CRTAC1 gene region is linked to strength. A GWAS by Tikkanen, E.; et al. (2018) "Biological Insights into Muscular Strength: Genetic Findings in the UK Biobank" and athlete studies by Moreland, E.; et al. (2022) "Polygenic Profile of Elite Strength Athletes" support this, suggesting potential effects on musculoskeletal integrity. The reference allele is G (alternatives A,T).

Strength

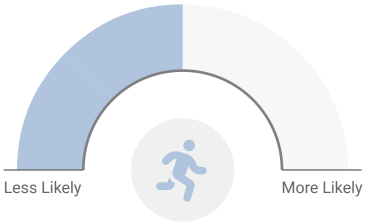

For your Strength trait with the GBE1 gene (rs9877408), having the A/A genotype might offer some benefits in strength-related activities.

• **Gene:** GBE1 • **rsID:** rs9877408 • **Your Genotype:** A/A • **Zygosity:** homo_ref

Description

Performance in strength-based sports is based on multiple factors. However, the factors considered to contribute substantially to strength phenotypes include skeletal muscle hypertrophy (muscle fibre size), hyperplasia, the predominance of fast-twitch muscle fibres, a greater muscle fascicle pennation angle, improved neurological adaptation, high glycolytic capacity, and increased circulatory testosterone [297]. Importantly, evidence exists that strength athletes exhibit vastly different transcriptomic, biochemical, anthropometric, physiological, and biomechanical characteristics compared to endurance athletes and/or controls

Extra Information

The A-allele of rs9877408 in GBE1 (glycogen synthesis) is linked to strength. A GWAS by Tikkanen, E.; et al. (2018) "Biological Insights into Muscular Strength: Genetic Findings in the UK Biobank" and athlete studies by Moreland, E.; et al. (2022) "Polygenic Profile of Elite Strength Athletes" support this, possibly due to efficient glycogen storage impacting muscle energy. The reference allele is A (alternatives C,G,T).

Strength

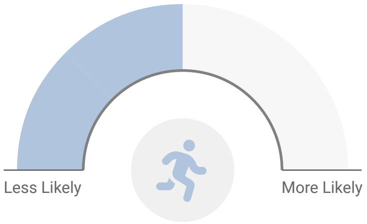

As a G/G carrier, we don't find a strong relationship with strength for your ITPR1 variant.

• **Gene:** ITPR1 • **rsID:** rs901850 • **Your Genotype:** G/G • **Zygosity:** homo_ref

Description

Performance in strength-based sports is based on multiple factors. However, the factors considered to contribute substantially to strength phenotypes include skeletal muscle hypertrophy (muscle fibre size), hyperplasia, the predominance of fast-twitch muscle fibres, a greater muscle fascicle pennation angle, improved neurological adaptation, high glycolytic capacity, and increased circulatory testosterone [297]. Importantly, evidence exists that strength athletes exhibit vastly different transcriptomic, biochemical, anthropometric, physiological, and biomechanical characteristics compared to endurance athletes and/or controls

Extra Information

The T-allele of rs901850 in ITPR1 (calcium release channel) is linked to strength. A GWAS by Tikkanen, E.; et al. (2018) "Biological Insights into Muscular Strength: Genetic Findings in the UK Biobank" and athlete studies by Moreland, E.; et al. (2022) "Polygenic Profile of Elite Strength Athletes" support this, as variations in calcium signaling can directly impact muscle force. The reference allele is G (alternative T).

Name:
DoB:
Gender:
Report ID: WBWG_01_P001_262
Patient ID: 01_P001_262
Date: 6/7/2025

Strength

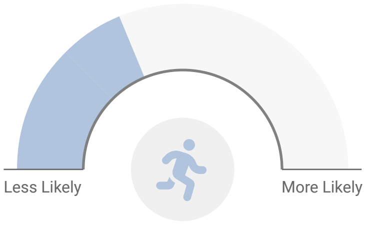

With the A/A genotype in NPIPB6, you might enjoy enhanced strength benefits.

• **Gene:** NPIPB6 • **rsID:** rs2726036 • **Your Genotype:** A/A • **Zygosity:** homo_ref

Description

Performance in strength-based sports is based on multiple factors. However, the factors considered to contribute substantially to strength phenotypes include skeletal muscle hypertrophy (muscle fibre size), hyperplasia, the predominance of fast-twitch muscle fibres, a greater muscle fascicle pennation angle, improved neurological adaptation, high glycolytic capacity, and increased circulatory testosterone [297]. Importantly, evidence exists that strength athletes exhibit vastly different transcriptomic, biochemical, anthropometric, physiological, and biomechanical characteristics compared to endurance athletes and/or controls

Extra Information

The A-allele of rs2726036 near NPIPB6 is linked to strength. A GWAS by Tikkanen, E.; et al. (2018) "Biological Insights into Muscular Strength: Genetic Findings in the UK Biobank" on handgrip strength and Moreland, E.; et al. (2022) "Polygenic Profile of Elite Strength Athletes" in athlete cohorts linked the A-allele to strength phenotypes. The reference allele is A (alternatives C,T).

Strength

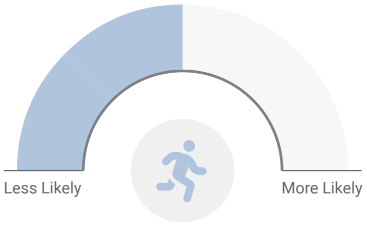

For Strength and the PLEKHB1 gene (rs7128512), A/A doesn't seem to change your potential.

• **Gene:** PLEKHB1 • **rsID:** rs7128512 • **Your Genotype:** A/A • **Zygosity:** homo_ref

Description

Performance in strength-based sports is based on multiple factors. However, the factors considered to contribute substantially to strength phenotypes include skeletal muscle hypertrophy (muscle fibre size), hyperplasia, the predominance of fast-twitch muscle fibres, a greater muscle fascicle pennation angle, improved neurological adaptation, high glycolytic capacity, and increased circulatory testosterone [297]. Importantly, evidence exists that strength athletes exhibit vastly different transcriptomic, biochemical, anthropometric, physiological, and biomechanical characteristics compared to endurance athletes and/or controls

Extra Information

The G-allele of rs7128512 in PLEKHB1 is linked to strength. Moreland, E.; et al. (2022) "Polygenic Profile of Elite Strength Athletes" and a GWAS by Matteini, A.M.; et al. (2016) "GWAS analysis of handgrip and lower body strength in older adults in the CHARGE consortium" support this. PLEKHB1's role in muscle is not fully clear. The reference allele is A (alternatives G,T).

Strength

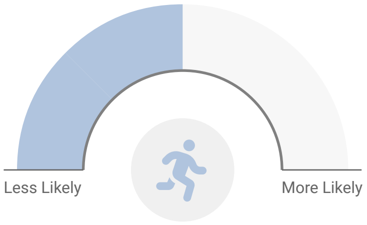

The G/G genotype for Strength with PPARA doesn't indicate a significant impact on your capabilities.

• **Gene:** PPARA • **rsID:** rs4253778 • **Your Genotype:** G/G • **Zygosity:** homo_ref

Description

Performance in strength-based sports is based on multiple factors. However, the factors considered to contribute substantially to strength phenotypes include skeletal muscle hypertrophy (muscle fibre size), hyperplasia, the predominance of fast-twitch muscle fibres, a greater muscle fascicle pennation angle, improved neurological adaptation, high glycolytic capacity, and increased circulatory testosterone [297]. Importantly, evidence exists that strength athletes exhibit vastly different transcriptomic, biochemical, anthropometric, physiological, and biomechanical characteristics compared to endurance athletes and/or controls

Extra Information

The C-allele of rs4253778 in PPARA (lipid metabolism regulator) is linked to strength. Ahmetov, I.I.; et al. (2006) "PPARalpha gene variation and physical performance in Russian athletes," Ahmetov, I.I.; et al. (2013) "The association of ACE, ACTN3 and PPARA gene variants with strength phenotypes in middle school-age children," and Petr, M.; et al. (2014) "PPARA intron polymorphism associated with power performance in 30-s anaerobic Wingate Test" (power context but relevant) reported positive associations. Moreland, E.; et al. (2022) "Polygenic Profile of Elite Strength Athletes" had contrary findings. The reference allele is G (alternatives C,T).

Strength

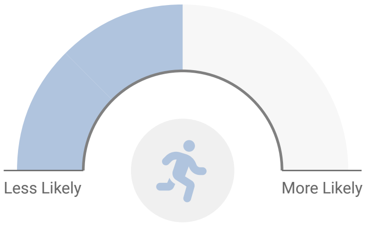

Having C/C under the PPARG gene doesn't show a strong impact on your strength capabilities.

• **Gene:** PPARG • **rsID:** rs1801282 • **Your Genotype:** C/C • **Zygosity:** homo_ref

Description

Performance in strength-based sports is based on multiple factors. However, the factors considered to contribute substantially to strength phenotypes include skeletal muscle hypertrophy (muscle fibre size), hyperplasia, the predominance of fast-twitch muscle fibres, a greater muscle fascicle pennation angle, improved neurological adaptation, high glycolytic capacity, and increased circulatory testosterone [297]. Importantly, evidence exists that strength athletes exhibit vastly different transcriptomic, biochemical, anthropometric, physiological, and biomechanical characteristics compared to endurance athletes and/or controls

Extra Information

The G-allele (Ala12) of rs1801282 (Pro12Ala) in PPARG is linked to strength. Moreland, E.; et al. (2022) "Polygenic Profile of Elite Strength Athletes," Ahmetov, I.I.; et al. (2008) "PPARG Gene polymorphism and locomotor activity in humans," and Maciejewska-Karlowska, A.; et al. (2013) "Association between the Pro12Ala polymorphism of the peroxisome proliferator-activated receptor γ gene and strength athlete status" reported positive associations, possibly via effects on body composition or muscle metabolism. The reference allele is C (Pro12).

Strength

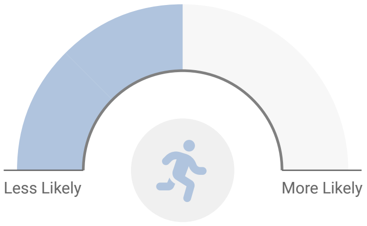

The C/C genotype in RASGRF1 (rs1521624) doesn't show significant effects on your strength traits.

• **Gene:** RASGRF1 • **rsID:** rs1521624 • **Your Genotype:** C/C • **Zygosity:** homo_ref

Description

Performance in strength-based sports is based on multiple factors. However, the factors considered to contribute substantially to strength phenotypes include skeletal muscle hypertrophy (muscle fibre size), hyperplasia, the predominance of fast-twitch muscle fibres, a greater muscle fascicle pennation angle, improved neurological adaptation, high glycolytic capacity, and increased circulatory testosterone [297]. Importantly, evidence exists that strength athletes exhibit vastly different transcriptomic, biochemical, anthropometric, physiological, and biomechanical characteristics compared to endurance athletes and/or controls

Extra Information

The A-allele of rs1521624 near RASGRF1 (growth signaling) is linked to strength. A GWAS by Tikkanen, E.; et al. (2018) "Biological Insights into Muscular Strength: Genetic Findings in the UK Biobank" and athlete studies by Moreland, E.; et al. (2022) "Polygenic Profile of Elite Strength Athletes" support this, possibly via RASGRF1's role in cellular signaling influencing muscle development. The reference allele is C (alternatives A,T).

Strength

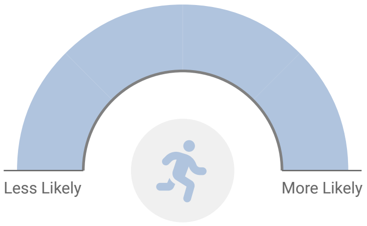

Your C/C genotype in RMC1 (rs303760) might boost your strength potential in athletic endeavors.

• **Gene:** RMC1 • **rsID:** rs303760 • **Your Genotype:** C/C • **Zygosity:** homo_ref

Description

Performance in strength-based sports is based on multiple factors. However, the factors considered to contribute substantially to strength phenotypes include skeletal muscle hypertrophy (muscle fibre size), hyperplasia, the predominance of fast-twitch muscle fibres, a greater muscle fascicle pennation angle, improved neurological adaptation, high glycolytic capacity, and increased circulatory testosterone [297]. Importantly, evidence exists that strength athletes exhibit vastly different transcriptomic, biochemical, anthropometric, physiological, and biomechanical characteristics compared to endurance athletes and/or controls

Extra Information

The C-allele of rs303760 (C1orf127) is linked to strength. A GWAS by Tikkanen, E.; et al. (2018) "Biological Insights into Muscular Strength: Genetic Findings in the UK Biobank" and athlete studies by Moreland, E.; et al. (2022) "Polygenic Profile of Elite Strength Athletes" support this as a strength marker, though the biological mechanism is unclear. The reference allele is C (alternative T).

Strength

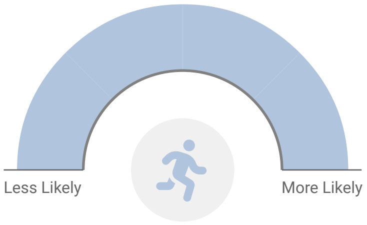

Having the A/A genotype in SLC39A8 (rs13135092) might enhance your strength, possibly aiding performance in strength sports.

• **Gene:** SLC39A8 • **rsID:** rs13135092 • **Your Genotype:** A/A • **Zygosity:** homo_ref

Description

Performance in strength-based sports is based on multiple factors. However, the factors considered to contribute substantially to strength phenotypes include skeletal muscle hypertrophy (muscle fibre size), hyperplasia, the predominance of fast-twitch muscle fibres, a greater muscle fascicle pennation angle, improved neurological adaptation, high glycolytic capacity, and increased circulatory testosterone [297]. Importantly, evidence exists that strength athletes exhibit vastly different transcriptomic, biochemical, anthropometric, physiological, and biomechanical characteristics compared to endurance athletes and/or controls

Extra Information

The A-allele of rs13135092 in SLC39A8 (manganese/zinc transporter) is linked to strength. A GWAS by Tikkanen, E.; et al. (2018) "Biological Insights into Muscular Strength: Genetic Findings in the UK Biobank" and athlete studies by Moreland, E.; et al. (2022) "Polygenic Profile of Elite Strength Athletes" support this, potentially via metal ion transport impacting muscle health. The reference allele is A (alternative G).

Strength

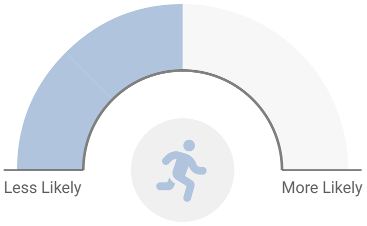

Having the G/G genotype in the TFAP2D gene doesn't appear to influence strength significantly.

• **Gene:** TFAP2D • **rsID:** rs56068671 • **Your Genotype:** G/G • **Zygosity:** homo_ref

Description

Performance in strength-based sports is based on multiple factors. However, the factors considered to contribute substantially to strength phenotypes include skeletal muscle hypertrophy (muscle fibre size), hyperplasia, the predominance of fast-twitch muscle fibres, a greater muscle fascicle pennation angle, improved neurological adaptation, high glycolytic capacity, and increased circulatory testosterone [297]. Importantly, evidence exists that strength athletes exhibit vastly different transcriptomic, biochemical, anthropometric, physiological, and biomechanical characteristics compared to endurance athletes and/or controls

Extra Information

The T-allele of rs56068671 near TFAP2D (transcription factor) is linked to strength. A GWAS by Tikkanen, E.; et al. (2018) "Biological Insights into Muscular Strength: Genetic Findings in the UK Biobank" and athlete studies by Moreland, E.; et al. (2022) "Polygenic Profile of Elite Strength Athletes" support this, suggesting it may regulate genes involved in muscle function. The reference allele is G (alternative T).

Strength

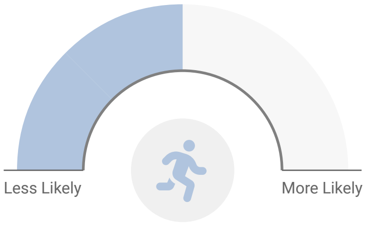

Possessing a T/T genotype hasn't shown notable correlations with strength in the context of ZKSCAN5.

• **Gene:** ZKSCAN5 • **rsID:** rs3843540 • **Your Genotype:** T/T • **Zygosity:** homo_ref

Description

Performance in strength-based sports is based on multiple factors. However, the factors considered to contribute substantially to strength phenotypes include skeletal muscle hypertrophy (muscle fibre size), hyperplasia, the predominance of fast-twitch muscle fibres, a greater muscle fascicle pennation angle, improved neurological adaptation, high glycolytic capacity, and increased circulatory testosterone [297]. Importantly, evidence exists that strength athletes exhibit vastly different transcriptomic, biochemical, anthropometric, physiological, and biomechanical characteristics compared to endurance athletes and/or controls

Extra Information

The C-allele of rs3843540 near ZKSCAN5 (transcription factor) is linked to strength. A GWAS by Tikkanen, E.; et al. (2018) "Biological Insights into Muscular Strength: Genetic Findings in the UK Biobank" and athlete studies by Moreland, E.; et al. (2022) "Polygenic Profile of Elite Strength Athletes" support this, suggesting it could regulate genes important for muscle function. The reference allele is T (alternatives C,G).

Strength

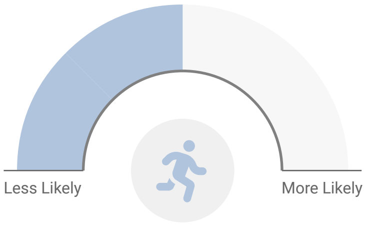

A C/C genotype doesn't display a significant relationship with strength changes for the ZNF608 gene.

• **Gene:** ZNF608 • **rsID:** rs4626333 • **Your Genotype:** C/C • **Zygosity:** homo_ref

Description

Performance in strength-based sports is based on multiple factors. However, the factors considered to contribute substantially to strength phenotypes include skeletal muscle hypertrophy (muscle fibre size), hyperplasia, the predominance of fast-twitch muscle fibres, a greater muscle fascicle pennation angle, improved neurological adaptation, high glycolytic capacity, and increased circulatory testosterone [297]. Importantly, evidence exists that strength athletes exhibit vastly different transcriptomic, biochemical, anthropometric, physiological, and biomechanical characteristics compared to endurance athletes and/or controls

Extra Information

The G-allele of rs4626333 related to ZNF608 (transcription factor) is linked to strength. Matteini, A.M.; et al. (2016) "GWAS analysis of handgrip and lower body strength in older adults in the CHARGE consortium" and Grishina, E.E.; et al. (2019) "Three DNA Polymorphisms Previously Identified as Markers for Handgrip Strength Are Associated with Strength in Weightlifters..." support this, suggesting ZNF608 may regulate genes for muscle development. The reference allele is C (alternative T).

Metabolism

Metabolic rate and nutrient processing are governed by genetic variants that influence insulin sensitivity, fat oxidation, and energy expenditure. These genetic factors can determine tendencies toward weight gain, energy levels, and responses to different dietary macronutrients. Personalized dietary strategies based on genetic information can optimize metabolic health and weight management.

Metabolic Syndrome

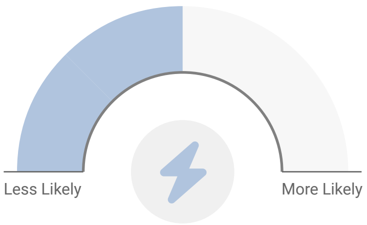

With the TT/AA genotype in the APOA5 gene, your likelihood of metabolic syndrome is average.

• **Gene:** APOA5 • **rsID:** rs662799 • **Your Genotype:** G/G>A/A • **Zygosity:** Homozygous

Description

Metabolic syndrome is a term used to describe a cluster of risk factors that elevate the likelihood of developing heart disease and other health issues, including diabetes and stroke. The term ""metabolic"" refers to the biochemical processes integral to the body's normal functioning. Risk factors encompass traits, conditions, or habits that increase the probability of disease development. In this context, ""heart disease"" primarily refers to coronary heart disease (CHD), wherein plaque, a waxy substance, accumulates inside the coronary arteries, leading to their hardening and narrowing. This process reduces blood flow to the heart muscle, potentially causing chest pain, heart attacks, cardiac damage, or even death. For more information, you can visit the National Heart, Lung, and Blood Institute; National Institutes of Health; U.S. Department of Health and Human Services website: https://www.nhlbi.nih.gov/health-topics/metabolic-syndrome.

Extra Information

rs662799 (G-A, or C-T) is a SNP in the APOA5 gene.The rarer rs662799(G or C) allele has been associated in multiple other reports to be associated with higher triglyceride levels. The rs662799 AA/TT genotype has been associated with an average likelihood of metabolic syndrome, according to a study (PMID: 23468858). In summary for other studies, The G allele is considered the minor allele and has several associations: each G allele is linked to higher triglyceride levels; the AG genotype is associated with a 1.4 times higher risk of early heart attacks and less weight gain on high-fat diets; while the GG genotype is associated with a 2 times higher risk of early heart attacks and also less weight gain on high-fat diets.

Obesity

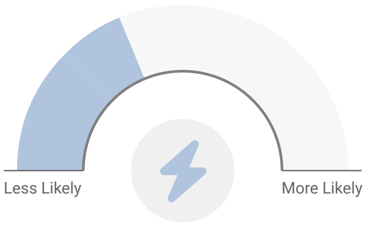

Your G/G genotype puts you at an average likelihood for obesity, but balancing diet can help.

• **Gene:** ADIPOQ • **rsID:** rs17300539 • **Your Genotype:** G/G • **Zygosity:** homo_ref

Description

Overweight and obesity have become prevalent conditions in the United States, characterized by an increase in the size and number of fat cells in the body. Medical professionals utilize measurements such as body mass index (BMI) and waist circumference to screen and diagnose these conditions. Obesity, a serious medical issue, can lead to complications like metabolic syndrome, high blood pressure, atherosclerosis, heart disease, diabetes, high blood cholesterol, cancers, and sleep disorders. Treatment approaches vary based on the cause, severity, and presence of complications, including lifestyle changes such as heart-healthy eating and increased physical activity, FDA-approved weight-loss medicines, and, in some cases, surgical interventions. (Source: National Heart, Lung, and Blood Institute; National Institutes of Health; U.S. Department of Health and Human Services.)

Extra Information

The rs17300539 GG genotype is associated with an average likelihood of obesity susceptibility, as indicated by studies (PMID: 25223469).The rs17300539(A) allele confered protection from weight regain, particularly at 32-60 weeks after the low-calorie dietary intervention, when improvement in GG subjects had disappeared [PMID 18949681].A: Increased adiponectin levels. G: Lower production of adiponectin.

Obesity

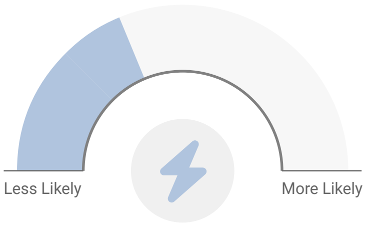

The G/G genotype seems to be associated with a normal likelihood of obesity for you.

• **Gene:** Intergenic • **rsID:** rs7138803 • **Your Genotype:** G/G • **Zygosity:** homo_ref

Description

Overweight and obesity have become prevalent conditions in the United States, characterized by an increase in the size and number of fat cells in the body. Medical professionals utilize measurements such as body mass index (BMI) and waist circumference to screen and diagnose these conditions. Obesity, a serious medical issue, can lead to complications like metabolic syndrome, high blood pressure, atherosclerosis, heart disease, diabetes, high blood cholesterol, cancers, and sleep disorders. Treatment approaches vary based on the cause, severity, and presence of complications, including lifestyle changes such as heart-healthy eating and increased physical activity, FDA-approved weight-loss medicines, and, in some cases, surgical interventions. (Source: National Heart, Lung, and Blood Institute; National Institutes of Health; U.S. Department of Health and Human Services.)

Extra Information

The rs7138803 AG genotype has been linked to an increased likelihood of obesity, as reported in studies (PMID(s): 21912638, 21527513). The risk allele A is asoosciated with body mass index[PMID 20935630].The GG genotype seems have anormal likelihood of obesity.

Obesity

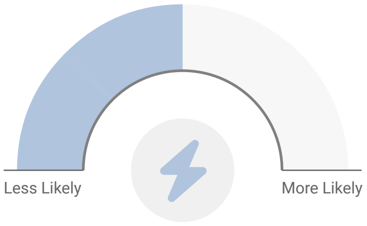

Having the G/G genotype indicates an average likelihood for obesity according to current studies.

• **Gene:** Intergenic • **rsID:** rs12970134 • **Your Genotype:** G/G • **Zygosity:** homo_ref

Description

Overweight and obesity have become prevalent conditions in the United States, characterized by an increase in the size and number of fat cells in the body. Medical professionals utilize measurements such as body mass index (BMI) and waist circumference to screen and diagnose these conditions. Obesity, a serious medical issue, can lead to complications like metabolic syndrome, high blood pressure, atherosclerosis, heart disease, diabetes, high blood cholesterol, cancers, and sleep disorders. Treatment approaches vary based on the cause, severity, and presence of complications, including lifestyle changes such as heart-healthy eating and increased physical activity, FDA-approved weight-loss medicines, and, in some cases, surgical interventions. (Source: National Heart, Lung, and Blood Institute; National Institutes of Health; U.S. Department of Health and Human Services.)

Extra Information

The rs12970134 GG genotype is associated with an average likelihood of obesity and GA/AA is associated with higher risk of obesity, as indicated by studies (PMID(s): 18454146, 23049848). The risk allele A is asoociated with high BMI in obese women(PMID: 31429705). A study of 14,000 Indian Asian or Caucasian adults indicates that rs12970134(A) alleles are associated with obesity. The average increase in waist circumference is 0.9cm BMI units per risk allele. [PMID 18454146]

Resting Metabolic Rate

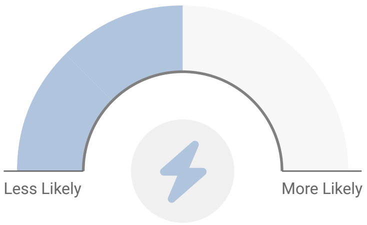

The G/G genotype suggests a typical resting metabolic rate and reduced weight gain potential with certain antipsychotic medications.

• **Gene:** LEPR • **rsID:** rs8179183 • **Your Genotype:** G/G • **Zygosity:** homo_ref

Description

Resting (basal) metabolic rate is a measure that quantifies the energy expended in performing and sustaining basic autonomic functions, including pupillary response, respiration, and heartbeat. These essential functions are typically carried out involuntarily and contribute to the body's overall energy expenditure.

Extra Information

The rs8179183 CG/GG genotype is linked to the usual resting metabolic rate (PMID: 16231024).rs8179183 is a SNP in the leptin receptor LEPR gene.In a study of 101 mostly Caucasian patients prescribed the atypical antipsychotic risperidone, carriers of a rs8179183(G) allele were less likely to gain significant weight compared to rs8179183 CC genotype carriers, as assessed by physiogenomic analysis of corresponding weight profiles. Two other SNPs, rs6837793 and rs705381, were also significantly associated with weight profiles in these patients.[PMID 17199131]

Weight Gain

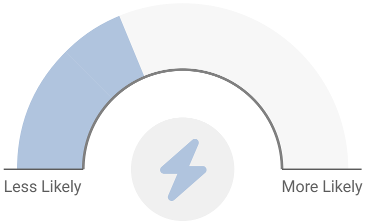

With a C/C genotype, you are likely at an average risk for weight gain.

• **Gene:** PPARG • **rsID:** rs1801282 • **Your Genotype:** C/C • **Zygosity:** homo_ref

Description

The ease with which one gains or loses weight is influenced by many factors, such as physical activity and diet. Other biological factors also influence the rate at which one gains or loses weight, including age, sex, and specific genetic variants.

Extra Information

The rs1801282 CC genotype is associated with an average risk of weight gain (PMID(s): 23666678, 26361038).In comparison, the minor G allele is associated with reduced weight gain. rs1801282, also known as Pro12Ala, is a common SNP in the peroxisome proliferator-activated receptor PPARG gene. The more common (C) allele (in dbSNP orientation) encodes the 'Pro' amino acid at this SNP position. rs1801282 G allele has been also reported to be associated with:lower PPAR gamma activity,more benefits to exercise(having one G allele makes you more responsive to the beneficial health effects of exercise),increased obesity rates,lower heart disease,lower risk for diabetes,poor response to a high-fat diet,protective against colorectal cancer,protective against psoriatic arthritis.higher risk for rheumatoid arthritishigher risk for sarcoidosis.

Behavior

Behavioral tendencies, such as impulsivity, risk tolerance, and social interaction styles, are shaped by genetic factors affecting neurotransmitter pathways and receptor sensitivities. These genetic influences can affect decision-making patterns, stress responses, and even susceptibility to behavioral conditions. Understanding these factors allows for tailored behavioral interventions and stress management techniques.

Hunger Response Control

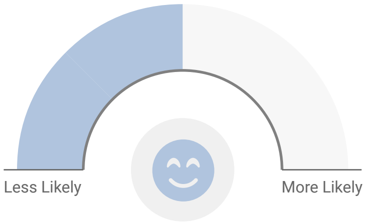

With the G/T genotype, you might experience average dietary disinhibition and have a usual hunger response.

• **Gene:** NMB • **rsID:** rs1051168 • **Your Genotype:** G/G>G/T • **Zygosity:** Heterozygous

Description

Hunger is characterized by a sense of physical discomfort and potentially mental anxiety when food is not present, coupled with a desire to eat. The body's capacity to regulate the sensation of hunger, known as hunger response control, is influenced by factors such as diet and lifestyle. Additionally, specific genetic variants can also contribute to shaping this aspect of hunger regulation.

Extra Information

The rs1051168, a missense mutation (G to T, p.P73T) that located in the neuromedin-_ gene is associated with higher levels of disinhibition, susceptibility to hunger, and body weight gain over time(PMID:21527296). Also it has been earlier associated with obesity and abnormal eating behaviour in adults(PMID:20010906). TG/GG(AC/CC) genotype has been linked to an average likelihood of dietary disinhibition and hunger susceptibility, according to studies (PMID(s): 18271693, 15585758, Arguello 2018).and TT/AA polymorphism has been associated with eating behaviors and increased risk of obesity.

Snacking Behavior

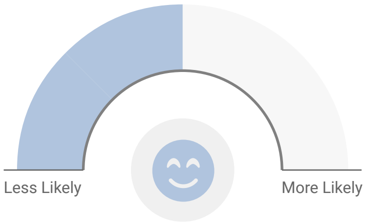

There's no substantial evidence linking the A/A genotype with specific snacking behaviors.

• **Gene:** LEPR • **rsID:** rs2025804 • **Your Genotype:** G/G>A/A • **Zygosity:** Homozygous

Description

Snacking is generally defined as the behavior of eating a small portion of food in between regular meals.

Extra Information

The rs2025804 AG genotype is linked to the usual snacking eating pattern and an average BMI (PMID(s): 17192493, 17903300, 22810975).Scientists established that people with two copies of the G allele (GG genotype) at position rs2025804 are twice as likely to show 'extreme snacking behaviour' (which is defined as consuming more than 15% of their energy intake as snacks in between meals).Common Genetic Variations in CCK, Leptin, and Leptin Receptor Genes Are Associated With Specific Human Eating Patterns.

Sugar Intake

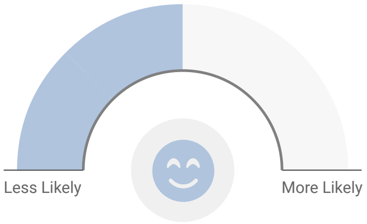

Your G/G genotype likely correlates with an average sugar intake and usual dental health.

• **Gene:** SLC2A2 • **rsID:** rs5400 • **Your Genotype:** G/G • **Zygosity:** homo_ref

Description

Genetic factors play a role in the variation of daily sugar intake levels among individuals, influenced by environmental factors such as the availability of sugars and dietary choices. Recent studies have identified genetic associations with differences in sugar consumption patterns.

Extra Information

The rs5400 CC(GG) genotype is linked to average sugar intake and typical susceptibility to developing dental cavities, according to studies (PMID(s): 18349384, 26112465).The CT(GA) and TT(AA) genotype is associated with significantly higher sugar consumption.

Sweet Tooth

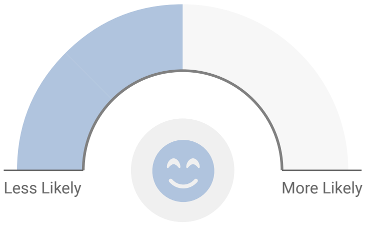

G/G genotype doesn’t show notable impact on your preference for sweet foods.

• **Gene:** FUT1 • **rsID:** rs838133 • **Your Genotype:** A/A>G/G • **Zygosity:** Homozygous

Description

Some individuals greatly enjoy the taste of sweets, such as candy, frosting, cookies, and ice cream. Others express preferences for various non-sweet flavors. Certain genetic variants are associated with sweet taste preference.

Extra Information

The rs838133 TT genotype is associated with a higher likelihood of preferring sweet food (PMID(s): 23372041, 28467924)*.A 2017 study of ~6,500 individuals with detailed eating records concluded that the rs838133(T) allele (in SNPedia and dbSNP orientation) increased the odds ratio (OR) of being in the top 33% of people for total intake of all types of sweet-tasting foods, with an OR of 1.18 per T-allele (CI: 1.06?.32, p = 0.003). When sweet intake was divided into ?andy?and ?ake,?individuals carrying the T-allele had higher candy intake (OR 1.19, CI: 1.07?.32, p = 0.0007), whereas intake of cake was the same regardless of rs838133 genotype.In comparison, CC genotype is associated with lower odds of preferring candy or sweet snacks, and CT represents sweeth tooth tendency and slightly higher odds of preferring candy.

Name:
DoB:
Gender:
Report ID: WBWG_01_P001_262
Patient ID: 01_P001_262
Date: 6/7/2025

Nutrition and Diet

Genetic variations can influence how nutrients are metabolized, impacting dietary preferences, nutrient deficiencies, and even food sensitivities. For instance, polymorphisms in genes related to lactose tolerance, gluten sensitivity, or omega-3 metabolism can determine the optimal diet for an individual. Nutrigenomics enables personalized nutrition plans for improved health outcomes.

Alcohol Flush

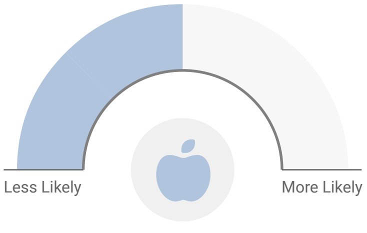

With the G/G genotype, you likely have a typical reaction to alcohol without the flush.

• **Gene:** ALDH2 • **rsID:** rs671 • **Your Genotype:** G/G • **Zygosity:** homo_ref

Description

Alcohol sensitivity, also recognized as alcohol flush reaction or alcohol intolerance, is associated with the body's capacity to metabolize alcohol. This response commonly manifests as facial flushing (reddening of the face), heart palpitations (rapid, irregular, or fluttering heartbeat), lightheadedness, and nausea. These symptoms arise mainly from the accumulation of acetaldehyde, a byproduct formed when the enzyme aldehyde dehydrogenase fails to convert ethanol (alcohol) into acetate at an adequate pace. There are identifiable genetic variations that play a role in influencing alcohol sensitivity.

Extra Information

AA: Commonly referred to as ""Asian Flushers,"" individuals with this genotype have an increased risk of esophageal cancer, particularly those of East Asian ancestry. Disulfiram, a medication used to treat alcoholism, is usually not effective for individuals with this genotype.AG: Individuals with this genotype also experience the Asian Flush phenomenon and may have worse hangovers. They share an increased risk of esophageal cancer, especially among those of East Asian ancestry. Disulfiram is also likely ineffective for treating alcoholism in individuals with this genotype.GG: Individuals with this genotype do not typically experience the alcohol flush reaction. They have normal hangovers and are at average risk for alcoholism and esophageal cancer. Disulfiram is considered effective for treating alcoholism in individuals with this genotype.

Caffeine Metabolism

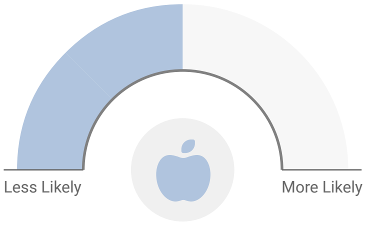

A/C genotype means you might process caffeine slightly faster than some, so enjoy that extra energy boost.

• **Gene:** CYP1A2 • **rsID:** rs762551 • **Your Genotype:** C/C>C/A • **Zygosity:** Heterozygous

Description

Caffeine, a bitter substance, naturally occurs in over 60 plants, including coffee beans, tea leaves, kola nuts (used to flavor colas), and cacao pods (utilized in chocolate production). Synthetic caffeine is also artificially created and added to certain medicines, foods, and beverages. Examples include pain relievers, cold medicines, over-the-counter alertness medications, energy drinks, as well as energy-boosting gums and snacks.The primary sources of caffeine consumption are beverages, with varying caffeine content in different drinks. Generally, the caffeine amounts are as follows: 8-ounce cup of coffee = 95-200 mg, 12-ounce can of cola = 35-45 mg, 8-ounce energy drink = 70-100 mg, 8-ounce cup of tea = 14-60 mg. Caffeine affects the body's metabolism in various ways; it stimulates the central nervous system, providing a feeling of wakefulness and energy boost. Caffeine acts as a diuretic, promoting the elimination of excess salt and water through increased urination. It also enhances stomach acid release, potentially causing upset stomach or heartburn. Moreover, caffeine may impact calcium absorption and elevate blood pressure.Upon consumption, caffeine reaches its peak level in the bloodstream within one hour, with effects lasting for four to six hours. For more detailed information, you can visit https://medlineplus.gov/caffeine.html#summary.

Extra Information

A meta-analysis conducted by Denden et al. showed that the rs762551 AA genotype is associated with higher coffee intake and faster caffeine clearance than CC and CA genotype. CA genotype is associated with a slightly faster rate of caffeine metabolism than CC genptype (PMID(s): 29282363, 20390257,27173183).

Caffeine Metabolism

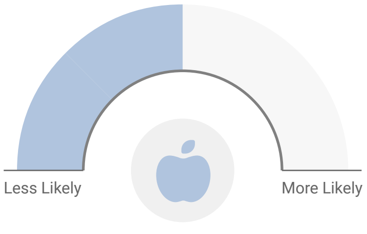

For your Caffeine Metabolism trait with the Near CYP1A2 gene (rs2472297), having the C/C genotype is linked to average coffee consumption.

• **Gene:** Near CYP1A2 • **rsID:** rs2472297 • **Your Genotype:** C/C • **Zygosity:** homo_ref

Description

Caffeine, a bitter substance, naturally occurs in over 60 plants, including coffee beans, tea leaves, kola nuts (used to flavor colas), and cacao pods (utilized in chocolate production). Synthetic caffeine is also artificially created and added to certain medicines, foods, and beverages. Examples include pain relievers, cold medicines, over-the-counter alertness medications, energy drinks, as well as energy-boosting gums and snacks.The primary sources of caffeine consumption are beverages, with varying caffeine content in different drinks. Generally, the caffeine amounts are as follows: 8-ounce cup of coffee = 95-200 mg, 12-ounce can of cola = 35-45 mg, 8-ounce energy drink = 70-100 mg, 8-ounce cup of tea = 14-60 mg. Caffeine affects the body's metabolism in various ways; it stimulates the central nervous system, providing a feeling of wakefulness and energy boost. Caffeine acts as a diuretic, promoting the elimination of excess salt and water through increased urination. It also enhances stomach acid release, potentially causing upset stomach or heartburn. Moreover, caffeine may impact calcium absorption and elevate blood pressure.Upon consumption, caffeine reaches its peak level in the bloodstream within one hour, with effects lasting for four to six hours. For more detailed information, you can visit https://medlineplus.gov/caffeine.html#summary.

Extra Information

The rs2472297 CC genotype has been associated with average daily coffee consumption, as indicated by studies (PMID(s): 21357676, 21876539, 25288136).A meta-analysis of four GWAS studies of coffee consumption among a total of ~8,000 coffee drinkers of European ancestry found that the?s2472297(T) was strongly associated with increased consumption (p = 5.4 x 10e-14). The estimated effect was an increase of 0.2 cups a day per allele for both this SNP and one other (rs6968865).[PMID 21357676]. CYP1A2 contains instructions for an enzyme that breaks down caffeine.CT-Associated with (slightly) increased coffee consumption.TT-Associated with (slightly) increased coffee consumption.

Name: | Report ID: WBWG_01_P001_262
DoB: | Patient ID: 01_P001_262
Gender: | Date: 6/7/2025

Caffeine Metabolism

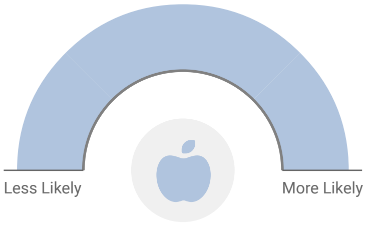

For your Caffeine Metabolism trait with the Near AHR gene (rs4410790), the T/T genotype indicates you might enjoy more coffee daily.

- **Gene:** Near AHR
- **rsID:** rs4410790
- **Your Genotype:** T/T
- **Zygosity:** homo_ref

Description

Caffeine, a bitter substance, naturally occurs in over 60 plants, including coffee beans, tea leaves, kola nuts (used to flavor colas), and cacao pods (utilized in chocolate production). Synthetic caffeine is also artificially created and added to certain medicines, foods, and beverages. Examples include pain relievers, cold medicines, over-the-counter alertness medications, energy drinks, as well as energy-boosting gums and snacks.The primary sources of caffeine consumption are beverages, with varying caffeine content in different drinks. Generally, the caffeine amounts are as follows: 8-ounce cup of coffee = 95-200 mg, 12-ounce can of cola = 35-45 mg, 8-ounce energy drink = 70-100 mg, 8-ounce cup of tea = 14-60 mg. Caffeine affects the body's metabolism in various ways; it stimulates the central nervous system, providing a feeling of wakefulness and energy boost. Caffeine acts as a diuretic, promoting the elimination of excess salt and water through increased urination. It also enhances stomach acid release, potentially causing upset stomach or heartburn. Moreover, caffeine may impact calcium absorption and elevate blood pressure.Upon consumption, caffeine reaches its peak level in the bloodstream within one hour, with effects lasting for four to six hours. For more detailed information, you can visit https://medlineplus.gov/caffeine.html#summary.

Extra Information

The C allele is linked to lower levels of caffeine intake. Individuals with the TT genotype tend to consume about one-third more coffee per day compared to those with the CT or CC genotypes. A genome-wide meta-analysis has pinpointed regions on chromosomes 7p21 (AHR) and 15q24 (CYP1A2) as key determinants of habitual caffeine consumption. The AHR protein enhances the production of enzymes CYP1A1 and CYP1A2, responsible for metabolizing caffeine. This finding is supported by research indicating associations between genetic variations in the AHR and CYP1A1-CYP1A2 gene regions and habitual caffeine consumption.

Lactose Intolerance

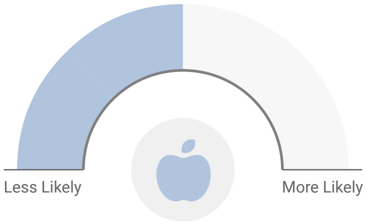

For lactose intolerance, with the MCM6 gene (rs4988235), your G/G genotype doesn't show notable effects.

- **Gene:** MCM6
- **rsID:** rs4988235
- **Your Genotype:** G/G
- **Zygosity:** homo_ref

Description

Lactose intolerance refers to an impaired ability to digest lactose, a sugar present in milk and other dairy products. In infancy, lactose is typically broken down by an enzyme called lactase, produced by cells in the lining of the small intestine. However, the production of this enzyme ceases during or after weaning, and most adult mammals, including humans, become unable to efficiently digest lactose. Primary lactase deficiency, also known as lactase nonpersistence, is the most prevalent type of lactase deficiency.

Extra Information

The rs4988235 CC genotype is associated with lactose intolerance in adults.Individuals with the CC genotype at the rs4988235 SNP are typically unable to continue producing lactase into adulthood, leading to lactose intolerance. This intolerance manifests as gastrointestinal symptoms such as bloating, diarrhea, and abdominal pain following the consumption of dairy products due to the undigested lactose fermenting in the gut.On the other hand, individuals with the CT or TT genotypes are more likely to continue producing lactase throughout their lives, allowing them to digest milk and other dairy products without adverse effects. The TT genotype is associated with a higher likelihood of being able to digest milk in adulthood compared to the CT genotype.

Polyunsaturated Fats

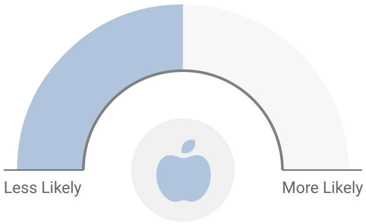

The T/T genotype in your trait related to polyunsaturated fats doesn't show any significant effects.

- **Gene:** FADS1
- **rsID:** rs174547
- **Your Genotype:** T/T
- **Zygosity:** homo_ref

Description

This type of fat, known as polyunsaturated fatty acids (PUFAs), remains in a liquid state at room temperature. Two main categories of PUFAs are omega-6 and omega-3. Omega-6 fatty acids are present in liquid vegetable oils like corn oil, safflower oil, and soybean oil. On the other hand, omega-3 fatty acids are derived from plant sources such as canola oil, flaxseed, soybean oil, and walnuts, as well as from seafood, including fatty fish (e.g., salmon, tuna, trout) and shellfish (e.g., crab, mussels, oysters). Specific omega-3 fatty acids like EPA and DHA are abundant in seafood, while another type, ALA (alpha-linolenic acid), is found in various foods, including some vegetable oils like canola and soy. Omega-3s are also available in dietary supplements, with fish oil supplements containing EPA and DHA, and flaxseed oil supplements containing ALA. While there is moderate evidence supporting the health benefits of consuming seafood, the health advantages of omega-3 dietary supplements remain unclear (Source: National Center for Complementary and Integrative Health; National Institutes of Health; U.S. Department of Health and Human Services). For additional information, you can visit https://ods.od.nih.gov/factsheets/Omega3FattyAcids-HealthProfessional/.

Extra Information

The genomic variant c.1248+52 A to G , or T to C, also known as rs174547. Studies involving individuals of European descent indicate that those with the rs174547 homozygous mutated GG/CC genotype exhibit lower levels of polyunsaturated fatty acid(PMID: 24823311) and also for CT genotype as the risk allele carrier.The "C" allele was associated with lower LDL (low density lipoprotein) concentration (P = 0.03) [PMID: 22451038].This rs174547 C minor allele was associated with a higher proportion of linoleic acid, lower arachidonic acid and docosahexaenoic acid, as well as lower delta-6-desaturase and delta-5-desaturase activity. Female C allele carriers had lower android fat percentages and lower levels of low-density lipoprotein-cholesterol, while male C allele carriers had lower gynoid fat percentages and higher triglyceride after adjusting for age, income, BMI, behavioral risk factors, and regional fat percentages(PMID: 28359317).

Saturated Fat and Weight

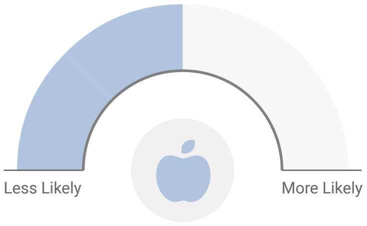

There's no strong evidence showing a notable effect on your weight regarding saturated fat intake with the A/A genotype near APOA2.

• **Gene:** Near APOA2 • **rsID:** rs5082 • **Your Genotype:** G/G>A/A • **Zygosity:** Homozygous

Description

Saturated fat is a type of?ietary fat. It is one of the unhealthy fats, along with?rans fat. These fats are most often solid at room temperature. Foods like butter, palm and coconut oils, cheese, and red meat have high amounts of saturated fat.Weight gain. Many high-fat foods such as pizza, baked goods, and fried foods have a lot of saturated fat. Eating too much fat can add extra calories to your diet and cause you to gain weight. All fats contain 9 calories per gram of fat. This is more than twice the amount found in carbohydrates and protein.

Extra Information

rs5082 is a SNP in the apolipoprotein APOA2 gene, and may influence obesity and heart disease risk.Individuals homozygous for the -265T>C polymorphism in rs5082, i.e. individuals with the rs5082(CC) genotype of the Apolipoprotein A-II gene (APOA2) promoter, are associated with increased Body Mass Index and food intake in the Genetics of Lipid Lowering Drugs and Diet Network (GOLDN) Study of ~1000 individuals. The odds ratio for obesity in (CC) individuals compared to rs5082(T) allele carriers was 1.70 (CI: 1.02-2.80, p=0.039). Total energy, total fat, and total protein intake were all significantly higher in (CC) individuals.[PMID 17446329].CC genotype is associated with saturated fat contributes to obesity, but 0.57 % lower risk for coronary artery disease.

Vitamin B12

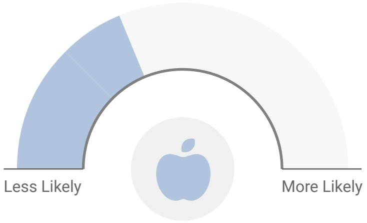

The G/G genotype suggests lower levels of vitamin B12, which might affect your energy levels.

• **Gene:** FUT2 • **rsID:** rs602662 • **Your Genotype:** G/G • **Zygosity:** homo_ref

Description

Vitamin B12 (cobalamin) is a compound crucial for various metabolic processes in the body. It naturally occurs in animal products like meat, organs, and dairy. Additionally, lower levels of vitamin B12 are present in fortified plant-based foods and grains, as well as in vitamin supplements. Inadequate levels of vitamin B12 can lead to conditions such as anemia, fatigue, constipation, and peripheral neuropathy, affecting nerve sensation in the hands and feet. Specific genetic variants have been identified to influence the circulating levels of vitamin B12 in the bloodstream.

Extra Information

The rs602662 AG genotype is linked to reduced vitamin B12 levels (PMID(s): 18776911, 27995393)*.In comparison, AA represents Higher vitamin B12 levels, GG represents Lower vitamin B12 levels.

Vitamin B6

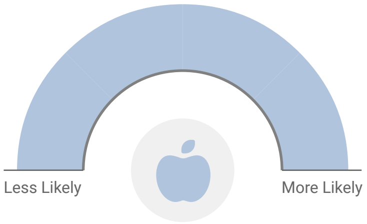

With the C/C genotype, you might have lower levels of vitamin B6, which could influence your health.

• **Gene:** NBPF3 • **rsID:** rs4654748 • **Your Genotype:** C/C • **Zygosity:** homo_ref

Description

Vitamin B6 is composed of six compounds that play crucial roles in various metabolic processes in the body, including the production of neurotransmitters used for nerve communication and other vital biological functions. It is naturally present in meats, animal organs (such as beef liver), starchy vegetables, and non-citrus fruits. Additionally, vitamin B6 can be found in lower amounts in fortified plant-based foods, grains, and vitamin supplements. Inadequate levels of vitamin B6 may lead to anemia, skin issues, and a weakened immune system. Specific gene variants have been identified as influencers of the circulating levels of vitamin B6 in the bloodstream.

Extra Information

The rs4654748 CT and CC genotype is linked to reduced serum levels of vitamin B6 (PMID: 19303062), especially CC geotype on a biger extent.TT genptype is more likely to have normal levels of serum Vitamin B6.

Vitamin C

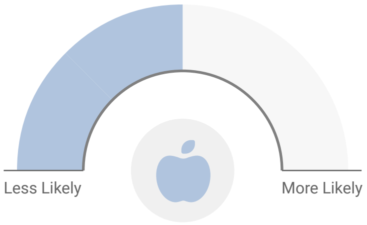

With C/C, Vitamin C levels aren't clearly impacted in any direction, so keep munching on those veggies!

• **Gene:** SLC23A1 • **rsID:** rs33972313 • **Your Genotype:** C/C • **Zygosity:** homo_ref

Description

Vitamin C (L-ascorbic acid) is a compound utilized in various biological processes, including the synthesis of collagen (a skin component), immune system function, and acting as an antioxidant. Antioxidants like vitamin C help eliminate free radicals and reactive oxygen species that could harm cells. Natural sources of vitamin C include citrus fruits, vegetables, and fortified foods, and it is also available in vitamin supplements. Severe vitamin C deficiency may lead to scurvy, characterized by symptoms such as fatigue, anemia, skin issues, and immune system deficiencies. Specific genetic variations have been identified as influencing the levels of circulating vitamin C in the bloodstream.

Extra Information

The rs33972313 GG genotype is associated with typical or higher serum levels of vitamin C (PMID(s): 20519558, 23737080,25948669).The GA and AA genptype is more likely to have lower plasma vitamin C levels.

Vitamin D

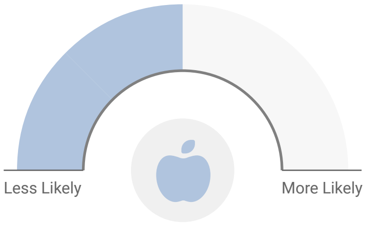

T/T doesn't show a particular influence on Vitamin D levels, so your regular routine should be just fine.

• **Gene:** GC • **rsID:** rs2282679 • **Your Genotype:** T/T • **Zygosity:** homo_ref

Description

Vitamin D plays multiple roles in the body and is technically classified as a hormone rather than a vitamin. It facilitates the absorption of calcium, a crucial component for bone formation. Inadequate levels of vitamin D can contribute to bone disorders like osteoporosis or rickets. Additionally, vitamin D is involved in the proper functioning of the nervous, muscular, and immune systems.

Extra Information

The rs2282679 AA genotype is linked to typical vitamin D levels (PMID(s): 20541252, 23924835, 23191998, 25174667).rs2282679, located in the group-specific component (vitamin D binding protein) GC gene on chromosome 4p12, has been linked by several studies to vitamin D serum concentrations.In both studies, the allele associated with lower vitamin D, and thus the potential for vitamin D insufficiency, is rs2282679(C). Carriers of two such alleles have lower vitamin D than carriers of one allele, who in turn on average have lower vitamin D levels than rs2282679(AA) individuals.In comparision, CC genotype represents lower vitamin D levels.

Vitamin E

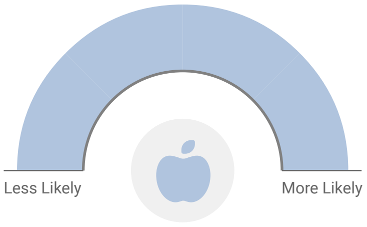

You have the C/C genotype, which aligns with typical vitamin E levels. Keep on indulging in those nutritious seeds and nuts!

• **Gene:** Intergenic • **rsID:** rs12272004 • **Your Genotype:** C/C • **Zygosity:** homo_ref

Description

Vitamin E is an antioxidant that contributes to immune system function and metabolic processes. It can be obtained from various sources, including vegetable oils, nuts, seeds, leafy greens like spinach, and fortified foods or drinks. In most cases, people receive sufficient vitamin E from their diet, and supplements are usually unnecessary. However, individuals with specific conditions like cystic fibrosis, liver diseases, or Crohn's disease might require additional vitamin E.

Extra Information

The rs12272004 CC genotype is associated with typical vitamin E levels (PMID(s): 19185284, 21729881).The A allele of rs12272004 was associated with 0.07 SD higher _-tocopherol (a type of vitamin E), with a p value of 7.8 _ 10_10.

Name:
DoB:
Gender:
Report ID: WBWG_01_P001_262
Patient ID: 01_P001_262
Date: 6/7/2025

Sensory Perception

The way we perceive sensory inputs such as taste, smell, and sound is influenced by genetic variants that affect receptor function and neural processing. These variations can alter taste preferences, odor sensitivities, and even auditory perception. Understanding these genetic traits can explain individual differences in sensory experiences and preferences.

Asparagus Odor Detection

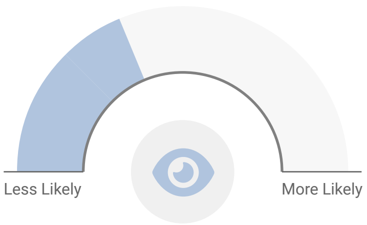

As someone with the G/G genotype, you might struggle to detect the unmistakable asparagus scent in urine.

• **Gene:** Near OR2M7 • **rsID:** rs4481887 • **Your Genotype:** A/A>G/G • **Zygosity:** Homozygous

Description

Asparagus contains a unique compound called asparagusic acid, believed to be responsible for the distinctive scent in urine after its consumption, along with its sulfur-containing metabolites. However, not everyone can perceive this odor. Research indicates variations among individuals in the production of these odorous compounds and their ability to detect the scent. Anosmia, the loss of the sense of smell, is often referred to as 'asparagus anosmia' when describing the inability to detect these compounds in urine. Genetic changes have been identified as factors linked to the ability to perceive the scent of asparagus urine.

Extra Information

Studies involving people of European ancestry suggest that individuals with the rs4481887 AG genotype are more adept at detecting the scent of asparagus in urine, reducing the chances of experiencing asparagus anosmia (PMID(s): 20585627, 20876394, 27965198). Those with the AA genotype are the most likely to detect the scent of asparagus metabolites in urine, while those with the GG genotype are the least likely to perceive it.

Cilantro (Coriander) Preference

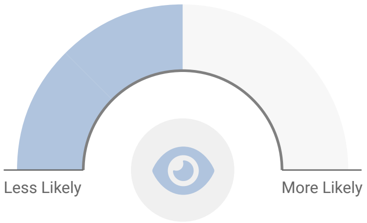

We don't see a strong genetic influence on cilantro taste perception for the C/T genotype.

• **Gene:** Intergenic • **rsID:** rs3930459 • **Your Genotype:** T/T>T/C • **Zygosity:** Heterozygous

Description

Coriandrum sativum is the plant responsible for producing both cilantro and coriander. Cilantro is typically harvested during the initial growth cycle before flowering, while coriander is harvested after the plant has blossomed and developed seeds. While some people enjoy the taste of cilantro, incorporating it into dishes like rice, burritos, and guacamole, others strongly dislike its flavor and aroma, often describing it as ""soapy."" Research has identified specific genetic variants associated with cilantro preference.

Extra Information

The rs3930459 TT genotype has been associated with an average likelihood of disliking the taste of cilantro, as indicated in the study by Fayzullina in 2015*.C allele (CC genotype) associated with slightly higher odds of disliking cilantro.

Misophonia

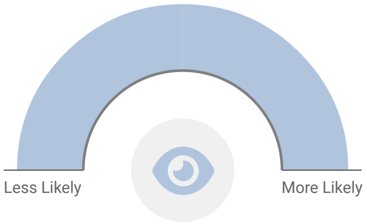

With your A/G genotype, you might have a heightened sensitivity to chewing sounds (misophonia).

• **Gene:** Intergenic • **rsID:** rs2937573 • **Your Genotype:** G/G>G/A • **Zygosity:** Heterozygous

Description

Misophonia is a term used to describe a sensitivity to certain sounds, combining the Greek words 'misein' (to hate) and 'phonos' (voice or sound). Individuals with this condition may struggle to tolerate specific sounds like keyboard typing, pen clicking, or breathing. Notably, misophonia related to the sound of chewing has been found to be associated with a specific genetic variant.

Extra Information

The rs2937573 AG genotype has been associated with an increased likelihood of being sensitive to the sound of chewing, known as misophonia, according to a study (Fayzullina 2015).Other genotypes seems showing typical likelihood of the sound.

Name:
DoB:
Gender:
Report ID: WBWG_01_P001_262
Patient ID: 01_P001_262
Date: 6/7/2025

Nearsightedness(myopia)

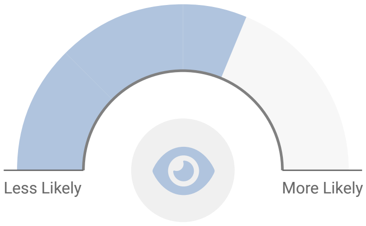

The A/G genotype indicates a reduced risk compared to A/A for nearsightedness.

• **Gene:** Intergenic • **rsID:** rs560766 • **Your Genotype:** G/G>G/A • **Zygosity:** Heterozygous

Description

Nearsightedness, also known as myopia or short-sightedness, is a condition characterized by a mismatch between the focusing power of the eye and the length of the eye. In nearsightedness, light rays are focused in front of the retina rather than directly on it. Individuals with nearsightedness may experience difficulty seeing objects that are far away, while maintaining the ability to focus on close objects.

Extra Information

The rs560766 AA genotype has been associated with increased odds of being nearsighted, according to studies (PMID(s): 20835239, 23131718, 22665138)*,compared to AG or GG genotype.

Nearsightedness(myopia)

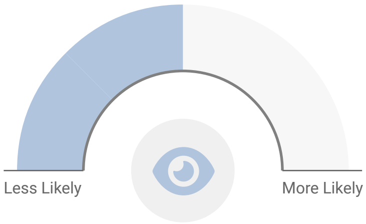

Holding the C/T genotype throughout rs10034228 doesn't convey prominent myopia tendencies.

• **Gene:** Intergenic • **rsID:** rs10034228 • **Your Genotype:** T/T>T/C • **Zygosity:** Heterozygous

Description

Nearsightedness, also known as myopia or short-sightedness, is a condition characterized by a mismatch between the focusing power of the eye and the length of the eye. In nearsightedness, light rays are focused in front of the retina rather than directly on it. Individuals with nearsightedness may experience difficulty seeing objects that are far away, while maintaining the ability to focus on close objects.

Extra Information

The rs10034228 TT genotype has been found to be associated with a typical risk of high-grade myopia in the Chinese population, as reported in studies (PMID(s): 21505071, 22150588).Specifically, the T allele at this locus was found to have a protective effect against high myopia. Individuals carrying the C/C genotype at rs10034228 were found to have a higher risk of high myopia compared to those with the T allele.

Odor detection

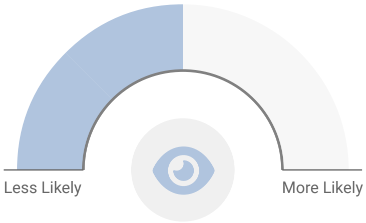

With C/C, you might have heightened sensitivity to specific odors like isovaleric acid.

• **Gene:** Intergenic • **rsID:** rs1953558 • **Your Genotype:** T/T>C/C • **Zygosity:** Homozygous

Description

The ability to smell is attributed to olfactory receptors located in the nasal cavity. These receptors interact with airborne molecules, and the olfactory nerves connected to them transmit signals to the brain, which interprets the scent. While each individual has a unique perception of scents, genetic factors play a role in determining the ability to detect specific molecules.

Extra Information

The rs1953558 single nucleotide polymorphism (SNP) is located within the human OR11H7 gene, which encodes for an olfactory receptor. This receptor is involved in the sense of smell and can influence an individual's sensitivity to specific odors. Specifically, the rs1953558 SNP has been associated with the perception of isovaleric acid, a chemical compound commonly associated with the smell of sweat, particularly the odor of feet.According to the search results provided, individuals with the TT genotype at the rs1953558 locus exhibit a slightly reduced sensitivity to the smell of isovaleric acid. This means that these individuals may require a higher concentration of isovaleric acid to detect the odor compared to those with other genotypes. In contrast, individuals with the TC or CC genotypes may have a heightened sensitivity to this odorant.The variation in olfactory sensitivity linked to the rs1953558 SNP is part of a broader pattern of genetic diversity in human olfaction. It is estimated that there is about a 30% difference in olfactory receptors among individuals, which can explain why some people can detect certain smells while others cannot. (PMID: 17973576)*.

Name:
DoB:
Gender:
Report ID: WBWG_01_P001_262
Patient ID: 01_P001_262
Date: 6/7/2025

Pain Sensitivity

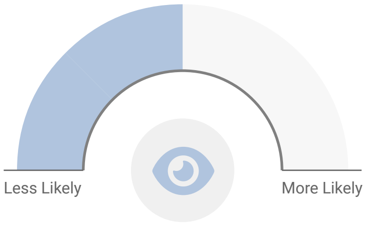

Your A/G genotype may give you moderate pain sensitivity, falling in between the other genotypes.

• **Gene:** COMT • **rsID:** rs6269 • **Your Genotype:** A/A>A/G • **Zygosity:** Heterozygous

Description

Sensitivity to pain varies among individuals, and genetic differences contribute to this variation.

Extra Information

The rs6269 AA genotype is associated with typical pain sensitivity (PMID(s): 19094200, 24533707)*.The SNP rs6269 accounted for 6% of the variation in pain sensitivity as determined by analysis of variance (ANOVA, P <0.01).In the context of pain sensitivity, individuals with the GG genotype at the rs6269 locus (homozygous for the Val allele) may exhibit increased pain sensitivity compared to those with the AG or AA genotypes. This is because the higher COMT activity associated with the GG genotype may result in reduced levels of pain-modulating neurotransmitters, such as dopamine, in brain regions involved in pain processing.

Photic Sneeze Reflex

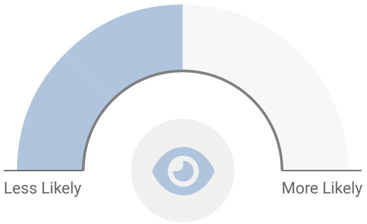

If you have the T/T genotype, your odds of experiencing a photic sneeze reflex are typical.

• **Gene:** Intergenic • **rsID:** rs10427255 • **Your Genotype:** C/C>T/T • **Zygosity:** Homozygous

Description

Genetic variants influence the occurrence of the photic sneeze reflex, a phenomenon where an individual is compelled to sneeze upon sudden exposure to bright light, such as looking at a fluorescent light bulb or exiting a dark building or tunnel into bright sunlight.

Extra Information

The rs10427255 TT genotype is associated with typical odds of having the photic sneeze reflex (PMID: 20585627). This association has not yet been replicated.For photic sneeze reflex, we find a novel association with rs10427255 (score 10.9 and an OR of 1.32). We also find a suggestive association with rs11856995 (score 7.13 and OR of 0.78). 10.1371/journal.pgen.1000993.The C allele appears to be associated with higher odds of photic sneezing.

Sweetness Detection

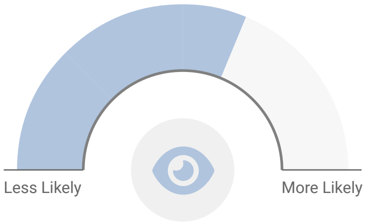

Possessing the C/C genotype for the TAS1R3 gene, you might have typical sensitivity to sucrose.

• **Gene:** TAS1R3 • **rsID:** rs35744813 • **Your Genotype:** T/T>C/C • **Zygosity:** Homozygous

Description

Sugar is a carbohydrate molecule known for its sweet taste and can exist in various forms, including fructose, sucrose, galactose, and glucose. Sucrose, commonly known as table sugar, is a compound of fructose and glucose naturally present in sources like sugar cane. Variations in individuals' ability to perceive differences in sucrose levels have been associated with specific gene variants.

Extra Information

The rs35744813 CC genotype is linked to the typical sensitivity to sucrose , TT and CT genptype strongly correlate with human taste sensitivity to sucrose (decreased Sucrose Sensitivity) and explain 16% of population variability in perception (PMID: 19559618). T alleles of rs35744813 occur at lowest frequencies in European populations.

Taste Preference and Perception: bitter taste

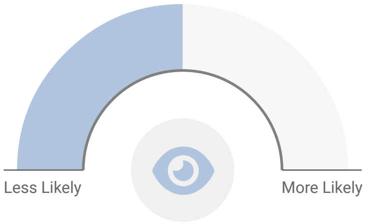

No strong evidence links the A/A genotype to bitter taste perception.

• **Gene:** TAS2R38 • **rsID:** rs1726866 • **Your Genotype:** G/G>A/A • **Zygosity:** Homozygous

Description

The perception of ""bitterness"" is inherent in our taste buds. Compounds like the phenylthiocarbamide (PTC) molecule and its counterparts are recognized for their bitter taste and can be present in various foods, including coffee, broccoli, and Brussels sprouts. The sensitivity of our taste bud receptors to these compounds is influenced by a combination of environmental and genetic factors.

Extra Information

The rs1726866 CC genotype has been linked to an elevated ability to detect bitter taste, as reported in studies (PMID(s): 12595690, 27711175)*.The TT genotype represents a possibility of unabling to taste bitter.

Taste Preference and Perception: bitter taste

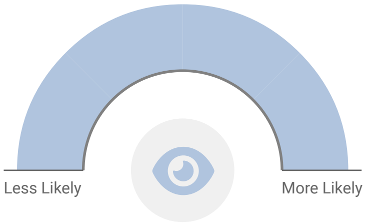

For your Taste Preference trait with the TAS2R38 gene (rs713598), having the C/C genotype makes you a bitter taster, possibly making you sensitive to foods like broccoli and coffee.

- **Gene:** TAS2R38
- **rsID:** rs713598
- **Your Genotype:** C/C
- **Zygosity:** homo_ref

Description

The perception of ""bitterness"" is inherent in our taste buds. Compounds like the phenylthiocarbamide (PTC) molecule and its counterparts are recognized for their bitter taste and can be present in various foods, including coffee, broccoli, and Brussels sprouts. The sensitivity of our taste bud receptors to these compounds is influenced by a combination of environmental and genetic factors.

Extra Information

rs713598 is one of three SNPs that form the main haplotypes behind the ability to perceive as bitter the taste of the compound phenylthiocarbamide (PTC) and similar molecules in foods (like cabbage and raw broccoli) or drinks (like coffee and dark beers).Due to it's minus orientation and nearly 50/50 split of allele frequency it is easily confused.In the orientation shown in dbSNP and used in SNPedia, rs713598(G) is the ""non-tasting"" allele,rs713598(C) is the ""tasting"" allele.In the GRCh37 orientation currently (2017) reported by 23andMe, rs713598(C) is the ""non-tasting"" allele rs713598(G) is the ""tasting"" allele. Tasting is dominant, so heterozygotes are able to taste bitterness.If you are a ""taster"", you're also likely to carry at least one rs10246939(C) and one rs1726866(C) allele since, along with rs713598(G), these three SNPs form the most common tasting haplotype. If you lack these alleles, you're quite likely (~80%) to be a non-taster of bitterness, meaning that foods that may taste bitter to others taste far less bitter to you.[PMID 12595690]

Substance Reaction

Reactions to substances like caffeine, alcohol, and various medications are influenced by genetic variants affecting metabolic pathways. Enzyme activity levels, influenced by genetic polymorphisms, can determine the speed at which substances are metabolized, influencing tolerance levels and adverse reactions. Pharmacogenomics uses this knowledge to optimize drug efficacy and minimize side effects.

Nicotine Response

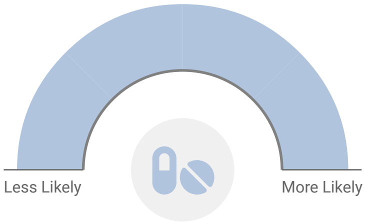

Carriers of the A/A genotype might face challenges quitting smoking and an increased lung cancer risk if they smoke.

- **Gene:** CHRNA3
- **rsID:** rs1051730
- **Your Genotype:** G/G>A/A
- **Zygosity:** Homozygous

Description

Nicotine is a naturally occurring compound found in the tobacco plant, Nicotiana tabacum, and is present in tobacco products like cigarettes, dip, and chew. Its high addictiveness is attributed to its interaction with receptors in the brain, leading to the release of neurotransmitters, including dopamine, associated with pleasurable feelings. The response to nicotine exposure is influenced by specific genetic factors, which can impact susceptibility to addiction.

Extra Information

The rs1051730 CT (which is GA in the forward strand) genotype has been associated with an increased quantity of cigarettes smoked per day among smokers, as reported in studies (PMID(s): 18385739, 19247474, 19132693, 20418890, 27127891)*.AA genotype is associated with increased risk of lung cancer; reduced response to alcohol, therefore possibly increased risk of alcohol abusers.1051730, also known as D398N, is a SNP in the nicotinic acetylcholine receptor alpha 3 subunit CHRNA3 gene.In two recent (2008) studies, together comprising over 6,000 lung cancer patients of European ancestry, the rs1051730(A) allele was very significantly associated with increased risk. Having one copy (i.e. being a rs1051730(GA) genotype) increased risk for lung cancer about 1.3x, and having two copies (rs1051730(AA) individuals) represented 1.8x increased risk. Up to 14% of lung cancer incidence may be attributable to this allele.[PMID 18385738, PMID 18385676]An independent study published at the same time concluded that (A) allele carriers for SNP rs1051730 are not at higher risk of becoming smokers compared to (G) carriers. However, if they do smoke, (A) carriers are quite likely to smoke more cigarettes than (G) carriers, and as an apparent consequence, they are at higher risk for lung cancer as reported in this and other studies. This study therefore links rs1051730 directly to nicotine dependence, and indirectly to lung cancer. [PMID 18385739] 23andMe blog (AA) makes it harder to quit smoking.

Name: | Report ID: WBWG_01_P001_262
DoB: | Patient ID: 01_P001_262
Gender: | Date: 6/7/2025

Wellness

Overall wellness is affected by genetic predispositions towards certain health conditions, stress resilience, and lifestyle adaptability. Genes influencing circadian rhythms, inflammation pathways, and detoxification processes can impact daily energy levels, mood stability, and overall health. Genetic insights provide a roadmap for lifestyle modifications that enhance well-being and longevity.

Deep Sleep

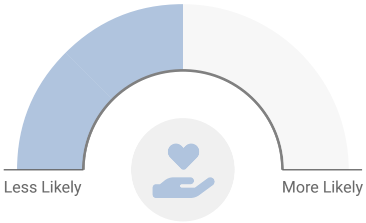

For your Deep Sleep trait with the ADA gene (rs73598374), having a C/C genotype doesn't show notable effects.

- **Gene:** ADA
- **rsID:** rs73598374
- **Your Genotype:** C/C
- **Zygosity:** homo_ref

Description

Deep sleep, also called slow-wave sleep,?ccurs in the third stage of?on-rapid eye movement?NREM) sleep. During deep sleep, electrical activity in the brain appears in long, slow waves called delta waves. These waves have a frequency of 0.5 to 2 Hertz.Research studies have identified a variation in the ADA gene, revealing its influence on deep sleep. The ADA gene, responsible for producing the adenosine deaminase enzyme, plays a crucial role in breaking down adenosine? molecule central to the regulation of various physiological processes. Alterations in the ADA gene correlate with a diminished conversion of adenosine, potentially resulting in an accumulation of adenosine and heightened fatigue. This insight underscores the significance of genetic factors in shaping the intricacies of deep sleep and its impact on overall physiological well-being.

Extra Information

The rs73598374 polymorphism with T allele leading to decreased adenosine deaminase (ADA) activity due to an amino acid substitution, has been extensively studied regarding its impact on sleep patterns and cognitive functions in humans. ADA enzyme is vital in the metabolic pathway of adenosine, a key regulator of sleep and arousal. A study (PMID: 21734253) highlights that this functional ADA polymorphism, particularly the T allele (TT genotype) of rs73598374, results in reduced ADA activity due to an amino acid substitution, and is associated with deeper sleep and decreased vigilant attention. Consequently, individuals carrying the T allele may experience more profound sleep phases and might find it challenging to sustain alertness during specific tasks.

Sleep Movement

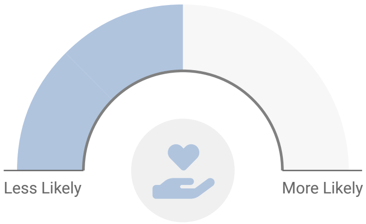

With the A/G genotype, you might have a slightly increased risk for restless legs syndrome.

- **Gene:** BTBD9
- **rsID:** rs3923809
- **Your Genotype:** A/A>A/G
- **Zygosity:** Heterozygous

Description

Abnormal movements and behaviors during sleep are part of a larger group of nocturnal events that may occur during sleep, wake, or the transitions into or out of sleep. These events are most common early in life, affecting approximately 15 to 20 percent of children and 4 percent of adults?"This SNP, located in an intron of the BTBD9 gene, has a variant that is seen somewhat more frequently in individuals with restless legs syndrome. The risk allele is rs3923809(A). Carriers of two such alleles, i.e. those with rs3923809(AA) genotypes, are estimated to be 1.9 fold more likely to have restless legs syndrome than rs3923809(G;G) individuals. The authors of this study suggest that perhaps half of the cases of restless legs syndrome may involve the rs3923809 risk genotypes. [PMID 17634447]Consistent with this finding, another report about rs3923809 links the (G) minor allele to a lower frequency of restless legs syndrome with an overall odds ratio of 0.57 (CI: 0.48-0.68). [PMID 17637780].AA genotype represents 1.9x risk for developing restless legs syndrome; AG genotype represents slightly increased risk of developing restless legs syndrome;GG genotype represents normal risk of developing restless legs syndrome."

Extra Information

This SNP, located in an intron of the BTBD9 gene, has a variant that is seen somewhat more frequently in individuals with restless legs syndrome. The risk allele is rs3923809(A). Carriers of two such alleles, i.e. those with rs3923809(AA) genotypes, are estimated to be 1.9 fold more likely to have restless legs syndrome than rs3923809(G;G) individuals. The authors of this study suggest that perhaps half of the cases of restless legs syndrome may involve the rs3923809 risk genotypes. [PMID 17634447]Consistent with this finding, another report about rs3923809 links the (G) minor allele to a lower frequency of restless legs syndrome with an overall odds ratio of 0.57 (CI: 0.48-0.68). [PMID 17637780].AA genotype represents 1.9x risk for developing restless legs syndrome; AG genotype represents slightly increased risk of developing restless legs syndrome;GG genotype represents normal risk of developing restless legs syndrome.

Hormones

Hormonal balance, crucial for physiological and psychological health, is regulated by genetic variants that influence hormone synthesis, receptor sensitivity, and feedback mechanisms. These genetic factors can determine susceptibility to conditions like thyroid disorders, metabolic syndrome, and reproductive health issues. Understanding these influences aids in personalized hormone management strategies.

Menopause Age

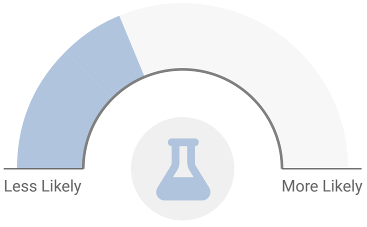

For G/G, the typical age of menopause onset is expected, possibly slightly sooner than average.

• **Gene:** MCM8 • **rsID:** rs16991615 • **Your Genotype:** G/G • **Zygosity:** homo_ref

Description

Menopause refers to the cessation of a woman's menstrual cycle, indicating the end of her reproductive capability. This natural biological process also signifies the conclusion of her ability to conceive children. Menopause involves significant hormonal changes, leading to symptoms like hot flashes and mood alterations. The onset of menopause is typically recognized when there has been a 12-month span without menstruation. In the United States, the average age of menopause is around 51 years, though individual variations exist. Factors influencing the timing of menopause include natural hormonal variations, medical conditions or treatments affecting hormone production, and surgical interventions like the removal of the ovaries, a key source of female sex hormones. Additionally, genetic variations play a role in determining the age at which menopause occurs.

Extra Information

The rs16991615 GG genotype has been associated with the typical age of menopause onset, according to studies (PMID(s): 20952801, 23508249, 23592221, 19448621).The AA genotype is associated with menopause increased by avg of 22 months and AG genotype is slight increase (11 months) in avg age at menopause.rs16991615 is a nonsynonymous SNP on chromosome 20.A case control study of ~2,000 women found that each rs16991615(A) allele was associated with an average increase in age at menopause of 11 months. This also meant that each rs16991615(G) allele yielded an odds ratio of 1.85 (CI: 1.51?.2, p = 1.45 x 10e-9) of early menopause, defined as occurring before the age of 46.[PMID 20952801].Overall, women homozygous for the ""early"" alleles at all 4 SNPs found in this study were ~4 times more likely to undergo menopause early compared to women who had a total of 3 or less risk alleles.

Testosterone Levels

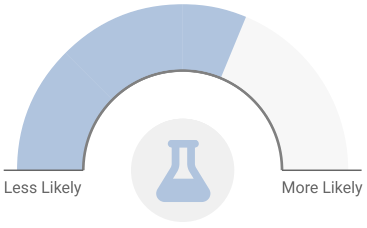

Your Testosterone Levels with the SHBG gene (rs1799941) might be on the higher side with the A/A genotype, supporting increased SHBG and testosterone.

• **Gene:** SHBG • **rsID:** rs1799941 • **Your Genotype:** G/G>A/A • **Zygosity:** Homozygous

Description

Testosterone, an androgenic hormone associated with male development, is primarily produced in the testicles and ovaries. Additionally, it can be converted from other androgens produced by the adrenal glands. In men, testosterone levels are correlated with aspects such as sexual interest (libido), fertility, mood, and erectile function. These levels naturally decline with age, and genetic factors can contribute to individual variations in testosterone levels.

Extra Information

The rs1799941 is significantly associated with sex-hormone binding globulin (SHBG) levels as well as sex hormone regulation. AG genotype is linked to elevated levels of serum testosterone and Sex Hormone-Binding Globulin (PMID(s): 24327369, 29264510). "A" allele was associated with increasing testosterone levels in men (P= 8.11 _ 10_6)(PMID: 19574343).Subjects with the "A" allele (GA+AA) had a trend for lower free estradiol index (FEI) compared to the GG genotype (p=0.04)(PMID: 19679209). Each copy of the "A" allele is associated with a 0.20 SDs increase in sex hormone binding globulin (SHBG) levels(PMID: 19933169). Carriers of the minor allele "A" had higher circulating SHBG, independently of sex, age, and total body fat (P = 0.025)(PMID: 20841609).

Longevity

Longevity is shaped by genetic variants that regulate cellular repair mechanisms, oxidative stress response, and metabolic efficiency. Telomere length, mitochondrial function, and DNA repair capacity are key factors influenced by these genes, contributing to the aging process and lifespan. Genetic profiling for longevity markers provides insights into personalized anti-aging interventions.

Longevity

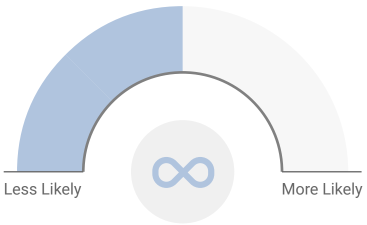

For longevity in FOXO3, the T/T genotype lacks strong evidence indicating notable effects.

• **Gene:** FOXO3 • **rsID:** rs2764264 • **Your Genotype:** C/C>T/T • **Zygosity:** Homozygous

Description

Longevity is a complex trait influenced by numerous factors, exhibiting significant variability between individuals. The aging process can manifest differently, with some individuals showing signs of aging, such as extensive skin wrinkles, completely gray hair, loss of muscle tone, and reduced mobility, in their 60s. In contrast, others may maintain a more youthful appearance well into their 90s or even close to 100 years of age. This diversity in aging patterns is attributed to the interplay of genetic and environmental factors, both of which contribute significantly to the observed variability in the aging process.

Extra Information

The rs2764264 CT genotype has been associated with a longer lifespan in men, as indicated by studies (PMID(s): 18765803, 19196970, 24350933). Also, CC genotype is associated with greater odds of longevity [PMID 20849522].Replication of an association of variation in the FOXO3A gene with human longevity using both case-control and longitudinal data.[PMID 19415983] Association of the FOXO3A locus with extreme longevity in a southern Italian centenarian study.[PMID 18765803] FOXO3A genotype is strongly associated with human longevity.[PMID 19196970] Association of FOXO3A variation with human longevity confirmed in German centenarians.[PMID 24350933] Association Between Genetic Variations In The Insulin/Insulin-Like Growth Factor (Igf-1) Signaling Pathway And Longevity: A Systematic Review And Meta-Analysis[PMID 31009445] Longevity-Associated Forkhead Box O3 (FOXO3) Single Nucleotide Polymorphisms are Associated with Type 2 Diabetes Mellitus in Chinese Elderly Women.

Name: | Report ID: WBWG_01_P001_262
DoB: | Patient ID: 01_P001_262
Gender: | Date: 6/7/2025

Physical Appearance

Genetic factors significantly determine physical characteristics such as skin pigmentation, hair texture, and facial features. Variants in genes affecting melanin production, hair follicle shape, and facial bone structure contribute to the diversity in human appearance. Understanding these genetic influences can also provide insights into conditions like alopecia and skin disorders.

Earwax Type

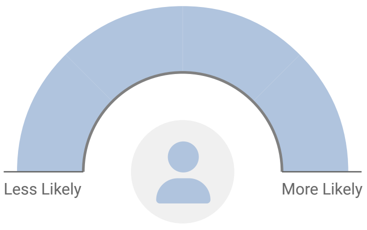

For your Earwax Type trait with the ABCC11 gene (rs17822931), having the C/C genotype likely means you have wet earwax, which is common in European and African populations.

- **Gene:** ABCC11
- **rsID:** rs17822931
- **Your Genotype:** C/C
- **Zygosity:** homo_ref

Description

Earwax, scientifically termed cerumen, is a universal waxy substance produced by glands in the ear canals, serving as a protective barrier for the ear's sensitive skin and defense against germs. Determined by the ABCC11 gene variant, individuals of European and African descent typically have wet earwax, while those of East Asian descent often exhibit dry, crumbly earwax. This genetic diversity highlights the subtle yet fascinating ways in which our genes shape seemingly ordinary aspects of our physiology, emphasizing the essential role earwax plays in maintaining ear health across different populations.

Extra Information

rs17822931, also known as c.538G>A (C>T) or G180R, is a SNP in the ATP-binding cassette, sub-family C (CFTR/MRP), member 11 ABCC11 gene. The ABCC11 protein helps transport small molecules across apical membranes such as those in apocrine secretory cells.[PMID 16444273] This SNP determines wet vs dry earwax as well as sweat production, and it is also associated with lipid secretion. The C allele is associated increased wetness of earwax. The T allele is associated with increased dryness of earwax. It is commonly presenting TT genotype for East Asians and CC for Europeans and Africans.

Eye Color

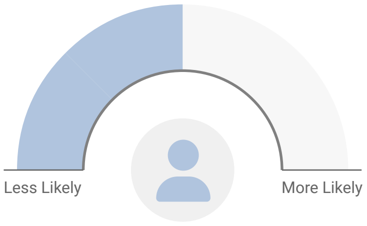

Your G/G genotype hasn't shown significant influence on eye color in studies.

- **Gene:** TYR
- **rsID:** rs1393350
- **Your Genotype:** G/G
- **Zygosity:** homo_ref

Description

Eye colors are determined by the variable pigmentation of the iris and the scattering of light by the iris. Individuals with a higher concentration of the dark pigment melanin tend to have darker eye colors, while those with a lower concentration generally have lighter-colored eyes. Light eye colors, such as blue, green, and hazel, are not produced by pigments but are mainly the result of the variable scattering of light by the iris.

Extra Information

Research conducted on individuals of European ancestry indicates that the rs1393350 AA genotype is linked to an increased likelihood of having blue eyes (PMID(s): 17952075, 23100201, 23986280, 20585627) while other genotypes are less likely to have blue eyes.

Eye Color

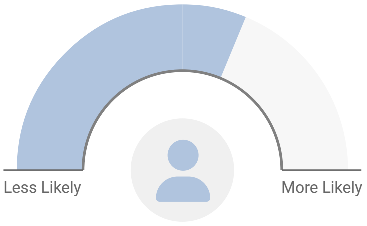

For your Eye Color trait with the HERC2 gene (rs12913832), the A/A genotype is often linked with brown eyes.

- **Gene:** HERC2
- **rsID:** rs12913832
- **Your Genotype:** A/A
- **Zygosity:** homo_ref

Description

Eye colors are determined by the variable pigmentation of the iris and the scattering of light by the iris. Individuals with a higher concentration of the dark pigment melanin tend to have darker eye colors, while those with a lower concentration generally have lighter-colored eyes. Light eye colors, such as blue, green, and hazel, are not produced by pigments but are mainly the result of the variable scattering of light by the iris.

Extra Information

Studies conducted on individuals of European ancestry indicate that the rs12913832 CT genotype is associated with an increased likelihood of having brown eye color (PMID(s): 18252222, 18172690)*.rs12913832?s a SNP near the?CA2/HERC2?ene that may be functionally linked to blue or brown?ye color, due to a lowering of promoter activity of the?CA2?ene. The genotypes rs12913832:AA and rs12913832:GA are most often observed in individuals with brown eye colours, whereas rs12913832:GG is most often observed in individuals with blue eye colours.

Finger Length Ratio

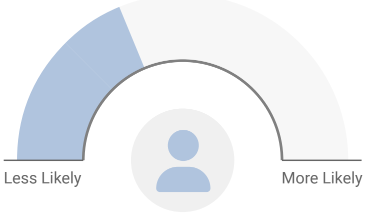

The A/G genotype could mean a slightly more masculine 2D:4D ratio.

• **Gene:** SMOC1 • **rsID:** rs2332175 • **Your Genotype:** G/G>G/A • **Zygosity:** Heterozygous

Description

Finger length is commonly measured from the point where the finger joins the hand to the tip of the finger. The finger length ratio, often denoted as 2D:4D, represents the ratio of the lengths of the index and ring fingers. This ratio is calculated by dividing the length of the index finger on one hand by the length of the ring finger on the same hand. Evidence suggests that the 2D:4D ratio may correlate with certain human characteristics, such as increased reproductive success or a reduced risk of video game addiction. These correlations vary between males and females, with the differences thought to result from in utero exposure to hormones.In general, 2D:4D is higher in females (M = 0.979 SD = 0.030) than in males (M = 0.964, SD = 0.031); this difference was highly significant [t(977) = _7.3, P = 6.48 _ 10_13].More men than women have shorter index fingers than ring fingers, resulting in a lower 2D:4D ratio. Therefore, a lower 2D:4D ratio is indicative of a more masculine hormonal profile or environment during fetal development. These differences are influenced by a combination of genetic and environmental factors.

Extra Information

The rs2332175 AA genotype is reported to be associated with the typical difference in length between the 2nd and 4th fingers, as indicated by a study (PMID: 23263445). Each additional copy of the G allele was associated with a 0.0074 decrease in 2D:4D(P value 3.44 _ 10_8).

Freckling

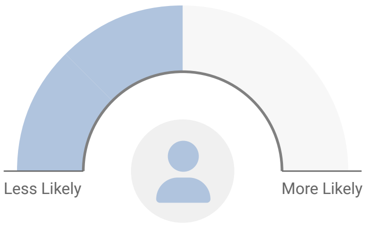

With the C/C genotype, you have an average likelihood of freckling.

• **Gene:** MC1R • **rsID:** rs1805007 • **Your Genotype:** C/C • **Zygosity:** homo_ref

Description

Freckles are clusters of concentrated melaninized cells that are particularly noticeable on fair skin. The primary environmental trigger for the formation of freckles is exposure to sunlight. When exposed to UV-B radiation, melanocytes, the cells responsible for producing melanin, increase their melanin production. This heightened melanin production causes freckles to darken, making them more visible. In cases where there are densely distributed concentrations of melanin, freckles may multiply and extend over a broader area of the skin.

Extra Information

Research conducted on individuals of European ancestry suggests that the rs1805007 CC genotype is associated with an average likelihood of freckling (PMID(s): 20876667, 17952075). CT genotype represents carrier of a red hair associated variant; higher risk of melanoma.TT genotype represents increased response to anesthetics, 13-20x higher likelihood of red hair, higher likelihood of freckling presense in childhood and increased risk of melanoma.

Hair Color

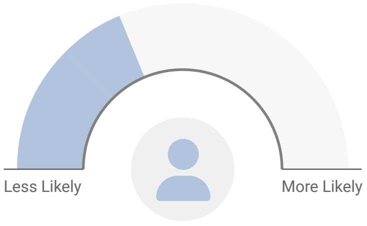

A T/T genotype might mean an average chance of having blonde hair.

• **Gene:** Intergenic • **rsID:** rs12821256 • **Your Genotype:** T/T • **Zygosity:** homo_ref

Description

Hair color is primarily determined by the relative levels of two pigments in the hair: eumelanin, comprising brown and black variants, and pheomelanin, contributing a red hue. Higher eumelanin levels result in dark hair, while lower levels of both pigments lead to nearly white to yellowish hair. With varying eumelanin proportions, hair color spans from very pale blond to brown shades. Hair low in eumelanin can exhibit colors from light blond to reddish ""strawberry"" blond or red, depending on varying pheomelanin amounts.

Extra Information

Research on individuals of European ancestry indicates that the rs12821256 TT genotype is associated with an average likelihood of having blond hair (PMID(s): 24880339, 17952075, 26184321),CC genotype is 4x more likely to have blond hair and CT genptype is 2x more likely to have blond hair.

Hair curl

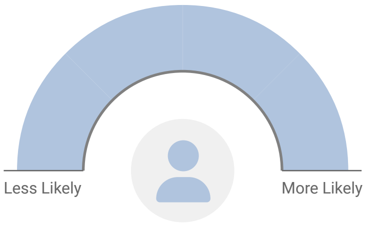

For your Hair curl trait with the WNT10A gene (rs7349332), having the C/C genotype might mean you're more likely to enjoy straighter hair.

• **Gene:** WNT10A • **rsID:** rs7349332 • **Your Genotype:** C/C • **Zygosity:** homo_ref

Description

The curliness of an individual's hair is determined by the shape of the hair shaft. A hair shaft that is very round allows for fewer bonds between molecules, resulting in straight hair. Conversely, a flatter hair shaft promotes more bonds between adjacent molecules, leading to a bent shape and expressing as hair curl.

Extra Information

The rs7349332 CT and TT genotype has been associated with an increased likelihood of having curly hair, according to studies (PMID(s): 26414620, 28370528, 20585627)*.CC genotype is associated with straighter hair.

Name:
DoB:
Gender:
Report ID: WBWG_01_P001_262
Patient ID: 01_P001_262
Date: 6/7/2025

Hair curl

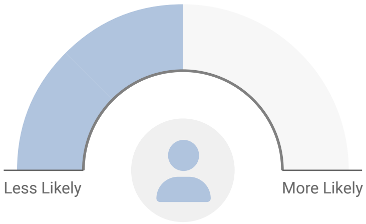

For the Hair curl trait with the TCHHL1 gene (rs17646946), we don't have strong data linking the G/G genotype to specific hair textures.

• **Gene:** TCHHL1 • **rsID:** rs17646946 • **Your Genotype:** G/G • **Zygosity:** homo_ref

Description

The curliness of an individual's hair is determined by the shape of the hair shaft. A hair shaft that is very round allows for fewer bonds between molecules, resulting in straight hair. Conversely, a flatter hair shaft promotes more bonds between adjacent molecules, leading to a bent shape and expressing as hair curl.

Extra Information

Research conducted on individuals of European ancestry suggests that the rs17646946 CC genotype is associated with an average likelihood of having straight hair (PMID(s): 29220522, 20585627, 19896111, 26414620) .The minor allele is associated with straighter hair, with each A conferring a reduction in curliness of about 0.29 points on a scale from 0 to 5(PMID: 20585627).

Hair thickness

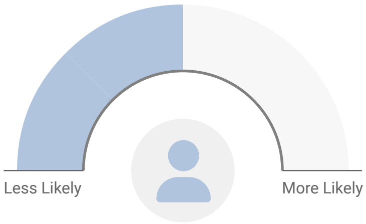

For Hair thickness with your EDAR gene (rs3827760), we don't have strong data correlating the A/A genotype with hair thickness.

• **Gene:** EDAR • **rsID:** rs3827760 • **Your Genotype:** A/A • **Zygosity:** homo_ref

Description

Hair thickness, or volume, is contingent on the size of the hair follicle. A larger circumference of the hair follicle typically results in thicker hair.

Extra Information

In Asian individuals, the rs3827760 TT genotype has been associated with typical hair thickness, as suggested by studies (PMID(s): 18065779, 18704500, 18561327, 23793515, 27487801).CC genotype: Associated with straighter, thicker hair and an increased likelihood of having shovel-shaped incisors.CT genotype: Linked to an increased chance of having straighter, thicker hair, as well as shovel-shaped incisors.The rs3827760(C) allele is far more prevalent in East Asians and Native Americans than in any other population, and it is used as such in ancestry tests [PMID 22749789]. There are at least four physical characteristics that have been linked to this allele, and pretty much all in a copy-dependent manner as well (inheriting both alleles leads to a stronger expression than inheriting one):Hair follicle thickness [PMID 18065779]Hair straightness (i.e. lack of curliness).

Height

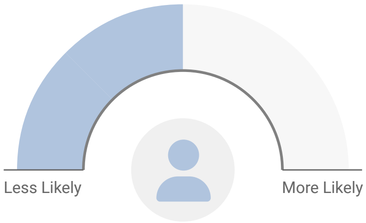

With the C/T genotype in the HMGA2 gene, you might stand a little taller, about 0.4 cm more than average.

• **Gene:** HMGA2 • **rsID:** rs1042725 • **Your Genotype:** C/C>C/T • **Zygosity:** Heterozygous

Description

Height is the measurement of the length from the floor to the top of an individual's head when standing upright. This measurement is expressed in inches, feet, centimeters, or meters and tends to remain relatively stable throughout adulthood until later stages of life. Both genetic and environmental factors, including nutrition and exercise, play roles in influencing an individual's height. Specific genetic variations have been identified in association with the final height achieved by an individual.

Extra Information

The rs1042725 TT genotype has been associated with average height, based on studies (PMID(s): 19139030, 17767157, 19930247, 21921580, 26536448) while CC genotype indicates- ~0.8cm taller height; CT genotype indicates ~0.4cm taller height. SNP rs1042725 is associated with height (P = 4E-8) in a study involving over 20,000 individuals. The gene harboring this SNP, HMGA2, is a strong biological candidate for having an influence on height, since rare, severe mutations in this gene are known to alter body size in mice and humans.rs1042725 is estimated to explain approx 0.3% of population variation in height in both adults and children (approx 0.4 cm increased adult height per C allele).[PMID 19139030] rs1042725 is associated with increased height (0.36 cm 95% IC[0.12-0.61] per C allele, P=0.004). in men rs1042725 may explain 3% of height variability.

Iris Patterns

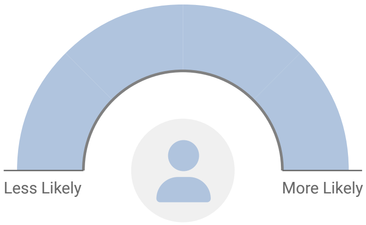

For your Iris Patterns trait with the TRAF3IP1 gene, having the A/A genotype might give you extended contraction furrows in your iris.

• **Gene:** TRAF3IP1 • **rsID:** rs3739070 • **Your Genotype:** A/A • **Zygosity:** homo_ref

Description

The human iris, aside from its color, exhibits distinctive features such as Fuchs?crypts, nevi, Wolfflin nodules, and contraction furrows (circular grooves running in a circular pattern around the iris). These unique characteristics are currently employed as biomarkers for various purposes. The tissue patterns present in the human iris are utilized for automatic personal identification, providing a reliable basis for distinguishing individuals. Additionally, these tissue markers in the iris can be associated with various eye diseases, offering insights and diagnostic potential in the field of ophthalmology.

Extra Information

Research involving individuals of European ancestry suggests that the rs3739070 AA genotype is associated with the presence of extended contraction furrows, as indicated in studies (PMID(s): 21835309, 26909168)*compared to other genotypes.

Male Pattern Baldness

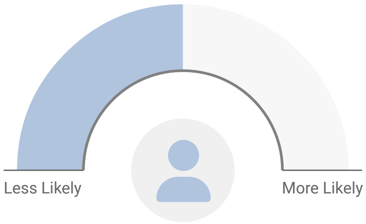

For Male Pattern Baldness with the SLC14A2 gene, having the A/C genotype doesn't show notable effects.

• **Gene:** SLC14A2 • **rsID:** rs8085664 • **Your Genotype:** C/C>C/A • **Zygosity:** Heterozygous

Description

Male Pattern Baldness is characterized by hair loss that occurs when the hair follicle starts to shrink and continues to do so over time. This loss is attributed to an interaction between specific genetic factors and the male hormone dihydrotestosterone (DHT). The hair loss follows a well-defined pattern, typically starting above both temples. As the hair follicle continues to shrink, the affected hair becomes shorter and finer, ultimately leading to a lack of new hair growth in the affected areas.

Extra Information

Research on individuals of European ancestry suggests that the rs8085664 CC genotype is associated with higher risk of baldness, while other genotypes may be associated with an average likelihood of male baldness, as reported in studies (PMID(s): 22693459, 28196072)*.

Male Pattern Baldness

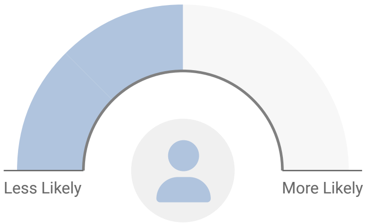

Currently, C/C doesn't show a strong effect on your likelihood of male pattern baldness.

• **Gene:** C1orf127 • **rsID:** rs2003046 • **Your Genotype:** C/C • **Zygosity:** homo_ref

Description

Male Pattern Baldness is characterized by hair loss that occurs when the hair follicle starts to shrink and continues to do so over time. This loss is attributed to an interaction between specific genetic factors and the male hormone dihydrotestosterone (DHT). The hair loss follows a well-defined pattern, typically starting above both temples. As the hair follicle continues to shrink, the affected hair becomes shorter and finer, ultimately leading to a lack of new hair growth in the affected areas.

Extra Information

Research involving individuals of European ancestry indicates that the rs2003046 GG genotype (homozygous mutation) is associated with a higher likelihood of male baldness, as reported in studies (PMID(s): 22693459, 28196072) while other genotypes are associated with lower risk of baldness.

Pigmentation

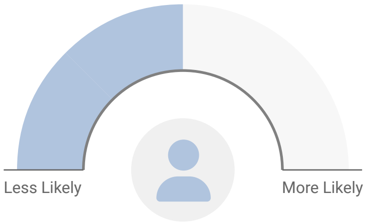

The G/G genotype for Pigmentation doesn't indicate noticeable changes under current research.

• **Gene:** OCA2 • **rsID:** rs4778138 • **Your Genotype:** A/A>G/G • **Zygosity:** Homozygous

Description

Pigmentation refers to the coloring of skin, eyes, hair, and nails, with melanocytes responsible for producing melanin?he primary pigment influencing coloration. Higher melanin levels are linked to darker hues, while lower levels are associated with lighter tones. Melanin also contributes to the formation of freckles, characterized by brown spots resulting from sun exposure. The synthesis of melanin involves a complex pathway influenced by various environmental factors, including UV rays, alongside genetic factors.

Extra Information

The rs4778138 TT genotype is linked to blue eye color, heightened freckling, and a greater number of moles in comparison to individuals with brown eyes (PMID: 17236130). Other genptypes are assoicated with brwon eyes and darker skin.

Pigmentation

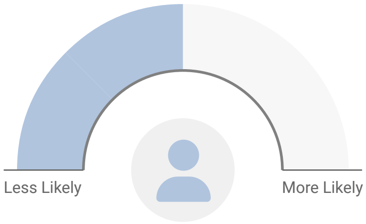

C/C does not exhibit a significant correlation with changes in pigmentation.

• **Gene:** HERC2 • **rsID:** rs1667394 • **Your Genotype:** C/C • **Zygosity:** homo_ref

Description

Pigmentation refers to the coloring of skin, eyes, hair, and nails, with melanocytes responsible for producing melanin?he primary pigment influencing coloration. Higher melanin levels are linked to darker hues, while lower levels are associated with lighter tones. Melanin also contributes to the formation of freckles, characterized by brown spots resulting from sun exposure. The synthesis of melanin involves a complex pathway influenced by various environmental factors, including UV rays, alongside genetic factors.

Extra Information

The rs1667394 A allele increases susceptibility to Blue rather than brown eyes 29.43 times [PMID 17952075] and increases susceptibility to Blue rather than green eyes 6.74 times [PMID 17952075]. GG genotype is associated with darker skin, eye and hair color. AA genotype is associated with blond hair and blue eyes.

Name:
DoB:
Gender:

Report ID: WBWG_01_P001_262
Patient ID: 01_P001_262
Date: 6/7/2025

Pigmentation

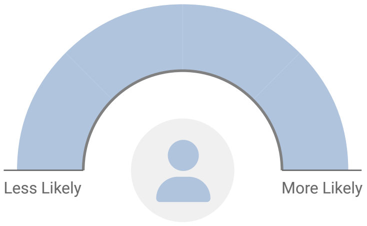

For your pigmentation with the LOC105370627 gene (rs12896399), having the G/G genotype often means a likelihood of blue eyes and blond hair.

• **Gene:** LOC105370627 • **rsID:** rs12896399 • **Your Genotype:** G/G • **Zygosity:** homo_ref

Description

Pigmentation refers to the coloring of skin, eyes, hair, and nails, with melanocytes responsible for producing melanin?he primary pigment influencing coloration. Higher melanin levels are linked to darker hues, while lower levels are associated with lighter tones. Melanin also contributes to the formation of freckles, characterized by brown spots resulting from sun exposure. The synthesis of melanin involves a complex pathway influenced by various environmental factors, including UV rays, alongside genetic factors.

Extra Information

Studies in Europeans show that the rs12896399 GG genotype is associated with a typical likelihood of having blue eyes and blond hair and TT genotype is associated with lighter hair color and more likelyhood of blue eyes (PMID: 17952075).

Red hair color

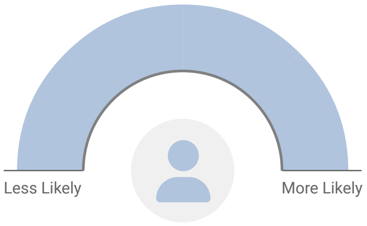

For your Red hair color trait with the MC1R gene (rs1805007), having the C/C genotype significantly increases your chance of having red hair and potentially higher melanoma risk.

• **Gene:** MC1R • **rsID:** rs1805007 • **Your Genotype:** C/C • **Zygosity:** homo_ref

Description

Red hair, often referred to as orange or ginger hair, is a distinctive human hair color present in 1?% of the global population, with a higher prevalence (2?%) among individuals of Northern or Northwestern European descent and less common occurrence in other populations. The unique hue is primarily attributed to a recessive allele on chromosome 16, leading to a modified version of the MC1R protein. This genetic trait is most prevalent in individuals homozygous for this recessive allele, highlighting the fascinating interplay between genetics and hair color expression in human populations.

Extra Information

rs1805007 CC genotype, known as Arg151Cys or R151C, one of several SNPs in the MC1R gene associated with red hair color (redheads), and in redheaded females.CT genotype represents carrier of a red hair associated variant; higher risk of melanoma.TT genotype represents increased response to anesthetics, 13-20x higher likelihood of red hair, higher likelihood of freckling presense in childhood and increased risk of melanoma.

Red hair color

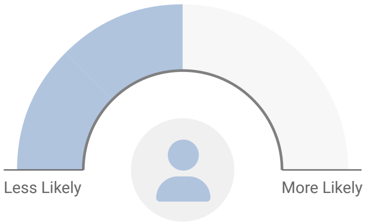

This genotype is not typically associated with red hair.

• **Gene:** MC1R • **rsID:** rs1805008 • **Your Genotype:** C/C • **Zygosity:** homo_ref

Description

Red hair, often referred to as orange or ginger hair, is a distinctive human hair color present in 1?% of the global population, with a higher prevalence (2?%) among individuals of Northern or Northwestern European descent and less common occurrence in other populations. The unique hue is primarily attributed to a recessive allele on chromosome 16, leading to a modified version of the MC1R protein. This genetic trait is most prevalent in individuals homozygous for this recessive allele, highlighting the fascinating interplay between genetics and hair color expression in human populations.

Extra Information

rs1805008, known as Arg160Trp or R160W, is one of several SNPs in the MC1R gene associated with red hair color (redheads), in this case in an Irish population [PMID 9665397] although this has also been reported in Icelandic and Dutch populations [PMID 18488028].The TT genotype is associated with ~7-10x higher likelihood of red hair and higher risk of melanoma, compared to CC genotype. CT genotype represents carrier for red hair gene.

Red hair color

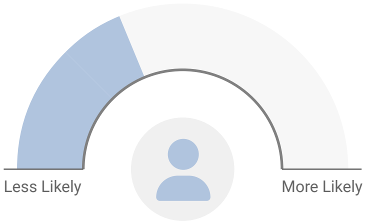

The G/G genotype is common and may not increase your chances of having red hair.

• **Gene:** MC1R • **rsID:** rs1805009 • **Your Genotype:** G/G • **Zygosity:** homo_ref

Description

Red hair, often referred to as orange or ginger hair, is a distinctive human hair color present in 1?% of the global population, with a higher prevalence (2?%) among individuals of Northern or Northwestern European descent and less common occurrence in other populations. The unique hue is primarily attributed to a recessive allele on chromosome 16, leading to a modified version of the MC1R protein. This genetic trait is most prevalent in individuals homozygous for this recessive allele, highlighting the fascinating interplay between genetics and hair color expression in human populations.

Extra Information

rs1805009, known as Asp294His or D294H and located in the MC1R gene, is a variant associated with red hair (redheads) and low tanning in one study. [PMID 30531825O A-icon.png],[PMID 7581459] Reported as i3002507 by 23andMe in January 2015.CC-red hair possible, higher risk of melanoma. CG genotype represents red hair carrier, higher risk of melanoma and GG represents common risk.

Skin pigmentation

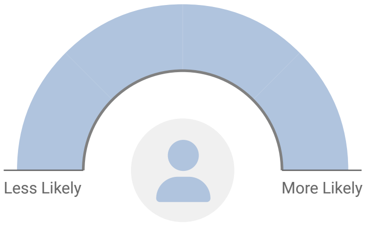

For your skin pigmentation with the SLC45A2 gene (rs16891982), the G/G genotype suggests lighter skin common in European ancestries, but take care for melanoma risks.

• **Gene:** SLC45A2 • **rsID:** rs16891982 • **Your Genotype:** C/C>G/G • **Zygosity:** Homozygous

Description

Skin pigmentation, which refers to how much melanin the body generates, determines the color of the skin. The two main types of melanin, eumelanin, and pheomelanin, are produced by melanocytes in the epidermal layer of the skin.Constitutive skin pigmentation is a polygenic trait and, in recent years, the number of genes and the allelic variants that affect human skin pigmentation that have been identified has significantly increased.

Extra Information

This SNP is associated with skin, hair, and eye colour, and risk of melanoma. Individuals with the G;G genotype are generally of European descent and have fair skin.CC:generally non-European, but if European, 7x more likely to have black hair, average skin pigmentation.CG:if European, 7x more likely to have black hair;lower risk of melanoma. GG:Generally European; Lighter skin pigmentation; Possibly an increased risk of melanoma. Brown eye color is predicted for the C/C gneotype plus A/A or G/A at rs12913832, green eye color is predcited by C/Cc genotype plus G/G at rs12913832 [R].The "C" allele was strongly associated with melanoma and pigmentation traits (OR 0.51; P_=_0.001) [R].

Skin pigmentation

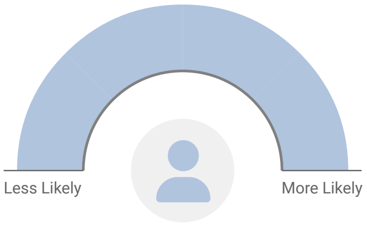

Having the A/A genotype for skin pigmentation in the SLC24A5 gene (rs1426654) suggests a likelihood of light skin, typical of West Eurasian ancestry.

• **Gene:** SLC24A5 • **rsID:** rs1426654 • **Your Genotype:** A/A • **Zygosity:** homo_ref

Description

Skin pigmentation, which refers to how much melanin the body generates, determines the color of the skin. The two main types of melanin, eumelanin, and pheomelanin, are produced by melanocytes in the epidermal layer of the skin.Constitutive skin pigmentation is a polygenic trait and, in recent years, the number of genes and the allelic variants that affect human skin pigmentation that have been identified has significantly increased.

Extra Information

This SNP influences skin pigmentation. The rs1426654 A allele, indicates light-skinned West Eurasian ancestry. [PMID 16847698, PMID 16357253]It appears as if this SNP is a relatively new one in human evolution; one estimate [PMID 17182896] is that the rs1426654(A) allele, in other words, light skin pigmentation, spread through the European population around 6,000 - 12,000 years ago. Prior to that, ""European ancestors"" were most likely relatively brown-skinned. Another study ([PMID 24048645]) has concluded that almost individuals carrying the A111T variant can trace ancestry back to a single person who most likely lived at least 10,000 years ago.This SNP is one of three from the SLC24A5 gene that can be analyzed to categorize the ancestry of a person as either West Eurasian (Middle Eastern, Caucasian, European, etc.), African, or East Eurasian, based on a 2009 study.[PMID 19440451]

Personality

Personality traits, such as extroversion, conscientiousness, and emotional stability, have genetic underpinnings linked to neurotransmitter activity and brain structure. Genetic variations in serotonin and dopamine pathways can influence mood regulation, stress resilience, and social behavior. These insights help in understanding individual differences in personality and psychological well-being.

Agreeableness

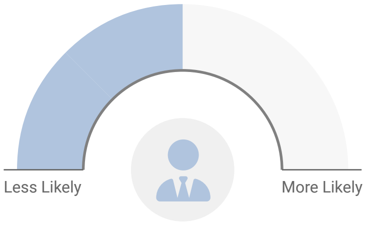

Carrying the A/G genotype doesn't have a noticeable effect on your level of agreeableness, so just be yourself.

• **Gene:** TCF4 • **rsID:** rs7240986 • **Your Genotype:** G/G>G/A • **Zygosity:** Heterozygous

Description

Agreeableness is personality trait that describes a person's ability to put others needs before their own. Those who are more agreeable are more likely to be empathetic and find pleasure in helping others and working with people who need more help

Extra Information

This variant with allele G is identified from the paper "A genome-wide investigation into the underlying genetic architecture of personality traits and overlap with psychopathology"(PMID: 39134740).This variant is associated with a small decrease in agreeableness with an effect size of -0.0307.

Agreeableness

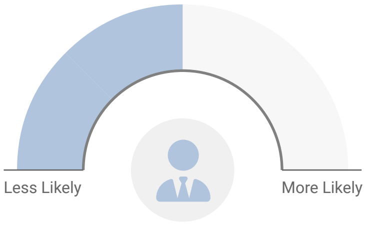

The C/C genotype at this location doesn't seem to significantly impact your agreeableness levels.

• **Gene:** FOXP2 • **rsID:** rs17137124 • **Your Genotype:** T/T>C/C • **Zygosity:** Homozygous

Description

Agreeableness is personality trait that describes a person's ability to put others needs before their own. Those who are more agreeable are more likely to be empathetic and find pleasure in helping others and working with people who need more help

Extra Information

This variant with allele T is identified from the paper "A genome-wide investigation into the underlying genetic architecture of personality traits and overlap with psychopathology"(PMID: 39134740).This variant slightly decreases agreeableness with an effect size of -0.03273.

Agreeableness

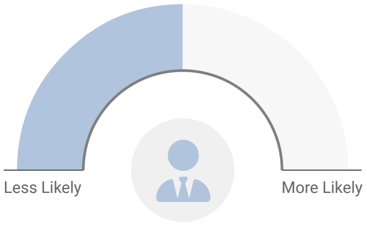

The G/G genotype doesn't appear to have a measurable effect on agreeableness.

• **Gene:** Intergenic • **rsID:** rs7833945 • **Your Genotype:** G/G • **Zygosity:** homo_ref

Description

Agreeableness is personality trait that describes a person's ability to put others needs before their own. Those who are more agreeable are more likely to be empathetic and find pleasure in helping others and working with people who need more help

Extra Information

This variant with allele T is identified from the paper "A genome-wide investigation into the underlying genetic architecture of personality traits and overlap with psychopathology"(PMID: 39134740).Associated with a small decrease in agreeableness with an effect size of -0.03161.

SZA Longevity
A healthy future awaits you

Name: | Report ID: WBWG_01_P001_262
DoB: | Patient ID: 01_P001_262
Gender: | Date: 6/7/2025

Conscientiousness

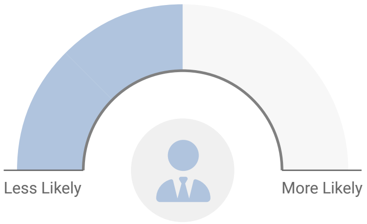

Possessing the A/A genotype does not show notable influences on conscientiousness, leaving your discipline up to you.

• **Gene:** FOXP2 • **rsID:** rs936145 • **Your Genotype:** G/G>A/A • **Zygosity:** Homozygous

Description

Conscientiousness is a core personality trait that involves being diligent, responsible, and careful. When someone is conscientious, they are able to exercise self-discipline and self-control in order to pursue and ultimately achieve their goals.People with high conscientiousness are also organized, determined, and able to postpone immediate gratification?ll of which contribute to a more successful life.

Extra Information

This variant with allele G is identified from the paper "A genome-wide investigation into the underlying genetic architecture of personality traits and overlap with psychopathology"(PMID: 39134740).Shows a small decrease in conscientiousness with an effect size of -0.0331.

Conscientiousness

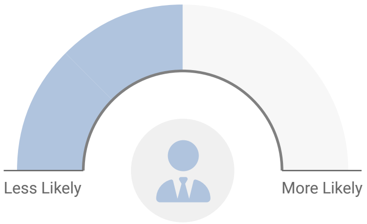

Your A/A genotype doesn't indicate any specific effect on conscientiousness, leaving room for your unique character to thrive.

• **Gene:** Intergenic • **rsID:** rs78446248 • **Your Genotype:** A/A • **Zygosity:** homo_ref

Description

Conscientiousness is a core personality trait that involves being diligent, responsible, and careful. When someone is conscientious, they are able to exercise self-discipline and self-control in order to pursue and ultimately achieve their goals.People with high conscientiousness are also organized, determined, and able to postpone immediate gratification?ll of which contribute to a more successful life.

Extra Information

This variant with allele G is identified from the paper "A genome-wide investigation into the underlying genetic architecture of personality traits and overlap with psychopathology"(PMID: 39134740).This variant is associated with a moderate decrease in conscientiousness with an effect size of -0.08581.

Extraversion

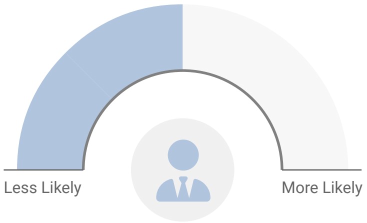

The T/T genotype shows no evident impact on your extraversion.

• **Gene:** Intergenic • **rsID:** rs35424804 • **Your Genotype:** C/C>T/T • **Zygosity:** Homozygous

Description

Extroversion is personality trait typically characterized by outgoingness, high energy, and/or talkativeness. In general, the term refers to a state of being where someone ?echarges,?or draws energy, from being with other people; the opposite?rawing energy from being alone?s known as introversion

Extra Information

This variant with allele C is identified from the paper "A genome-wide investigation into the underlying genetic architecture of personality traits and overlap with psychopathology"(PMID: 39134740).This variant is associated with a slight increase in extraversion with an effect size of 0.037166

Extraversion

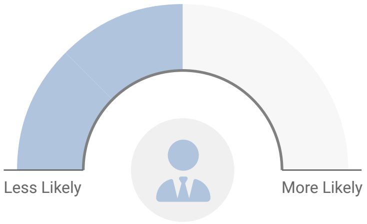

For your Extraversion trait with this genetic variant, the A/C genotype does not exhibit a notable impact.

• **Gene:** Intergenic • **rsID:** rs12971383 • **Your Genotype:** A/A>A/C • **Zygosity:** Heterozygous

Description

Extroversion is personality trait typically characterized by outgoingness, high energy, and/or talkativeness. In general, the term refers to a state of being where someone ?echarges,?or draws energy, from being with other people; the opposite?rawing energy from being alone?s known as introversion

Extra Information

This variant with allele A is identified from the paper "A genome-wide investigation into the underlying genetic architecture of personality traits and overlap with psychopathology"(PMID: 39134740).Shows a slight increase in extraversion with an effect size of 0.054198.

Name:
DoB:
Gender:
Report ID: WBWG_01_P001_262
Patient ID: 01_P001_262
Date: 6/7/2025

Extraversion

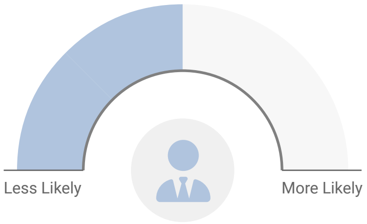

• **Gene:** Intergenic • **rsID:** rs5831479 • **Your Genotype:** GA/GA>GA/G • **Zygosity:** Heterozygous

Description
Extroversion is personality trait typically characterized by outgoingness, high energy, and/or talkativeness. In general, the term refers to a state of being where someone ?echarges,?or draws energy, from being with other people; the opposite?rawing energy from being alone?s known as introversion

Extra Information
This variant with allele GAis identified from the paper "A genome-wide investigation into the underlying genetic architecture of personality traits and overlap with psychopathology"(PMID: 39134740).This variant slightly decreases extraversion with an effect size of -0.03516.

Extraversion

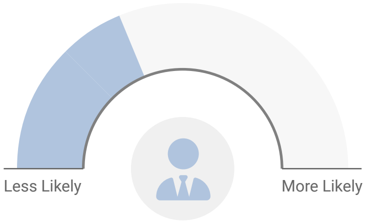

If you have the A/G genotype, this could slightly lower your extraversion.

• **Gene:** Intergenic • **rsID:** rs7606514 • **Your Genotype:** A/A>A/G • **Zygosity:** Heterozygous

Description
Extroversion is personality trait typically characterized by outgoingness, high energy, and/or talkativeness. In general, the term refers to a state of being where someone ?echarges,?or draws energy, from being with other people; the opposite?rawing energy from being alone?s known as introversion

Extra Information
This variant with allele A is identified from the paper "A genome-wide investigation into the underlying genetic architecture of personality traits and overlap with psychopathology"(PMID: 39134740).Shows a slight decrease in extraversion with an effect size of -0.04297.

Extraversion

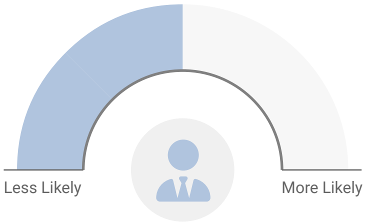

With the C/T genotype, we don't see significant changes in your extraversion trait.

• **Gene:** Intergenic • **rsID:** rs1444978 • **Your Genotype:** T/T>T/C • **Zygosity:** Heterozygous

Description
Extroversion is personality trait typically characterized by outgoingness, high energy, and/or talkativeness. In general, the term refers to a state of being where someone ?echarges,?or draws energy, from being with other people; the opposite?rawing energy from being alone?s known as introversion

Extra Information
This variant with allele T is identified from the paper "A genome-wide investigation into the underlying genetic architecture of personality traits and overlap with psychopathology"(PMID: 39134740).Shows a slight increase in extraversion with an effect size of 0.036666.

Extraversion

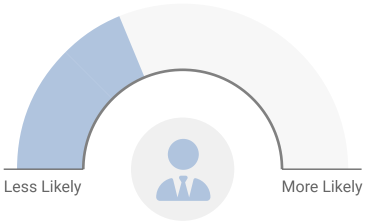

Your G/T genotype could subtly reduce your levels of extraversion.

• **Gene:** Intergenic • **rsID:** rs7739331 • **Your Genotype:** G/G>G/T • **Zygosity:** Heterozygous

Description
Extroversion is personality trait typically characterized by outgoingness, high energy, and/or talkativeness. In general, the term refers to a state of being where someone ?echarges,?or draws energy, from being with other people; the opposite?rawing energy from being alone?s known as introversion

Extra Information
This variant with allele G is identified from the paper "A genome-wide investigation into the underlying genetic architecture of personality traits and overlap with psychopathology"(PMID: 39134740).This variant is associated with a small decrease in extraversion with an effect size of -0.03211.

Extraversion

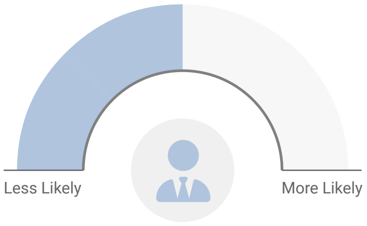

For your Extraversion trait with this genetic variant, the A/A genotype does not exhibit a notable impact.

• **Gene:** Intergenic • **rsID:** rs17688916 • **Your Genotype:** A/A • **Zygosity:** homo_ref

Description

Extroversion is personality trait typically characterized by outgoingness, high energy, and/or talkativeness. In general, the term refers to a state of being where someone ?echarges,?or draws energy, from being with other people; the opposite?rawing energy from being alone?s known as introversion

Extra Information

This variant with allele T is identified from the paper "A genome-wide investigation into the underlying genetic architecture of personality traits and overlap with psychopathology"(PMID: 39134740).Associated with a small decrease in extraversion, with an effect size of _0.04406.

Extraversion

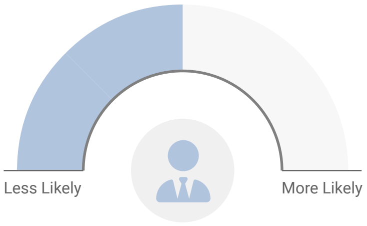

For your Extraversion trait with this genetic variant, the T/T genotype does not exhibit a notable impact.

• **Gene:** WSCD2 • **rsID:** rs3764002 • **Your Genotype:** T/T • **Zygosity:** homo_ref

Description

Extroversion is personality trait typically characterized by outgoingness, high energy, and/or talkativeness. In general, the term refers to a state of being where someone ?echarges,?or draws energy, from being with other people; the opposite?rawing energy from being alone?s known as introversion

Extra Information

This variant with allele C is identified from the paper "A genome-wide investigation into the underlying genetic architecture of personality traits and overlap with psychopathology"(PMID: 39134740).This variant slightly decreases extraversion with an effect size of -0.03928.

Extraversion

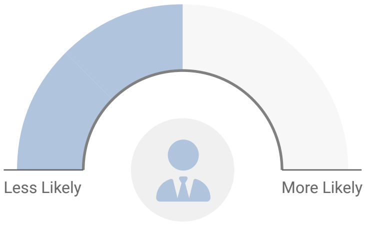

The C/C genotype doesn't show notable effects on your Extraversion trait.

• **Gene:** FAM172A • **rsID:** rs1011501 • **Your Genotype:** C/C • **Zygosity:** homo_ref

Description

Extroversion is personality trait typically characterized by outgoingness, high energy, and/or talkativeness. In general, the term refers to a state of being where someone ?echarges,?or draws energy, from being with other people; the opposite?rawing energy from being alone?s known as introversion

Extra Information

This variant with allele T is identified from the paper "A genome-wide investigation into the underlying genetic architecture of personality traits and overlap with psychopathology"(PMID: 39134740).Associated with a small decrease in extraversion with an effect size of -0.03858.

Extraversion

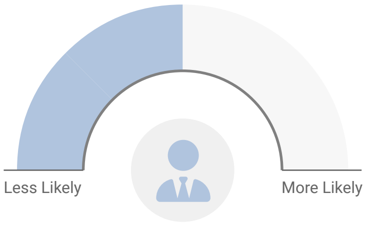

• **Gene:** Intergenic • **rsID:** rs35918640 • **Your Genotype:** AT/AT • **Zygosity:** homo_ref

Description

Extroversion is personality trait typically characterized by outgoingness, high energy, and/or talkativeness. In general, the term refers to a state of being where someone ?echarges,?or draws energy, from being with other people; the opposite?rawing energy from being alone?s known as introversion

Extra Information

This variant with allele A is identified from the paper "A genome-wide investigation into the underlying genetic architecture of personality traits and overlap with psychopathology"(PMID: 39134740).Shows a slight increase in extraversion with an effect size of 0.034989.

Extraversion

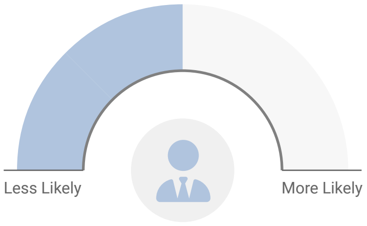

G/G genotype isn't linked to any strong differences in your extraversion trait.

• **Gene:** Intergenic • **rsID:** rs11209774 • **Your Genotype:** G/G • **Zygosity:** homo_ref

Description

Extroversion is personality trait typically characterized by outgoingness, high energy, and/or talkativeness. In general, the term refers to a state of being where someone ?echarges,?or draws energy, from being with other people; the opposite?rawing energy from being alone?s known as introversion

Extra Information

This variant with allele T is identified from the paper "A genome-wide investigation into the underlying genetic architecture of personality traits and overlap with psychopathology"(PMID: 39134740).Associated with a small decrease in extraversion with an effect size of -0.03406.

Neuroticism

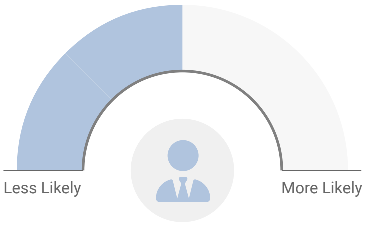

The T/T genotype doesn't appear to influence your level of neuroticism.

• **Gene:** CDH8 • **rsID:** rs6498809 • **Your Genotype:** C/C>T/T • **Zygosity:** Homozygous

Description

Neuroticism is trait that reflects a person's level of emotional stability. It is often defined as a negative personality trait involving negative emotions, poor self-regulation (an inability to manage urges), trouble dealing with stress, a strong reaction to perceived threats, and the tendency to complain

Extra Information

This variant with allele C is identified from the paper "A genome-wide investigation into the underlying genetic architecture of personality traits and overlap with psychopathology"(PMID: 39134740).Associated with a small increase in neuroticism, with an effect size of 0.03672.

Neuroticism

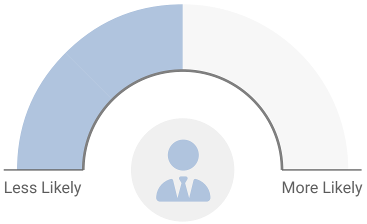

With a C/T genotype, we haven't observed any significant impact on your neuroticism.

• **Gene:** Intergenic • **rsID:** rs2139053 • **Your Genotype:** T/T>T/C • **Zygosity:** Heterozygous

Description

Neuroticism is trait that reflects a person's level of emotional stability. It is often defined as a negative personality trait involving negative emotions, poor self-regulation (an inability to manage urges), trouble dealing with stress, a strong reaction to perceived threats, and the tendency to complain

Extra Information

This variant with allele T is identified from the paper "A genome-wide investigation into the underlying genetic architecture of personality traits and overlap with psychopathology"(PMID: 39134740).Associated with a small increase in neuroticism, with an effect size of 0.042452.

Neuroticism

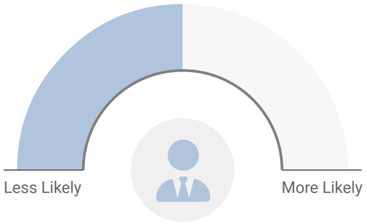

For C/T carriers of this variant, we don't see notable changes in neuroticism according to current research.

• **Gene:** MAD1L1 • **rsID:** rs6948912 • **Your Genotype:** T/T>T/C • **Zygosity:** Heterozygous

Description

Neuroticism is trait that reflects a person's level of emotional stability. It is often defined as a negative personality trait involving negative emotions, poor self-regulation (an inability to manage urges), trouble dealing with stress, a strong reaction to perceived threats, and the tendency to complain

Extra Information

This variant with allele C is identified from the paper "A genome-wide investigation into the underlying genetic architecture of personality traits and overlap with psychopathology"(PMID: 39134740).This variant slightly decreases neuroticism with an effect size of -0.04524.

Name:
DoB:
Gender:
Report ID: WBWG_01_P001_262
Patient ID: 01_P001_262
Date: 6/7/2025

Neuroticism

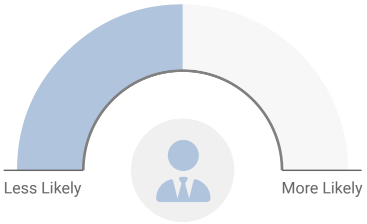

For the A/G genotype, there's no notable change in neuroticism.

• **Gene:** Intergenic • **rsID:** rs7825636 • **Your Genotype:** A/A>A/G • **Zygosity:** Heterozygous

Description

Neuroticism is trait that reflects a person's level of emotional stability. It is often defined as a negative personality trait involving negative emotions, poor self-regulation (an inability to manage urges), trouble dealing with stress, a strong reaction to perceived threats, and the tendency to complain

Extra Information

This variant with allele C is identified from the paper "A genome-wide investigation into the underlying genetic architecture of personality traits and overlap with psychopathology"(PMID: 39134740).Results in a slight increase in neuroticism with an effect size of 0.04446.

Neuroticism

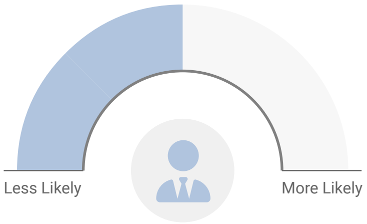

The A/T genotype might slightly increase your neuroticism, but not by much.

• **Gene:** XKR6 • **rsID:** rs2001433 • **Your Genotype:** T/T>T/A • **Zygosity:** Heterozygous

Description

Neuroticism is trait that reflects a person's level of emotional stability. It is often defined as a negative personality trait involving negative emotions, poor self-regulation (an inability to manage urges), trouble dealing with stress, a strong reaction to perceived threats, and the tendency to complain

Extra Information

This variant with allele T is identified from the paper "A genome-wide investigation into the underlying genetic architecture of personality traits and overlap with psychopathology"(PMID: 39134740).This variant slightly increases neuroticism with an effect size of 0.046409.

Neuroticism

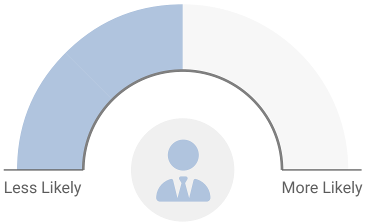

Having C/C for the TSNARE1 gene suggests no significant effect on your neuroticism trait.

• **Gene:** TSNARE1 • **rsID:** rs4129585 • **Your Genotype:** A/A>C/C • **Zygosity:** Homozygous

Description

Neuroticism is trait that reflects a person's level of emotional stability. It is often defined as a negative personality trait involving negative emotions, poor self-regulation (an inability to manage urges), trouble dealing with stress, a strong reaction to perceived threats, and the tendency to complain

Extra Information

This variant with allele A is identified from the paper "A genome-wide investigation into the underlying genetic architecture of personality traits and overlap with psychopathology"(PMID: 39134740).This variant is associated with a small decrease in neuroticism scores, with an effect size of -0.05241.

Neuroticism

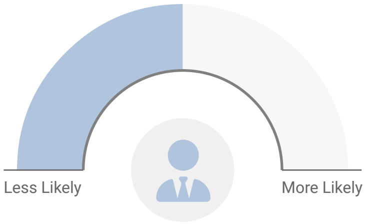

G/G at this intergenic spot does not present a notable effect on neuroticism.

• **Gene:** Intergenic • **rsID:** rs574307253 • **Your Genotype:** G/G • **Zygosity:** homo_ref

Description

Neuroticism is trait that reflects a person's level of emotional stability. It is often defined as a negative personality trait involving negative emotions, poor self-regulation (an inability to manage urges), trouble dealing with stress, a strong reaction to perceived threats, and the tendency to complain

Extra Information

This variant with allele A is identified from the paper "A genome-wide investigation into the underlying genetic architecture of personality traits and overlap with psychopathology"(PMID: 39134740).Associated with a small increase in neuroticism, with an effect size of 0.059817.

Neuroticism

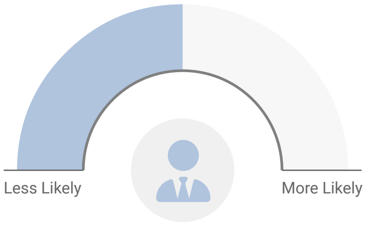

The A/A genotype for the NSF gene variant doesn’t show a notable effect on neuroticism.

• **Gene:** NSF • **rsID:** rs116956554 • **Your Genotype:** A/A • **Zygosity:** homo_ref

Description

Neuroticism is trait that reflects a person's level of emotional stability. It is often defined as a negative personality trait involving negative emotions, poor self-regulation (an inability to manage urges), trouble dealing with stress, a strong reaction to perceived threats, and the tendency to complain

Extra Information

This variant with allele G is identified from the paper "A genome-wide investigation into the underlying genetic architecture of personality traits and overlap with psychopathology"(PMID: 39134740).Shows a slight increase in neuroticism with an positive effect size of 0.057427.

Neuroticism

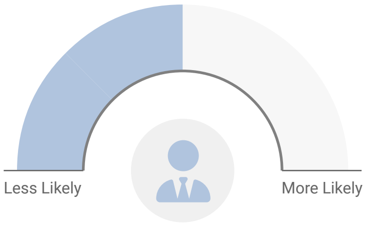

Having C/C means no noticeable change in emotional stability.

• **Gene:** BMAL1 • **rsID:** rs7396943 • **Your Genotype:** C/C • **Zygosity:** homo_ref

Description

Neuroticism is trait that reflects a person's level of emotional stability. It is often defined as a negative personality trait involving negative emotions, poor self-regulation (an inability to manage urges), trouble dealing with stress, a strong reaction to perceived threats, and the tendency to complain

Extra Information

This variant with allele G is identified from the paper "A genome-wide investigation into the underlying genetic architecture of personality traits and overlap with psychopathology"(PMID: 39134740).Associated with a small increase in neuroticism, with an effect size of 0.046283.

Neuroticism

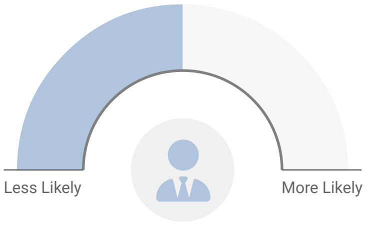

The T/T genotype doesn't strongly correlate with neuroticism changes based on current data.

• **Gene:** Intergenic • **rsID:** rs615632 • **Your Genotype:** T/T • **Zygosity:** homo_ref

Description

Neuroticism is trait that reflects a person's level of emotional stability. It is often defined as a negative personality trait involving negative emotions, poor self-regulation (an inability to manage urges), trouble dealing with stress, a strong reaction to perceived threats, and the tendency to complain

Extra Information

This variant with allele C is identified from the paper "A genome-wide investigation into the underlying genetic architecture of personality traits and overlap with psychopathology"(PMID: 39134740).This variant slightly increases neuroticism with an effect size of 0.040914.

Neuroticism

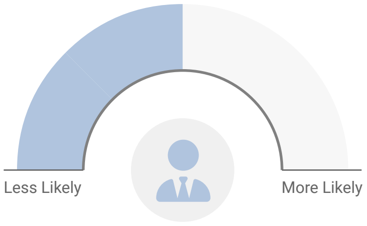

For the T/T genotype, there's no indication of significant influence on neuroticism.

• **Gene:** TSNARE1 • **rsID:** rs117713019 • **Your Genotype:** T/T • **Zygosity:** homo_ref

Description

Neuroticism is trait that reflects a person's level of emotional stability. It is often defined as a negative personality trait involving negative emotions, poor self-regulation (an inability to manage urges), trouble dealing with stress, a strong reaction to perceived threats, and the tendency to complain

Extra Information

This variant with allele C is identified from the paper "A genome-wide investigation into the underlying genetic architecture of personality traits and overlap with psychopathology"(PMID: 39134740).Shows a moderate increase in neuroticism with an effect size of 0.08952.

Name: Report ID: WBWG_01_P001_262
DoB: Patient ID: 01_P001_262
Gender: Date: 6/7/2025

Openness

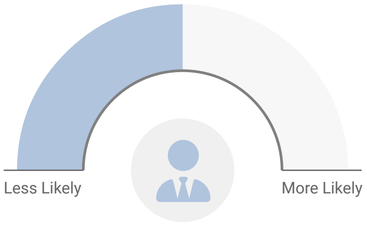

Possessing A/T for rs6725323 doesn't appear to affect your engagement with new experiences.

• **Gene:** CLIP4 • **rsID:** rs6725323 • **Your Genotype:** A/A>A/T • **Zygosity:** Heterozygous

Description

Openness to experience, or simply openness, is a basic?ersonality?rait denoting receptivity to new ideas and new experiences. It is one of the five core personality dimensions that drive behavior?nown as the?ive-factor model?f personality, or the Big 5. People with high levels of openness are more likely to seek out a variety of experiences, be comfortable with the unfamiliar, and pay?ttention?o their inner feelings more than those who are less open to novelty. They tend to exhibit high levels of curiosity and often enjoy being surprised. People with low levels of openness prefer familiar routines, people, and ideas; they can be perceived as closed-minded"

Extra Information

This variant with allele A is identified from the paper "A genome-wide investigation into the underlying genetic architecture of personality traits and overlap with psychopathology"(PMID: 39134740).Shows a slight decrease in openness with an effect size of -0.03361.

Openness

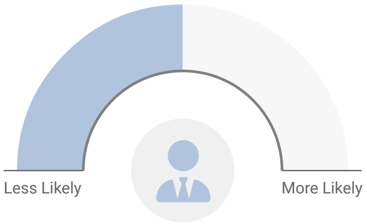

The C/T genotype doesn't significantly sway your openness.

• **Gene:** Intergenic • **rsID:** rs919013 • **Your Genotype:** T/T>T/C • **Zygosity:** Heterozygous

Description

Openness to experience, or simply openness, is a basic?ersonality?rait denoting receptivity to new ideas and new experiences. It is one of the five core personality dimensions that drive behavior?nown as the?ive-factor model?f personality, or the Big 5. People with high levels of openness are more likely to seek out a variety of experiences, be comfortable with the unfamiliar, and pay?ttention?o their inner feelings more than those who are less open to novelty. They tend to exhibit high levels of curiosity and often enjoy being surprised. People with low levels of openness prefer familiar routines, people, and ideas; they can be perceived as closed-minded"

Extra Information

This variant with T is identified from the paper "A genome-wide investigation into the underlying genetic architecture of personality traits and overlap with psychopathology"(PMID: 39134740).This variant slightly increases openness with an effect size of 0.033444.

Openness

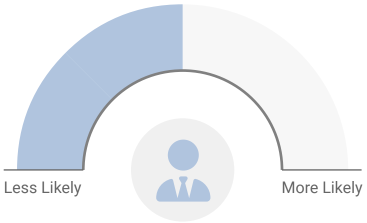

With an A/C genotype for openness and variant rs11996715, the data doesn't suggest strong correlations currently.

• **Gene:** Intergenic • **rsID:** rs11996715 • **Your Genotype:** C/C>C/A • **Zygosity:** Heterozygous

Description

Openness to experience, or simply openness, is a basic?ersonality?rait denoting receptivity to new ideas and new experiences. It is one of the five core personality dimensions that drive behavior?nown as the?ive-factor model?f personality, or the Big 5. People with high levels of openness are more likely to seek out a variety of experiences, be comfortable with the unfamiliar, and pay?ttention?o their inner feelings more than those who are less open to novelty. They tend to exhibit high levels of curiosity and often enjoy being surprised. People with low levels of openness prefer familiar routines, people, and ideas; they can be perceived as closed-minded"

Extra Information

This variant with allele C is identified from the paper "A genome-wide investigation into the underlying genetic architecture of personality traits and overlap with psychopathology"(PMID: 39134740).Associated with a small increase in openness with an effect size of 0.030703.

Openness

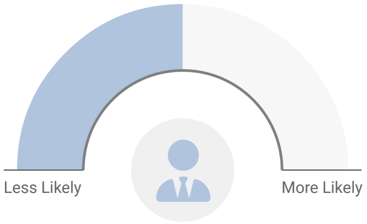

• **Gene:** Intergenic • **rsID:** rs61689447 • **Your Genotype:** GTT/GTT>[illegible] • **Zygosity:** Heterozygous

Description

Openness to experience, or simply openness, is a basic?ersonality?rait denoting receptivity to new ideas and new experiences. It is one of the five core personality dimensions that drive behavior?nown as the?ive-factor model?f personality, or the Big 5. People with high levels of openness are more likely to seek out a variety of experiences, be comfortable with the unfamiliar, and pay?ttention?o their inner feelings more than those who are less open to novelty. They tend to exhibit high levels of curiosity and often enjoy being surprised. People with low levels of openness prefer familiar routines, people, and ideas; they can be perceived as closed-minded"

Extra Information

This variant with deletions of one or more T allele is identified from the paper "A genome-wide investigation into the underlying genetic architecture of personality traits and overlap with psychopathology"(PMID: 39134740).This variant slightly decreases openness with an effect size of -0.03312.

Openness

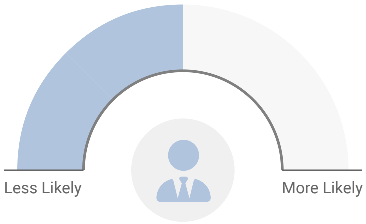

No significant effect on your openness from having the C/C genotype.

• **Gene:** C11orf80 • **rsID:** rs7570 • **Your Genotype:** C/C • **Zygosity:** homo_ref

Description

Openness to experience, or simply openness, is a basic?ersonality?rait denoting receptivity to new ideas and new experiences. It is one of the five core personality dimensions that drive behavior?nown as the?ive-factor model?f personality, or the Big 5. People with high levels of openness are more likely to seek out a variety of experiences, be comfortable with the unfamiliar, and pay?ttention?o their inner feelings more than those who are less open to novelty. They tend to exhibit high levels of curiosity and often enjoy being surprised. People with low levels of openness prefer familiar routines, people, and ideas; they can be perceived as closed-minded"

Extra Information

This variant with allele G is identified from the paper "A genome-wide investigation into the underlying genetic architecture of personality traits and overlap with psychopathology"(PMID: 39134740).This variant slightly decreases openness with an effect size of -0.04711.

Openness

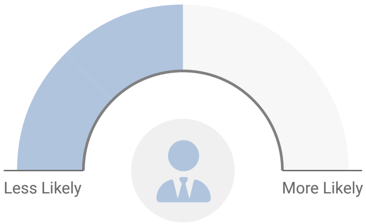

Having the G/G genotype doesn't notably affect your openness.

• **Gene:** Intergenic • **rsID:** rs117890891 • **Your Genotype:** G/G • **Zygosity:** homo_ref

Description

Openness to experience, or simply openness, is a basic?ersonality?rait denoting receptivity to new ideas and new experiences. It is one of the five core personality dimensions that drive behavior?nown as the?ive-factor model?f personality, or the Big 5. People with high levels of openness are more likely to seek out a variety of experiences, be comfortable with the unfamiliar, and pay?ttention?o their inner feelings more than those who are less open to novelty. They tend to exhibit high levels of curiosity and often enjoy being surprised. People with low levels of openness prefer familiar routines, people, and ideas; they can be perceived as closed-minded"

Extra Information

This variant with allele T is identified from the paper "A genome-wide investigation into the underlying genetic architecture of personality traits and overlap with psychopathology"(PMID: 39134740).Shows a significant increase in openness with an effect size of 0.145052.

Openness

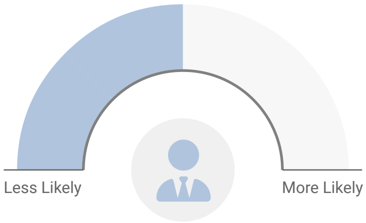

Possessing a T/T genotype at this variant seems to leave your openness unchanged.

• **Gene:** Intergenic • **rsID:** rs6996198 • **Your Genotype:** T/T • **Zygosity:** homo_ref

Description

Openness to experience, or simply openness, is a basic?ersonality?rait denoting receptivity to new ideas and new experiences. It is one of the five core personality dimensions that drive behavior?nown as the?ive-factor model?f personality, or the Big 5. People with high levels of openness are more likely to seek out a variety of experiences, be comfortable with the unfamiliar, and pay?ttention?o their inner feelings more than those who are less open to novelty. They tend to exhibit high levels of curiosity and often enjoy being surprised. People with low levels of openness prefer familiar routines, people, and ideas; they can be perceived as closed-minded"

Extra Information

This variant with allele C is identified from the paper "A genome-wide investigation into the underlying genetic architecture of personality traits and overlap with psychopathology"(PMID: 39134740).Associated with a small decrease in openness with an effect size of -0.04602.

Genetic Terms Glossary

Understanding Your Genetic Report

This glossary provides definitions for common genetic terms used throughout your report. Understanding these terms will help you better comprehend your genetic findings.

Carrier Status

When a person has one copy of a genetic variant associated with a recessive condition but typically does not have the condition themselves.

Dominant Inheritance

A pattern where a genetic trait or disorder can be passed on to offspring even if only one parent has the variant.

DNA (Deoxyribonucleic Acid)

The molecule that carries genetic instructions for the development and functioning of all known living organisms.

Gene

A segment of DNA that contains the instructions for making a specific protein or determines a particular characteristic.

Recessive Inheritance

A pattern where a genetic trait or disorder only appears when a person has two copies of the variant, one from each parent.

Exome

The part of the genome that contains protein-coding sequences. Most known disease-causing variants are found in the exome.

Genetic Variant

A change in the DNA sequence compared to a reference genome. Variants can be benign, pathogenic, or of uncertain significance.

Pharmacogenetics

The study of how genetic differences influence individual responses to medications.

Chromosome

A structured package of DNA found in the nucleus of cells. Humans typically have 23 pairs of chromosomes.

Pathogenic Variant

A genetic change that is known to increase the risk of disease or cause a specific condition.

Benign Variant

A genetic change that is not known to cause disease or increase health risks.

VUS (Variant of Uncertain Significance)

A genetic change where there is not enough evidence to classify it as either pathogenic or benign.

Penetrance

The proportion of people with a particular genetic variant who exhibit the associated trait or condition.

Phenotype

The observable physical or biochemical characteristics of an organism, as determined by both genetic makeup and environmental influences.

Genotype

The genetic makeup of an organism, which determines traits in combination with environmental factors.

Technical Notes and Methodology

DNA Sequencing Technology

Our whole genome sequencing is performed using Illumina's NovaSeq™ 6000 Sequencing System, representing the pinnacle of next-generation sequencing technology. This platform utilizes sequencing by synthesis (SBS) technology, capable of generating up to 6 terabases of data per run with exceptional accuracy. We maintain an average coverage depth of 30x across the genome, ensuring high-quality variant calling and minimal false positives. The system's advanced patterned flow cell technology and two-channel chemistry significantly reduce noise and cross-talk between bases, resulting in superior base calling accuracy.

Our DNA extraction and library preparation protocols follow industry-leading standards, incorporating quality controls at each step. We use the Illumina DNA PCR-Free Prep kit to minimize PCR-induced artifacts and biases, ensuring a more uniform coverage across the genome, including traditionally difficult-to-sequence regions rich in GC content.

Bioinformatics Pipeline

Raw sequencing data undergoes rigorous quality control using FastQC, followed by adapter trimming and quality filtering. Reads are aligned to the GRCh38/hg38 reference genome using BWA-MEM2, chosen for its superior performance with long reads and improved accuracy in repetitive regions. Post-alignment processing includes duplicate marking, base quality score recalibration, and local realignment around indels.

Variant calling is performed using an ensemble approach combining GATK HaplotypeCaller, DeepVariant, and Strelka2, leveraging the strengths of each caller. This multi-caller strategy, combined with sophisticated filtering and machine learning-based recalibration, achieves high sensitivity while maintaining a low false discovery rate. Structural variants are detected using a combination of Manta, DELLY, and CNVnator, providing comprehensive coverage of different variant types.

Variant Annotation and Classification

Variants are extensively annotated using Ensembl's Variant Effect Predictor (VEP), incorporating SIFT, PolyPhen-2, and other in silico prediction tools. Our custom annotation pipeline integrates data from multiple sources including gnomAD for population frequencies, ClinVar for clinical significance, and COSMIC for somatic mutations.

Variant classification follows ACMG/AMP guidelines, incorporating evidence from multiple databases and literature sources. We utilize a sophisticated scoring system that weighs different types of evidence, including:
• Population databases (gnomAD, 1000 Genomes)
• Disease databases (ClinVar, OMIM, HGMD)
• Functional studies from published literature
• Computational predictions
• Segregation data when available

Reference Databases

Our analysis incorporates data from numerous authoritative sources:

• Population Databases:
- gnomAD (>140,000 individuals)
- 1000 Genomes Project (2,504 individuals)
- UK Biobank (500,000 participants)

• Clinical Databases:
- ClinVar (NIH-curated clinical variants)
- OMIM (Online Mendelian Inheritance in Man)
- HGMD (Human Gene Mutation Database)

Technical Limitations and Considerations

While our sequencing and analysis pipeline represents current best practices, it's important to acknowledge certain limitations:

- Some genomic regions may have suboptimal coverage due to technical limitations
- Complex structural variants may be more challenging to detect accurately
- Novel variants may have limited available evidence for classification
- Some regions of the genome remain difficult to sequence or interpret
- Certain types of mutations (e.g., some repeat expansions) may not be reliably detected

We continuously update our methodologies as new technologies and knowledge become available, always striving to provide the most accurate and comprehensive genetic analysis possible.

SZA Longevity
A healthy future awaits you

Name: Report ID: WBWG_01_P001_262
DoB: Patient ID: 01_P001_262
Gender: Date: 6/7/2025

Gene List

American College of Medical Genetics and Genomics (Secondary Finding List v3.1) (ACMG Genes)

ACTA2, ACTC1, ACVRL1, APC, APOB, ATP7B, BMPR1A, BRCA1, BRCA2, BTD, CACNA1S, CASQ2, COL3A1, DSC2, DSG2, DSP, DSP, ENG, FBN1, FLNC, GAA, GLA, HFE, HNF1A, KCNH2, KCNQ1, LDLR, LMNA, MAX, MEN1, MLH1, MSH2, MSH6, MUTYH, MYBPC3, MYH11, MYH7, MYH7, MYL2, MYL3, NF2, OTC, PALB2, PCSK9, PKP2, PMS2, PRKAG2, PTEN, RB1, RET, RET, RET, RPE65, RYR1, RYR2, SCN5A, SCN5A, SCN5A, SDHAF2, SDHB, SDHC, SDHD, SMAD3, SMAD4, SMAD4, STK11, TGFBR1, TGFBR2, TMEM127, TMEM43, TNNI3, TNNT2, TNNT2, TP53, TPM1, TRDN, TSC1, TSC2, TTN, VHL, WT1, BAG3, DES, RBM20, TNNC1, TTR

Cardiovascular Diseases (219 Genes)

ACTA2, ACTC1, APOB, BAG3, CASQ2, COL3A1, DES, DSC2, DSG2, DSP, FBN1, FLNC, GLA, KCNH2, KCNQ1, LDLR, LMNA, MYBPC3, MYH11, MYH7, MYL2, MYL3, PCSK9, PKP2, PRKAG2, RBM20, RYR2, SCN5A, SMAD3, TGFBR1, TGFBR2, TMEM43, TNNC1, TNNI3, TNNT2, TPM1, TRDN, TTN, ABCC6, ABCC9, ACTN2, ACVRL1, ADD1, AGT, AGTR1, AKAP9, ALG10B, ATP13A3, ATP1A2, ATP1B1, BMPR2, CACNA1A, CACNA1C, CACNB2, CAV1, CAV3, CFC1, CRYAB, CSRP3, CYP3A5, DPP6, ECE1, EDNRA, EIF2AK4, ENG, ENPP1, ESR1, EYA4, FKTN, FOXF1, GATA4, GDF1, GDF2, GJA5, GNAI2, GNAQ, GNB3, GPD1L, HCN4, HTRA1, JPH2, KCNA5, KCNE1, KCNE2, KCNE3, KCNJ2, KCNJ5, KCNK18, KCNK3, LAMA4, LDB3, LOX, MFAP5, MYH6, MYLK2, MYOZ2, NEXN, NOS3, NOTCH1, NPPA, PLN, PRKG1, PSEN2, PTGIS, RGS5, ROBO4, SCN1A, SCN1B, SCN3B, SCN4B, SDHA, SGCD, SMAD4, SMAD6, SMAD9, SNTA1, TAFAZZIN, TBX20, TCAP, TGFB2, TLL1, TNF, VCL, ACE, ADRB1, ALPK3, ANGPT1, ANKRD1, APOA2, BAG5, CALM3, CDH2, CFAP45, CFAP52, CIROP, COL4A1, COL5A1, COLGALT1, DZIP1, EPHB4, EPHX2, FHOD3, GATA5, GHR, HAAO, HS3ST6, KIF20A, KNG1, KYNU, LMOD2, MNS1, MYLK, MYOF, NADSYN1, NOTCH3, PLG, PPCS, PPP1CB, PPP1R17, PRDM6, PSEN1, RNF213, RPL3L, SMAD2, TBX1, THSD1, THSD4, TMPO, ACVR2B, ADA2, CALM1, CALM2, CCDC174, CFAP53, CITED2, COL1A2, COL4A2, CORIN, CRELD1, CTNNA3, DCHS1, F12, FLNA, GATAD1, GJA1, GLMN, JAG1, KCND3, LRP6, MED13L, MEF2A, MGAT2, MMP21, MYL4, MYPN, NKX2-5, NKX2-6, NODAL, NR2F2, NT5E, NUP155, PKD1L1, PRPH2, PUF60, RAF1, RBM10, SCN2B, SCN9A, SERPING1, SHOC2, STOX1, TAB2, TECRL, TEK, TFAP2B, TGFB3, TKT, TNNI3K, ZFPM2

Endocrine and Metabolic Disorders (691 Genes)

BTD, GAA, OTC, ABCA1, ABCC8, ACADM, ACADVL, ACAT1, AGL, AGPAT2, AGXT, ALDH18A1, ALDOA, ALG1, ALG11, ALG12, ALG2, ALG3, ALG6, ALG8, ALG9, ANOS1, APOA1, APOA2, APOA5, APOC2, APOE, ARG1, ASL, ASS1, ATAD1, ATP13A2, AUH, B4GALT1, BCKDHA, BCKDHB, BMP2, BSCL2, BTK, C19orf12, CACNA1S, CHD7, CIDEC, CLCN2, CLN3, CLN5, CLN6, CLN8, CLPB, COG1, COG4, COG5, COG6, COG7, COG8, CP, CPS1, CPT1A, CPT2, CTNS, CTSD, CTSF, CYP11B1, CYP17A1, CYP21A2, DBT, DCAF17, DDB2, DLL3, DNAJC19, DNAJC5, DOLK, DPAGT1, DPM1, DPM3, DUOX2, ELN, ENO3, EPHX2, ERCC2, ERCC3, ERCC4, ERCC5, ERCC6, ERCC8, ETFA, ETFB, ETFDH, FA2H, FAH, FBLN5, FCSK, FGF8, FGFR1, FTH1, FTL, FUT8, G6PC1, GALE, GALK1, GALT, GAMT, GATM, GBE1, GCDH, GCH1, GCK, GH1, GHR, GLB1, GLRA1, GLRB, GLUD1, GNAS, GNAS-AS1, GNE, GPHN, GPIHBP1, GRHPR, GRN, GYG1, HADH, HADHA, HADHB, HAMP, HES7, HFE, HJV, HLCS, HMGCL, HOGA1, HPRT1, HSD3B2, HTRA2, HYMAI, IDUA, INS, INSR, IVD, IYD, KCNJ11, KCNJ5, KRT5, LARS1, LCAT, LDHA, LDLR, LDLRAP1, LEP, LEPR, LFNG, LIPE, LMBRD1, LMNA, LPL, LRMDA, MAN2C1, MARS1, MC1R, MCCC1, MCCC2, MCEE, MESP2, MFSD8, MGAT2, MMAA, MMAB, MMACHC, MMADHC, MMUT, MOCOS, MOCS1, MOCS2, MOGS, MPDU1, MPI, MTR, MTRR, NAGS, NEU1, NGLY1, NPC1, NPC2, NUDT15, OPA3, Oca.02, PAH, PANK2, PAX8, PCCA, PCCB, PCSK1, PDE10A, PDE8B, PDX1, PFKM, PGAM2, PGM1, PHKA1, PHKA2, PHKB, PHKG2, PLA2G6, PLIN1, PLPBP, PMM2, POFUT1, POGLUT1, POLH, POMC, PPARG, PPP1R17, PPT1, PRDX1, PROK2, PROKR2, PSAP, PYGM, QDPR, RFT1, SCN4A, SDHA, SDHAF2, SDHB, SDHC, SDHD, SERAC1, SLC10A1, SLC16A1, SLC17A5, SLC22A12, SLC22A5, SLC25A13, SLC25A20, SLC2A1, SLC2A9, SLC35A1, SLC35C1, SLC37A4, SLC40A1, SLC45A2, SLC5A2, SLC6A5, SLC6A8, SMPD1, SRD5A3, SRY, STAR, STX16, TAFAZZIN, TFR2, TIMM50, TJP2, TPO, TPP1, TSHB, TSHR, TYR, TYRP1, VDR, WDR45, XDH, XPA, XPC, ZFP57, ABCB4, ABCD4, ACOX1, ADCY10, ADRB2, ADRB3, AGRP, ALG13, ALKBH8, ALPL, AMPD3, ANGPTL3, APOB, APPL1, AQP7, ARSK, ATP1A1, ATP6AP2, AXL, BCAT2, BCHE, BNC1, C12orf4, C14orf39, C18orf32, CAMK2A, CAPN10, CARTPT, CCDC141, CCR5, CES1, CLDN16, CLPX, COA7, CRAT, CREB3L3, CTH, CTSA, CYP2C8, DBH, DCT, DDOST, DIO1, DPM2, DPYS, DUSP6, EDEM3, EGR2, EIF3F, ENPP1, ERCC1, ESR2, F9, FEZF1, FGF17, FSHR, FTCD, GALM, GALNT2, GATA1, GBA1, GCM2, GCSH, GDF9, GGT1, GHRL, GLS, GNRH1, GPAA1, GPNMB, GRIA1, HAL, HCFC1, HNF4A, HSF2BP, IL2RA, IMPDH2, IRS4, KCNMB1, KDM5B, KRT18, LCT, LDHD, LHCGR, LINGO1, LRP5, MAFA, MAGT1, MAX, MCM6, METTL5, MPZ, MRPS22, MSH2, MSH4, MSH5, MSH6, MTHFS, NAA20, NAXD, NDNF, NHLH2, NKX2-5, NR0B2, NUP107, ODC1, PANK4, PAX4, PCK2, PCSK9, PEX7, PIDD1, PIGB, PIGC, PIGH, PIGK, PIGP, PIGQ, PIGS, PMP22, PMS2, PRKACA, PRX, PTH, REPS1, RINT1, RSRC1, RUSC2, SDC3, SEMA3E, SERPINA7, SLC28A1, SLC36A2, SLC6A19, SLC6A20, SLCO1B1, SLCO1B3, SMAD6, SNCA, SNCB, SOHLH1, SPIDR, SPRY4, SQOR, SRA1, SSR4, STT3A, SUGCT, SUMO4, TAF13, TBC1D4, TBL1X, TCF12, TDO2, THAP11, TMEM165, TMLHE, TPH2, TPMT, TREH, TRH, TRHR, TRMT1, TRPV6, TUSC3, TXNRD2, UCP3, USF1, UVSSA, WDR11, XRCC2, ZBTB11, ZNF143, ZSWIM7, A2M, AARS1, AASS, ABAT, ABCB11, ABCD3, ABHD5, ACADSB, ACOX2, ACP2, ACY1, ADSL, AFP, AKT2, ALAD, ALAS2, ALB, ALDH4A1, ALDH6A1, AMACR, AMH, AMHR2, ANKH, AP2S1, AP5Z1, APC2, APOC3, APRT, ARSL, ASAH1, ATL1, ATL3, ATP6V0A2, ATP6V1A, ATP6V1E1, ATP8B1, BCO1, BLK, BLVRA, BMP15, BPGM, BPNT2, CACNA1A, CACNA1H, CAD, CAT, CAV1, CC2D1A, CCDC115, CD320, CEL, CEP19, CERS1, CETP, CHST3, CNNM2, COASY, COG2, CRADD, CRBN, CTLA4, CYP11B2, CYP19A1, CYP24A1, CYP2R1, CYP7B1, DCDC2, DCPS, DCXR, DHCR24, DHTKD1, DIAPH2, DMGDH, DNAJC12, DNMT1, DPYD, DUOXA2, EBP, ECM1, EDC3, EEF1A2, EGF, ELOVL5, ELP2, EXOSC3, EXOSC8, EXT2, FBXO31, FECH, FGF12, FIGLA, FMN2, FMR1, FOLR1, FOXL2, FSHB, FXYD2, GABRB1, GCLC, GK, GLI3, GLUL, GLYCTK, GM2A, GNA11, GNMT, GNRHR, GOT1, GPD1, GPX4, GRID2, GRIK2, GRIN2D, GRM1, GYS1, H6PD, HCN1, HFM1, HIBCH, HMGCS2, HMOX1, HNMT, HSD11B1, HYAL1, IDH2, IL31RA, IMPA1, ITPA, KCNA2, KCNB1, KCNJ6, KHK, KISS1, KISS1R, KLF11, KYNU, L2HGDH, LBR, LDHB, LHB, LINS1, LIPC, LMAN2L, LTC4S, MAN1B1, MAOA, MBOAT7, MCM8, MCM9, MED23, METTL23, MKRN3, MLH1, MSMO1, NADK2, NBAS, NDST1, NECAP1, NEUROD1, NOBOX, NR3C1, NSUN2, NUS1, OPLAH, PAPSS2, PCK1, PCNA, PDE11A, PEPD, PEX1, PEX6, PGAP1, PGK1, PIGN, PIGT, PNLIP, PNPLA2, POF1B, PPM1K, PPOX, PRKAR1A, PRKCSH, PRLR, PRODH, PRPS1, PRSS12, PSMC3IP, PYCR1, RAD50, RBCK1, RIPPLY2, RSPO1, SAR1B, SARDH, SC5D, SCP2, SEC63, SECISBP2, SERPINA6, SHPK, SLC16A12, SLC1A1, SLC1A3, SLC1A4, SLC24A5, SLC25A19, SLC26A2, SLC30A10, SLC34A1, SLC35A3, SLC39A14, SLC4A4, SLC52A1, SLC6A17, SLC6A9, SPINK1, SPTLC1, SPTLC2, SQSTM1, SRD5A2, ST3GAL3, ST3GAL5, STAG3, STT3B, SUMF1, SYCE1, TAC3, TALDO1, TBXAS1, TDP1, TECR, TG, THRB, TMEM199, TNIK, TPK1, TRAPPC9, TRPM6, TSPYL1, TTR, UBA5, UGT1A1, UPB1, UROC1, VAC14, VPS33A, VRK1, WASHC4, WNT4, WWOX, ZC3H14, ZMPSTE24

Hematologic Disorder (176 Genes)

ABCD4, ANK1, C3, CD46, CDAN1, CDIN1, CFB, CFH, CFHR1, CFHR3, CFHR4, CFI, CYB5A, CYB5R3, CYBA, CYBB, ELANE, EPB41, EPB42, F10, F5, F8, F9, G6PC3, GATA1, GFI1, HAMP, HAX1, HBA1, HBA2, HBB, HBB-LCR, HJV, JAGN1, KIF23, KLF1, KLKB1, LMAN1, LMBRD1, MCFD2, MMACHC, MMADHC, NCF1, NCF2, NCF4, PIK3CD, PIK3R1, PRDX1, PROC, PROS1, RPL11, RPL15, RPL18, RPL26, RPL27, RPL35, RPL35A, RPL5, RPS10, RPS15A, RPS17, RPS19, RPS24, RPS26, RPS27, RPS28, RPS29, RPS7, SEC23B, SERPINC1, SLC11A2, SLC4A1, SPTA1, SPTB, STEAP3, THBD, TMPRSS6, TSR2, VPS45, WAS, ATP11C, ATRX, BCAM, CALR, CASK, CLPB, CYBC1, EPHB2, EPX, F7, FGA, FGB, FGG, FLI1, FYB1, GP1BA, GP1BB, GP9, GSR, HABP2, HBG1, HBG2, HOXA11, HSCB, IKZF5, IL6R, IL6ST, ITGA2B, ITGB3, MECOM, PLA2G4A, PLAT, RACGAP1, RHCE, RHD, SH2B3, SH3KBP1, SLC39A7, SMARCD2, SPI1, TBXA2R, TCF3, TERT, TNFRSF13B, TUBA8, VWF, ZNF341, ABCB6, AICDA, AK1, ANO6, CD36, CD40, CD59, CDC42, CEBPE, CYCS, DHFR, F12, FCGR2C, FTL, GFI1B, GGCX, GP6, GPI, HRG, HSPA9, IFNG, IKBKG, JAK2, KCNN4, KNG1, MPL, MPO, NBEAL2, NT5C3A, P2RY12, PIEZO1, PIGM, PLAU, PRKACG, RAC2, RASGRP2, RHAG, RPIA, SERPIND1, SERPINE1, SERPINF2, SLFN14, STIM1, THPO, TPI1, TUBB1, UMPS, UNG, VKORC1

Immune Disorders (204 Genes)

ADA, CTLA4, FGA, FGB, FGG, FLG, GSS, HLA-DQA1, HLA-DQB1, IL2RG, MYO9B, NLRC4, NLRP12, NLRP3, NPC1, NPC2, PLCG2, PRF1, SH2D1A, STX11, STXBP2, UNC13D, XIAP, ABCB1, ANGPT2, ARHGEF1, BACH2, C1QA, C1QB, C1QC, CALCRL, CARD10, CARD11, CARD8, CARMIL2, CCL11, CCL2, CCL3, CCL3L1, CCL5, CCR5, CD209, CD3G, CD4, CD70, CELSR1, CTNNBL1, CX3CR1, CXCL12, CXCR1, DEF6, EPHB4, F10, FCHO1, FNIP1, GINS1, GPX1, HLA-C, IFIH1, IFITM3, IFNAR1, IFNG, IFNGR1, IFNGR2, IFNL3, IKBKB, IKBKG, IKZF3, IL10, IL21R, IL23R, IL37, IL4R, INAVA, IRF3, IRF9, IVNS1ABP, KIR3DL1, KNSTRN, LAT, LCP2, LIG1, LTA, MBL2, MCM10, MPEG1, MRTFA, MS4A2, NCKAP1L, PIK3CD, PIK3CG, PLA2G7, POLR3F, POMP, PRNP, PSMB10, PSMB4, PSMB9, PSMG2, PTPRC, RAC2, RASGRP1, RC3H1, REL, RELB, RIPK1, SASH3, SLC11A1, SLC29A3, SPPL2A, SYK, TAP1, TAPBP, TBX21, TET2, TICAM1, TIE1, TLR1, TLR2, TLR3, TLR7, TLR8, TOM1, TPP2, TRAF3, UNC93B1, ZNFX1, ATG16L1, ATP2C1, ATP6AP1, B2M, BCL10, BCL11B, C1S, C4A, C4B, CARD14, CARD9, CD247, CD27, CD8A, CIITA, CORO1A, DNASE1L3, DOCK2, EPG5, FADD, FBN1, FCGR3A, FCN3, FLT4, GATA2, GJC2, IFNAR2, IGKC, IL12B, IL12RB1, IL17F, IL17RA, IL17RC, IL2RA, IL6, IRF5, IRF7, IRF8, IRGM, ISG15, ITK, LBR, LCK, MAGT1, MALT1, MASP2, MCM4, MSN, MYD88, NOD2, ORAI1, OTULIN, PIEZO1, PSMB8, PSTPIP1, RAG1, RFX5, RFXAP, RORC, RPSA, SERPING1, STAT1, STAT2, STIM1, STK4, TAP2, TFRC, TNFRSF1A, TNFRSF4, TRAC, TRAF3IP2, TYK2, UNC119, VEGFC, WIPF1, ZAP70

Neurological Disorders (1145 Genes)

AARS1, ABCA4, ABCA7, ACTG1, ADCY1, ADH1C, AIFM1, AIPL1, ALS2, AMPD2, ANG, ANO5, APOE, APP, ARHGEF9, ARMS2, ARX, ASPM, ATAD1, ATP13A2, ATP1A3, ATP2B2, ATXN2, ATXN7, ATXN8OS, BSCL2, C3, CACNA1A, CACNA1F, CACNA1H, CACNB4, CAPN3, CAV3, CC2D2A, CCDC50, CCDC88C, CCM2, CDC14A, CDH23, CDK5RAP2, CDK6, CENPE, CENPJ, CEP135, CEP152, CEP290, CFH, CFHR1, CFHR3, CHMP1A, CHRNA2, CHRNA4, CHRNB2, CIB2, CISD2, CLCF1, CLCN2, CLDN14, CLP1, CLPP, CNTNAP1, COCH, COL11A2, COL6A1, COL6A2, COL6A3, CRB1, CRLF1, CRX, CST3, CSTB, CTC1, DAG1, DCAF8, DCDC2, DCTN1, DCX, DES, DIABLO, DIAPH1, DISC1, DNAJB2, DNAJB6, DRD3, DYNC1H1, DYNC2H1, DYSF, EFHC1, EGR2, ELOVL4, ELP4, EPM2A, ERCC6, ESPN, ESRRB, EXOSC3, EXOSC8, EXOSC9, EYA4, F12, FBLN5, FBXO7, FGD4, FGFR1, FIG4, FKRP, FKTN, FOXI1, FUS, FXN, GAB1, GABRA1, GABRB3, GAN, GARS1, GBA1, GDAP1, GH1, GIGYF2, GIPC1, GIPC3, GJB1, GJB2, GJB3, GJB6, GLI2, GLRA1, GLRB, GLUD2, GMPPB, GNB4, GPHN, GPSM2, GRHL2, GRIN2A, GRK1, GRM6, GRXCR1, GSDME, GUCY2D, HFE, HGF, HK1, HLA-DQB1, HMCN1, HNRNPDL, HSPB1, HSPB8, HTRA1, HTRA2, IFT80, IGHMBP2, ILDR1, IMPDH1, IMPG1, IMPG2, INF2, ITGA7, JPH1, KARS1, KCNA1, KCNJ10, KCNJ13, KCNQ2, KCNQ3, KCNQ4, KCNT1, KCTD17, KIF1A, KIF1B, KIF21A, KLHL7, KNL1, KRIT1, LAMA2, LAMB1, LARGE1, LCA5, LGI1, LHFPL5, LITAF, LMNA, LMX1A, LOXHD1, LRAT, LRP12, LRRK2, LRTOMT, MAPT, MARS1, MARVELD2, MCPH1, MECP2, MFN2, MFSD2A, MICAL1, MIR96, MME, MPDZ, MPO, MPZ, MSRB3, MT-CO1, MT-RNR1, MT-TS1, MT-TT, MTMR2, MYH14, MYH6, MYH7, MYH9, MYLK2, MYO15A, MYO3A, MYO6, MYO7A, MYOT, NAXD, NAXE, NDRG1, NEFH, NEFL, NHLRC1, NLGN3, NLGN4X, NMNAT1, NOS3, NOTCH2NLC, NR4A2, NRG1, NRXN1, NUP62, NYX, OTOA, OTOF, P2RX2, PAFAH1B1, PARK7, PAX6, PCDH15, PCLO, PDCD10, PDXK, PHC1, PHOX2A, PIK3CA, PINK1, PJVK, PLA2G6, PLAU, PLEC, PLK4, PMP22, PNKD, POLR3A, POLR3B, POMGNT1, POMT1, POMT2, POU3F4, POU4F3, PRICKLE1, PRKN, PRNP, PRODH, PROM1, PRPH, PRPH2, PRPS1, PRRT2, PRRX1, PRX, PSEN1, PSEN2, PTCH1, PTPRQ, RAB7A, RARS2, RAX2, RBM12, RD3, RDH12, RDX, REEP1, RELN, REST, RETREG1, RIPOR2, RPE65, RPGRIP1, RPGRIP1L, RYR1, S1PR2, SAG, SASS6, SBF1, SBF2, SCN2A, SCN8A, SCN9A, SELENON, SEPSECS, SERPINB6, SERPING1, SETX, SGCA, SGCE, SGCG, SH3TC2, SHANK3, SHH, SIGMAR1, SIX1, SIX3, SLC17A8, SLC1A1, SLC1A3, SLC25A46, SLC26A4, SLC26A5, SLC5A7, SLC6A5, SMN2, SNCA, SNCAIP, SNRPN, SOD1, SPATA7, SPG11, STIL, STN1, STRC, TARDBP, TBC1D23, TBL1Y, TBP, TBR1, TCAP, TECTA, TGIF1, TMC1, TMEM67, TMIE, TMPRSS3, TNPO3, TPRN, TRAPPC11, TRIM32, TRIM44, TRIOBP, TRPM1, TRPV4, TSEN15, TSEN2, TSEN34, TSEN54, TTC21B, TTN, TTR, TUBA1A, TUBB3, TUBGCP4, TUBGCP6, TULP1, UCHL1, USH1C, VAPB, VPS35, VPS53, VRK1, WDR19, WDR62, WDR81, WFS1, WHRN, WNK1, WT1, YARS1, ZIC2, ZNF335, ABCC1, ABHD16A, ACER3, ACTA1, ACTL6B, ADAM10, ADAM22, ADARB1, ADCY5, ADH1B, ADPRS, AFG3L2, AIMP2, ALOX5AP, ANAPC7, ANXA11, AOPEP, AP2M1, APC2, APOL2, APOL4, ARHGEF2, ARID2, ASH1L, ATG5, ATG7, ATP11A, ATP1A1, ATP1A2, ATP2B1, ATP6V0A1, ATP6V1A, ATXN8, BDP1, BPTF, BRAT1, C2, C9orf72, CACNA1B, CACNA1E, CACNA1G, CADM3, CAMK2A, CAMK2B, CAMK2G, CCDC88A, CCL2, CCNF, CDC40, CDC42BPB, CDH2, CDK19, CEACAM16, CELF2, CEP85L, CFAP43, CFB, CHD5, CHI3L1, CHP1, CHRNA7, CIC, CILK1, CLCN3, CLDN11, CLDN9, CLEC3B, CLRN2, CLTC, CNP, CNPY3, COASY, COL11A1, COMP, COMT, COPB2, CPLX1, CPSF3, CSF1R, CSNK2B, CTNNA2, CUL3, CUX2, CYB561, CYFIP2, CYLD, DAB1, DALRD3, DAOA, DCTN2, DEGS1, DHDDS, DHPS, DHX30, DHX37, DLG4, DLL1, DMD, DMXL2, DNM1L, DNMT3B, DOCK3, DPYSL5, DRD4, DRD5, DRP2, DTYMK, DYNC1I2, EIF2AK2, ELMOD3, EMC10, EPRS1, EPS8L2, ESRP1, EXOC2, EXOC7, EXOC8, EXOSC1, F2, F5, FAT2, FBP2, FBXO28, FDFT1, FGF13, FKBP5, FOXH1, FRG1, GABBR2, GABRA2, GABRA5, GABRB2, GABRD, GABRG2, GAD1, GBF1, GDAP2, GEMIN2, GEMIN4, GEMIN5, GNAI1, GNAO1, GNAQ, GNB2, GPRASP2, GRAP, GREB1L, GRIA1, GRIA2, GRIA4, GRIK2, GRIN1, GRM1, GRM7, HCN1, HCN4, HDAC4, HEPACAM, HID1, HPDL, HTR2A, IL6, INPP5K, INTS1, INTS8, JAG1, JAG2, JAM2, KAT5, KCNC2, KCND3, KCNMA1, KCNN2, KCNQ5, KCNT2, KDM4B, KDM6B, KIF14, KLF13, KMT2B, KMT5B, KRAS, LIPT2, LMAN2L, LMNB1, LMNB2, LNPK, LOC111365204, LRIF1, MACF1, MADD, MAP1B, MAPK8IP3, MCM3AP, MDH1, MED13, MED27, MINPP1, MORC2, MPV17, MPZL2, MT-CO2, MT-CO3, MT-CYB, MT-ND1, MT-ND5, MT-ND6, MT-TC, MT-TF, MT-TK, MT-TL1, MT-TQ, MT-TS2, MT-TV, MT-TW, MTHFD1, MTHFR, MTOR, MYORG, NAA15, NARS1, NARS2, NBEA, NCAPD2, NCAPD3, NCAPH, NCDN, NEUROD2, NEXMIF, NFASC, NKX6-2, NLRP3, NODAL, NOVA2, NR2E3, NRCAM, NSF, NTN1, NTNG2, NTRK2, NUAK2, NUP37, NUS1, OGDHL, OGT, OPTN, OXR1, PACS2, PARS2, PCDHGC4, PCYT2, PDE1C, PDGFB, PDGFRB, PDZD7, PHACTR1, PHIP, PI4KA, PIGA, PITRM1, PLAA, PLD3, PLS1, PLXNA1, PMP2, PNKP, PNPT1, POLR2A, POLR3K, POPDC3, PPIL1, PPIP5K2, PPP1R21, PPP2CA, PPP3CA, PRDM13, PRDX3, PRKAR1B, PRKCH, PRUNE1, PTCHD1, PTPN23, PUM1, RAB11B, RAC3, RALA, RALGAPA1, RBL2, RDH5, RFC1, RGS9, RGS9BP, RHOBTB2, RILPL1, RNF13, RNF170, RNF220, ROR1, RORB, RPL10, RRP7A, RTN4R, SAMD9L, SARS1, SCAPER, SCD5, SCN1A, SEC31A, SELENOI, SEMA6B, SET, SETD1A, SGCB, SGCD, SGPL1, SHANK2, SHMT2, SHQ1, SLC12A2, SLC12A5, SLC38A3, SLC44A4, SLC5A6, SLC6A4, SLC7A6OS, SLC9A7, SMC1A, SMCHD1, SMG8, SMN1, SMNDC1, SMPD4, SORD, SPATA5L1, SPNS2, SPOP, SPTBN4, SSBP1, STAG1, STEEP1, STUB1, SUPT16H, SVBP, SYN1, SYN2, TAF8, TAS2R16, TBC1D24, TBC1D2B, TBXT, THG1L, TIA1, TIAM1, TLCD3B, TLK2, TMEM106B, TMEM132E, TMEM222, TMEM43, TMEM63A, TMEM63C, TMX2, TNR, TOE1, TPH2, TPM2, TRAK1, TRAPPC14, TRAPPC4, TRAPPC6B, TREM2, TRIM36, TRIO, TRIP12, TRPM7, TRRAP, TTC5, TUBB4B, UBAP1, UBE4A, UBQLN2, UBTF, UFC1, UFM1, UGDH, UGP2, UQCRC1, USP45, USP7, VAMP2, VANGL1, VARS1, VCP, VPS11, VPS13D, VPS16, VPS35L, VPS41, VPS50, VPS51, VWA1, WARS1, WARS2, WASF1, WBP2, WDFY3, WDR45B, XBP1, XRCC1, YWHAG, ZMIZ1, ZNF142, ZNF292, ZNF526, ZSWIM6, AARS2, ABCG2, ACO2, ACSL4, ACTB, ADAM9, ADAMTS18, ADD3, ADGRG1, ADSS1, AIMP1, AKT3, ANKLE2, ANO3, AP1S2, AP3B2, AP4B1, AP4E1, AP4M1, AP4S1, AR, ARHGEF10, ARHGEF6, ARV1, ASCC1, ATAD3A, ATCAY, ATF6, ATL1, ATN1, ATOH7, ATP2B3, ATP7A, ATXN1, ATXN10, ATXN3, B3GALNT2, B4GAT1, BCAP31, BEAN1, BEST1, BICD2, BRF1, BRWD3, BVES, C1QTNF5, CABP2, CABP4, CACNA2D4, CACNG2, CAMTA1, CASQ1, CCDC22, CCND2, CD164, CDH15, CDK5, CERT1, CHAMP1, CHCHD10, CHCHD2, CHMP2B, CHST6, CIT, CLIC5, CNGA3, CNGB3, COL12A1, COL18A1, COL25A1, COL4A6, CPA6, CPT1C, CRYAB, CRYM, CSNK1D, CSNK2A1, CTDP1, CTNNA1, CWF19L1, CX3CR1, CYP2U1, CYP4V2, CYP7B1, DARS1, DCC, DDHD1, DDX3X, DEAF1, DENND5A, DEPDC5, DHTKD1, DIAPH3, DISC2, DLG3, DNAJC3, DNAJC6, DNAL4, DNMT1, DOCK7, DPP6, DRAM2, DST, EEF1A2, EEF2, EFEMP1, EIF2B1, EIF2B2, EIF2B3, EIF2B4, EIF2B5, EIF2S3, EMC1, EMX2, EPB41L1, EPS8, ERBB4, ERLIN1, FA2H, FBN2, FBXO38, FGF14, FLNC, FLVCR1, FOXG1, FOXP2, FRMD4A, FRMD7, FRMPD4, FRRS1L, FTSJ1, FUZ, GAL, GCNT2, GDF6, GDI1, GNAL, GNAT1, GNAT2, GNB1, GNB3, GOSR2, GPR143, GPR179, GRIN2B, GRN, GRXCR2, GUCA1A, HARS1, HCRT, HECW2, HERC2, HIKESHI, HIVEP2, HNRNPA1, HNRNPK, HNRNPU, HOMER2, HPCA, HSPB3, HSPD1, HSPG2, HTT, HUWE1, IL1RAPL1, IQSEC2, ITM2B, ITPR1, JPH3, KANK1, KATNB1, KCNC1, KCNC3, KCNH1, KCNV2, KIF2A, KIF4A, KIF5A, KIF5C, KIRREL3, KITLG, KLHL15, LAMA1, LAMC3, LDB3, LRIT3, MAPKAPK3, MATR3, MBD5, MCM2, MDH2, MED17, MED25, MEF2C, MET, MFSD8, MICU1, MID2, MOG, MR1, MRE11, MYF6, MYT1L, NACC1, NAGLU, NALCN, NDE1, NGF, NIPA1, NOL3, NOP56, NOTCH3, NPRL2, NPRL3, OPN1SW, OSBPL2, OTOG, PABPN1, PDE6B, PDE6C, PDE6H, PDK3, PDYN, PER2, PER3, PFN1, PIK3R2, PIK3R5, PITPNM3, PLA2G5, PLEKHG5, PMPCA, POC1B, POLR1C, POMK, PPP2R2B, PRDM12, PRDM8, PRKCG, PRKRA, PURA, PYCR2, RAB28, RAB39B, RAD51, RARS1, RBP4, RCBTB1, REEP2, RERE, RHO, RIMS1, RLBP1, RLIM, RNASET2, RNF168, RP1L1, RPGR, RPS6KA3, RTN2, RTN4IP1, RUBCN, RXYLT1, SCARB2, SCN10A, SCN11A, SCN1B, SEMA4A, SERPINI1, SLC20A2, SLC24A1, SLC33A1, SLC6A2, SLC9A1, SLITRK1, SMPX, SNX14, SPAST, SPEG, SPG21, SPTBN2, SRPX2, STAC3, STRADA, STX1B, SYNE1, SYNE4, SYNGAP1, SYNJ1, SYP, SYT14, SZT2, TAF1, TANGO2, TBC1D7, TBCD, TBCK, TBK1, TBL1XR1, TDP2, TEAD1, TELO2, TENM4, TGFBI, TGM6, THAP1, TIMP3, TM4SF20, TMEM240, TMTC3, TNC, TOR1A, TOR1AIP1, TPP1, TRIM2, TRIP4, TRPA1, TRPC3, TSPAN7, TSPEAR, TTBK2, TTLL5, TUBA4A, TUBA8, TUBB, TUBB2A, TUBB2B, TUBB4A, TUBG1, TYROBP, UBA1, UBA5, UNC80, USP27X, USP9X, VANGL2, VMA21, VPS13C, VWA3B, WASHC5, WWOX, XK, XPR1, XYLT2, YME1L1, ZBTB18, ZC4H2, ZDHHC15, ZFYVE27, ZNF711

Hereditary Cancers (148 Genes)

AIP, AKT1, ALK, ANKRD26, APC, ATM, ATR, AXIN2, BAP1, BARD1, BLM, BMPR1A, BRCA1, BRCA2, BRIP1, BUB1B, CASR, CDC73, CDH1, CDK4, CDKN1B, CDKN1C, CDKN2A, CEBPA, CEP57, CHEK2, CTC1, CTNNA1, CTRC, CYLD, DDB2, DDX41, DICER1, DIS3L2, DKC1, EGFR, EGLN1, ELANE, ENG, EPCAM, ERCC1, ERCC2, ERCC3, ERCC4, ERCC5, EXT1, EXT2, EZH2, FAN1, FANCA, FANCB, FANCC, FANCD2, FANCE, FANCF, FANCG, FANCI, FANCL, FANCM, FH, FLCN, GALNT12, GATA2, GPC3, HOXB13, HRAS, IKZF1, KIF1B, KIT, LZTR1, MAX, MBD4, MEN1, MET, MITF, MLH1, MLH3, MRE11, MSH2, MSH3, MSH6, MUTYH, NBN, NF1, NF2, NHP2, NOP10, NTHL1, PALB2, PALLD, PAX5, PDGFRA, PHOX2B, PIK3CA, PMS2, POLD1, POLE, POLH, POT1, PRKAR1A, PRSS1, PTCH1, PTCH2, PTEN, RAD50, RAD51C, RAD51D, RB1, RECQL4, REST, RET, RHBDF2, RNF43, RUNX1, SAMD9L, SBDS, SDHA, SDHAF2, SDHB, SDHC, SDHD, SLC45A2, SLX4, SMAD4, SMARCA4, SMARCB1, SMARCE1, SPINK1, SRP72, STK11, SUFU, TERC, TERT, TGFBR1, TINF2, TMEM127, TP53, TRIP13, TSC1, TSC2, TYR, VHL, WRAP53, WRN, WT1, XPA, XPC, XRCC2

Organ Health (934 Genes)

AAGAB, ABCA12, ABCA3, ABCC6, ACE, ACP5, ACTA1, ACTG1, ACTG2, ADAMTSL4, ADCY1, AGT, AGTR1, ALDH18A1, ALOX12B, ALOXE3, AMPD1, AQP2, ARHGAP31, ARX, ASB10, ATP2B2, ATP5F1E, ATP6V0A2, ATPAF2, AVPR2, BAG3, BCOR, BCS1L, BIN1, BMP4, BMPR1B, BTD, CAV3, CCDC50, CDC14A, CDH23, CEP290, CERS3, CFAP410, CFHR5, CFL2, CFTR, CHRDL1, CIB2, CISD2, CLCN1, CLCN5, CLCN7, CLDN14, CLMP, CLPP, CLRN1, COCH, COL11A1, COL11A2, COL2A1, COL4A3, COL4A4, COL4A5, COL8A2, COL9A1, COL9A2, COL9A3, COMP, CPAMD8, CRTAP, CRYAB, CSF2RA, CTSC, CUL3, CYP1B1, CYP4F22, DCDC2, DCX, DES, DGUOK, DIABLO, DIAPH1, DLAT, DLD, DLL3, DMP1, DNM2, DOCK6, DZIP1L, ECE1, ECEL1, EDN3, EDNRA, EDNRB, EFEMP2, ELP4, ENPP1, EOGT, ESPN, ESRRB, EXT2, EYA1, EYA4, FBLN5, FBN1, FBXL4, FGF23, FGFR1, FGFR2, FGFR3, FKBP10, FLNA, FLNB, FLNC, FN1, FOXC1, FOXE3, FOXI1, GAA, GAB1, GANAB, GDF3, GDF5, GDF6, GDNF, GIPC3, GJB2, GJB3, GJB6, GLIS2, GPSM2, GPX4, GRHL2, GRXCR1, GSDME, GUCY2C, GYG1, HARS1, HGF, HMOX1, HOXD13, IFITM5, IHH, ILDR1, INVS, IQCB1, IRF6, ITPR2, JAG1, KARS1, KBTBD13, KCNE1, KCNJ10, KCNQ1, KCNQ4, KLHL3, KLHL40, KRT12, KRT14, KRT3, KRT5, LAMA3, LAMB1, LAMB3, LAMC2, LDB3, LHFPL5, LIPN, LMX1A, LOXHD1, LRP4, LRP5, LRPPRC, LRTOMT, LTBP2, MARVELD2, MEOX1, MGME1, MIA3, MIR96, MITF, MMP1, MMP14, MMP2, MPV17, MSRB3, MSX1, MT-ATP6, MT-ND1, MT-ND4, MT-ND5, MT-ND6, MT-TA, MT-TC, MT-TD, MT-TF, MT-TH, MT-TK, MT-TL1, MT-TL2, MT-TM, MT-TN, MT-TQ, MT-TR, MT-TS2, MT-TT, MT-TV, MT-TW, MT-TY, MTM1, MTMR14, MUC5B, MYBPC1, MYH14, MYH3, MYH8, MYH9, MYO15A, MYO18B, MYO3A, MYO6, MYO7A, MYOC, MYOT, NALCN, NDUFS4, NEB, NEK8, NF2, NIPAL4, NOTCH2, NPHP1, NPHP3, NPHP4, NTF4, OCRL, OPN1LW, OPN1MW, OPTN, OTOA, OTOF, OVOL2, P2RX2, P3H1, PADI3, PAFAH1B1, PAM16, PARN, PAX3, PAX6, PC, PCDH15, PDHA1, PDHB, PDHX, PEX1, PEX6, PHEX, PIEZO2, PITX2, PITX3, PJVK, PKD1, PKD2, PKHD1, PNPLA1, POLR1C, POLR1D, POU4F3, PPIB, PTPRQ, PXDN, PYCR1, RBCK1, RBPJ, RDX, RELN, REN, REST, RET, RIPOR2, ROBO2, RPGRIP1L, RRM2B, RTEL1, S1PR2, SCN4A, SCNN1A, SCNN1B, SCNN1G, SDCCAG8, SDHA, SERPINB6, SERPINF1, SERPINH1, SFTPA2, SFTPB, SFTPC, SIX1, SIX5, SIX6, SLC17A8, SLC26A2, SLC26A4, SLC26A5, SLC34A1, SLC4A11, SLC9A3R1, SNAI2, SOS1, SOST, SOX10, SOX17, SOX2, SP7, SQSTM1, STRC, SUCLA2, SUCLG1, SUMO1, SURF1, TBX6, TCHH, TCOF1, TECTA, TERT, TGM1, TGM3, TIMM8A, TMC1, TMEM67, TMEM70, TMIE, TMPRSS3, TNNI2, TNNT1, TNNT3, TNXB, TP63, TPM2, TPM3, TPRN, TRAF3IP1, TRIM44, TRIOBP, TRIP11, TRPV4, TUBA1A, TWNK, USH1C, USH1G, VSX2, WDR19, WDR36, WFS1, WHRN, WNK1, WNK4, WT1, XYLT1, XYLT2, ZEB1, ZFPM2, ABCG8, ACTL9, ACTN2, AGBL1, AIFM1, AK7, ALPK1, AMTN, AR, ARMC2, ARR3, ASCC1, ASIP, ASPN, ASPRV1, ASTL, B2M, BPY2, BRDT, BTG4, C14orf39, C2CD6, C9, CALCR, CANT1, CASP14, CATIP, CATSPER2, CCDC134, CCDC62, CD55, CDY1, CDY2A, CEP112, CFAP251, CFAP43, CFAP44, CFAP47, CFAP58, CFAP65, CFAP69, CFAP70, CFAP91, CFI, CIBAR1, CILP, CLDN10, COG4, COL1A1, COL1A2, CPSF1, CREB3L1, CRYBA4, CTHRC1, CTRC, CTSB, CYP3A4, DACH1, DAZ1, DAZ2, DAZ3, DDRGK1, DDX3Y, DGKE, DLX5, DNAH1, DNAH10, DNAH17, DNAH2, DNAH8, DNAJB11, DNHD1, DNMBP, DSTYK, DZIP1, EPS8L3, ERBB3, ERCC1, ERCC6, EXOC6B, EXTL3, FANCM, FARSA, FARSB, FBXO43, FCGR2A, FGA, FHL1, FLG2, FREM2, FSIP2, FXR1, FZD2, FZD6, GATD3, GCNA, GFRA1, GGN, GJA1, GJB4, GLI1, GP1BA, GPR68, GREB1L, HACD1, HERC2, HES7, HESX1, HLA-A, HLA-B, HRURF, HSFY1, IFT172, IFT74, IKBKG, INSL3, IQCE, IRF4, ITGA6, ITGB4, KDELR2, KDM5D, KIAA0825, KITLG, KRT1, KRT10, KRT83, LBR, LFNG, LGR4, LMX1B, LOXL1, LOXL3, LRRK1, LSS, LTBP1, LTBP3, M1AP, MAMLD1, MAP2K1, MAP3K20, MATN3, MBTPS1, MBTPS2, MC1R, MEFV, MEIOB, MESD, MESP2, MET, METTL13, MIR140, MMP13, MOV10L1, MSH4, MSH5, MSMO1, MSR1, MT-CO1, MT-CO3, MT-CYB, MT-ND2, MT-ND4L, MT-RNR1, MT-TS1, MYH11, MYL1, MYL11, MYL2, MYMK, MYOD1, NEPRO, NHS, NRIP1, NRTN, OTX2, Oca.02, PANK4, PANX1, PAPPA2, PATL2, PAX2, PAX7, PDHA2, PDLIM4, PERCC1, PERP, PIK3CA, PLEC, PLEKHM1, PLVAP, PMFBP1, PMVK, PNLDC1, POLE, PORCN, POU3F4, PPP2R3C, PRDX3, PRICKLE3, PRKG2, PRPS1, PRSS2, PRY, PRY2, QRICH2, RAX, RBMY1A1, REC114, RELT, RNF212, RNU4ATAC, RPA1, RPL10L, RPS4Y2, RSPO2, SASH1, SDR9C7, SFRP4, SGMS2, SHOC1, SHOX, SIK3, SIX3, SLC10A7, SLC24A4, SLC26A1, SLC2A9, SLC36A2, SLC41A1, SLC45A2, SLC6A19, SLC6A20, SMARCAD1, SMPX, SOHLH1, SPAG17, SPEF2, SPINK2, SREBF1, STAG3, STX3, SULT2B1, SVIL, SYCP2, TBX4, TCF4, TDRD9, TEK, TENT5A, TERB1, TERB2, TEX14, TEX15, TFAP2A, TGFB1, THBS2, TMEM251, TMEM53, TONSL, TPCN2, TRIP13, TRPM4, TSGA10, TTC21A, TTC29, TUBA3D, TYR, TYRP1, UFSP2, UNC45B, VAX1, VCY, WEE2, WNT1, WNT2B, WNT7A, XKRY, XRCC2, ZC4H2, ZCCHC8, ZFHX4, ZP2, ZP3, ZPBP, ZSWIM7, ABCB6, ACAN, ACP4, ADAM10, ADGRE2, ADGRG2, AFF4, AGK, AKT1, ALDH1A3, ALX1, ALX3, ALX4, AMBN, AMER1, ANKS6, ANLN, ANO5, APCDD1, APOA1, AQP5, ASPH, ATP2A2, AURKC, B3GALT6, BANF1, BFSP1, BFSP2, BGN, BHLHA9, BMP1, BMPER, BMS1, CA12, CARD14, CATSPER1, CCDC78, CCN6, CD151, CDSN, CEP164, CEP83, CHMP4B, CHST8, CILK1, CKAP2L, CLEC7A, CNTN1, COG6, COL17A1, COL27A1, COL7A1, COPA, CRB2, CRIPT, CRYAA, CRYBA1, CRYBA2, CRYBB1, CRYBB2, CRYBB3, CRYGB, CRYGC, CRYGD, CRYGS, CSF2RB, CSTA, DCN, DDR2, DLL4, DLX3, DLX4, DNMT3A, DPY19L2, DSC3, DSG1, DSG4, DSPP, DST, DYM, EDA, EIF4A3, ELN, ENAM, EPCAM, EPHA2, ESCO2, EXPH5, FAM111A, FAM111B, FAM20A, FAM83H, FAN1, FBLN1, FDPS, FERMT1, FGF10, FGF16, FGF20, FLCN, FLG, FREM1, FTL, FYCO1, GCNT2, GGCX, GJA3, GJA8, GLB1, GLI3, GORAB, GPC6, GREM2, GSC, HPGD, HR, HSF4, HTR1A, IGSF3, INF2, INPPL1, IRAK4, ITGA3, ITGA8, ITGB6, KANK2, KERA, KIF22, KIF7, KLHL10, KLHL24, KLK4, KLLN, KRT16, KRT17, KRT2, KRT25, KRT6B, KRT6C, KRT71, KRT74, KRT81, KRT86, KRT9, KY, LEMD2, LIM2, LIPH, LMBR1, LMNA, LORICRIN, LPAR6, LRP1, LRP6, LRPAP1, LYZ, MAF, MAP3K7, MAPKBP1, MAPRE2, MARS1, MFRP, MIP, MMP19, MMP20, MMP9, MSTN, MSX2, MUC1, MVD, MYO1E, MYO5B, MYPN, NANOS1, NANS, NBAS, NCSTN, NEK9, NKX3-2, NLRP1, NOG, NOTCH1, NPR2, ODAPH, ORAI1, P3H2, P4HA2, PAX9, PCYT1A, PDE10A, PDGFRB, PIKFYVE, PITX1, PLCD1, PLCZ1, POLR1A, POMP, POP1, PRIMPOL, PRKAR1A, PRSS1, PRSS56, PSEN1, PSENEN, PTH1R, PTHLH, PTPN11, PUS3, PYROXD1, RAB33B, RBP4, RIN2, RPL21, RSPO4, RSPRY1, SALL2, SALL4, SCO2, SEC23B, SEC61A1, SEPTIN12, SERPINB7, SERPINB8, SF3B4, SHH, SIPA1L3, SLC17A9, SLC26A8, SLC39A5, SLC4A1, SLC9A3, SLURP1, SMOC2, SNRPE, SNX10, SPARC, SPATA16, SPATA5, SPINK1, SPINT2, ST14, STIM1, SUN5, SYCE1, SYCP3, TACSTD2, TAF4B, TAPT1, TBX15, TBX18, TBX22, TBXAS1, TDRD7, TENM3, TERC, TEX11, TGDS, TGFBI, TMEM38B, TMEM98, TMPRSS15, TRPC6, TRPV3, TSHZ1, TTC21B, TUBB, TUBB8, TWIST1, UBB, UBIAD1, UMOD, USP9Y, VIM, VSX1, WDR72, WNT10B, WNT3, WNT4, XPNPEP3, ZIC1, ZMYND15, ZNF141, ZNF423, ZNF644, ZNF687, ZNF750, ZP1, ZSWIM6

Syndromic Diseases (723 Genes)

ACTB, ACTG1, ACTG2, ACTN4, ADAMTS10, ADAMTS2, ADGRG1, AHI1, AKT1, APOC2, ARFGEF2, ARL13B, ARL6, B3GALT6, B4GALT7, B9D1, B9D2, BBS10, BBS12, BBS2, BBS5, BBS7, BBS9, BCOR, BSND, C1R, C1S, CC2D2A, CCDC103, CCDC28B, CCDC39, CCDC40, CCDC65, CCDC8, CCNO, CDH23, CENPF, CEP290, CFAP298, CFAP300, CHAT, CHRNA1, CHRNE, CISD2, CLCNKA, CLCNKB, CLRN1, COLQ, COX7B, CST6, CUL7, DNAAF1, DNAAF11, DNAAF2, DNAAF3, DNAAF4, DNAAF6, DNAH1, DNAH11, DNAH5, DNAI1, DNAI2, DNAJB13, DNAL1, DOK7, DRC1, DYNC2H1, EDA, EDAR, EDARADD, EDN3, EDNRB, EFL1, ELF4, EPS8L3, ERF, EYA1, FBN1, FGD1, FGF9, FGFR1, FGFR2, FGFR3, FKBP14, FLNA, GAS8, GDF5, GDF6, GFPT1, GNS, GPC3, GPC4, GPIHBP1, GRHL3, H19-ICR, HARS1, HCCS, HGSNAT, HMGA2, HRURF, HYDIN, IFNG, IFT122, IFT43, IGF2, INF2, INPP5E, IRF6, KCNJ1, KIF7, KRT16, KRT17, KRT6A, KRT6B, L1CAM, LAGE3, LMNA, LPL, LTBP2, MAGED2, MITF, MKS1, MMP14, MMP2, MSX2, MVK, MYO7A, NAA10, NAGLU, NDUFB11, NEK1, NIPBL, NME8, NOG, NPHP1, NPHP3, NPHS1, NPHS2, OBSL1, ODAD1, ODAD2, ODAD3, ODAD4, OFD1, OSGEP, PCDH15, PGAP2, PGAP3, PI4KA, PIGO, PIGV, PIGW, PIGY, PLAG1, PLK4, PLOD1, POLR1C, POLR1D, POR, PRDM5, RIPK4, ROR2, RPGRIP1L, RSPH1, RSPH3, RSPH4A, RSPH9, SCN4A, SF3B2, SGSH, SIX1, SLC12A1, SLC39A13, SMC1A, SMC3, SNAI2, SOX10, SPAG1, TBX5, TCF12, TCOF1, TCTN1, TCTN2, TMEM216, TMEM67, TNFAIP3, TP53RK, TPRKB, TRIM32, TRPC6, TSC1, TSC2, TTC8, TUBGCP4, TUBGCP6, TWIST1, UGT1A1, USH1C, USH1G, WDPCP, WDR19, WDR35, WDR73, WFS1, WT1, ZMYND10, ZNF469, AARS1, ABCA5, AEBP1, AFF3, AHSG, APC, ARF1, ARL3, ARMC9, ARSG, AVIL, BBS4, BCL11B, BICRA, BLTP1, BMP2, BMPR1B, BRD4, C1GALT1C1, CAPN15, CDH1, CDH11, CDKL5, CELA2A, CEP120, CEP41, CEP55, CHUK, CLCN4, CLIC2, CNKSR2, COL1A1, COL1A2, COL4A3, COL4A4, CPLANE1, CPLX1, CTBP1, CTNND1, CWC27, DACT1, DNAAF5, DNAH9, DNAJC21, DNASE2, DPF2, DVL1, DYNC2LI1, EIF2S3, ESCO2, EXOSC2, FAM149B1, FAM50A, FANCB, FAR1, FBXO11, FGFRL1, FLCN, FOXI1, FOXJ1, FRA16E, FREM2, GAS2L2, GATM, GLE1, GLRA2, GNAS, GON7, GTPBP2, GZF1, H1-4, HDAC8, HDC, HEPHL1, HS6ST2, HTT, HUWE1, IFT172, IFT57, IFT74, IFT81, IL6, INTU, IRF2BPL, ITPR1, KANK2, KCNJ10, KDM6A, KIAA0753, KIRREL1, KITLG, KRAS, LETM1, LMOD1, LRRC56, LSS, MAGI2, MAP1B, MARS1, MCIDAS, MCM5, MIF, MKKS, MSL3, MT-ATP6, MT-CO3, MT-ND1, MT-ND2, MT-ND3, MT-ND4, MT-ND5, MT-ND6, MT-TI, MT-TK, MT-TL1, MT-TL2, MT-TV, MT-TW, MTTP, MYH11, MYH3, MYL9, MYLK, MYO9A, NDUFAF6, NEK10, NKAP, NOS1AP, NSD2, NUP107, NUP133, NUP160, NUP85, NUP88, NXN, OTUD6B, PAX6, PDZD7, PIBF1, PIK3C2A, PIK3CA, PISD, POLA1, POLR1B, POLR3A, PTCH1, PTCH2, PTEN, QRICH1, RAB39B, RAB3GAP1, RAB5IF, RAC1, RAD21, RPL10, SBDS, SDCCAG8, SIX6, SLC25A1, SLC25A24, SLC5A7, SLCO2A1, SMARCC2, SMARCD1, SMO, SNRPN, SOX4, SREBF1, SRP54, STK36, SUFU, SYT1, SYT2, TARS1, TBC1D8B, TBCE, TCTN3, TFE3, TMEM107, TMEM138, TMEM218, TMEM231, TMEM237, TOGARAM1, TP73, TRIP11, TSPEAR, TTC12, TTC21B, TXNDC15, UBA1, VPS4A, WARS2, WDR1, WDR26, WDR4, WHCR, YRDC, YY1, ZNHIT3, ABCC2, ABCG5, ABCG8, ACP5, ADAMTS17, ADAT3, ADNP, AHDC1, ALPL, AMMECR1, AMPD2, ANK3, ANKRD11, ANTXR1, APOE, AR, ARHGDIA, ARID1A, ARL6IP1, ARMC5, ARX, ASCL1, ASNS, ASXL1, ATP13A2, ATP6AP2, ATP6V1B2, ATP8A2, ATR, ATRX, AUTS2, AXIN1, B4GALNT1, BBIP1, BGN, BMP4, BRPF1, BSCL2, C19orf12, C2CD3, CA8, CAPN1, CASK, CAST, CCDC88A, CCT5, CD96, CDC45, CDC6, CDH3, CDT1, CENPJ, CEP104, CEP63, CFAP418, CHRM3, CHRNG, CIB2, CNTNAP2, COG5, COL10A1, COL11A1, COL2A1, COL4A5, CTCF, CTNNB1, CTSC, CUL4B, CYP26B1, CYP26C1, DCTN1, DDHD2, DDX59, DEAF1, DGKE, DHODH, DIAPH1, DIS3L2, DLX3, DNA2, DPH1, DSG1, DSP, DSTYK, DVL3, DYM, DYNC2I1, DYNC2I2, DYNLT2B, DYRK1A, DYRK1B, EDNRA, EFNB1, EHHADH, ELOVL4, EML1, EMP2, ENTPD1, ERCC6, ERLIN2, ERMARD, EVC, EVC2, FAM111A, FGF10, FIG4, FKBP10, FMR1, FOXE1, FOXP1, FREM1, GABRB3, GATA3, GATAD2B, GBA2, GDNF, GJA1, GJB2, GJB6, GJC2, GMNN, GMPPA, GPT2, GRHL2, GRIA3, GRIN2B, GTF2E2, GUF1, H19, HACE1, HDAC6, HMGB3, HMX1, HNRNPH2, HOXA1, HOXA13, HOXB1, HOXC13, HPGD, HSD11B2, HSPA9, IARS1, IBA57, IFIH1, IFT140, IFT27, IFT52, IFT80, IGBP1, IGF1, IGF1R, IGFALS, IL11RA, INSR, KAT6B, KATNIP, KCTD1, KDF1, KDM5C, KIAA0586, KIDINS220, KIF14, KIF1C, KIFBP, KLC2, KREMEN1, KRT14, KRT74, KRT85, LAMA3, LAMB2, LARP7, LAS1L, LEMD3, LMF1, LONP1, LZTFL1, MAB21L2, MAF, MAG, MBTPS2, MECP2, MED12, MEGF8, MIR17HG, MIR184, MIR204, MPLKIP, MPZ, MSX1, MYH8, MYH9, NECTIN1, NECTIN4, NEDD4L, NEXMIF, NFIX, NIN, NLRC4, NONO, NPR2, NR2F1, NSDHL, NSMCE2, NT5C2, NUP205, NUP93, OPHN1, ORC4, ORC6, OTX2, P4HB, PACS1, PAX2, PAX3, PCDH19, PDE3A, PDE6D, PHF8, PITX2, PKP1, PLCB1, PLCG2, PLOD2, PMP22, PNPLA6, POC1A, POGZ, PPP2R1A, PPP2R5D, PRPS1, PSAP, PTDSS1, PTPRO, QARS1, RARB, RBBP8, RBMX, RDH11, RHBDF2, RIGI, RNF113A, RNF135, ROGDI, RTTN, RUNX2, SALL4, SAMD9, SATB2, SCN9A, SCNN1G, SEC24D, SETBP1, SETD2, SGO1, SH3PXD2B, SHROOM4, SIN3A, SIX5, SLC2A2, SLC34A1, SLC4A11, SLC6A3, SLITRK1, SLITRK6, SMARCA2, SMARCA4, SMARCAD1, SMARCB1, SMARCE1, SMCHD1, SNAP29, SNRPB, SOX11, SOX2, SOX3, SPART, SPECC1L, SPG11, SPTAN1, ST3GAL3, STAMBP, SYN1, TAF1, TBX22, TBX3, TCF4, TECPR2, TFG, TGFB1, THOC2, TMCO1, TP63, TRAIP, TRIO, TRPS1, TWIST2, TYR, UBE2A, UBE3A, UBE3B, UPF3B, USP18, USP9X, VAX1, VPS37A, WDR81, WHRN, WNT10A, WNT5A, WNT7A, ZBTB20, ZDHHC9, ZFYVE26, ZIC3, ZMYND11

Other Diseases (925 Genes)

ABCC9, ACE, ACTC1, ACTN2, AIP, ALX4, ANKRD1, ARHGAP26, ARMC5, ASPA, ATP1A2, ATRX, AVP, BAG3, BCL10, BLM, BMP2, BRAF, BRCA2, CACNA1A, CASP10, CAV3, CBL, CBX2, CDH1, CDH11, CEP250, CEP78, CFTR, CHRNA4, CLCN1, CLIC2, CNGA3, CNGB3, COL6A2, COL6A3, CR2, CREBBP, CRYAB, CSRP3, CTNNB1, CYP2A6, DCHS1, DES, DGUOK, DHH, DSC2, DSG2, DSP, EDN1, EDNRA, ELP1, EP300, EPO, ERBB2, ERBB3, ESR1, EWSR1, EYA4, FANCC, FAT4, FCGR2A, FKTN, GABBR2, GATA1, GBA1, GCH1, GIMAP5, GLB1, GNAI3, GNAS, GNAT2, GPC3, GPC4, H19, H19-ICR, H3-3A, H3-3B, H4C11, H4C3, HCN4, HEXA, HFE, HRAS, HSPG2, IDH1, IER3IP1, IFNL3, IGF2, IL1RN, IL6, IL6ST, IRF5, JPH2, JUP, KCNK18, KCNN4, KIF15, KRAS, KRT74, LAMA4, LBX1, LDB3, LIFR, LMNA, LPAR6, LZTR1, MAP2K1, MAP3K1, MCOLN1, MLH3, MSH3, MSH6, MSX2, MYBPC3, MYH6, MYH7, MYL2, MYL3, MYLK2, MYO1H, MYOZ2, NEXN, NF1, NR0B1, NR5A1, NRAS, PDCD1, PHOX2B, PI4KA, PIEZO1, PIGA, PIGN, PIGQ, PIGT, PKP2, PLCB4, PLN, PON1, POU6F2, PRF1, PRKAG2, PRKAR1A, PRLR, PSEN1, PSEN2, PTCH2, PTPN11, PTRH2, RAB39B, RAD54B, RAD54L, RAF1, RBM20, RIT1, RMRP, RYR2, SCN1A, SCN5A, SCUBE3, SDHA, SGCD, SHOC2, SLC6A3, SMPD1, SOD2, SOS1, SPECC1L, SPR, SPRED1, SRY, STAT3, STAT4, SUFU, TAFAZZIN, TCAP, TGFB1, TGFB3, TH, TLR5, TMEM43, TMPO, TNF, TNNC1, TNNI3, TNNT2, TP53, TPM1, TTC7A, TTN, USP8, VCL, VEGFA, WT1, YARS1, YIPF5, A2ML1, AARS1, ABCA2, ABCC11, ABCC8, ABCG2, ABL1, ACBD5, ACKR1, ACKR3, ACTL6B, ACTN3, ADCY3, ADCY5, ADRB1, ADRB2, AGO2, AGTPBP1, AGXT2, AKT1, AKT2, ALOX5, ANGPTL4, ANKRD17, ANXA5, AP1B1, AP1G1, AP1S3, APC, APOA1, APOL1, ARL2, ASPN, ASXL1, ATAD3A, ATN1, ATP5MC3, AURKA, AXIN1, AXIN2, B4GALNT2, BAP1, BAX, BCAS3, BCORL1, BCR, BHLHE41, BICC1, BNC2, BTNL2, BUB1, BUB1B, C5, CACNA1S, CACNA2D2, CALR, CARS1, CASP8, CATSPER2, CBFB, CCDC32, CCDC47, CCL11, CCL2, CCND1, CCNK, CD209, CD244, CD36, CDH2, CDK10, CDK8, CEBPA, CENPT, CFAP410, CFHR3, CHD1, CHD3, CHD8, CHEK2, CHI3L1, CHIC2, CHRNA3, CHRNA5, CHST11, CIB1, CIITA, CISH, CLCN6, CLDN2, CLEC1A, CLEC7A, CNOT1, CNOT2, CNOT3, CNTNAP2, COL18A1, COL2A1, COL4A1, COL4A2, COL4A3, COL4A4, COL6A1, COLEC10, COPB1, COPB2, COQ2, CPE, CR1, CRY1, CSF1R, CSGALNACT1, CTBP1, CTLA4, CTU2, CUBN, CUX1, CX3CR1, CXCR2, DAAM2, DBR1, DCC, DDB1, DDR2, DDX6, DHX16, DICER1, DLC1, DNAAF4, DNASE1, DNMT3A, DONSON, DSG3, EDAR, EDN3, EDNRB, EED, EGLN1, EIF2AK1, EIF2AK2, EIF4E, EIF5A, ELN, ELOVL1, EN1, ENPP1, ERCC6, ERGIC1, ESPN, ETV6, EXOSC5, F13A1, F2, F5, F7, FAAH, FASLG, FBXL3, FBXW11, FCGR2B, FFAR4, FGFR2, FGFR3, FITM2, FLCN, FLT3, FLT4, FOXD3, FOXE3, FRZB, FSHR, FTO, FUT2, FUT3, G6PD, GARS1, GATA2, GCGR, GCK, GCKR, GCLC, GCLM, GDF11, GDNF, GGPS1, GJA5, GJA8, GLCCI1, GLS, GNB1, GPD2, GPR101, GSTZ1, GSX2, GYPA, GYPB, GYPC, H4C5, H4C9, HBB, HCN2, HDAC4, HLA-B, HLA-C, HLA-DQA1, HLA-DQB1, HLA-DRB1, HLA-G, HMGA1, HNF1A, HNF1B, HNF4A, HNMT, HS2ST1, HTR2A, HYOU1, ICAM1, IFNAR2, IFNG, IFNGR1, IGF2BP2, IGF2R, IL10, IL10RB, IL13, IL18BP, IL1B, IL23R, IL6R, IL7, IPO8, IQSEC1, IRAK3, IREB2, IRF1, IRGM, IRS1, IRS2, ITGB3, ITPA, ITPR3, JAG1, JAK1, JAK2, KARS1, KAT8, KCNA4, KCNJ11, KCNJ16, KCNJ18, KCNK4, KCNMA1, KCNN3, KDM3B, KIDINS220, KIF3B, KIF5A, KIT, KLF6, KLHDC8B, KMT2C, KMT2E, KRT75, LACC1, LEMD2, LGALS2, LGI4, LGR4, LIG4, LIMA1, LIPC, LMBRD2, LMNB2, LPA, LPP, LRP5, LRP8, LRRC32, LTA, MAB21L1, MADD, MAP3K8, MAPK8IP1, MAPKAPK5, MARK3, MAST1, MATN3, MC1R, MC3R, MC4R, MCC, MCM3AP, MDM2, MECP2, MED12, MED12L, MEIS2, MET, MIAT, MIEF2, MLLT10, MLXIPL, MMP3, MN1, MORC2, MPIG6B, MPL, MRAP2, MRPS25, MTHFD1, MTNR1B, MTX2, MUC7, MUTYH, MYF5, MYMX, MYO9B, MYOCD, MYRF, NCAPG2, NCR3, NDUFB10, NEB, NEK1, NEMF, NEUROD1, NFE2L2, NFIA, NFIB, NFKBIL1, NHLRC2, NLGN1, NLGN3, NLGN4X, NLRP1, NLRP3, NOS2, NPC1L1, NPM1, NPR3, NPSR1, NR2F2, NR4A2, NRROS, NSD2, NUDT2, NUMA1, NUP188, NUP214, NUTM2B-AS1, OAS1, OGG1, OLR1, OPA1, OPN1MW, OPTN, OR2J3, OTUD5, P4HTM, PAICS, PAK1, PAX4, PBRM1, PBX1, PCDH12, PCGF2, PCSK1, PDE2A, PDE6C, PDE6H, PDGFRA, PDGFRL, PDX1, PHF21A, PICALM, PIGF, PIGG, PIGU, PIK3CA, PKDCC, PLA2G2A, PLA2G7, PLCB3, PLD1, POLR3GL, POU3F3, POU4F1, PPARG, PPM1D, PPP1R12A, PPP1R3A, PPP2R1B, PPP2R3C, PPP3CA, PRCC, PRDM13, PRKACA, PRKACB, PRKAG3, PRKN, PRR12, PSAP, PSMA6, PSMC3, PSMD12, PTCHD1, PTGDR, PTGER2, PTPN1, PTPN12, PTPN22, PTPRJ, PUS7, RAD21, RAD51C, RARA, RASA1, RB1, RELA, RET, RETN, RHOA, RHOH, RIC1, RIMS2, RIPK1, RNF2, RNF212, RORA, RPL10, RPL13, RPS23, RRM2B, RSPO2, RUNX1, SATB1, SCARB1, SCGB3A2, SCYL2, SELENBP1, SERPINA7, SETD1A, SETD1B, SF3B1, SFTPA1, SH2B3, SH3GL1, SH3TC2, SHANK2, SIAE, SIAH1, SLC11A1, SLC12A2, SLC17A3, SLC19A1, SLC22A18, SLC22A4, SLC25A42, SLC2A2, SLC30A8, SLC39A14, SLC44A1, SLC45A1, SLC4A1, SLC51B, SLC5A6, SLC6A6, SLC9A9, SMARCA2, SMO, SNORA31, SOCS1, SOD1, SORT1, SOX6, SP110, SPEN, SPRY2, SPTBN1, SRC, SRCAP, STAG2, STAT2, STRC, STX3, SUZ12, SYNE1, TAL1, TAL2, TANC2, TAOK1, TAS2R16, TASP1, TBK1, TBR1, TBX2, TCF20, TCF7L2, TERT, TET2, TET3, TIRAP, TKFC, TLR2, TLR7, TMC6, TMC8, TMEM260, TMEM94, TNFRSF1A, TNFSF4, TNPO2, TNRC6B, TOP2B, TOP3A, TOR1A, TPH2, TRAF3IP2, TRAF7, TRAPPC12, TRAPPC2L, TREX1, TRIM71, TRIM8, TRPV4, TRRAP, TTC26, TUBB6, TUBGCP2, TULP3, UBA2, UBR7, UCHL1, UCP2, UGT1A1, UGT2B17, UNC45A, VHL, WBP11, WDR37, WDR4, WFS1, WIPI2, WLS, WWC1, XPNPEP2, YIF1B, ZBTB7A, ZFAT, ZFHX2, ZIC1, ZMYM2, ZNF365, ZNF407, ZNF462, ZNF699, ZPR1, A4GALT, ACACA, ACAT2, ADCY6, ADGRG6, ADGRV1, ADIPOQ, ARCN1, ARNT2, ASXL2, ASXL3, ATP6V1B2, BCL11A, BHLHA9, C12orf57, CAPN5, CAV1, CD207, CD2AP, CD4, CDCA7, CDK13, CHD4, CHUK, CNNM4, CNTNAP1, COLEC11, CPN1, CRB2, CYP2B6, DDX11, DIP2B, EBF3, ECE1, EGFR, EHMT1, EIF4G1, ELANE, EMG1, ERF, EYA1, FBN1, FGF23, FGF5, FIBP, FLVCR2, FN1, FREM1, FUT1, FUT6, GALNT3, GDF5, GFI1, GHR, GIGYF2, GLDN, GLE1, GNB5, GPR88, HAGH, HELLS, HERC1, HOXA2, HOXD10, HP, HPSE2, HTRA2, HYLS1, INPP5E, INSR, IRF6, IRX5, ITCH, JAM3, KCNK9, KDM1A, KIF7, KL, KLF1, KLK1, KMT2A, KPTN, L1CAM, LHCGR, LRIG2, MASP1, MED25, MIR2861, MITF, MKKS, MYBPC1, NALCN, NAT8L, NEK8, NEK9, NFIX, NHS, NKX2-1, NOD2, NPHP3, NPR2, NRXN1, NSMCE3, NTRK2, OCLN, PADI6, PANK2, PAX1, PAX3, PDGFRB, PGR, PHYKPL, PIEZO2, PIP5K1C, PLEKHG2, PLOD3, PLS3, PNP, POLD1, POLE, PPA2, PPP1R15B, PRG4, PRKD1, PROZ, PRRX1, PTEN, PTH1R, RBBP8, RNF125, RNF139, ROBO3, SCARF2, SEC23A, SETD5, SLC10A2, SLC25A32, SLC30A2, SLC33A1, SLC49A4, SMG9, SMOC1, SNIP1, SNORD118, SOBP, SON, SOX5, SPTA1, SQSTM1, SRD5A3, TAF2, TAF6, TBCE, TBX21, THOC6, TLE6, TRMT10A, TRNT1, TTI2, TWIST1, TXNL4A, VANGL1, VSX1, WAC, WDPCP, XIST, YAP1, YY1AP1, ZBTB16, ZBTB24, ZBTB42, ZNF148

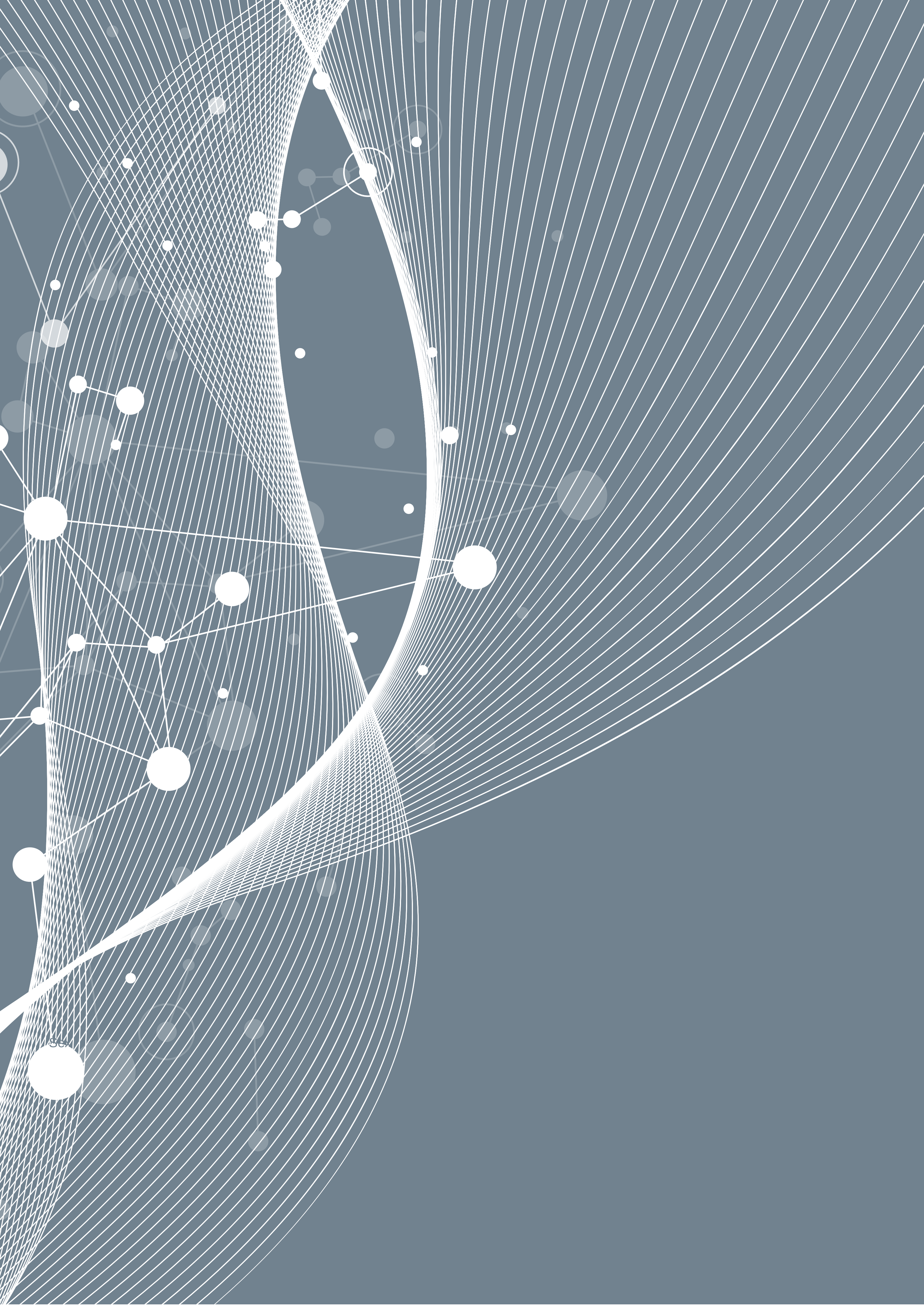

Supplement: Supplementary 1 — Figs. S1 to S10 Tables S1 to S4 Data S1 to S6 [file csbj.0011.f1.zip › Supplementary Material 2.pdf]
