# Supplementary material for: GenRiskPro: A Comprehensive Whole-Genome Sequencing Analysis Platform for Clinical and Wellness Applications
Source: Comput Struct Biotechnol J. 2026 Mar 6;35(2):0011. doi: 10.34133/csbj.0011 (PMC13394978; doi:10.34133/csbj.0011)
Supplement: Supplementary 1 — Figs. S1 to S10 Tables S1 to S4 Data S1 to S6 [file csbj.0011.f1.zip › Supplementary Material 3.pdf]

Benchmarking of the in-silico predicted scores

Methods
To evaluate the performance of our variant prioritisation thresholds, we extracted all variants with ClinVar assertions from the annotated VCF file (clinvar_20240611.annotated.vcf.gz). Only variants classified as “Pathogenic”, “Likely_pathogenic”, “Benign”, or “Likely_benign” were retained. For each variant, we parsed the VEP CSQ field to obtain the following in-silico scores: REVEL (REVEL_score), BayesDel with and without allele frequency (BayesDel_addAF_score, BayesDel_noAF_score), AlphaMissense pathogenicity score (am_pathogenicity), dbscSNV AdaBoost and random forest scores (ada_score, rf_score), and SpliceAI donor/acceptor scores (SpliceAI_pred_DS_AG, DS_AL, DS_DG, DS_DL). When multiple transcripts were present, the maximum value across transcripts was taken for each score.

For each score, a binary prediction was generated using the thresholds described in the main text (REVEL > 0.75, BayesDel_addAF > 0.0692655, BayesDel_noAF > −0.0570105, AlphaMissense > 0.5, dbscSNV Ada or rf > 0.6, any SpliceAI DS score > 0.5). A combined prediction was defined as a variant exceeding any of these thresholds (OR logic). Sensitivity and specificity were calculated against the ClinVar labels (pathogenic/likely pathogenic = positive, benign/likely benign = negative).

To visualise the discriminatory power and the effect of the OR logic, we generated parallel coordinate plots for missense and splicing scores separately. For each plot, 500 pathogenic and 500 benign variants were randomly sampled, and each score was normalised to the [0,1] interval (higher values indicate higher predicted deleteriousness). Thresholds were projected onto each axis as black triangles with the original threshold value annotated. The resulting plots illustrate that many pathogenic variants cross at least one threshold line, while benign variants tend to remain below all thresholds.

Results
The combined "OR" logic demonstrated superior performance in capturing pathogenic variants compared to any single tool alone. While individual tools showed varying levels of sensitivity (ranging from 3.5% to 40.8%), their combination through the OR rule substantially improved the detection of known pathogenic variants, increasing sensitivity by 15.3 percentage points over the best-performing individual predictor. This improvement reflects the complementary nature of the different algorithms, each capturing distinct subsets of pathogenic variants based on their underlying predictive principles.

Importantly, this enhanced sensitivity was achieved while maintaining high specificity (98.0%), confirming that the OR logic does not come at the cost of excessive false positives. The parallel coordinate plots (Figure 1 and Figure 2) visually illustrate this complementarity: many pathogenic variants that fall below the threshold for one tool are captured by another, whereas benign variants consistently remain below all thresholds.

Predictor	TP	FP	TN	FN	Sensitivity	Specificity	PPV	NPV
REVEL	9294	384	1,077,653	258,100	0.035	0.9996	0.960	0.807
BayesDel_addAF	109,171	7,752	1,070,285	158,223	0.408	0.9928	0.934	0.871
BayesDel_noAF	108,173	12,220	1,065,817	159,221	0.405	0.9887	0.898	0.870
AlphaMissense	45,492	7,002	1,071,035	221,902	0.170	0.9935	0.867	0.828
dbscSNV (ada or rf)	37,781	3,374	1,074,663	229,613	0.141	0.9969	0.918	0.824
SpliceAI (any DS)	39,654	3,257	1,074,780	227,740	0.148	0.9970	0.924	0.825
Combined (OR)	149,899	21,812	1,056,225	117,495	0.561	0.9798	0.873	0.900

Table 1. Performance metrics of in-silico thresholds on ClinVar variants. True positives (TP), false positives (FP), true negatives (TN), false negatives (FN), sensitivity, specificity, positive predictive value (PPV), and negative predictive value (NPV) are reported for each prediction tool using the thresholds defined in the main text. The combined "OR" rule indicates a variant predicted as deleterious if it exceeds any of the individual thresholds. Analysis was performed on 267,394 pathogenic/likely pathogenic (P/LP) and 1,078,037 benign/likely benign (B/LB) ClinVar variants.

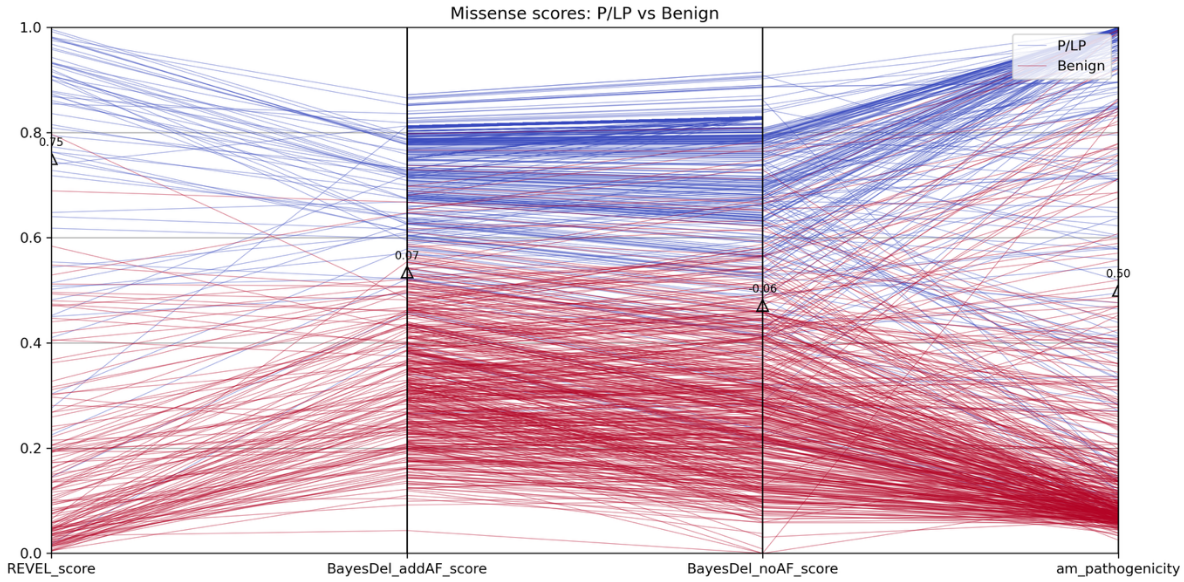

Figure 1. Parallel coordinate plot of missense in-silico scores for ClinVar pathogenic and benign variants. Scores (REVEL, BayesDel_addAF, BayesDel_noAF, AlphaMissense pathogenicity) were normalised to the [0,1] range, with higher values indicating greater predicted deleteriousness. Each line represents one variant, coloured by ClinVar classification (red: pathogenic/likely pathogenic; blue: benign/likely benign). Black triangles mark the position of the applied thresholds (original values shown above each axis). The plot demonstrates that many pathogenic variants exceed at least one threshold, whereas benign variants rarely do, supporting the OR-logic used for prioritisation.

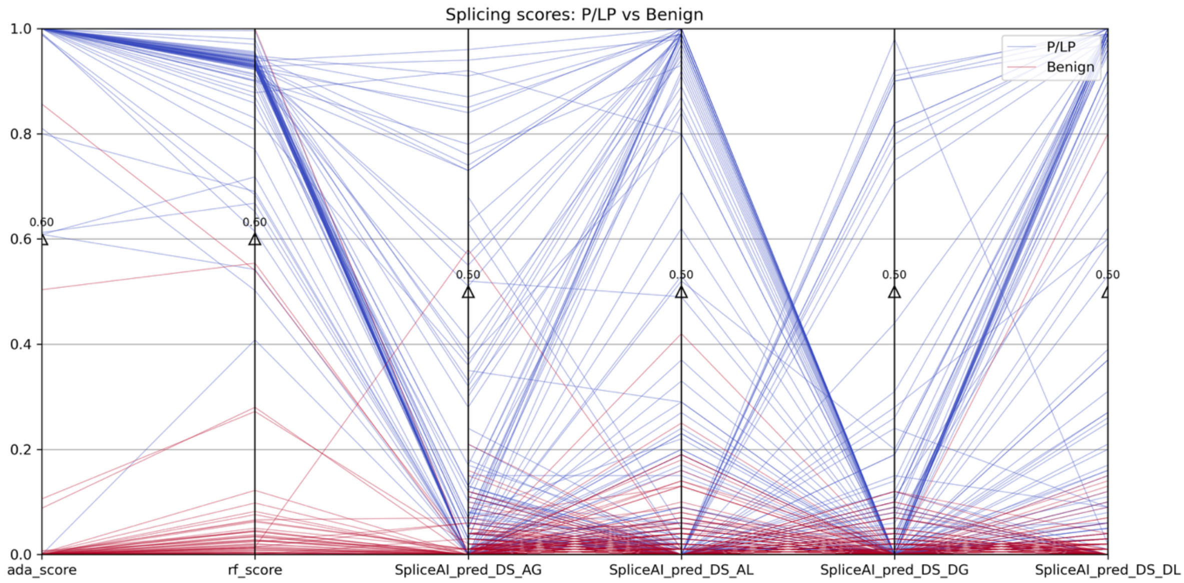

Figure 2. Parallel coordinate plot of splicing-related in-silico scores for ClinVar pathogenic and benign variants. Scores (dbscSNV Ada, dbscSNV rf, SpliceAI DS_AG, DS_AL, DS_DG, DS_DL) were normalised and displayed as in Figure S1. Thresholds (0.6 for dbscSNV scores, 0.5 for SpliceAI) are indicated by black triangles. The visualisation confirms that the combination of these scores captures variants with potential splice-altering effects while maintaining high specificity.
