# Supplementary material for: GenRiskPro: A Comprehensive Whole-Genome Sequencing Analysis Platform for Clinical and Wellness Applications
Source: Comput Struct Biotechnol J. 2026 Mar 6;35(2):0011. doi: 10.34133/csbj.0011 (PMC13394978; doi:10.34133/csbj.0011)
Supplement: Supplementary 1 — Figs. S1 to S10 Tables S1 to S4 Data S1 to S6 [file csbj.0011.f1.zip › Supplementary Table 2_scoring.docx]

**Supplementary Table 2. Scoring design of clinical risk variants by integrating variant pathogenicity and gene-disease associations**

3-1. General scores setting

| **Scoring Criteria** | **Sub-category** | **Score** |
| --- | --- | --- |
| **Variant-centric scoring** | |  |
| 1a. ClinVar variants | Pathogenic | 5 |
|  | Likely_pathogenic | 4 |
|  | Conflicting (P & VUS) | 2 |
|  | Conflicting (LP & VUS) | 1 |
|  | Not P/LP/CPLP | 0 |
| 1b. Predicted risk variants | pLoFs by VEP | 3 |
|  | Other in-silico predicted risk variants | 2 |
| **Gene-disease associations scoring** | |  |
| High confidence | | 10 |
| Moderate confidence | | 5 |
| Low confidence | | 0 |

3-2. ClinVar variants submitted diseases name matching with GeneRiskDB

| **Gene-disease pairs** | **Gene Match** | **Disease Match Type** | **Total Score** | **Output Logic** |
| --- | --- | --- | --- | --- |
| High confidence | Yes | Exact disease ID/names | + 10 | keep record and adding scores |
| Moderate confidence | Yes |  | + 5 |  |
| High confidence | Yes | Word subset for partial name matching | + 10 |  |
| Moderate confidence | Yes |  | + 5 |  |
| Low confidence | Yes | Any | + 0 | No gene-centric score, but keep record as low confidence |
| Gene Not in GenRiskPro | No | N/A | + 0 |  |
| Intergenic Variant | N/A | N/A | + 0 |  |
