# Supplementary material for: GenRiskPro: A Comprehensive Whole-Genome Sequencing Analysis Platform for Clinical and Wellness Applications
Source: Comput Struct Biotechnol J. 2026 Mar 6;35(2):0011. doi: 10.34133/csbj.0011 (PMC13394978; doi:10.34133/csbj.0011)
Supplement: Supplementary 1 — Figs. S1 to S10 Tables S1 to S4 Data S1 to S6 [file csbj.0011.f1.zip › Supplementary Table 4_variants.docx]

**Table S4. The summary of identified variants, categorized by the AFs in the TR and the SW cohort**

|  | **Cohort** | **Total** | **AFs in the cohort** | | | | **AFs in public databases (Maximum in sub-populations)** | | | | |
| --- | --- | --- | --- | --- | --- | --- | --- | --- | --- | --- | --- |
|  |  |  | **Singletons (AC=1)** | **Rare**  **(AC >1, AF<=0.01)** | **Low frequency (0.01<AF<=0.05)** | **Common (AF>0.05)** | **No public AF data** | **Rare**  **(MAX_AF <=0.01)** | **Low Freq**  **(0.01<MAX_AF<=0.05)** | **Common**  **(MAX_AF>0.05)** |  |
| **All variants** | **TR (n=275)** | 35,725,453 | 12,774,164 | 7,794,154 | 5,190,846 | 9,966,289 | 6,750,593 | 7,553,401 | 5,585,906 | 15,830,688 |  |
| **Autosomal variants** |  | 34,510,548 | 12,514,101 | 7,523,359 | 5,009,598 | 9,463,490 | 6,488,718 | 7,299,191 | 5,399,087 | 15,318,979 |  |
| **Novel variants** |  | 5,296,948 | 3,476,397 | 1,313,827 | 322,838 | 183,886 | 5,296,948 | / | / | / |  |
| **All variants** | **SW**  **(n=101)** | 20,085,548 | 5,188,952 | 1,598,278 | 3,866,306 | 9,432,012 | 720,920 | 2,861,118 | 3,242,184 | 13,259,520 |  |
| **Autosomal variants** |  | 19,346,483 | 5,018,167 | 1,495,047 | 3,711,417 | 9,121,852 | 675,241 | 2,739,183 | 3,120,157 | 12,810,176 |  |
| **Novel variants** |  | 505,284 | 437,525 | 27,187 | 16,156 | 24,416 | 5,05,284 | / | / | / |  |
